# Environmental transcriptome analysis reveals physiological differences between biofilm and planktonic modes of life of the iron oxidizing bacteria *Leptospirillum spp. *in their natural microbial community

**DOI:** 10.1186/1471-2164-11-404

**Published:** 2010-06-24

**Authors:** Mercedes Moreno-Paz, Manuel J Gómez, Aida Arcas, Víctor Parro

**Affiliations:** 1 Department of Molecular Evolution, Centro de Astrobiología (INTA-CSIC), Carretera de Ajalvir km 4, Torrejón de Ardoz, 28850 Madrid, Spain

## Abstract

**Background:**

Extreme acidic environments are characterized by their high metal content and lack of nutrients (oligotrophy). Macroscopic biofilms and filaments usually grow on the water-air interface or under the stream attached to solid substrates (streamers). In the Río Tinto (Spain), brown filaments develop under the water stream where the Gram-negative iron-oxidizing bacteria *Leptospirillum *spp. (*L. ferrooxidans *and *L. ferriphilum*) and *Acidithiobacillus ferrooxidans *are abundant. These microorganisms play a critical role in bioleaching processes for industrial (biominery) and environmental applications (acid mine drainage, bioremediation). The aim of this study was to investigate the physiological differences between the free living (planktonic) and the sessile (biofilm associated) lifestyles of *Leptospirillum *spp. as part of its natural extremely acidophilic community.

**Results:**

Total RNA extracted from environmental samples was used to determine the composition of the metabolically active members of the microbial community and then to compare the biofilm and planktonic environmental transcriptomes by hybridizing to a genomic microarray of *L. ferrooxidans*. Genes up-regulated in the filamentous biofilm are involved in cellular functions related to biofilm formation and maintenance, such as: motility and quorum sensing (*mqsR, cheAY, fliA, motAB*), synthesis of cell wall structures (*lnt, murA, murB*), specific proteases (*clpX/clpP*), stress response chaperons (*clpB, clpC, grpE-dnaKJ, groESL*), etc. Additionally, genes involved in mixed acid fermentation (*poxB*, *ackA*) were up-regulated in the biofilm. This result, together with the presence of small organic acids like acetate and formate (1.36 mM and 0.06 mM respectively) in the acidic (pH 1.8) water stream, suggests that either *L. ferrooxidans *or other member of the microbial community are producing acetate in the acidophilic biofilm under microaerophilic conditions.

**Conclusions:**

Our results indicate that the acidophilic filaments are dynamic structures in which different mechanisms for biofilm formation/dispersion are operating. Specific transcriptomic fingerprints can be inferred for both planktonic and sessile cells, having the former a more active TCA cycle, while the mixed acid fermentation process dominate in the latter. The excretion of acetate may play a relevant ecological role as a source of electron donor for heterotrophic Fe^3+ ^reducers like some Alphaproteobacteria, *Acidobacterium *spp. and *Sulfobacillus *spp., also present in the biofilm. Additionally, acetate may have a negative effect on bioleaching by inhibiting the growth of chemolithotrophic bacteria.

## Background

The prokaryotic diversity of the extremely acidic waters of the Río Tinto (southwestern Spain) has been studied for many years and is well characterized [1-4]. Among the most abundant microorganisms are the Gram-negative iron oxidizing bacteria *Leptospirillum ferrooxidans *and *Acidithiobacillus ferrooxidans*, both accounting for more than 70% of the prokaryotic population in the water column [2]. These bacteria have special relevance for the biomining industry because they are used to extract metals through the bioleaching of sulfide ores, and may produce acidification of mine drainages [5,6]. In addition, due to their very limited nutrient requirements and their association with iron and sulfur minerals, they are good models for the study of the origin, evolution and adaptation of life on Earth and elsewhere, particularly Mars [7-10]. The acidophile prokaryotic communities play a critical ecological role because they are responsible for maintaining the low pH and, as a consequence, other physicochemical conditions of the ecosystem, like the elevated heavy metal concentration. They are also critical for the existence of fully operative iron and sulfur cycles in the Río Tinto ecosystem [10].

We previously reported the environmental transcriptomic fingerprint of *L. ferrooxidans *in its natural planktonic microbial community under high iron and sulfur content (20 and 80 g L^-1 ^respectively), as well as high oxidative stress [11]. The cell density in the water column is relatively low (10^4^-10^6 ^cells mL^-1^), however, there are several types of macroscopic filaments (floating and submerged, streamers) in the river or attached to solid substrates forming true biofilms with similar prokaryotic diversity as that found in the water column [3].

Many bacterial species live predominantly in biofilms in both natural and artificial environments [12,13]. Biofilms can be defined as matrix-enclosed bacterial populations [12], dynamic structures in which transitions between the planktonic and biofilm modes of growth occur as a response to different environmental signals. Biofilms constitute habitats where microorganisms exhibit physiological heterogeneity and behavioral characteristics that makes them different of those with a free living lifestyle [14,15].

It has been reported that ore bioleaching requires the formation of biofilms as the mode for bacterium-mineral interaction [16]. Extracellular polymeric substances from biofilms seem to mediate in the attachment of cells to the solid substrates (metals, ores, etc.) and the electrochemical reactions operating in biocorrosion and bioleaching processes [17]. Among the factors affecting the biofilm formation and maintenance is the so called quorum sensing, by which microorganisms communicate via the secretion of chemical signaling molecules like acyl homoserine lactone, principally used by Gram-negative bacteria [18]. Up to two different acyl homoserine lactone production systems have been identified in *A. ferrooxidans *[19], however very little is known about quorum sensing in *Leptospirillum spp*. Metaproteomic [20] and metagenomic [21] studies in acidic biofilms dominated by *Leptospirillum ferriphilum *reported the presence of the *luxRI-*like genes, involved in quorum sensing through acyl homoserine lactone. Due to the important role of extremely acidophilic biofilms, both in microbial ecology and in industrial bioleaching, the aim of this work was to gain insights into the physiological differences between the planktonic (the free "swimming" cells) and sessile (in filamentous biofilms) modes of life through the analysis of the *Leptospirillum *spp. environmental transcriptomes.

## Results

### Physicochemical and microbiological characterization of sampling sites

**Figure 1:**
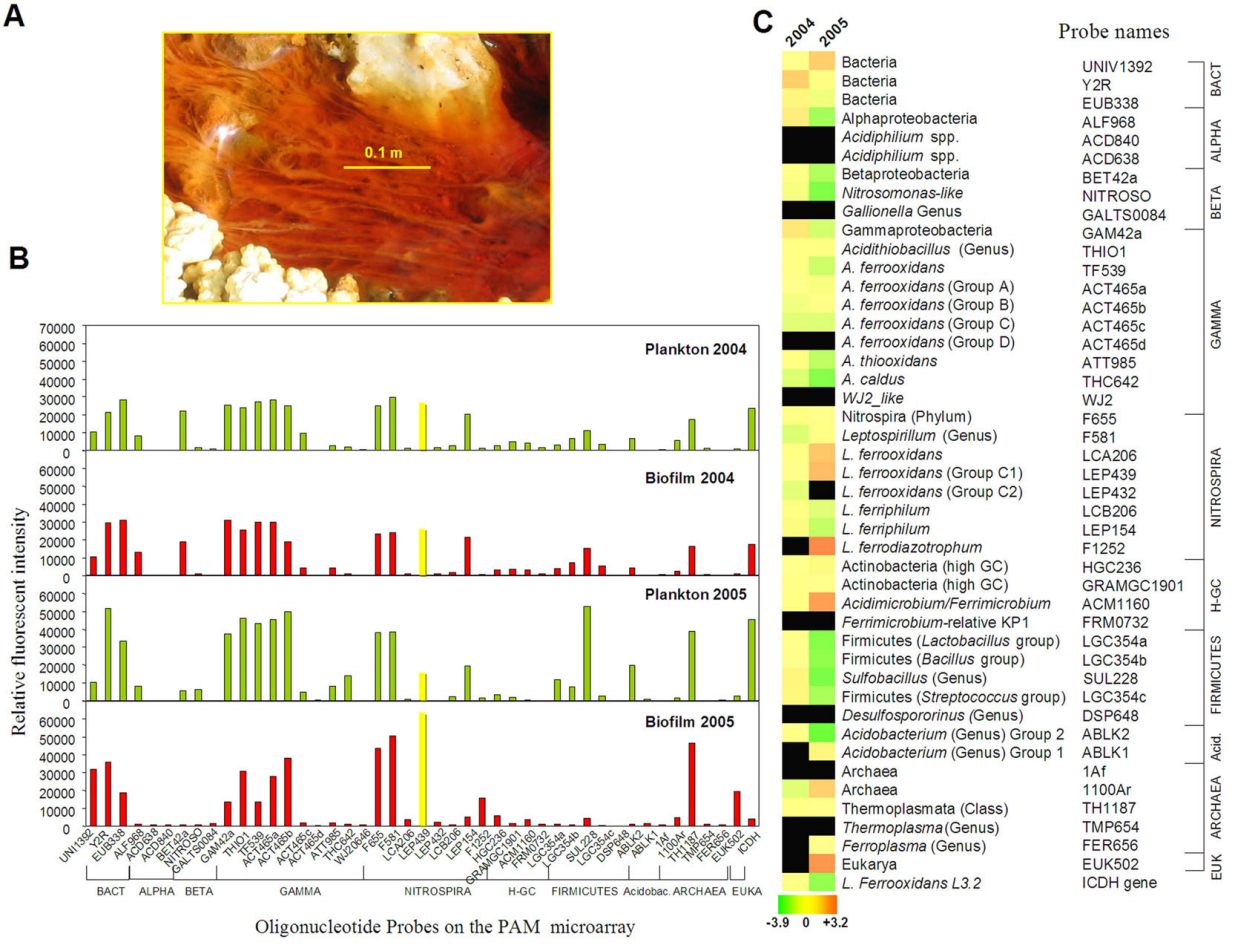
**Determining the microbial community composition of the sampling site**. (A) A picture of the field site in one of the Río Tinto springs showing details of the filamentous biofilms. (B) Estimation of the active microorganisms in the sampling site by using a prokaryotic acidophile microarray (PAM). Histograms show the signal intensities of each sample (biofilm and planktonic cells) after PAM micrarray hybridizations performed by simultaneous two-color labeled total RNA sample incubation (biofilm+planktonic) of each campaign. *yellow bars*, probe LEP439 for the *L. ferrooxidans *strain printed on the DNA microarray for transcriptomic studies. (C) Estimation of the relative proportion of rRNA in each sampling campaign by the analysis of Log_2 _ratio of biofilm/planktonic fluorescent signals (colored figure: *red*, dominate in biofilm; *green*, dominate in plankton; *yellow*, similar proportion in both places). The names of the microorganisms and the probes, as well as their phylogenetic adscription are indicated. H-GC, High GC content Actinobacteria.

Biomass was collected from water and filamentous biofilms (Fig. 1). The sampling site was mainly characterized by a high metal (10 to 20 g/L of Fe and 1.5 to 4.0 g/L of Al) and salt (>80 g/L of sulfate) content (Table 1). However, differences in some parameters could be observed between the two sampling times. In general, the 2005 samples contained higher concentrations of iron, sulfate, Al, Cu, Mg, Mn or Zn, while they had a lower amount of K^**+ **^and acetate (not detected) than the 2004 ones (Tables 1 and Additional file Table S1). Significant concentrations of acetate in water were also determined in other campaigns: 3.54 mM in June 2006, or 0.34 mM in July 2008.

**Table 1:** Physico-chemical parameters of the water in the Río Tinto sampling site.

	2004	2005
pH	1.8 ± 0.02	1.82 ± 0.07
T (°C)	21 ± 0.5	20.1 ± 0.5
Conductivity (mS Cm^-1^)	17.63 + 0.09	27.3 ± 0.01
Salinity	10.45 + 0.05	16.65 ± 0.05
O_2 _(% sat.)	-------	13.1 ± 3
SO_4_^= ^(mg L^-1^)	70200 ± 220	128000 ± 410
Fe tot (mg L^-1^)	13970 ± 230	19540 ± 2680
Fe^+2^/Fe^+3^	0.034	0.052
Formate, HCOO^- ^(mg L^-1^)	2.8 (0.06 mM)	ULD
Acetate, CH_3_COO^- ^(mg L^-1^)	82.68 (1.36 mM)	ULD

Other acetate measurements: 3.54 mM on June 2006, or 0.34 mM on July 2008.

ULD, under the limit of detection.

We estimated the microbial community composition by two color simultaneous hybridization of total RNA (from biofilm and water, the same used for gene expression studies, see below) to a prokaryotic acidophile microarray (PAM) already tested and validated with this type of samples [4,22]. The PAM analysis (Fig. 1BC) indicated the predominance of *A. ferrooxidans *(Gammaproteobacteria) and *Leptospirillum spp. *(*L. ferrooxidans *and *L. ferriphilum*, from Nitrospira phylum), both in water and in filaments along the two sampling times. Other groups of the microbial community with significant representation were the Firmicutes, some Alpha- and Beta-proteobacteria, *Acidimicrobium *spp., and some eukaryotes (only in the 2005 biofilm). In addition, the PAM analysis allowed us to estimate the relative proportion of the RNA of each strain between the plankton and biofilm (Fig. 1C), which was considered for normalization purposes (see methods). The composition of the free-living and the sessile prokaryotic communities was very similar in 2004, while it showed clear differences in 2005, when some groups like Firmicutes (specially *Sulfobacillus *spp.), Alpha and Betaproteobacteria, and *Acidomicrobium *predominated in plankton, while *Leptospirillum ferrodiazotrophum *was only detected in the biofilm.

We assume that other *Leptospirillum *spp. (*L. ferriphilum *and *ferrodiazotrophum*) present in the biofilm (Fig. 1) may be contributing to the microarray hybridization, as deduced from comparative genome hybridization (CGH) of *L. ferriphilum *using the *L. ferrooxidans *microarray (Additional file 1 Figure S1). By comparing the genome sequences of *L. ferrooxidans *to that of *L. rubarum *and *L. ferrodiazotrophum *(NCBI Project IDs 18795 and 37907 respectively ), we estimated a degree of sequence identity around 70-75%. This level of sequence identity has, in fact, allowed us to perform transcriptome studies of *L. ferriphilum *using the *L. ferrooxidans *microarray [23]. We hybridized at a temperature 5°C lower than that used for *L. ferrooxidans *transcriptome studies and the environmental transcriptome reported herein. Finally, the possible interference on the transcriptome studies by the other major component of the microbial community, the *A. ferrooxidans *group, was considered negligible following the CGH results (Additional file 1 Figure S1).

### Comparing the *Leptospirillum spp. *environmental biofilm and planktonic transcriptomes

Total amplified RNA from plankton and biofilm was fluorescently labeled (Cy3 for plankton and Cy5 for biofilm), mixed and set to hybridize with the genomic *L. ferrooxidans *DNA microarray. The signal intensities and the quality of the microarrays assayed with the samples collected in 2004 were higher than those of 2005, probably due to a better quality of the extracted RNA or to a higher metabolic activity in the 2004 samples (Additional file 1 Figure S2A). As expected, most of the probes on the microarray showed a Log_2 _ratio (biofilm/planktonic) between -1 and +1 in the experiments with samples from the two campaigns (the microarray data were deposited on Gene Expression Omnibus-GEO database with No. GSE20267 [25]). More than 50 probes bearing the *L. ferrooxidans *16S rRNA gene showed strong reproducible signals and Log_2 _ratios between -0.8 and + 0.8 from the 2004 and 2005 microarrays after normalization. These consistent results suggested the absence of RNA amplification bias in any of the samples, although we cannot exclude the possibility of some preference in the amplification of particular genes or regions. We selected 2524 different clone probes (out of 5348 of the whole microarray) having a significant intensity (more than a factor of 3 over the background signal) in at least one of the microarrays for further analysis. Then, up to 173 probes were selected having a biofilm vs plankton ratio higher than 2 in both campaign experiments, suggesting that the up-regulation of these genes was tightly related to the biofilm architecture and organization processes, rather than to differences in the environmental conditions (Additional file 1 Figure S2 B).

**Figure 2:**
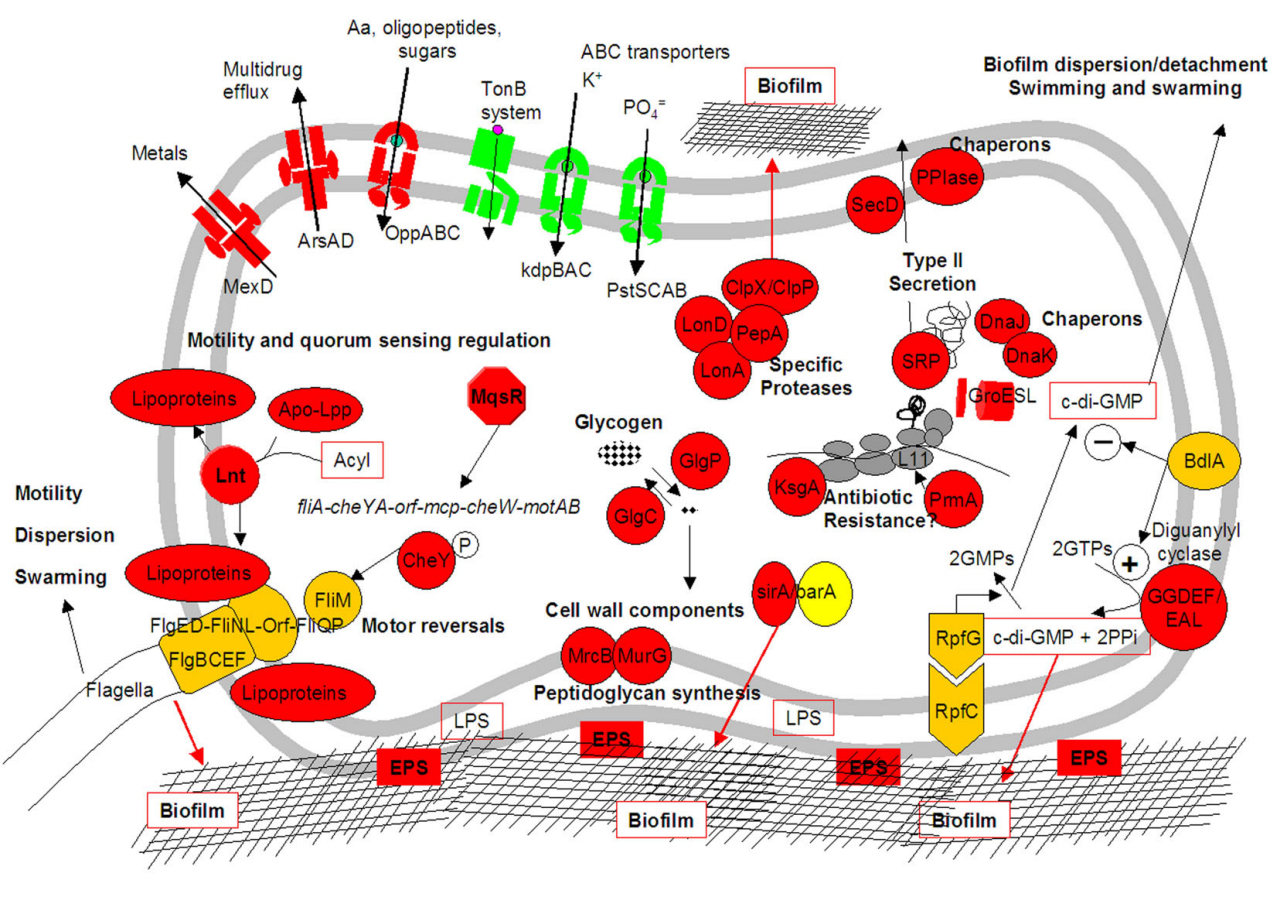
***Leptospirillum *spp. specific transcriptomic signatures in natural filamentous biofilm**. A schematic cell showing the most relevant proteins whose genes are preferentially expressed in biofilm (*red*) or in planktonic cells (*green*). *Yellow *and *orange *symbols indicate that the genes are well expressed in both places but with no clear preferential pattern. The extended names of the genes and the hypothetical proteins or functions are indicated in Additional file 1 Table S2 (see also text for explanation). The main functional categories affected are indicated: Biofilm formation and maintenance, quorum sensing, specific proteases, chaperons, and cell wall components. *EPS*, exopolysaccharydes; *LPS*, lipopolysaccharydes.

In previous works [11,24,26], those spots showing special up or down-regulation were selected and the corresponding clones were sequenced from both ends. We identified different open reading frames preferentially expressed in biofilm, others preferentially expressed in free living cells, and others with high expression levels in both sites (Additional file 1 Table S2, and summarized in Fig. 2). Many of them were assigned to already known genes and some corresponded to hypothetical conserved proteins of unknown function. As expected, the genes whose transcription was induced in biofilm corresponded preferentially to several functional categories related to biofilm formation and maintenance or key steps of central metabolism: (i) Cell envelope biogenesis and exopolysaccharide (EPS) synthesis; (ii) quorum-sensing and chemotaxis (*mqsR*); (iii) specific endopeptidase systems (*clpX*/P, *lon*); (iv) two-component sensor kinase systems (*sirA*-like, *rpfG*-like); (v) cofactor biosynthesis (heme, cobalamine, pantothenate); (vi) carbohydrate transport and metabolism; (vii) pentose phosphate pathway; (viii) deoxynucleotide biosynthesis; and (ix) mixed acid fermentation (*poxB, ackA, acsA*). On the other hand, the functional categories up-regulated in free living cells were related to: (i) ion transport, like PO_4_^= ^and K^+^; (ii) amino acids (His) and fatty acids biosynthesis; and (iii) Tricarboxylic acid (TCA) cycle enzymes (*pta*, *idh *encoding an ICDH-NAD^+ ^-dependent).

**Figure 3:**
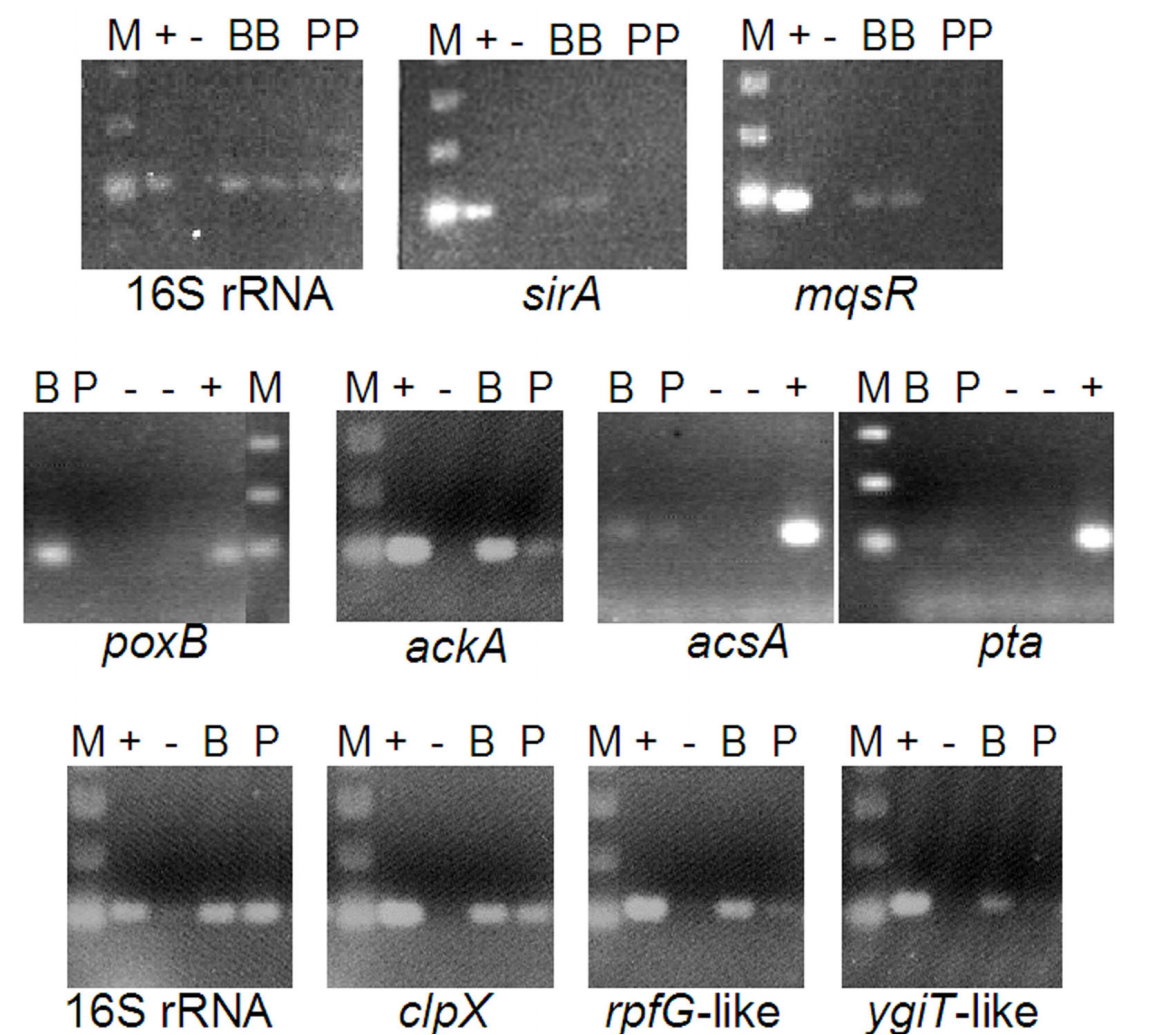
**Transcriptional up-regulation of a selected set of genes of *L. ferrooxidans *by RT-PCR analysis**. The transcriptional up-regulation of *sirA*-like, *mqsR*, *poxB*, *ackA*, *acsA*, *pta*, *clpX*, *rpfG*-like, and *ygiT(mqsA)*-like was confirmed by RT-PCR using sequences and primers from *L. ferrooxidans *(Additional file 1 Table S3) and total RNA from biofilm or planktonic cells. B, biofilm; P, planktonic cells; +, positive control with *L. ferrooxidans *genomic DNA; -, negative control (no template DNA); note that in the upper panel samples from biofilm and plankton are duplicated; M, relative molecular size ladder.

Some of the more relevant up-regulated genes were also checked by RT-PCR and qRT-PCR using total amplified RNA from biofilm and planktonic cells from the 2004 campaign. The preferential transcription detected by genome microarray experiments of *mqsR*, *sirA*-like, *poxB*, *acsA*, *pta*, *clpX*, *rpfG*-like, *ackA *and the unknown orf *ygiT*-like (downstream of *mqsR*), was confirmed by RT-PCR (Fig. 3). The qRT-PCR results further confirmed the induction of *mqsR *(4.1 fold), *sirA*-like (2.1 fold) and *poxB *(7.9 fold) in the biofilm, and *porA *(3.5 fold) and *pta *(2 fold) in planktonic cells.

### Effect of monosaccharides and small organic acids on the growth of *L. ferrooxidans*

**Figure 4:**
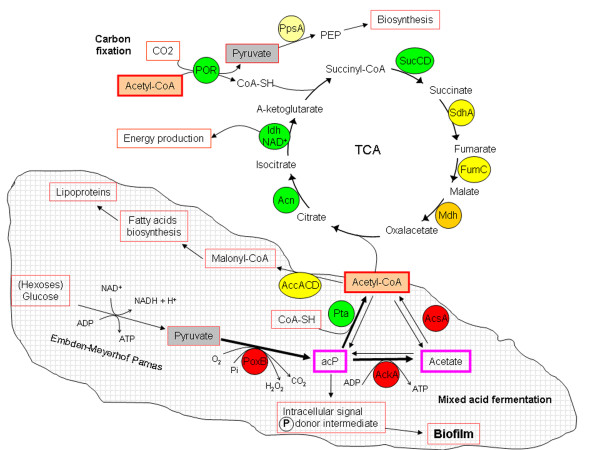
**Transcriptional evidence for metabolic differences between sessile (biofilm) and swimming (planktonic) modes of life**. Genes encoding mixed acid fermentation enzymes are up-regulated in the biofilm (*bottom part*). As a result, two metabolites can be produced: acetyl-phosphate (acP) and acetate. The former can be used as a phospho-donor intermediate, while the later can be transformed to acetyl-CoA or released to the medium. They could be produced by fermentation of sugars via the Embden-Meyerhof-Parnas pathway to pyruvate, then to acetyl phosphate (acP) by pyruvate oxidase (PoxB), and finally to acetate by AckA. In contrast, genes encoding key TCA cycle enzymes are up-regulated in the swimming (planktonic) cells (*top part; *see also text for explanations). Circles indicate the name of the enzymes whose genes are being expressed in the Río Tinto samples (*red*, preferentially induced in biofilm; *green*, in planktonic cells; and *yellow *transcribed in both places). All of them have been identified in the *L. ferrooxidans *genome sequence draft (to be published elsewhere; see Additional file 1 Table S2, and Additional file 2). POR, pyruvate oxidoreductase system; Pta, phosphotransacetylase; Idh, NAD+ dependent isocitrate dehydrogenase; Acn, Aconitase; Mdh, Malate dehydrogenase; FumC, fumarate hydratase component C; SdhA, succinate dehydrogenase component A; SucCD, Succinate dehydrogenase; PpsA, phosphoenolpyruvate synthase; AccACD, acetyl-CoA carboxylase.

**Figure 5:**
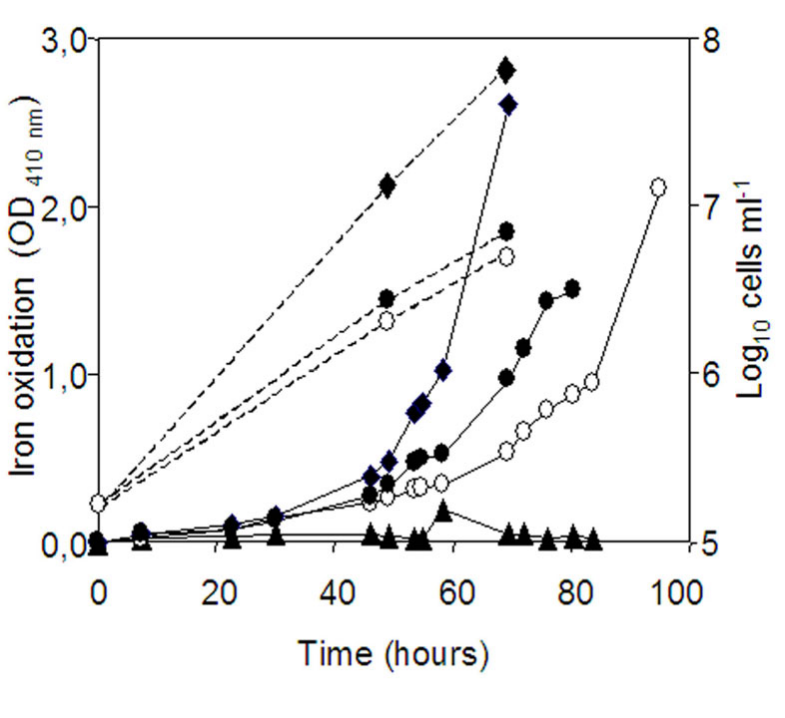
***L. ferrooxidans *cultivated in the presence of glucose, arabinose and acetate**. *L. ferrooxidans *was cultivated in Mckintosh medium with 2% (w/v) iron sulfate (*closed diamonds *♦), or 2% iron sulfate supplemented with 0.1% glucose (*open circles *○), 0.1% arabinose (*closed circles *●), or 1 mM acetate (*closed triangles *▲), and the growth monitored by measuring iron oxidation at OD_410 _nm. All the cultures were inoculated with 1.9 × 10^5 ^cells ml^-1^. After 48 h, the cell number clearly increased (*dashed lines*) with respect to the starting inoculums (1.9 × 10^5 ^cells ml^-1^).

The toxic effect of small organic acids (acetate, formate, etc) and other organic compounds on the growth of acidophile chemolithoautotrophs is well known [27]. The presence of significant amounts of acetate (up 3.54 mM on June 2006) in the acidic water of the Río Tinto spring suggested that *Leptospirillum *spp. were able either to grow or at least to tolerate this relatively high concentrations. Additionally, the induction of genes like *poxB, ackA*, or *acsA *also suggested that *L. ferrooxidans *and/or other *Leptospirillum *spp. are capable to produce acetate by mixed acid fermentation (Fig. 4). Acetate may be produced from the hexoses and pentoses in the biofilm matrix to render pyruvate via the Embden-Meyerhof-Parnas pathway and then to produce acetyl phosphate (acP) and acetate through reactions catalyzed by PoxB and AckA, as it was described in *Lactobacillus plantarum *[28]. To check whether the presence of hexoses and pentoses as well as organic acids were toxic, we cultivated our *L. ferrooxidans *strain under different concentrations of glucose, arabinose, pyruvate and acetate. The results showed a concentration-dependent growth on glucose and arabinose (0.1% w/vol allowed growth but not 0. 5% w/vol), as monitored by following the iron oxidation and cell counting (Fig. 5). No growth was detected in media containing pyruvate (0.25 mM) or acetate (1 mM).

## Discussion

### Transcriptomic fingerprints in extremely acidophilic biofilms

The analysis of the environmental transcriptomes of the *Leptospirillum *spp. in planktonic and biofilm natural communities resulted in the detection of several genes preferentially up-regulated in the biofilm. Most of them are related to functional categories and mechanisms known to be involved in biofilm formation and maintenance in other bacteria. In addition, genes involved in mixed acid fermentation were also induced in the biofilm. Some of these genes and their functional categories are discussed below and in *Additional file*.

#### Genes involved in biofilm production and maintenance

Several small size genes encoding hypothetical proteins were highly up-regulated in the streamers (Fig. 2 and Additional file 1 Table S2). A *sirA*-like gene that may be part of a two-component regulatory system was induced in the biofilm. In several species, SirA/BarA orthologues are required for virulence gene expression, exoenzyme and antibiotic production, motility, and biofilm formation [29]. A *bdlA*-like gene is highly expressed both in biofilm and plankton. This gene is involved in regulating chemotaxis and biofilm dispersion in *Pseudomonas aeruginosa *[30]. The authors proposed a model by which the BdlA protein acts as a sensor of environmental cues and controls both biofilm formation and biofilm dispersion/detachment by modulating the enzymatic activity of c-di-GMP-forming and hydrolyzing GGDEF/EAL diguanylate cyclase containing domains. Similarly, an *rpfG-rpfC *two-component system is being expressed in both lifestyles, with a slight preference for biofilm. The *rpfGC *operon is involved in the positive regulation of the synthesis of extracellular enzymes and polysaccharides [31], in the regulation of the formation and dispersal of biofilms, and is required for full virulence in *Xanthomonas campestris *[32]. Several proteins containing GGDEF or GGDEF/EAL domains are being expressed (Additional file 1 Table S2), some of them preferentially in the biofilm, and others in both lifestyles.

#### Genes for quorum sensing and motility

We identified a putative *mqsR *(*ygiU*)/*mqsA *(*ygiT*) -like operon [33,34] and both genes were highly up-regulated in the biofilm (Figs. 2, 3 and Additional file 1 Table S2). The MqsR-like protein shares 60% identity to the *E. coli *homologue, while MqsA (YgiT) has 45% identity to that of *E. coli *(Swissprot accession number Q46864.1). MqsR (*m*otility and *q*uorum *s*ensing *r*egulator) is directly associated with biofilm development and is linked to the development of persister cells [33,34]. In addition, and relevant for the streamer biofilms, deletion of *mqsR *leads to a reduction in biomass, substratum coverage, and changes the biofilm architecture when cultured in flow cell devices. MqsR transcriptionally regulates expression of genes necessary for motility, like *fliA *or *motA*. Very recently, Kim et al. [35] have reported that MqsR and MqsA (YgiT) are in fact a toxin/antitoxin (TA) pair which regulates additional loci besides its own. The system may be additionally regulated through the degradation of MqsA by ClpX/P and Lon proteases [35]. The genes encoding these proteases are also up-regulated in the Rio Tinto biofilm (see Additional file 1 Additional discussion). In our study, the *L. ferrooxidans *MqsR-like protein might be the responsible for more than two-fold upregulation of a chemotaxis and motility operon consisting of *fliA*-*cheY-cheA-orf-mcp-cheW-motA-motB*, where *fliA *is a sigma 28-like encoding gene (Fig. 2). A flagellar motor switch *fliM*-like gene, located downstream of a methyl-accepting chemotaxis gene *bdlA *(involved in biofilm dispersion) is also induced in biofilm streamers. On the contrary, a putative operon encoding a sigma-54 like factor followed by *flgB-flgC-fliE-fliFG-orf *is preferentially expressed in plankton, which may indicate different peculiarities in motility between both lifestyles: "swimming motility" in plankton and "swarming motility" in biofilm. A bioinformatic search of *mqsR*-like genes in public *Leptospirillum *spp. genome sequences (NCBI) was unsuccessful, so it seems to be only present in our *L. ferrooxidans *strain.

#### Genes for the synthesis of cell wall structures

Among the highest up-regulated genes was *lnt*, encoding an apolipoprotein N-acyltransferase. This enzyme is involved in lipoprotein biosynthesis by transferring the acyl group to the apolipoproteins. Lipoproteins are integral membrane proteins that bind to specific enzymes or transport proteins across the cell membrane. The Lnt protein is essential in *E. coli*, and its depletion causes mislocalization of outer membrane lipoproteins [36]. Lipoproteins are involved in functions like cell wall synthesis, secretion, or flagellar assembly and motility in *Salmonella *sp. [37]. Thermosensitive *lnt *mutants in *Salmonella *sp. are non-flagellate at 42°C. However, it has not been so far associated to biofilm formation or maintenance. Genes encoding key enzymes for the peptidoglycan synthesis are also up-regulated in biofilm, like *murB*, involved in the first committed step in the biosynthesis of the bacterial cell wall peptidoglycan [38]. Additionally, a *slt-*like gene, encoding a soluble lytic murein transglycosylase for recycling of muropeptides during cell elongation and/or cell division is also up-regulated. Several putative glycosyl transferases involved in EPS and lipoplysaccharide biosynthesis as well as cell wall biogenesis are being expressed both in biofilm and planktonic cells (not shown), some of them with slight preference for biofilm, like an O antigen polymerase and a putative lipoplysaccharide biosynthesis protein, and others with more than two fold induction in streamer biofilm (not shown).

#### Genes involved in mixed acid fermentation

Some genes involved in mixed acid fermentation showed up-regulation patterns as determined by microarray hybridization, RT-PCR and q-RT-PCR analysis (Figs. 2 and 3, Additional file 1 Table S2). This is the case for the genes *poxB*, *pta *and *ackA *encoding a pyruvate oxidase, phosphotransacetylase and acetate kinase enzymes, respectively. The *pta *gene is up-regulated in planktonic cells, while *poxB *and *ackA *are preferentially expressed in biofilm. These enzymes are critical for the control of the acetate, acetyl-phosphate (acP) and acetyl-CoA levels (Fig. 4). Wolfe *et al*. [39] showed evidence for an important role of acP as a global signal during the first steps of biofilm formation. The *pta *and *ackA *mutants showed aberrant biofilms and affected the expression of other genes involved in flagella, pili and fimbriae synthesis, stress proteins or colanic acid biosynthesis. Very recently, Gueriri *et al*. [40] reported that acP is an efficient phosphodonor for the response regulator DegU in *Listeria monocytogenes*, and that a double *pta-ackA *mutant unable to synthesize acP was strongly affected in chemotaxis, motility and biofilm formation.

The high induction of *poxB *in the biofilm (both by microarrays and q-RT-PCR), may be the responsible for the significant amounts of acetate measured in the water stream (Table 1). No *poxB *genes have been identified in other *Leptospirillum *genomes sequenced so far. The *L. ferrooxidans *PoxB protein (best BLAST e-values: 9e^-85 ^with *E. coli *PoxB, and 1e^-120 ^to that of *Oceanobacter *sp. RED65) is also very similar to other putative PoxB proteins from the Gammaproteobacteria and Firmicutes groups, like *Lactobacillus plantarum *(Additional file 1 Figure S5). The location of *poxB *and *mqsR *close to each other, next to transposase coding genes and at the end of a contig (Additional file 1 Figure S4), suggests that both genes may have been acquired by a horizontal gene transfer event from another microorganism.

**Figure 6:**
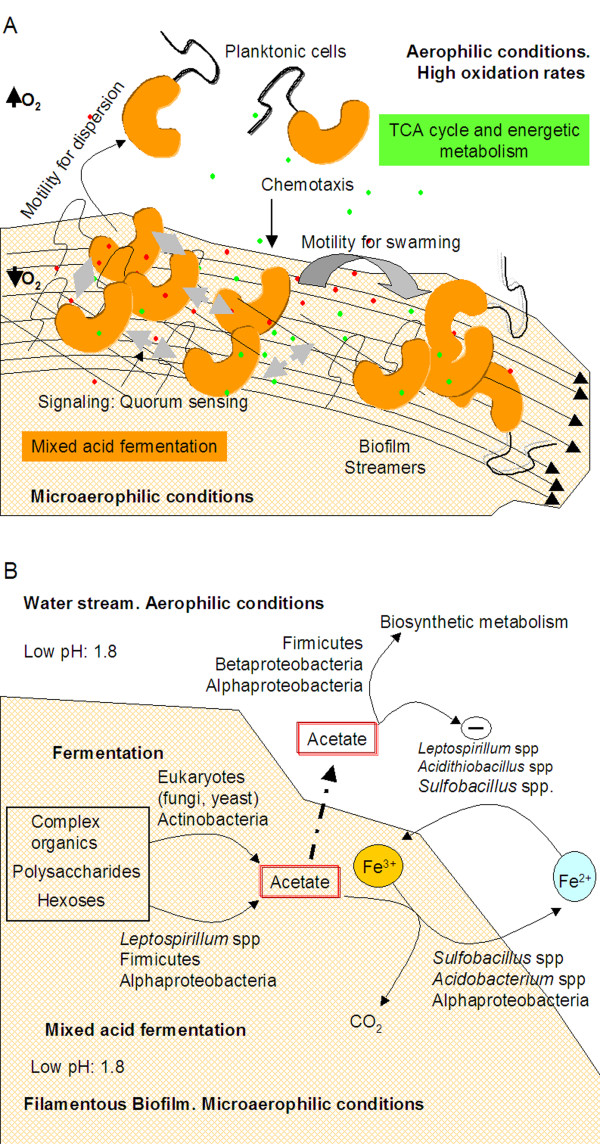
**Extremely acidophilic streamer filaments are dynamic biofilms and a source of small organic acids**. (A) Scheme showing the main functions related to biofilm formation, dispersion and maintenance that have been detected by environmental transcriptomic studies. In addition, two different metabolic states can be inferred: 1) under microaerophilic conditions in the biofilm, cells activate the fermentative pathways with a net production of acetate, and 2) free living cells under aerophilic conditions showed a high active TCA cycle for energetic metabolism. *Dashed arrows*, quorum sensing activities; *dots*, metabolites (acetate, formate, etc.). Long arrows indicate the water stream. (B) Role of acetate in the extremely acidophilic microbial community. The microorganisms identified by PAM (Fig. 1) are located as a function of their key metabolic role. Sugars from the biofilm (shadowed and hatched area) and other complex organic matter are decomposed by heterotrophs like Fungae and Actinobacteria. Secondary fermenters could render acetate that can be used by iron reducers to produce Fe^2+ ^which, in turn, is the energy source for iron oxidizers. The released acetate may inhibit the growth of chemolitotrophs like *Leptospirillum *spp. and *Acidithiobacillus *spp. (-), while it can be used by Firmicutes, *Sulfobacillus *spp., Alpha- and Betaproteobacteria as nutrient.

The oxygen concentration drops drastically in the water column, so most of the submerged streamers are microaerophilic. Under these conditions, the primary fermenters (Fungi, Actinobacteria, some Firmicutes) in the biofilm community can use the structural components of the biofilm (e.g. polysaccharides, hexoses, etc) to produce pyruvate via the Embden-Meyerhof-Parnas pathway. Then, the same or other bacteria (Firmicutes, Alphaproteobacteria, *L. ferrooxidans*) may oxidize and phosphorilate pyruvate by PoxB (pyruvate oxidase) to acetylphosphate (AcP) which, in turn, can be transformed to acetate by AckA (Fig. 4). A recent work reported the high hexose content in the extremely acidophilic biofilms from Río Tinto [41], which indicates that sugars from the same biofilm can be a primary source of fermentable material. It is known that acetic acid is toxic to chemolithoautotrophs like *A. ferrooxidans *[42] and *L. ferrooxidans *(see Results and below), however it can also be assimilated, like in *A. caldus *[43], or consumed by heterotrophic iron reducer microorganisms present in the community (e. g. *Acidobacterium *spp., some Alphaproteobacteria, *Sulfobacillus *spp.). A recent work by Nancucheo and Johnson [44] reported the presence of glycolic acid as an exudate in actively growing cultures of three chemolithotrophic acidophiles (*L. ferriphilum*, *A. ferrooxidans*, and *A. caldus*). The glycolic acid showed similar toxicity as acetic acid with 21 strains of acidophiles screened (among them *L. ferriphilum*), while only members of the Firmicutes group (essentially *Sulfobacillus *spp.) were capable to metabolize it. Similarly, the acetic acid we detected in the river's water can be consumed by *Sulfobacillus *spp. or other Firmicutes (Fig. 6). We did not detect glycolic acid nor acetic acid production in the culture media of *L. ferrooxidans *even when it was cultivated in the presence of 0.1% glucose or arabinose. This result, together with the environmental transcriptomic studies shown herein suggest that only sessile *L. ferrooxidans *cells in the biofilm are capable to produce acetate.

### The transcriptomic fingerprint of *L. ferrooxidans *planktonic cells

The highest induced gene (more than 20 fold) in planktonic cells was that encoding a NAD^+^-dependent isocitrate dehydrogenase that we reported previously [11]. The upregulation of this gene and other TCA genes could be explained by increased expression of genes involved in carbon fixation in the planktonic phase using the pyruvate oxido-reductase pathway (POR) and the reductive TCA cycle, as we suggested previously [11], and also proposed for another *Leptospirillum *species [45] (see above and Fig. 4). The next most up-regulated genes in planktonic cells corresponded to ABC transport systems for potassium (*kdpBAC*) and phosphate (*pstSCAB*). The induction of *kdpBAC *may be a consequence of the high salt concentration (more than 70 g/L of SO_4_^=^) in the water, which could be compensated by the accumulation of intracellular K^+ ^that would play a role as osmoregulator, as in the halophilic bacteria *Halomonas elongata *[46]. This would imply that biofilm structures are playing a protective role against salinity that renders unnecessary the intracellular accumulation of K^+ ^as osmoprotectant. Alternatively, a lower concentration of intracellular K^+ ^in biofilm streamers may favor cellular aggregation, as it has been reported very recently in *B. subtilis*, where some natural products that caused potassium leakage triggered multicellularity [47]. In addition, Ueda and Wood [48] reported that potassium and sodium transporters regulate cellular adhesion and virulence to barley in *P. aeruginosa*. In this scenario, the role of K^+ ^as osmoprotectant may be replaced by the compatible solute trehalose in the sessile cells, in which the trehalose synthase gene (*treS*) is up-regulated more than 6 fold (Additional file Table S2).

The *pstSCAB *operon, encoding the specific phosphate ABC transporter, is also preferentially expressed in planktonic cells. In *E. coli*, transcription of the *pst *operon is induced under phosphate starvation and the full-length mRNA is rapidly processed post-transcriptionally [49]. The high amounts of Fe^3+ ^and Al^3+ ^ions in the Río Tinto samples may favor phosphate precipitation [50], so that this nutrient is limited for planktonic cells. *In vitro *experiments with *P. aeruginosa *demonstrated that acute phosphate depletion resulted in increased expression of PstS and biofilm production [51]. Moreover, depletion of intestinal phosphate after operative injury activates the virulence of *P. aeruginosa*, causing lethal gut-derived sepsis. The Río Tinto planktonic cells may be in similar situation: the continuous phosphate deficiency and high levels of PstS shifts towards a biofilm producing phenotype and, once the biofilm is well established, the *pstSCAB *operon is repressed.

## Conclusions

Our results indicate that the main mechanisms governing biofilm formation and dispersion are operating in natural extremely acidophilic filamentous biofilms. The identified genes corresponded to others known to be involved in different stages of biofilm formation in other bacteria [52]. Therefore, we conclude that the acidophilic biofilm streamers are dynamic structures in which mechanisms like quorum sensing, motility and chemotaxis, swarming and biofilm dispersion/formation are all operating (Fig. 6). Specific transcriptomic fingerprints inferred for both planktonic and sessile cells showed metabolic differences, having the former a more active TCA cycle, while the mixed acid fermentation process dominated in the latter.

The up-regulation of the mixed acid fermentation genes suggests that the acetate detected in the extremely acidic water of the sampling site (up to 3.54 mM in June 2006) may be a consequence of acetic acid production by the acidophilic biofilms (Fig. 4). The excreted acetate could play an ecological role by controlling the growth of chemolithoautotrophs and as a source of electrons for heterotrophic Fe^3+ ^reducers like some Alphaproteobacteria, *Acidobacterium *spp. and *Sulfobacillus *spp., all detected with the PAM microarray (Fig. 1). The newly produced Fe^2+ ^could be used as energy source for iron oxidizers like *L. ferrooxidans *and *A. ferrooxidans*, closing the iron cycle (Fig. 6B).

## Methods

### Sample collection

Samples used in this study were collected from a permanent spring running under a pile of pyrite-containing rocks accumulated by mining activities. Sampling was performed in October 2004 and 2005, after dry summers and before any autumn rainfall in the area. Biomass from 4 liters of water was recovered by filtration through nitrocellulose membranes (0.22 μm of pore diameter, Millipore Co.) and filters were immediately placed in 5 ml of RNAlater solution (Ambion) according to the manufacturer's protocol. Up to 2 g of filament samples growing in the same sampling site were collected in 10 ml of RNAlater solution. All samples were frozen on dry ice and kept at -20°C until use.

### Determination of physicochemical parameters in the sampling sites

The pH, conductivity, salinity, dissolved oxygen and redox potential were measured in situ with a Multi197i multiprobe device (WTW GmbH, Weilheim, Germany). The elemental composition and concentration (Table 1) was determined by inductively coupled plasma spectrometry (ICP), with an Optima 3300 DV instrument (Perkin Elmer) by the Centro de Espectrometría Atómica, Departamento de Análisis Químico Elemental (UCM, Madrid). Total iron, Fe^3+ ^and Fe^2+ ^were determined by colorimetric methods [53]. Sulfate (SO_4_^=^) was determined by atomic absorption spectroscopy with a Perkin-Elmer 3030 instrument by the Centro de Espectrometría Atómica, Departamento de Análisis Químico Elemental (UCM, Madrid). Small organic acids (acetate, formate) were determined by ion chromatography with a Metrohm 861 Advanced Compact Ion Chromatographer IC (Metrohm AG, Herisau, Switzerland). Appropriate controls were run to discriminate between acetate and glycolate anions (not shown).

### Strain and culture conditions

*Leptospirillum ferrooxidans *RT32a, a natural isolate from Río Tinto was cultivated in Mckintosh medium [54] in the presence of different concentrations of glucose, arabinose, pyruvate or acetate. The growth was monitored by iron oxidation and cell counting by using a Newbauer chamber under an optical microscope as previously reported [26].

### Environmental RNA extraction and amplification

Samples preserved in RNAlater (Ambion) were centrifuged at 10.000 × g for 10 min and washed in acid water (0.1 M sulfuric acid) for subsequent RNA isolation. Total environmental RNA was extracted and amplified through a method based on T7 RNA polymerase linear amplification as described previously [24,11]

### Estimation of biodiversity by using a prokaryotic acidophile microarray

The prokaryotic diversity was determined by a prokaryotic acidophile microarray (PAM) as reported previously [4] ], and also used to monitor the prokaryotic diversity in industrial bioleaching [22]. The PAM was developed to monitor the prokaryotic diversity in extremely acidophilic environments with oligonucleotide probes targeting most known acidophilic microorganisms, including members of the Alpha, Beta, and Gammaproteobacteria, the Nitrospira phylum, acidobacteria, sulfur reducing bacteria, Actinobacteria, the low G+C Firmicutes group, and Archaea from the Ferroplasma and Thermoplasma genera. The biodiversity was analyzed using fluorescently-labeled total environmental RNA from the same samples used for transcriptomic analysis. PAM microarrays hybridizations were carried out at 50°C from 6 to 12 hours as described [4].

### Genomic microarrays, environmental RNA labeling and hybridization

A shotgun genomic DNA library from an environmental isolate of *Leptospirillum ferrooxidans *(Strain RT32a, renamed from the initially L3.2) was printed on a microarray for transcriptomic analysis [26]. Because the microarray had 2-3 fold genome coverage, most genes were redundantly represented by several overlapping probes (see also Figs. S5 to S7). Up to 2.5 μg of amplified total environmental RNA (metatranscriptome) from planktonic cells and from biofilms collected in two different campaigns (October 2004 and October 2005) were labeled by cDNA synthesis and hybridized with the *L. ferrooxidans *microarray as described [11]. Since our interest was to identify genes differentially expressed in planktonic or biofilm cells, we considered both campaigns as experimental replicates. In addition, technical replicates were carried out with the 2005 samples.

### Scanning and Data Analysis

Hybridized slides were scanned for Cy3 and Cy5 dyes in a GenePix 4100A Scanner and the images were analyzed with Genepix pro 6.0 software (Axon Instruments). The microarray hybridization results were analyzed using AlmaZen system v.2.1 software (Bioalma, Madrid, Spain) and normalized by applying the locally weighted scatter plot smoothing (LOWESS) algorithm [55]. Additionally, data were also normalized by taking into account the relative proportion of the strain used for whole transcriptomic analysis (*L. ferrooxidans *RT32a, probe LEP439 in the PAM microarray) between the biofilm and plankton. This proportion was nearly the same in plankton and biofilm in 2004, while it was twice in the 2005 biofilm when compared to the water stream (Fig. 1B, C). The criteria for selecting induced spots were: a signal intensity more than 3 times the background in at least one of the channels (Cy5 or Cy3), and an a minimal induction ratio of 2 in one of the two campaigns. In some well justified cases, such as genes that are part of well known pathways, induction ratio values between 1.5 to 2 were also considered. FASTA software was used to search for similarities against the non-redundant protein and DNA sequence databases of the National Center for Biotechnology (NCBI).

### RT-PCR and real time quantitative PCR (qRT-PCR) Analysis

The relative abundances of a set of up-regulated genes were determined in biofilm and planktonic cells by RT-PCR and real-time PCR. Specific primers for the genes of interest were designed (Additional file 1 Table S3) for amplifying products of 80 to 110 bp, having GC content and Tm of about 50% and 55°C, respectively. RNA samples were treated with Turbo DNase (Ambion) at 37°C for 30 min before applying RT-PCR protocols. Equal amounts of environmental DNase I-treated RNA samples were used to synthesize cDNA with Superscript II Reverse Transcriptase (Invitrogen Life Technologies) at 42°C for 50 min. The reactions were treated for 15 min at 70°C to inactivate the enzyme. To remove RNA complementary to the cDNA, 2 units of RNase H (Invitrogen Life Technologies) were added and incubated at 37°C for 20 min before RT-PCR and Q-RT-PCR experiments were performed. RT-PCR for several genes was performed at 54°C (annealing temperature) and the amplicon products analyzed on agarose gels stained with ethidium bromide. The qRT-PCR quantifications were performed on the cDNA obtained using 25 μl volumes in 96 well PCR plate format. The reactions were carried out with iQ™ SYBR^® ^Green Supermix according to the manufacturer's instructions with 0.4 μM primer concentration. Termocycling was conducted using a MyiQ™ Single-Colour Real-Time PCR Detection System (BioRad Laboratories). Cycling parameters were initially 5 min at 95°C followed by 40 cycles of 94°C 30 s, 60°C 30 s and 72°C 30 s. Each run was completed with a melting curve analysis to confirm the specificity of amplification and lack of primer dimers. Amplification plot and predicted threshold cycle (Ct) values were obtained with the IQ5 optical system v. 2.0. Software (BioRad Laboratories). The experiments were performed using triplicate dilution series for each gene and cDNA preparations (biofilm and planktonic cells) by the comparative threshold cycle method. PCR efficiencies were calculated using the standard curve method [56]. Fold-change and SD values were calculated by efficiency-corrected ΔCt method using the 16S rRNA gene as reference.

### DNA Sequencing and Analysis

Sequencing reactions were performed from plasmid minipreps extracted by the automated pipetting system epMotion 5075 VAC (Eppendorf). Sequencing was made using dye terminator cycle sequencing reactions and run in an ABI Prism 3730xl sequencer (Applied Biosystems). Sequences were analyzed and assembled using SeqMan (DNASTAR package software, LASERGENE Madison, WI) and Phred-Phrap-Consed [57]. Annotations were generated by an automatic pipeline that used Glimmer 3.03 [58] to predict genes and a combination of BLAST searches against the NCBI and Swissprot non redundant protein databases to identify putative functions. In addition, sequences were also analyzed by using RPSBLAST (NCBI) to assign the predicted gene products to protein families according to the schemes of the COG, PFAM, SMART and PRK databases. We have now sequenced the whole microarray clone library and we have generated an annotated genome sequence draft from *L. ferrooxidans *(to be published elsewhere). By mapping the clones onto the genome draft we can easily identify gene annotations associated with each probe on the microarray (Additional file 1 Figure S3).

## Authors' contributions

MMP carried out the sampling, RNA, microarray work and analysis, and RT-PCR and qRT-PCR experiments; MJG carried out the sequence annotation and participated in microarray analysis; AA contributed in sequence analysis and in silico metabolic studies; and VP participated in sampling, microarray analysis and wrote the paper. All authors read and approved the final manuscript.

## Supplementary Material

Additional file 1 **Additional discussion, tables and figures Additional discussion concerning: the upregulation of genes involved in other functions like sugar metabolism, pentose-phosphate pathway, and oligopeptide ABC transporters**. Additional Tables from S1 to S3 Additional figures from S1 to S5 Additional references. Click here for file

Additional file 2 **DNA sequences of the genes from Additional file **1 **Table S2**. Click here for file
