# Supplementary material for: Environmental transcriptome analysis reveals physiological differences between biofilm and planktonic modes of life of the iron oxidizing bacteria Leptospirillum spp. in their natural microbial community
Source: BMC Genomics. 2010 Jun 24;11:404. doi: 10.1186/1471-2164-11-404 (PMC2996932; doi:10.1186/1471-2164-11-404)
Supplement: Additional file 1 — Additional discussion, tables and figures Additional discussion concerning: the upregulation of genes involved in other functions like sugar metabolism, pentose-phosphate pathway, and oligopeptide ABC transporters. Additional Tables from S1 to S3 Additional figures from S1 to S5 Additional references. [file 1471-2164-11-404-S1.DOC]

# Additional file 1

# Environmental transcriptome analysis reveals physiological differences between biofilm and planktonic modes of life of the iron oxidizing bacteria *Leptospirillum spp.* in their natural microbial community.

**Mercedes Moreno-Paz, Manuel J. Gómez, Aida Arcas, and Víctor Parro^*^**

Department of Molecular Evolution, Centro de Astrobiología (INTA-CSIC), Carretera de Ajalvir km 4, Torrejón de Ardoz, 28850 Corresponding autor:

Victor Parro

Centro de Astrobiología (INTA-CSIC), Carretera de Ajalvir km 4, Torrejón de Ardoz, 28850 Madrid, Spain.

Phone: +34-915201071

Fax: +34-915201074

e-mail: [parrogv@inta.es](mailto:parrogv@inta.es)

# Additional Discussion

## Other functional categories up-regulated in environmental extremely acidic biofilms

In addition to the genes described in the main text, we identified up-regulated genes involved in other functional categories which have been reported in the literature to be related to biofilm formation and maintenance. These functional categories are:

*Genes for specific proteases.* Two gene clusters, one encoding a trigger factor and the ClpX-ClpP components of the Clp protease, and the other comprising *clpC-gcp* genes (encoding for ClpC protease and a glycoprotease, respectively), are also over-expressed in the biofilm streamers. The trigger factor is involved in protein export and acts as a chaperon by maintaining the newly synthesized protein in an open conformation. It has been reported that the trigger factor is involved in stress tolerance, competence development and biofilm formation in the dental pathogen *Streptococcus mutans* [1]. ClpX is the ATP-dependent specificity component of the ClpXP protease complex. It directs the protease to specific substrates and can perform chaperon functions in the absence of ClpP. ClpP cleaves peptides with a chymotrypsin-like activity in a process that requires ATP hydrolysis and plays a major role in the degradation of misfolded proteins. In general, the Clp ATPases are related to environmental stress tolerance and, particularly, the ClpXP protease complex is involved in tolerance to osmotic and oxidative stresses [2]. Both ClpC and ClpX are required for biofilm formation in *Staphylococcus aureus*, although ClpP-deficient cells are more prone to biofilm formation than wild type cells [3]. Very recently, de Bruijn and Raaijmakers provided evidence that serine protease ClpP regulates the biosynthesis of massetolides, cyclic lipopeptides involved in swarming motility, biofilm formation and antimicrobial activity of *P. fluorescens* SS101 [4].

Genes encoding putative LonA and LonD specific proteases were also up-regulated in biofilm. Marr *et al*. reported that Lon protease of *P. aeruginosa* is involved in swimming, swarming and twitching motility [5] . Kim *et al*. reported that ClpX, ClpP, and Lon proteases are involved in the mode of action of the MqsR/MqsA toxin anti-toxin system, probably by degrading MqsA [6].

*Genes encoding chaperons and stress related genes***.** Several genes encoding chaperons and stress related proteins are up-regulated in the biofilm streamers (Additional file 1, Table S2 ). Among the most induced are a gene encoding for a putative heat shock protein that contains a highly conserved DnaJ domain at the C-terminal end, and a putative *grpE-dnaK-dnaJ* operon, which has been reported to be involved in the biosynthesis regulation of the *P. putida* biosurfactant Putisolvin [7]. Putisolvin is a non-ribosomally synthesized cyclic lipopeptide which, among several functions, facilitates the swarming motility as well as inhibits biofilm formation [8]. A *clpB* gene is also up-regulated 5 to 7 fold in the acidophilic biofilm streamers, and it may convey protective functions against environmental stress [3]. A cluster of three putative peptidyl-prolyl cis-trans isomerases (PPIase) are up-regulated in the biofilm (Additional file 1, Table S2). Two of them could be PrsA-like foldases and the other one could be a PPIase with chaperone activity SurA. PPIases are required to assist extracellular and outer membrane protein folding [9]. Due to the high number of genes related to membrane proteins and secretion that are being up-regulated in the biofilm, a requirement for this type of folding-assisting proteins is not surprising. Consistently, genes from the general secretion pathway, like the main component of the signal recognition particle (*ffh*) or *secD*, were also up-regulated in biofilm, probably to secrete proteins involved in cell wall biogenesis.

*Sugar metabolism*. The expression of *treS* gene, for trehalose synthase, is induced in the biofilm. Trehalose is an important and widespread compatible solute which can function as a storage carbohydrate and protects against several stresses. The *glgC* and *glgP*, for glycogen biosynthesis and catabolism, are also up-regulated. It has been reported that glycogen biosynthesis and its subsequent turnover are both required for optimal biofilm formation [10]. Ectopic expression of either the glycogen biosynthetic genes (*glgC, glgA*) or the catabolic one (*glgP*) in a *csrA* wild-type strain significantly enhanced biofilm formation. Glycogen could serve as carbon and/or energy source for the formation of one or more adhesins or other factors necessary for biofilm formation. Additionally, genes involved in the pentose phosphate pathway, like a *pgl*-like gene, were induced in biofilm. The *pgl* gene encodes a 6-phosphogluconolactonase, the enzyme responsible for the hydrolysis of 6-phosphogluconolactone to 6-phosphogluconate, which is the key, second step, in the pentose phosphate pathway. In *E. coli,* the pentose phosphate pathway is used to create ribose molecules for biosynthesis, to increase reducing power in the cell, and to metabolize some sugars.

### *Oligopeptide ABC transporters.* A putative *appABC* operon encoding an oligopeptide transport system is highly expressed both in biofilm and plankton. However, a different *oppA-*like gene encoding a periplasmic oligopeptide transport system component is preferentially induced in biofilm and, with lower intensity, the *oppCB* components located in a separate region. The Opp system is important for the uptake of oligopeptides and for supplying a nitrogen source by recycling the cell wall peptides for the synthesis of new peptidoglycan [11]. This may be the role in the *L. ferrooxidans* streamer cells, because we detected induction of some genes related to peptidoglycan synthesis and degradation (Additional file 1, Table S2). Different functions of the Opp system have been reported in bacteria. For example, roles in stimulation of competence and sporulation in *Bacillus subtilis* [12], induction of intracellular aggregation in *Enterococcus faecalis* [13], signaling process of peptide taxis in *E. coli* and *Salmonella* [14], or effect on the adherence of *Streptococcus pneumoniae* to human lung cells [15]. Lee *et al*. reported a *Vibrio fluvialis* *oppA* mutant whose biofilm productivity was twice than that of the wild type [16] .

# Additional File 1 Tables and Figures

## Table S1 - Metal concentration and pH of the water at the Río Tinto sampling site.

|  | 2004 | 2005 |
| --- | --- | --- |
|  |  |  |
| pH | 1.8+ 0.02 | 1.82 ± 0.07 |
| Fe^+2^ (mg L^-1^) | 461 ± 76 | 973 ± 180 |
| Fe^+3^ (mg L^-1^) | 13507 ± 223 | 18567 ± 2653 |
| Al (μg ml^-1^) | 1798 | 3762 |
| As (μg ml^-1^) | 4.41 | 5.36 |
| Cd (μg ml^-1^) | 0.12 | 0.15 |
| Co (μg ml^-1^) | 9.2 | 14.8 |
| Cr (μg ml^-1^) | 0.15 | 0.21 |
| Cu (μg ml^-1^) | 6.9 | 16.7 |
| K (μg ml^-1^) | 0.41 | 0.21 |
| Mg (μg ml^-1^) | 900 | 1816 |
| Mn (μg ml^-1^) | 9.7 | 29.1 |
| Ni (μg ml^-1^) | 0.41 | 1.11 |
| Pb (μg ml^-1^) | 2.5 | 3.48 |
| Zn (μg ml^-1^) | 7.6 | 14.3 |

## Table S2 - Up-regulated genes in biofilm and planktonic cells. Genes are listed according to different functional categories. The best averaged ratio biofilm/planktonic cells in the two sampling campaigns is indicated on the right side.

Up-regulated genes in biofilm cells

|  | **Ratio*(Biofilm/Plankton)** | | |
| --- | --- | --- | --- |
| **Gene** | **Description** | **2004** | **2005** |
| **Cell wall components and structures** | | | |
| ***lnt*** | Apolipoprotein N-acyltransferase | 11.5 | 7.5 |
| ***murB*** | UDP-N-acetylmuramate dehydrogenase | 2.2 | 2.8 |
| ***murA*** | UDP-N-acetylglucosamine 1-carboxyvinyltransferase | 1.5 | 1.6 |
| ***slt*** | Soluble lytic murein transglycosylase | 2.1 | 4.0 |
| ***ugtP*** | Diacylglycerol glucosyltransferase (membrane glycolipids formation) | 3.0 | 1.5 |
| ***mrcB*** | Peptidoglycan glycosyltransferase. Murein Polymerase | 2.0 | 2.0 |
| ***shc*** | Squalene hopane cyclase (triterpenes synthesis) | 1.5 | 3.5 |
| ***uppS*** | Di-trans, poly-cis-decaprenylcistransferase | 1.5 | 2.1 |
|  |  |  |  |
| **Quorum sensing, chemotaxis and motility** | | | |
| ***mqsR*** | Motility and quorum sensing regulator | 8.1 | 4.1 |
| ***fliA*** | Motility and flagellar sigma factor sigma 28 | 1.8 | 1.6 |
| ***cheY*** | Chemotaxis protein CheY | 3.0 | 1.6 |
| ***cheA*** | Chemotaxis protein CheA | 2.8 | 1.7 |
| ***mcp*** | Methyl accepting chemotaxis protein | 2.8 | 1.6 |
| ***cheW*** | Chemotaxis protein CheW | 2.0 | 1.2 |
| ***motAB*** | Motility proteins A and B | 1.7 | 1.4 |
| ***fliM*** | Flagellar motor switch protein FliM | 1.7 | 2.1 |
| ***fliLN-fliO-fliP*** | Flagellar basal body, switch, and biosynthesis proteins | 6.5 | 2.5 |
|  |  |  |  |
| **Two-component regulatory systems** | | | |
| ***sirA*-like** | Response regulator with a BarA-like signal transduction His kinase | 11.3 | 9.0 |
| ***rpfG/rpfC*** | Response regulator RpfG and signal transduction His kinase | 1.8 | 2.0 |
| ***kdpD*** | Osmosensitive potassium His kinase KdpD | 4.0 | 5.4 |
| ***pilS/pilR-like*** | Putative PilS/PilR two-component system. Upstream of *flgBCfliEFG* | 1.8 | 1.0 |
|  |  |  |  |
| **GGDEF/EAL sensor proteins** | | | |
| ***orf_2505*** | GGDEF domain protein. Putative Diguanylate cyclase | 2.5 | 1.9 |
| ***orf_2506*** | GGDEF/EAL domains protein. Diguanylate cyclase/Phosphodiesterase | 3.0 | 1.8 |
| ***orf_3403*** | Putative diguanylate cyclase/phosphodiesterase with GAF sensor | 2.0 | 2.0 |
| ***orf_3404*** | Putative diguanylate cyclase/phosphodiesterase with PAS/PAC sensor | 2.0 | 2.0 |
| ***orf_1094*** | Putative diguanylate cyclase/phosphodiesterase with PAS/PAC sensor | 2.0 | 1.8 |
| ***orf_1930*** | Diguanylate cyclase | 2.0 | 2.0 |
|  |  |  |  |
| **Specific proteases** | | | |
| ***clpX/clpP*** | ATP-dependent Clp protease proteolytic subunit | 8.4 | 15.9 |
| ***lonD*** | ATP-dependent protease La 2 | 3.2 | 6.2 |
| ***lon*** | LonH1-like protease homolog 1, mitochondrial precursor | 5.6 | 1.7 |
| ***pepA*** | Probable cytosol aminopeptidase Leucine aminopeptidase | 2.0 | 3.1 |
|  |  |  |  |
| **ABC transporters** | | | |
| ***oppA*** | Oligopeptide transport system | 5.1 | 10.6 |
| ***helA*** | Transporter for heme to the periplasm for cytochrome c assembly | 4.1 | 5.9 |
| ***modB*** | Molybdenum transport system permease | 2.4 | 1.0 |
|  |  |  |  |
| **General secretion system** | | | |
| ***secD*** | Protein-export membrane protein SecD | 3.0 | 4.4 |
| ***ffh*** | Signal recognition particle protein (SRP) protein Ffh | 2.8 | 2.0 |
|  |  |  |  |
| **Other Membrane Proteins** | | | |
| ***ompA*** | Outer membrane protein OmpA family | 3.5 | 3.9 |
| ***yfgL*** | Lipoprotein YfgL. Outer membrane assembly processes. | 2.2 | 2.5 |
| ***orf_2983*** | Outer membrane efflux protein | 4.0 | 3.3 |
| ***orf_2498*** | Lipoprotein Surface Ag. Probably involved in machinary assembly | 2.5 | 3.6 |
| ***orf_2016*** | Permease of the major faclitator superfamily | 4.5 | 8.3 |
|  |  |  |  |
| **Chaperons** | | | |
| ***orf_3233*** | Heat shock DnaJ containing domain protein | 10.0 | 10.0 |
| ***clpB*** | ClpB chaperone | 7.0 | 5.0 |
| ***clpC-gcp*** | ATP proteases with chaperon activities | 5.0 | 13.0 |
| ***grpE-dnaKJ*** | GrpE, DnaK and DnaJ chaperons | 3.0 | 4.0 |
| ***groESL*** | 10 kDa chaperonin (Protein Cpn10) and GroEL chaperon | 2.6 | 2.6 |
| ***surA*** | Chaperon SurA. Prolyl isomerase/chaperon for OMP assembly | 2.2 | 3.0 |
| ***prsA*** | Foldase protein PrsA-like | 2.1 | 6.0 |
| ***hsp*** | Small heat shock protein Hsp20-like | 3.0 | 3.0 |
| ***dipZ*** | Thiol disulfide oxidoreductase. Oxidative protein folding pathway | 1.5 | 4.0 |
|  |  |  |  |
| **Antibiotics and multidrugs resistance. Efflux pumps** | | | |
| ***mrcB*** | Penicillin-binding protein 1A. Penicillin-insensitive transglycosylase | 3.6 | 4.3 |
| ***mexB*** | Multidrug resistance protein MexB | 3.4 | 0.1 |
| ***fusC*** | Fusaric acid resistance protein | 3.3 | 2.3 |
|  |  |  |  |
| **Metal resistance** | | | |
| ***arsA*** | Arsenite-activated ATPase ArsA | 3.7 | 7.3 |
| ***arsD*** | Arsenical resistance operon represor | 3.6 | 7.3 |
|  |  |  |  |
| **Sugars metabolism** | | | |
| ***pgl*** | 6-phosphogluconolactonase (pentose phosphate pathway) | 7.7 | 7.5 |
| ***treS*** | Trehalose synthase (Maltose alpha-D-glucosyltransferase) | 7.0 | 11.0 |
| ***jgt*** | Amylomaltase 4-alpha-glucanotransferase | 4.2 | 11.0 |
| ***araJ*** | Arabinose efflux permease | 3.9 | 5.7 |
| ***glgA2*** | Glycogen shynthase | 2.1 | 1.7 |
| ***glgC*** | Glucose-1-phosphate adenylyltransferase | 1.1 | 3.3 |
| ***glgP*** | Glycogen phosphorylase | 2.2 | 2.0 |
| ***rfbC*** | dTDP-L-rhamnose synthase | 2.6 | 2.1 |
| ***rfbF*** | Glucose-1-phosphate cytidylyltransferase | 2.2 | 1.8 |
|  |  |  |  |
| **Central metabolism. Mixed acid fermentation** | | | |
| ***poxB*** | Pyruvate oxidase-dehydrogenase (Ubiquinone) | 3.1 | 4.0 |
| ***ackA*** | Acetate kinase. Acetyl-CoA biosynthesis | 4.0 | 3.3 |
| ***acsA*** | Acetyl-CoA synthetase | 3.1 | 1.8 |
| ***mdh*** | Malate dehydrogenase | 2.3 | 2.9 |
| ***namA*** | NADPH dehydrogenase (xenobiotic reductase) | 2.2 | 2.1 |
|  |  |  |  |
| **Aminoacids metabolism** | | | |
| ***lysA*** | Diaminopimelate decarboxylase | 2.5 | 0.1 |
| ***trpE*** | Anthranilate synthase | 2.0 | 2.0 |
|  |  |  |  |
| **Energetic and respiratory metabolism** | | | |
| ***cydA*** | Cytochrome d ubiquinol oxidase subunit 1. Respiratory chain | 2.0 | 0.3 |
| ***hycDEG*** | Hydrogenase. Formate hydrogenlyase | 1.5 | 3.4 |
|  |  |  |  |
| **Cofactor biosynthesis: tetrapyrrole, cobalamine, thiamine** | | | |
| ***hemL*** | Glutamate-1-semialdehyde 2,1-aminomutase (GSA-AT) | 6.9 | 5.5 |
| ***moaA*** | Molybdenum cofactor biosynthesis protein A | 4.0 | 5.5 |
| ***cobT*** | Nicotinate-nucleotide-dimethylbenzimidazole phosphoribosyltransferase | 3.5 | 0.8 |
| ***nadA*** | Cofactor biosynthesis; NAD(+) biosynthesis | 3.3 | 3.3 |
| ***thiG/thiD/thiE*** | Thiamine biosynthesis | 2.6 | 3.6 |
| ***csd*** | Cysteine desulfurase | 4.0 | 1.0 |
|  |  |  |  |
| **Nucleic acids metabolism** | | | |
| ***pyrR*** | PyrR bifunctional protein | 2.9 | 2.1 |
| ***pyrF*** | Orotidine 5'-phosphate decarboxylase (OMP decarboxylase) | 3.4 | 7.9 |
| ***lig*** | DNA ligase | 3.4 | 8.1 |
| ***recJ*** | Single-stranded-DNA-specific exonuclease RecJ | 2.7 | 4.4 |
| ***ksgA*** | Dimethyladenosine transferase | 2.3 | 3.1 |
| ***prmA*** | Ribosomal protein L11 methyltransferase (L11 Mtase) | 3.1 | 3.7 |
| ***mod*** | Type III restriction-modification system StyLTI enzyme Mod | 2.1 | 3.9 |
| ***FHIT*** | Diadenosine tetraphosphate hydrolase | 3.9 | 9.4 |
|  |  |  |  |
| **Transposition elements** | | | |
| ***ydcM*** | Transposase for insertion sequence element IS201 | 3.4 | 2.1 |
| ***orf_3972*** | Hypothetical 45.4 kDa protein in *snaA-snaB* intergenic region | 3.6 | 5.0 |
| ***yncI*** | Putative transposase yncI family 11 | 2.4 | 2.0 |
| ***orf_3153*** | Transposase | 3.3 | 4.0 |
|  |  |  |  |
| **Proteins of unknown function** | | | |
| ***dfrA*** | Putative dihydroflavonol-4-reductase | 2.0 | 2.3 |
| ***orf_2555-6-7*** | Small ORFs for hypothetical proteins | 4.0 | 1.6 |
| ***orf_3039*** | Putative oxidoreductase | 3.2 | 4.5 |
| ***orf_1736*** | HD superfamily phosphohydrolase | 7.0 | 4.0 |
|  |  |  |  |

Up-regulated genes in planktonic cells

| **Ratio* (Biofilm/Plankton)** | | | |
| --- | --- | --- | --- |
| **Gene** | **Description** | **2004** | **2005** |
| **Central metabolism** | |  |  |
| ***pta*** | Phosphotransacetylase | 2.0 | 2.1 |
| ***kdpBAC*** | High affinity ATP-driven potassium transport | 3.0 | 2.6 |
| ***pstSCAB*** | Phosphate transport ABC system | 5.0 | 3.5 |
| ***tonB*** | TonB-like biopolymer transport system | 4.0 | 5.0 |
| ***idh*** | NAD(+)-dependent IDH (isocitrate dehydrogenase) | 1.9 | 20.0 |
| ***porABCforD2*** | Pyruvate ferredoxin oxidoreductase (POR) | 2.0 | 2.3 |
| ***fusA2-tuf1*** | Elongation factors G and F | 3.0 | 2.6 |
| ***rplK-rplA*** | Ribosomal proteins L11 and L1 | 2.0 | 3.7 |
| ***acnA*** | Aconitase | 2.0 | 2.5 |
| ***fdhA*** | Putative formate dehydrogenase Fe only (anaerobic) | 3.0 | 1.4 |
| ***sucCD*** | Succinate dehydrogenase | 2.0 | 2.1 |
|  |  |  |  |

*Ratio correspond to the best probes on the microarray fulfilling the selection criteria: signal intensity > 3 times background and a Ratio (in general) equal or higher than 2 in at least one of the sampling times (see also Methods and Results).

## Table S3 - ***Oligonucleotide pairs used for RT-PCR and qRT-PCR studies***.

| **Primer** | **Sequence (5’ – 3’)** | **nt** | **Tm (ºC)** | **Position** |
| --- | --- | --- | --- | --- |
|  |  |  |  |  |
| 16S_F | CTACGGGAGGCAGCAGTGAGG | 21 | 58.5 | 305..325 |
| 16S_R | TTCGTCCCGGGCAAAAGTGGT | 21 | 62.1 | 395..415 |
| acK_2_F | GACCGGTCCGATCTGCTTGAA | 21 | 58.6 | 97..117 |
| acK_2_R | ACCTCCATGAACGACCCTGACA | 22 | 57.9 | 195..174 |
| msqR_F | GATGGGACTTGGGTTTTCTGAC | 22 | 54 | 102..123 |
| msqR_R | ACCTTGTGATCGGCGTTCGT | 20 | 56.7 | 17..198 |
| rpfG_F | TGACTGTCGTAGGCGTTGATAA | 22 | 52.6 | 41..62 |
| rpfG_R | GACCTTTCTGATGCCGTTCTT | 21 | 52.6 | 156..136 |
| ygiT_F | GCGAGGCCATTCTTGACC | 18 | 52.7 | 122..139 |
| ygiT_R | TCTTTCTGACTTTGGCGATGTA | 22 | 51.9 | 238..217 |
| clpXP_F | AGGTTTTGCGATCTATGACACA | 22 | 51.5 | 21..237 |
| clpXP_R | GCTCCTGCTGCAAGAAGAAAT | 21 | 53 | 300..320 |
| SirA_F | TATTGCCCTGGTCCGCTTATG | 21 | 56.4 | 5..72 |
| sirA_R | TATCCTTGGCTGAACCCTCGTC | 22 | 56.9 | 133-154 |
| pta_F | CTCGAAGCCATGAAGGAAAACT | 22 | 54.3 | 121..142 |
| pta_R | TCAATGATCTCGACGGGAACA | 21 | 52.5 | 210..230 |
| map_F | TAATCGCCGTAACCCAAGAGT | 21 | 53.1 | 44..64 |
| map_R | AATTGCGCACTACAGAAAAACC | 22 | 53 | 151..172 |
| poxB_F | TCCCAGGCGGTGCGACAG | 18 | 60.1 | 424..441 |
| poxB_R | CCGTGGTGGAGCAGGAAGC | 19 | 57.7 | 513..495 |
| acsA_F | AATGGAACCCGCAAAACTATGAA | 23 | 56.5 | 194..216 |
| acsA_R | ATGGCGATGAGAGCGACTTTGT | 22 | 57.5 | 314..293 |
| alaS_F | CTTTACCCGCAGGCATCATAC | 21 | 53.8 | 240..260 |
| alaS_R | CAAGCTCCCGGGTCAGAAAT | 20 | 55.5 | 352..333 |
| porA_F | CATGAGAGGCGAGTCCGAGTTT | 22 | 57.2 | 252..273 |
| porA_R | TGGTCCCGCAGTTGTTGTGA | 20 | 57 | 342..323 |


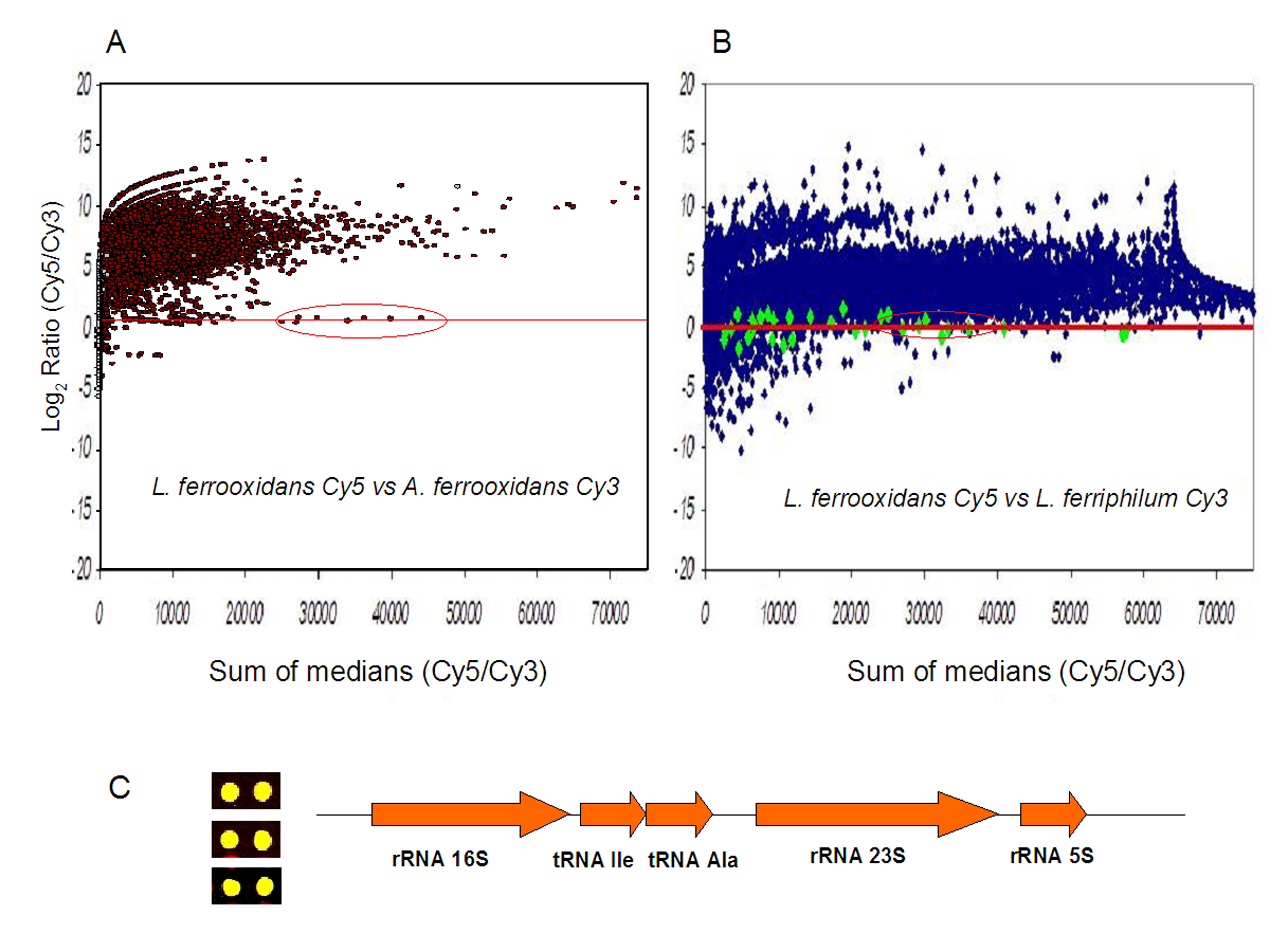


# Fig. S1. Checking the cross-hybridization between *L. ferrooxidans* and other bacteria present in the biofilm by Comparative Genome Hybridization (CGH). (A) CGH scatter plot obtained with total DNA from *L. ferrooxidans* and *Acidithiobacillus ferrooxidans* using the *L. ferrooxidans* DNA microarray. The red circle highlights some selected spots showing a Log_2_ Ratio (*L.fe*/*A.fe*) equal or near 0 (*yellow* spots in C). These spots corresponded to DNA fragments encoding totally or partially the rRNA operon (C, *filled arrows*). Ratios are massively displaced to *L. ferrooxidans*, indicating that there is very little sequence similarity between both genomes. (B) CGH scatter plot obtained with total DNA from *L. ferrooxidans* and *L. ferriphilum*. The ratios are also displaced to *L. ferrooxidans* but most of them fit between Log_2_ Ratio 0 and 5, indicating that there is still significant hibridization.


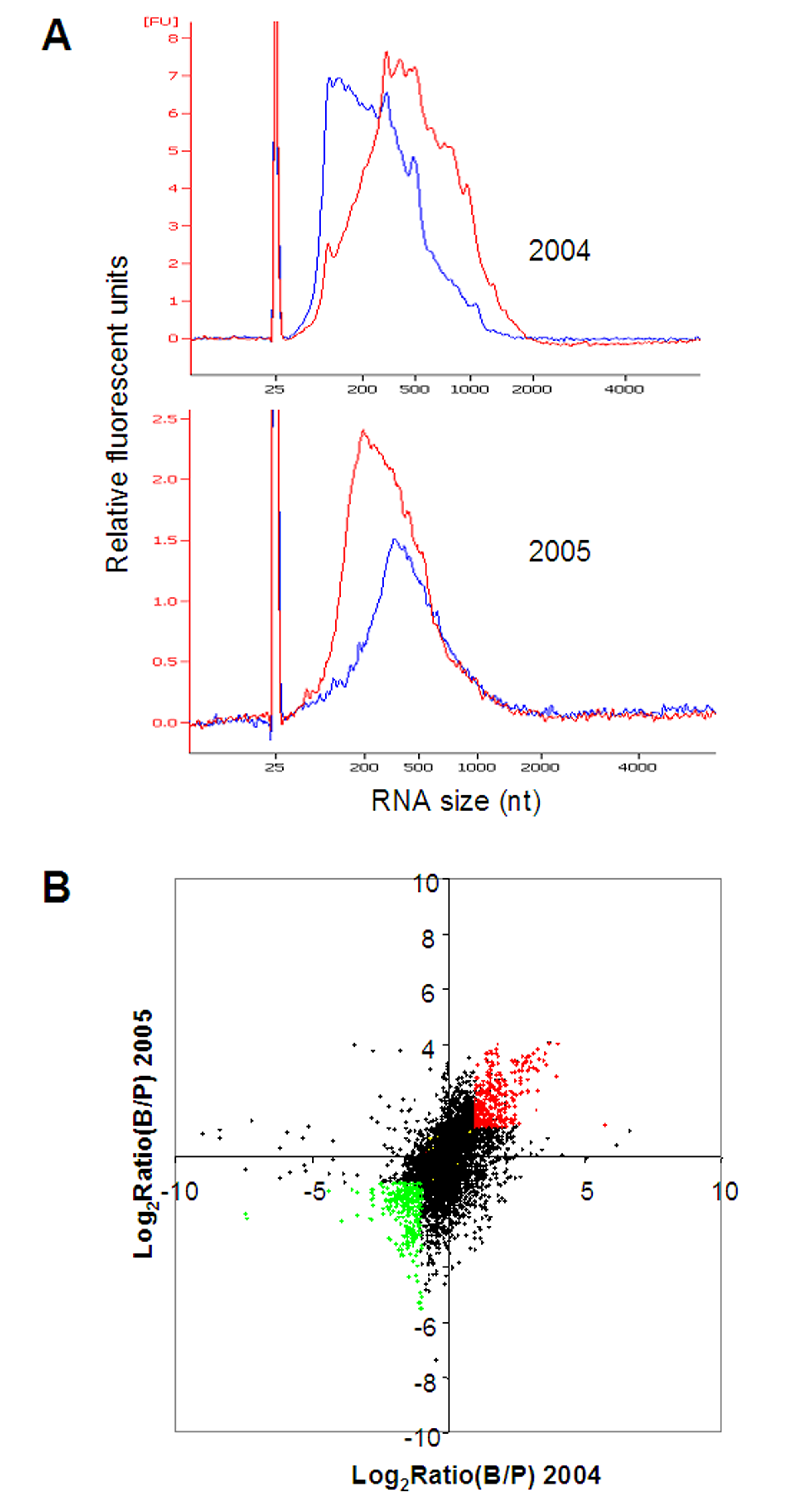


# Fig. S2. Environmental RNA amplification and *L. ferrooxidans* genomic microarray analysis. (A) Total environmental RNA from the sampling site (*red lines*, biofilm; *blue*, planktonic cells) at the two different campaigns was amplified and analyzed in an Agilent Bioanalizer 2100 (Experimental Procedures). *red*, biofilm RNA; *blue*, planktonic RNA. (B) Selection of those spots showing more than two fold induction in biofilm (*red*) or in planktonic cells (*green*) in both campaigns.


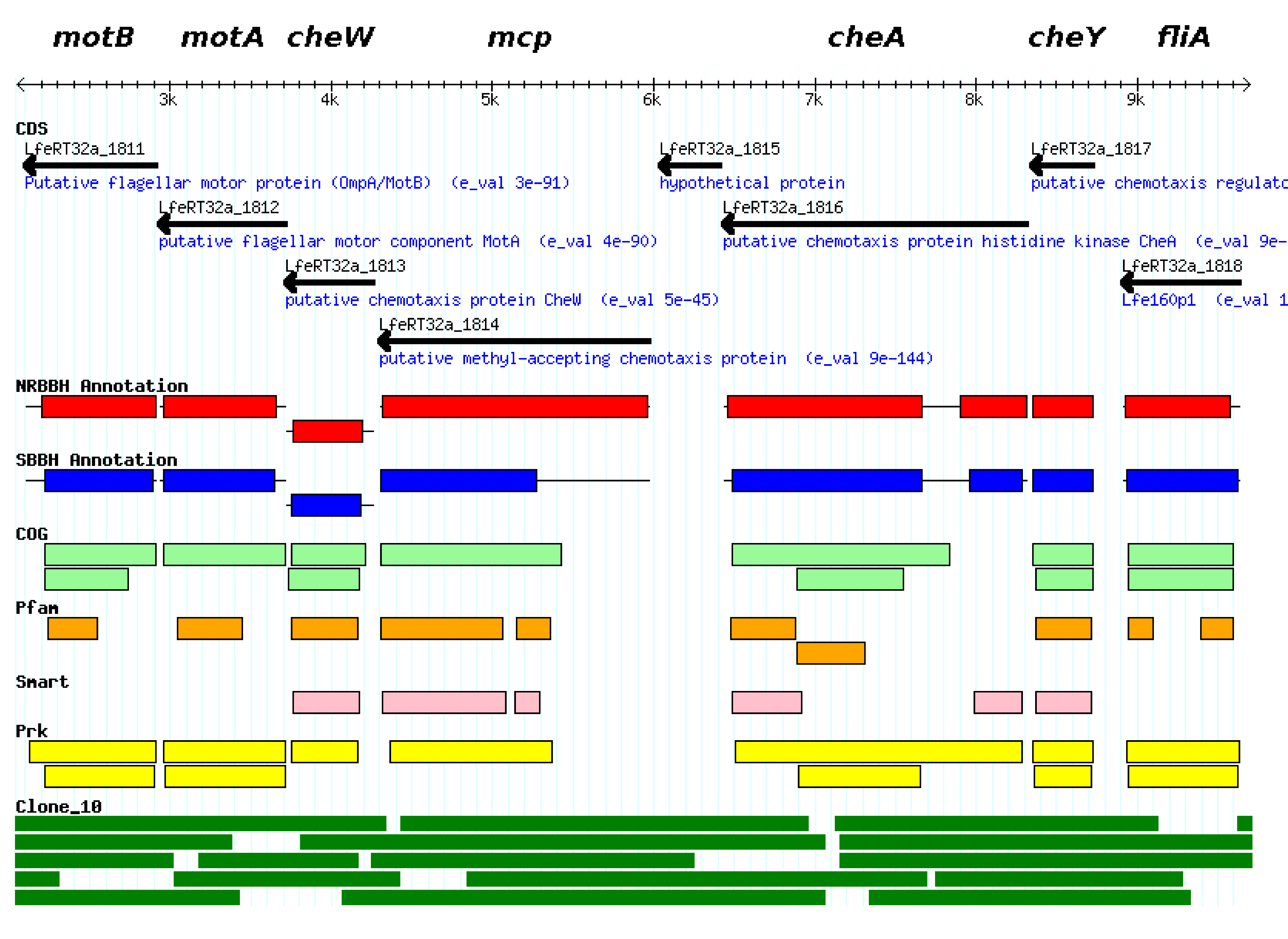


# Fig. S3. Visualization of the *L. ferrooxidans* genome sequence draft annotations with GBrowse. A region encompassing *fliA* and other genes involved in chemotaxis and motility is shown as example. The first track (*CDS*) shows the position of the predicted genes, labeled with their corresponding systematic identifier and a summary of their main annotation. The following two tracks represent the similarity between the predicted gene products to entries at the non redundant NCBI protein (*NRBBH*) and Swissprot (*SBBH*) databases, detected with BLAST. The following four tracks represent the affiliation of the predicted gene products to protein families within the *COG*, *Pfam*, *SMART* and *Prk* databases, established by RPSBLAST. The last track (*Clone_10*) depicts the position of clones used as templates for sequencing reactions and as a source of target DNA fragments in the expression microarray. Because the microarray had 2-3 fold genome coverage, a redundancy in the probe coverage is highly frequent, so that several probes overlap in the same region. Only some of the clones overlapping the region are shown.

#

**
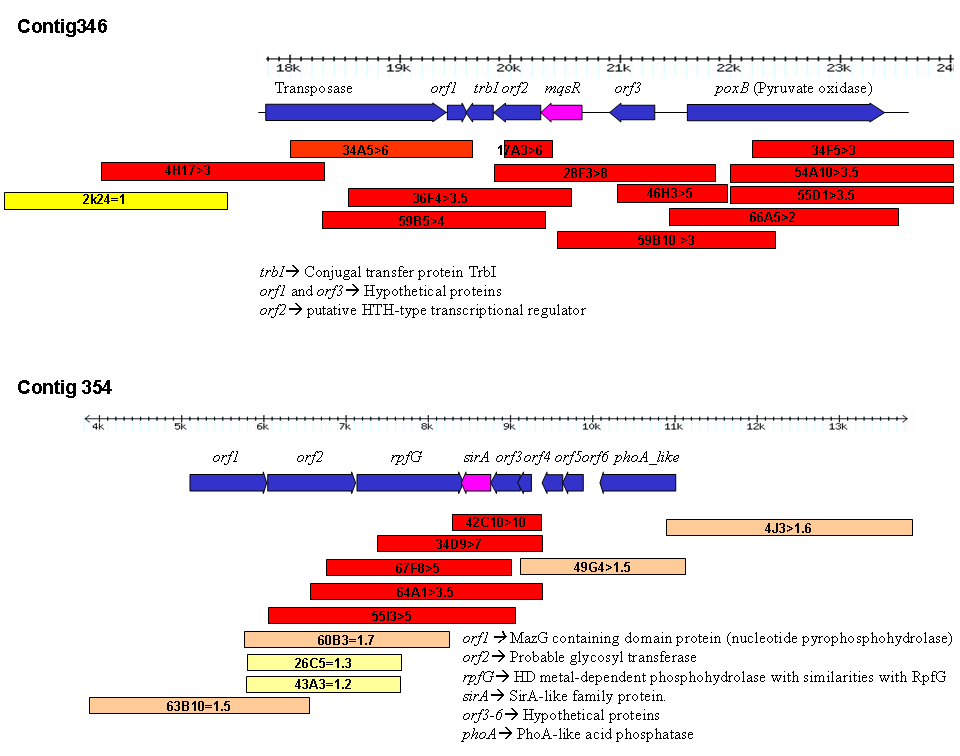
**

# Figs. S4. Partial regions from the sequence contigs of the *L. ferrooxidans* genome sequence draft showing two of the most up-regulated regions in the extremely acidic biofilms: (1) one comprising the motility and quorum sensing regulator (*mqsR*) and pyruvate oxidase (*poxB*; Contig 346), and (2) the *sirA*-like gene containing region (Contig 354). *Scaled lines* indicate the DNA fragment size; *blue arrows*, the open reading frames; *filled bars*, the different DNA fragments spotted on the microarray covering these zones (*orange* and *red*, upregulated in biofim; *yellow*, no preferential induction). The names of each DNA fragment and the induction ratio are indicated inside each rectangle (e.g. 28F3>8 means fragment 28F3, ratio >8). The *mqsR* and *sirA* ORFs are highlighted in purple.


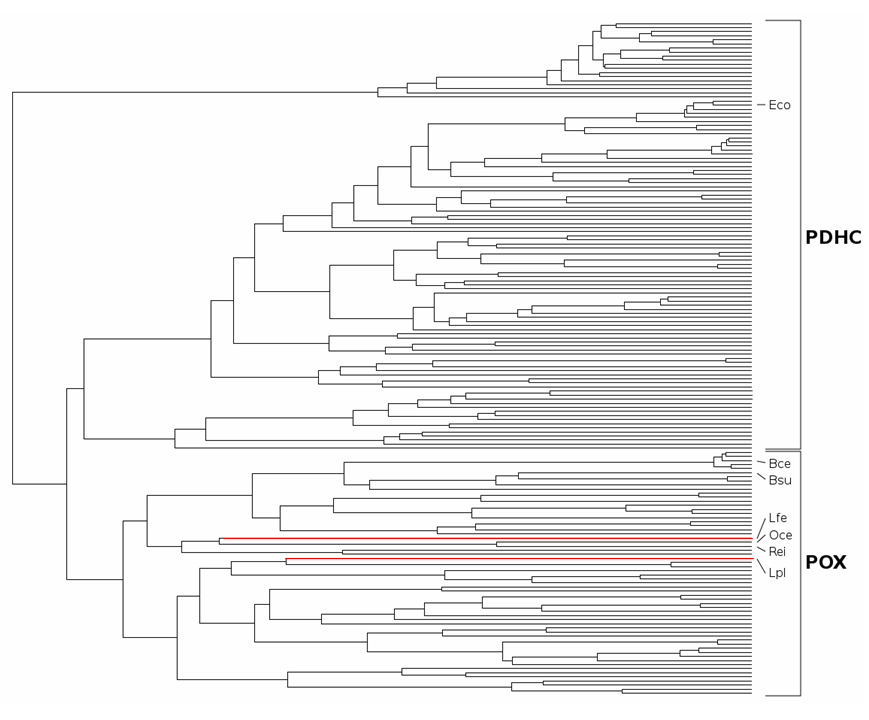


# Fig. S5. Phylogenetic tree of PoxB proteins. PoxB corresponds to pyruvate oxidase (EC 1.2.3.3) while PDHC is a pyruvate dehydrogenase cytochrome (EC 1.2.2.2). The phylogenetic analysis indicates that *L. ferrooxidans* PoxB (Lfe) is a true pyruvate oxidase, mostly similar to those described in the Gammaproteobacteria *Oceanobacter* spp. (Oce) and *Reinekea* spp. (Rei). It is also related to the PoxB of Firmicutes, like *Lactobacillus plantarum* (Lpl), and more distantly to that of *Bacillus cereus* (Bce) and *B.* *subtilis* (Bsu).

### Additional references

1. Wen ZT, Suntharaligham P, Cvitkovitch DG, Burne RA: **Trigger factor in *Streptococcus mutans* is involved in stress tolerance, competence development, and biofilm formation.** *Infect Immun* 2005, **73**:219-25.
2. Robertson GT, Ng WL, Foley J, Gilmour R, Winkler ME: **Global transcriptional analysis of *clpP* mutations of type 2 *Streptococcus pneumoniae* and their effects on physiology and virulence**. *J Bacteriol* 2002, **184**:3508-20.
3. Frees D, Chastanet A, Qazi S, Sørensen K, Hill P, Msadek T, Ingmer H: **Clp ATPases are required for stress tolerance, intracellular replication and biofilm formation in *Staphylococcus aureus***. *Mol Microbiol* 2004, **54**:1445-62.
4. de Bruijn I, Raaijmakers JM**:** **Regulation of cyclic lipopeptide biosynthesis in *Pseudomonas fluorescens* by the ClpP protease**. *J Bacteriol* 2008, **191**:1910-23.
5. Marr AK, Overhage J, Bains M, Hancock RE**:** **The Lon protease of *Pseudomonas aeruginosa* is induced by aminoglycosides and is involved in biofilm formation and motility**. *Microbiology* 2007, **153**:474-82.
6. Kim Y, Wang X, Zhang XS, Grigoriu S, Page R, Peti W, Wood TK: **Escherichia coli toxin/antitoxin pair MqsR/MqsA regulate toxin CspD**. *Environ Microbiol*  2010, **12**:1105-21.
7. Dubern JF, Lagendijk EL, Lugtenberg BJ, Bloemberg GV**:** **The heat shock genes *dnaK*, *dnaJ*, and *grpE* are involved in regulation of putisolvin biosynthesis in *Pseudomonas putida* PCL1445**. *J Bacteriol* 2005, **187**:5967-76.
8. Kuiper I, Lagendijk EL, Pickford R, Derrick JP, Lamers GEM, Thomas-Oates JE, Lugtenberg BJ., Bloemberg GV: **Characterization of two *Pseudomonas putida* lipopeptide biosurfactants, putisolvin I and II, which inhibit biofilm formation and break down existing biofilms**. *Mol Microbiol* 2003, **51**:97-113.
9. Rouvière PE, Gross CA**:** **SurA, a periplasmic protein with peptidyl-prolyl isomerase activity, participates in the assembly of outer membrane porins**. *Genes Dev* 1996, **10:**3170–3182.
10. Jackson DW, Suzuki K, Oakford L, Simecka JW, Hart ME, Romeo T: **Biofilm formation and dispersal under the influence of the global regulator CsrA of *Escherichia coli*.** *J Bacteriol* 2002, **184**:290-301.
11. Goodell EW, Higgins CF: **Uptake of cell wall peptides by Salmonella typhimurium and *Escherichia coli*.** *J Bacteriol* 1987, **169**:3861-3865.
12. Rudner DZ, LeDeaux JR, Ireton K, Grossman AD: **The Spo0K locus of *Bacillus subtilis* is homologous to oligopeptide permease locus and is required for sporulation and competence**. *J Bacteriol* 1991, **173**:1388-1398.
13. Leonard BA, Podbileski A, Hedberg PJ, Dunny GM: ***Enterococus faecalis* pheromone binding protein, PrgZ, recruits a chromosomal oligopeptide permease system to import sex pheromone cCF10 for induction of conjugation**. *Proc Natl Acad Sci USA* 1996, **93:**260-264.
14. Detmers FJM, Lanfermeijer FC, Poolman B: **Peptides and ATP binding cassette peptide transporters**. *Res Microbiol* 2001, **152**:245-258.
15. Cundell DR, Pearce BJ, Samderls J, Naughton AM, Masure HR: **Peptide permease from *Streptococcus pneumoneae* affect adherence to eukaryotic cells**. *Infect* *Immun* 1995, **63**:2493–2498.
16. Lee EM, Ahn SH, Park JH, Lee JH, Ahn SC, Kong IS: **Identification of oligopeptide permease (opp) gene cluster in *Vibrio fluvialis* and characterization of biofilm production by *oppA* knockout mutation.** *FEMS Microbiol Lett* 2004, **240**:21-30.
