# Supplementary material for: Environmental transcriptome analysis reveals physiological differences between biofilm and planktonic modes of life of the iron oxidizing bacteria Leptospirillum spp. in their natural microbial community
Source: BMC Genomics. 2010 Jun 24;11:404. doi: 10.1186/1471-2164-11-404 (PMC2996932; doi:10.1186/1471-2164-11-404)
Supplement: Additional file 2 — DNA sequences of the genes from Additional file 1 Table S2. [file 1471-2164-11-404-S2.DOC]

**Additional file 2**

Sequences of the genes described in this paper. Each entry has the name of the annotated gene as it is in Additional file Table S2, followed by the name of the *L. ferrooxidans* genome orf, a description, and finally the nucleotide sequence.

>lnt LfeRT32a_3194 Apolipoprotein N-acyltransferase

ATGCAACAAAAATCACTGGGAGAAGCTCTGACCACAGATAGGGAAAAGTCCTCCCGTAGAGGGTGGCTTTCTTTTTTTTC

AGCCATTGGAAATCACTGGGGAGAAGTTCTGCTTTTTGCGCTGTCGGGCTTTCTTCTTGGACTTTTCTTTATTCCTGACC

GGGTCTCTCCGCCGCTTCTTGCATTACTTCCGCTCGTGTTCATCCCCCTGTTCTTACCAGAAAAACGGAGTCAGGGAATG

TCTTTTTTCCTCGGATGGACATTCGGAACAGCGGAAGCAATCACCAGCCTTTGGTGGGTTGTCCCGACCATTGAACATTT

TGGACATCTCCCCTTCTCCCTGTCGTTTCTCTCCCTTTTTCTGCTTTCATCCTATCTGGGACTTTTTACAGGTTTTTTTC

AGTCAGGCATCGACCTTTGGAAAAGAAAAGGATCATCCGTCCTCTCCAATGCCGCCATTTTACCCCTTTTTGGGGCATGT

CTCTGGGTTATCACGGAGGTACTCAGGTCCGAACTCTTTACCGGATTTCCGCTCAACCCCCTTGGAGACCTGGTCTGGGG

GGAAAAGCTTCTGCTCCCCGACGTATCGATACTCGGCGCCACAGGGATTTCTTTTGCAACTGTTTTCATTTCCGGACTTT

TCTGTGAAAGCATTGCGCCTCTTCTCAGAAAAGTCTCTGGAGGACGTCTCTGGAAGGACGTTCAGCCCAAATACCTTTCT

TCCATTTTTTTTCTTTTTCTGACGATTTCCTCGCTTCTCATTTCAGGAGAGGTTCTCGGGAACAAATCAGGGAGTTCCCC

AATACCCTCGATAAAGGTCGGAATCATTCAGGGGAATATCCCCCAGGATCAAAAGTGGACCTCCCAATATCTGAAAGATA

TTCTCAAAACCTATCATGAGCTTTCAGAACAGGCTCTGGCACAAGGAGCAAAAATCCTTGTCTGGCCTGAAACGGCAGTT

CCTGTCGTAATCGACTCCCCTCCACGATCCGTCGCCTCTGACCTGAAAAAAGCACTGGAGCTTCCTGTTCCACTCGTTAC

CGGAACAATAGGGCTTGTCCGGGGAAAGGATCGTTTTGATTACTCCTTTTCAAACGATGCCATCATCAGATCCCAAACAG

GACAAACGCTCGGAAGCTATACAAAAATGCACCTCGTCCCGTTTGGTGAATACATTCCCTTTCCATCGATCTTCGGCTGG

CTCAGAAACTTAACCGGGATCACAGGCGACCTTGTTCCAGGCAAACAGCAGAAACTGTTCACCCCATTCAGCAACGCCCC

ATCCCGGTCCACGTCGGCGAATAACGGCAGCAGTATCCGGGTCGGACCGGTCATATGCTACGAGGCCATGTATCCGTCTC

TTGTACACCAGCTTGTCCTCAATGGAGCAAATATGCTCGTTGTCATCACTGATGATGCCTGGTATGGAAAAACGGCGGCA

TCCCACCAGCTCTACCAGCAAACCATGATGCGTGCAGTCGAGGAAGGGGTTCCAATGGTGAGAGCTGCCAACAGCGGGTA

TTCGGGAGCAATATCGGGAGAGGGCAGACTGCTCCGGACAAGTGGAAGATTTACAAAAGAGTCCATTGTGGTAACGGTTC

CACTCGACAGCCACCCAACATTTTACCAGAAGCACGGAGAGTGGATATTTCGTCTCTCCCTCCTCATCTTTCTTTTTCTC

CTTGCGATCAGGATT

>murB LfeRT32a_0889 UDP-N-acetylmuramate dehydrogenase

ATGACACATGCCGGGGAGATAAAAACGCTTAAGAACGAGCGTCTTTCAATGCATTCGACCATCCGGATCGGTGGCCCCGC

GGAGTATCTTTTCCTTCCGGAGACGGTTGAAGAGCTTATGGTAATTCTGGCGGACGTCTCGTCCGGGAGTTTGCCTTCTC

CCCTTCGCATTTTTGGAAAAGGGTCCAATATCCTTTTTGCTGACAAGGGACTTTCGGGAACCGTCATCAGTACAAAGCGC

CTGACAGCCATGACAGTCCTGCCGGATGGCAGTTTCCTGGCCCAGTCAGGTGTTTCGATGCCTTATCTTGCCCGTGTGGC

TGCTACTGCCGGGGTGTCCGGATTTTCGTTTATGTCCGGTATCCCCGGAACAGTTGGCGGTGGGGTGGCCATGAATGCCG

GGACTCCGGACGGAGATTTTTCCCGGATCGTCCGGGAAGTTCGTGTCCTTTCCCCATCAGGGACGATTACCCGGCTGACG

GGGGAAGACCTGCAATTCTCCTACCGGCATTCCATCTTTTCTGGGGTGCATTCAAAAACGATGGGCACTGGTTCAGGTTC

AAATCTTCAAGGTGGATCGATGATCCTCGATGTTCTTTTGTCCGGTGTTCCAGCTGATCCTCTCCTGTTGCAGGAAGCAG

GAAAAATGGCCTTGTTACGCAGAAGTGAGAGCCAGCCGCTCGATCAGCCAAGTTTGGGGTCTGTTTTCAGAAATCCTCAA

CCGGACTTTGCCGGACGCCTTATTGAACAGTCAGGGTTGAAGGGTGCCAAAAAGGGCGGTATCAGGATCAGTCCCAAGCA

CTGTAACTTTTTTATTAATGAAGGGTCCGGGACGGCAGGTGAGTTTCTCGACCTGATGGCCGAGGCGCGTCAACGTGTTC

AGGATTTGACAGGCGTTGTCCTGTTTCCGGAGATTCTCAATTTTTTC

>murA LfeRT32a_1717 UDP-N-acetylglucosamine 1-carboxyvinyltransferase

TTGGACCGCTTCCGTATCGAGGGCCGGCAGTTGCTAAACGGAATGATCCCTGTATCGGGATCAAAAAACGCAGCTTTGCC

CATTTTGTTTTCCACATTGCTGGGAGGGAACCTCAGAGTCGGGAACATCCCGCATTTGAGAGATATTACAACCGCCATCA

AACTCCTGGTCCAGCTGGGAGTTCAGGCAGAGTCCATTGCCTCTCCGGCTGACTGGACCGGTTCCGTCTCTTTTTCGGAA

ATGGATCTTTCTCCGACTGAGGCTCCGTATGATCTTGTCAGGGTCATGCGAGCCTCAATCCTCTGTCTTGGTCCCCTTTT

GTCCAGACGCCGGAGAGCCCGGGTGTCGCTTCCTGGCGGGTGTCTGATCGGGGCTCGGCCGGTCGACCTGCACCTCCATG

CTCTCCAGAAAATGGGGGCCCGCATCTCGATTGACCATGGCTATATTGATGCTTCAACCGATGGTTTGGTGGGTTGCGAC

ATTCACTTGTCCTACCCGACGGTGACCGGAACCGAAAATATTCTCATGGCAGCGGTCCTTGCTTCCGGGACAACCAGGAT

TTCGAATGCCGCACAGGAGCCCGAGATTGCTGATCTGGCCAACTGCCTGGTGGCCAGGGGCGCAAAAATTTCGGGGATTG

GATCATCTGTGCTGGAGATATCGGGTGTCGCTGAACTCAGGGATGCCAGTTATGACGTTATGTTCGACAGGATCGAGGCA

GGGACATTCCTTGTTGCGGGAGCTCTCATGGGAGATCCCCTGACTGTTTCGGGGGTTGTTCCGGAACAGATGTCCTCGAT

TCTCGCAACGCTTGGAGAGATGGGAGCCGAAATTGAGATCATGGGAGACAGGATCACCCTTTCCAGAGTTGTTTCCCCGC

AGGGTGCGACGGTGATGACCGATCCCTATCCCGGCTTTCCGACGGACATGCAGGCTCAGATTCTTCCCCTGATGGCAACG

GCGACCGGTCCATCGACGCTGATTGAAACGGTCTTTGAAAGCCGGTTTACCCATGTCATGGAAATGAATCGTATGGGAGC

CAATATTGAAGTTCGGGGTTCCCATGCCTTTGTCAGGGGAGAGTGTTCCCTTGAGGGAGCGTGCGTGATGGCGTCTGATC

TCCGGGCCTCTGCGGGGTTGGTTCTTGCTGGACTCGTGGCTCATGGAGAGACCGTTGTCCAGCGGGTCTACCATATTGAC

CGGGGATATGAGAGGATCGAGGAAAAGTTATCGGGAGTTGGCGGAAGAATCTGGCGCGAAAGTGAAGAGGTT

>slt LfeRT32a_1737 Soluble lytic murein transglycosylase

ATGAGCTGGCTTATAACCCAGTCCGTGTCCTGGTGTATTGGGGCACATCTTCTGACATCATCAGTTCCCTATCAGGCAAC

CATTACAAAAATCGCCTGTACGCATCACGTGAATCCAACACTGGTCGCCGCCATTATCCAGAAAGAATCCGGGTTCCATC

GGCGAAAAAGACGGGTTGAGCCCGCCATTCACGACATTTCAAGGGGACTTATGCAGATCACCCTCGGAACAGCAAGAATG

ATGGGTTTCCGGGGAAACCCCCGCAAACTTTACTCCCCATTGGTCAATATCCGGTATGGAGTCCGTTATCTGGCTTATTT

GCTGAAACGATATCCTTCGGGAGAGGATGCTATTGCGGCCTATAATGATGGACACCCCCACTTCAGGCGGGGACACTATG

TCAACTCAAGAGGGGGATATTCTGTCCAGCGCTACGTCAGCGATGTCCTCAGGAACACCAAAAATCTCATGATCGCATCT

GTCGAGAACTGGGATCTGGGAAACCATCTGGGATTCATGGGGGAACGCCCACCCTCACTTAACTCCAGATGGCTTCTGGC

CGATATTGGTCTTTCCGGGAATAACCATCCTGGGAGTGGCAGGACGGGTTTT

>ugtP LfeRT32a_0734 Diacylglycerol glucosyltransferase (membrane glycolipids formation)

GTGAAGGAGCCCTGTCTGGCAAAAATCATGGTGCTGTATGCCTCGATCGGCAGCGGTCATGAAAAGGCGGCCGAGGCGAT

TGTTGAGTCCCTGGTTTCTGGGGGGGAGAGGCACGATGTCTGCCTGATCGATGTCCTTTCCCTGATGAATCCACTGTACA

GAAACCTCGTTCCTCGGGGATATATCTGGCTGGCCAGATACTTCCCCCCTGTTTTGGGGTTTTTGTATCGTTTTTCAGAC

CATTCCTTCTCGATTCTGCCTCCTGCAAGATTTTTTCGGTCGCTTCTATCACGTCTGTTTTCCAGCGGATTCAAGCGGTT

CGTTCGTGAGTTTTCCCCCAAAATGATTGTCTGTACCCATTTCCTTCCGATGGAACTTTTGTCGTCGGACTTGATCTCCG

AGGGGTCCCCGCTTCTCTATGTTTCGATAACGGACATTATTCCCCATGCCTTCTGGATTGCTCCCGGGGTTGAGCGGTAT

TTTGTCGCCGCCGATGAATCGGTCAAGAGGATGAAGGACTTTGGGGTGCCGGATGAAAAGATCAGTGTGTCGGGAATTCC

TGTTCACCCTTCGTTTAAGTGGGCGAGGGTGGCATGTCCGGCGGTAGCCGAAAGAGCGGTCCCGTTGAAGCTTCTCGTCG

TGGCCGGAGGGGCTGGAGTTGGTCCCATTGAAAAGCTGCTTGAAGGGCTCTCCGGATCTCACCCGTCGATCTCCGTTACG

GTTGTCGCCGGGAGCAACCGGGCTCTCTTCAGAAGAGTTTTCCGGAAAAGGTTTTCCTATTCTTTTCCGGTCGAGGTCAG

GAGATATACGAAAAGAATGAGCCTCTTGATGGAGCAGGCCGATCTTGTGCTCACAAAGCCCGGTGGCTTGACGACAGCGG

AGTGTCTTGCCATCGGAAAGCCGATGATTCTTTTTTCTCCGATCCCGGGCCAGGAAGAAGACAATCTTGATATTCTGAAA

GCTTGGGGGGTGGCCAAAAGTCTTTCTCCGGCAGAGAAGCCACCCTGTTTTCTGGAGGATTTGTTAAAAAACCGGCAGGA

ATTGTTTTCCATGGAGGAAAGGGCAAGAGAGCGCGGGCGTCCGGACGCTTCAAGGGTCGTTTGCCAGGAGATCCTGCTCT

CCTTTCATGAAACCAGAAGCAAGGATGGGGGAAGCTCCTTGTCATGGAAGCTTTATCGGGCTGGGAGATGGGCGTTTTCC

AAATGGTTC

>mrcB LfeRT32a_3775 Peptidoglycan glycosyltransferase. Murein Polymerase

GTGACACAAGAATCTCCGACAGGGCCGGACGGACAGGATAACAGTCTCCCTCTAGCAGAAGACGCCGGTCCGGGGAAAGC

CAAAAAACCTTCATTGCCACCCATTCTATCAGGACCGGCCGGCACAAAAAAGACTCTCTGGCTGATCCTGTCCACACTTG

CCCTCGTTATTCTGGCCGTGGTCTGGGGATTCTCCCACCACCTGGACACAATGCTCTCTCACCGCCTGAAGATCCTTGCC

TCTCCCGGAGTTACCGTTTACTCCTCTTCTTTTTCCGTCTACCGGGGAGAAGAGATCACGCCATCCCTGATTCGACAACT

CGAGCCGGATACCAAAACACTGAACGTCCGAACAATCATTGTGACGGAAAATCATGCACTCCATACTCAAGGAAGACAAT

TCAAGCTTGTTTTCTCCCGAAATCTCAGGTACCTGGTCGCCATCAGCCCCATTGAGGGGATCGCCTCCGGCCTCCCCCCC

ACAGAATCGGTCCTCCTCCCTCCGGGATTTCTCGGAACCATCATCGACAAGACTCTTGCCCTGTATCGACCGATCACACT

GGATCATGTCTCTGACAAAATGAAGACCACACTCCTCGTCTCCGAAGACCGGAACTTCTACCAATCCCCCGCCCTTGACA

TCACAGGCATACTCCGGGCCTTCCTTCTGGATCTCCGCTCCCGGACATTCCGGGAGGGGGGCAGCACGCTGACCCAGCAG

GTAGTCAAGAATATCCTTCTCGGGCAACAGAAAACCATCGAACGAAAGTTCCTGGAAGTCCTTCTCGCTATCAAGCTCTC

AAGAACCCACACACCCGACCAGATCCTGACCCTCTACCTCAATCACACCGAATGGGGCACCAGCGGTTCAGAGAGAATCA

TTGGAATCGAAGCGGCCAGCGAGACCTTTTTCGGCCATTCGGCAAATCTCCTGAACTATCGTGAGTCGGCAACGCTTGCC

GCCATTCTCAGGGCACCGAACCGGAACAACCCCGTCCGGCACCCGAAAAGGGTCATCGCCATCAGGAACAGGATCCTGGA

GAGCCTCGGAGAGACCGGGCACCTGAAAGGCAGGCTTCTTCAAAGGGATCTGAAAAGCCCTCTCGGAATTTCGGCCAGAA

TCCCAAAGCCGCCGGGCCCCTACTTCACATCCTGGGCCATCAAAACAATCGGCCCTCTTCCGGCGGGAGCCAGTGTCACG

CTGACGATGGATCCTGTCCTCTCGGAAAAAGTCGATCAGATCGTGGCAGGAGACCTTCAACAGATCGATCGCATCATTCA

GAAGCACCACCCCCAAAAGCCACCCCTTGAAGCCGCCGCCATTGTCATGAATCCTCAGTCGGGGGCGATCCTTGCCCTGT

CAGGAGGGGACTCCTTTCGACTGGCGCCGTTCAATCGCGCCATCATGGCCAAACGCCAACCGGCATCGCTATTCAAGATT

GTTCCCTATATCGTCGCCCTCAATCCGCGAGGAGGCGGCCCCCCCCATGCCAATATCGAGACAGTCCTTTCAAACGATCC

CATTGCCGTAACGGCCGGAGGCCATTTATGGCGCCCCAGGAATGCCGAAAAGATCGAAGACAGCAAAATCACCCTTGAAG

AGGCCTTTACCCAGTCATTAAACCGTCCGATCCTCCATCTCGCGCGCATACTTTCGCCGGCGGAGATGGTCGCCACCGCC

AGAAGCCTGGGGCTCGACACGCCGGATGCGGGAGACCTGCCTCTCTCCTGGCCACTTGGAGTGACGCCACAGACTCCCCT

CCAGATAGCACGGGCTTATGCCGCTATTGCCAACGGGGGATACGCAGTCACTCCCCATACAATTACCAGTATCCGTTCGG

AAACAGGAACGCCGATCAAAAGCTTTCACCCGCTTCCTCCTGTTGTCCGCCGGGTTATCCCGGCCCAGACAGCCTACCTT

GTCGGGGACCTCCTGCGAAGGACCGTCTCTTCAGGAACCGGGAGACCCCTGGCCCGATTCACAAACCCGGATGGCTGGGG

AGGAAAGACAGGAACGGCCAACAAGGGCCGGGACACCTGGTTCGTTGCGACAAGTCCATCACGGATCCTCGTTGTCTGGG

CCGGGTACGACGACAACTCGCCGACATGGCGATTCGGTGCCACCCTTGCCCTTCCGATCGCCGGGAAAATCATCCGGCTC

ATGAATCTCACCCCGTCAATTCCGCCTCCGCCACCCGGGATACTGATGAACCTTGTGAGAACCGACTGCAATGCCCCTCC

CCGGATGATCCCGTCGATCGAGGGGAACCCGATCAATTCATCCGATTGCCAGGACACGGGGAAATCTCCCGGGCTCATGG

ACCAGATGGGGCTGTTTTTCAAAAAGATCTTC

>shc LfeRT32a_2499 Squalene hopane cyclase (triterpenes synthesis)

ATGGAGAAAGTAGCTTCCCCCAATGCAGGGGACCGGTCCTTATCGGATGACGAGAAACCGTTTCTGGATTCGAAAGGGAG

TTCTGGCTCCAGTTTTAGGGAAAGACTGGAAGAAGCCATCGCAAAGGCTGCCAGATCTCTTCTGGATCGCCAGCATCCGG

AGGGGTATTGGGTCGAACCTCTTGATGCAGATGTAACCATTCCTGCTGAGTATGTCATGTTTCAGTTTATCCTCGGGAGA

AAGGATGAGGTTTTTTTCCGCGATGTAGCGGAGTACATCCTTTCGATTCAGGGTGAGGATGGGGGGTGGCCTTTATTTAC

AGGAGGGGCAGCTGACATCAGTGCCAGCGTCAAGGCTTATTTTGCCTTGAAGCTTTTGGGGTATTCTCCCGATCATCCGG

CTCTGGCAAAAGCGCGTGCGCTCATTCTTGAAAGGGGCGGAGCCACTACTGTCAATGTTTTTACCCGGATCACCCTGGCT

CTTTTTGGGCAATACGACTGGAAGGGTATTCCGGCCTTGCCTTGTGAAATGATTCTGCTGCCAAAATGGTTCCCTCTTTC

GATCTATACCGTTTCCTACTGGTCAAGAACGGTCATTGTTCCCTTGCTGATCATCTATCACTATAAGCCGGTTACCCCGG

TTCCTCCGGGACAGGGAATGGATGAGCTGTTTTTAAAACCGATGGAGGAGGTTCACTTTGGCTATTCCTGGGATAAAAAA

CTTTTTTCCTGGAAAAACCTGTTTTTTGTCCTGGATTATTTTATCCAGCACTGGAACCGCCACCCCCCTGGATTTTTAAG

GAAAAGAGCTCTTGCAAGAGCTGTCGAATGGCTGATTCCCAGAATGAAAGGAGAGGGGGGCCTCGGTGCGATTTATCCGG

CAATGGCCAACAGTGTCATTGCAATGAGGCTTTCAGGGTACTCGGATGATCATCCGCTTGTGAAAAGGGCTATTGCATCG

ATTGATGACCTTGTCTTTGCCCGGGACAACATGCAGTCCGTTCAGCCTTGCCATTCTCCGATATGGGATACTGCGCTCAG

CCTTGGGGCCCTTTTTGAGGCGGGTGTTTCTGCCGATCATCCGGCAATTACCCGCGCACTTGAATGGTTTCGCAGAAAAG

AAGTCAGGACTGTTGGCGACTGGTCGGTTCATGTCAAGGGTGTCGAGCCTGGTGGTTGGGCCTTTGAATTTGAAAACGAT

TACTACCCTGACGTTGACGATACTGCTGTTATTCTGATGGACTTTGCCAAGTGGACCAATGGATTCAAGGGATATGAAGA

TGTTGTGAGGAGAGCTGCCAGATGGGTTCTGGCAATGCAGTGCACGGATGGGGGCTGGGCCTCCTTTGACAAGGACAATG

ATCTCCTGTTTTTAAACAACATACCTTTTGCCGATCACGGGGCGCTGCTCGATCCGTCAACGGCCGATCTGACGGGGCGT

GTCCTGGAGTTTTTGGGGCTGTACGGTTATCGGCCCGATTTTCCACCGGTTGCGCGGGCTCTCGATTACTTGCGTCGTGA

GCAGGAAGCTGACGGTTCATGGTATGGTCGATGGGGAGTCAACTATATTTATGGCACATGGTCTGTGATTTCGGCATTGA

AGGCGTTGGGGATCGATATGAAGTCATCGATGGTTCAGCGTGCAATGGGCTTTTTACTTGATCACCAGAATCCAGATGGG

GGATGGGGCGAAAGCTGCCTCTCCTATTACAAAAAAGAGTCTGCAGGTGTGGGGGATTCAACTCCATCGCAAACAGCGTG

GGCTCTTATTGCGCTGGTTCATGGGGAACATGCGGACAACCCAAAAGTCCGGAAGGGAGTTTCCTGGCTTCTTGAGAATA

TGCGACAAGATGGCCGCTGGGATGAATCACTCTATACCGGAACCGGTTTCCCCAGGGTTTTTTATCTTCGCTATAACATG

TACAGAGACTATTTCCCGTTGTGGGCACTAGCCCTTTACCAGAATGTCCATTTCGAGGGGGCTTCCCGTGTTTCCGGGAA

AGTAGCCGTATGGAGGAAGCAGCCCTTTGCCCCTCTCGCCTCATTTTTC

>uppS LfeRT32a_0709 Di-trans, poly-cis-decaprenylcistransferase

ATGAAGCATTCGGTCAACACCGAATCGCCCCATAGCGAAAAAGTCGATCTTTCCTGGCTGACGAACCCTCCCCGCCATGT

GGCGGTCATTATGGACGGAAACGGCAGATGGGCGACCAAGCGCCATCTGCCGAGGGTCATGGGACACCGAGCGGGGGCCG

AGAGCGTCAGAAGAGTTGTCACCGCATCGCGGCAGTGGAATATCCCGTATTTGACCCTCTATGCATTTTCGTGGGAAAAT

TGGACAAGACCGCGTCTGGAGGTCAATGCGCTGATGGCTCTTCTCGAGGAGTTTATCGATCAGGAAATCCAGACGATGCT

CAATAATTCCATCCGCTTCTTTGTTGTGGGCGCGCGGGAGCGTTTGCCGGAATCGGTTCTCAGGAAGATACGGATGGCGG

AGGAGCGGACCGCGGACTGTAATCACATGGTTTTGACGCTCGCATTGTCCTATTCAGGGAGAGAAGAGATCGTAAAGGCG

GCGGCCACATTTGCAAGGGATGTCCAGAAGGGGATCATCACCCCCGAAGAGCTTTCGGCAGAACGTTTTTCCGGGTACCT

CGATTCACGGGACCTTCCTCCTCCGGATCTTTTGATCCGGACCAGCGGTGAGGTGCGGATCAGCAATTTTCTCTTGTGGC

AGATTGCTTATACCGAACTTCATTTTACGCGGACGCTGTGGCCGGACTTTTCGGAGGACGATTACCGTCGGGCATTGATG

GACTATCAGGAAAGGGTTCGACGTTTCGGCAGAACCGGGGCGACCCCTGACGAAGGCACCATT

>mqsR LfeRT32a_2279 Motility and quorum sensing regulator

ATGGGACTTGGGTTTTCTGACATGCTGGCCATAGTGCTGGCACTCGAACCAGCGGATTTCTACAAAAGCATGACCACGAA

CGCCGATCACAAGGTTTGGCAGGATGTGTACCGCCCGAGCACCCAGGCGGGCGATGTTTATCTGAAGCTGACGGTCATTG

GCGATGTGTTAATTGTGTCCTTCAAGGAGCTA

>fliA LfeRT32a_1818 Motility and flagellar sigma factor sigma 28

ATGATGGAGCTTGATGAGGCCAGCCTGAAAGAGTTTGCTGTCACGGTGAAGTTTTTTGCCATGCGGTATGCCCATCGTCT

CCCTCCTGAGATCGATGTGGATGATCTCGTCAGTGTCGGAATGACAGGGTTGATCGATGCGGCAAAGAGATTTGATCCTT

CAAGGGGGATCAAGTTCAAGACGATGGCGGAGCATCGAATCCGGGGAGCGATGCTCGACGAGATCCGTTCGGCGGATTGG

GTCCCCAGATCGGTCAGGGAAAAGGCGTCATCGGTTCATGCCGTAAGGGAAGGGTTGCGTGCGGCTTTGCTGAGGGAGCC

GGACGACATGGAGATGGCCAAGGCACTCTCCATGTCGCTTGACGAGTATCTGACGCTTGAAAACGAGATCGAGCCTCACC

ACCTTTATTCGATCGAGGATCTCTTCGAGATGGAGGAGGGAGCCGGATCGTCGATTCTGGACCGGATGGTTGTTCCGGGG

GAGGCAGATCCTCTGTCGGAATTTCTGAAAAGCGAGGAAGCCAGCCTTCTGGAGAAGGCGCTTGATTCCCTTCCGGAAAA

GCAGCGACTGGTTCTGTCCCTGTACTATTACGAGGAGCTTTCCATGAAGGAGGTTGCAGCGGTCCTTGAAGTCAGCGAAT

CAAGAGTGTCCCAGATCCATACTCTTGCGATCAAGAACCTGAAAAAAGCACTTCTTTCCCTGGGTCATAAAGAAAGC

>cheY LfeRT32a_1817 Chemotaxis protein CheY

ATGAAAGTGCTGGTTGTTGACGATTTTTCCACGATGAGGAGGATTGTCAAAAACACCCTCCGTCAGATCGGATTCGTCAA

TATAGAAGAAGCCGAGGATGGGCAGAAAGCCTATGACCGGCTCGTTTCCGAGAAATTCGATTTTGTCGTGTCTGACTGGA

ATATGCCGAACATGACCGGGATTGATCTGCTTCGAAAGGTGCGGGCTACCCCCTCGATCAAGAATATTCCGTTTTTGATG

GTGACGGCCGAGGCGAAGCAGGAAAACGTTGTGGAGGCGATCAAGGCAGGCGTTTCAAATTATATCGTCAAGCCTTTCAC

CGTGGGAACACTTCAGGAGAAGGTTGCGAAGATTTTCAAGGAA

>cheA LfeRT32a_1816 Chemotaxis protein CheA

GTGGCGGAAGGCTTTGATAATGACGAGATGAAAGAAATTGTTCAGGAATTTCTTGCAGAGGCGCAGGAAATGCTTGAAGG

ATTGGACAATTATTTCGTCCAGCTTGAAGCAAGGCCTGATGACACATCGCTTTTGAACGAAATTTTCCGGGCGGCCCACA

GCATCAAGGGGTCGGCGGGATTTATCGGATTGACCCGGATTGTCGAGGTTGCCCATCATGCCGAAAATGTCCTGAACCAG

TTGCGCCAGGGAATGATGAAGGCGGAACCCGCGGTGATCGACATCATTCTTGAAGCGATGGATGCTCTGAAGCTTTTACT

CGAGGAAGTTCACACGGGTACCCAGGCCGATGTGGATATCGACACTCTCACGATGAAGCTTGATCTCCTGCTCCAGTGGG

GAGAAGACCAGGCCGCATCTCATGAGTCGACCGGGCCAAAAGCTCCTGACACGGAAGAGGCAGGGATGGTAGTGCCTCCG

GAGGAGCCGGAGCCCAAAGAGCCAGAGCCCGGGGAGCCCGTCTCTTCCCCCCCGTCCGCTGAAATTGTTTCGGGTGGCCA

GTCTGCCCATCCGCCATCACCCCCGACCCCGCCACCCTCTGCTCCTCCCCAGTCCGAAGCGGCCCAGGTCCAATCCTCCG

GCGTCGAGGTTGACCAGACGATCCGGGTCGAGACTTCGAGGCTGGATAATGTCATGAACCTTGTCGGAGAGCTCGTTCTT

GGACGGAACCGCCTGGTCCGGCTTGCGACAGATACCCGCGGCGATGATGACTGGGAGAAACAGCAGAAGGACATTGCGGA

AGCGGTTATCCAGCTTTCGAGAGTGACGACGGACCTCCAGCTTGCGGTCATCAAGACCAGAATGCAGCCCATCAAGAAAG

TTCTCGGAAAGTTCCCCAGGATGGTCAGGGATCTGTCGAGAAAGCTCGGAAAGGAAGCGCGTCTCGAGCTTTCCGGAGAA

GAAACGGAGCTTGATAAATCGGTCATCGAAGAGATCGGTGATCCTCTGGTGCATATTATCCGCAATGCGATCGACCATGG

ACTCGAGATGCCGGAAGAGCGCCTTGCCGCCGGGAAAAATGCTGAAGGAATTGTCAGAATATCCGCTTATCAGGAGGGGA

ACTCCATTGTCATCGAGATATCGGATGACGGCCGCGGGGTCAATGTGGACAGGGTCCGGAAAAAGGCCATTGAACGGAAT

CTGATTTCGGCATCAGATGCTGACCGGATGACAACCGAAGAGCTGGTCAACCTGATTTTCCTGCCGGGGTTCAGCACCGC

CGAGAAAGTGACCGATGTTTCCGGTCGCGGTGTCGGGATGGATGTTGTGAGGACGAATATCAACAAGATCAACGGTACCG

TCGAGATCCGTTCCCAACAGGGCCTTGGTTCGACCTTTGTGATCAATCTCCCGTTGACGATCGCCATTATCCAGGCATTG

ATGGTTGCAATCGGAAATGAGGTCTATGCGGTGCCCCTCCAGTCCGTGGTTGAGACAGTCAAGATTTCCGAGTCGGATAT

CCGGACCCTGTCCGGGGCAGAGGTCCTGAACCTCCGCAATCAGGTGTTGCCTCTCCTGCGGCTCCGGGATGAGTTCAAGA

TTCCCGGTGAGGCCAATGAATCGGCGGGAAAGAGCCGGTATGTGGTGGTTGTCCAGATCGGATCCCGGTCGGTTGGGCTT

GTTGTAGAGGCTCTTCCCTACCAGGAGGAAGTTGTCATCAAGAGCATGGGGCCAATTCTTTCAGGTATCCGGGGTATGGC

CGGAGCAACGATAACCGGGGATGGAAAAGTCGTTCTTATCCTTGATGTCGGAGAGATACTGCAGGATATCCAGATCCGGG

GTCACCAGGGTGTTTCTGCGGTTGCCAGA

>mcp LfeRT32a_1814 Methyl accepting chemotaxis protein C

TTGAAACTGCAGGGAAAAATCCAGCTGTCCGTTTTTTTGATCCTGTTTCTCTTCCTCTCGGTAACCGGGGTGTTGACCCT

TTACAAGGTTTCAGGCGATCTGATGCACCAGGAAAAAAGACGCTCCGACATGATGGGCCAGTCCATCATCCGGAGCCTTT

CGACGGTCATGATGTCGGTCAATGCTCCCGTGTTATCCCAGAAAATTGTCGAAGACCAGAAACACCTCGAAGGAATTTTG

AGGGTTCAGGTCCTTCGTCCATCCGGCAAGCAGGCCTTTTTCGACAACAAGGAAATTGACAGGGTCAATCTCTGGCGCCA

CTACAGGGCCTATTCTCCCCGCACCTTTCTGTTCTCTCCCAAACATGACTCCGGTCGGTTTGCGCAGGACCAGCACTTCA

GGGACGTTCTCGCATATGGTCGTGCTGTTTCCTTCCAGGAGAAAATTGATGGAGTCCCCGCGCTGACGAGGCTTATGCCC

ATCAAGATTTCCAACAACTGCCTCCTTTGCCATGGGTTCCAGAAAGATCAGCCAGTGATGGCGGTTCTGAGAATCAGTAC

GCCTCTTACGGAATTCAATCATTCAAAAAACTCGATGATGATCGAGATTGCGGGCCTGTCGTTTGCAACAGTGATTGTGC

TCCTGGCTCTTCTCTCCCTCACGATGAGATCGCTTGCCATCCGTCCTATCCAGGAAATCGTTGAAGTCATCGAGCAGACA

TCACAAGGTGACCTCACCCGGGTTGTGCATCCGAAAACCAGCGACGAGATCGGAAGCCTGATGACCCATTTCAATGAAAT

GGTCATGAAGATTCGGGAGGTTGTTGTGAAGCAGAGGGAAGAGGCGTCCCGTGTCATGCTGGTTGCCAAGGAAATTATCG

GAAAGCTTGATGGGATACGCTCCCGCACCGACCATGAGGCGGAAATGATCGCTGGTGCGGCCCACGAGACGGAAAAACTT

TCGGATTCGATCCGGTCTGTCAGTCAAAACACCCGCTCCCTGGCGGAGCTTTCCACCAAAACGGACCGGGAGGCCCAGCG

CGGACTGGAGTCGATTCAGCGTGCGGGACAGGAGCTGGCAAGGATCTCCGGTGTGGTTTCGGACGCGACCAGGAGCATTC

TGGAGCTTGGCAAGTCCTCTGAAGAAATCTCTCAGATCATCACGATTATTGACGAGATCGCAGAGCAGACCAATCTTCTT

GCCTTGAATGCGGCGATTGAGGCTGCCCGTGCCGGCGAACAGGGCAAGGGTTTTGCGGTTGTTGCCGATGAGGTCAGGAA

GCTTGCCGAGAGGACAACTCTTTCTACGCGGGAGATTGCCGAGACGATAAAGTCTATCCAGGTTCAGACCGAAAAGTCAG

TAAGGGTCATGTCTTCGGGATCGCAGGAAATGGTTGACCTGATGTCGGTCATGGAGGATGCTTCCGCGTTGCTCGCAGGC

ATTACCTCATCCGTGACCGAAGTGACTCTTCGCGTCAACGAGATTGCAGAAGCCTCAGCCCGTCAGAGCGAGGCTGTTTC

CAACGTGACGCAAGCCGTTGAGAGCTCTTCCAGGGGGGTTCAGCTGATCCGCCAGAATGCGAAAGAATCGGGGGAGGCGG

GGATAGAGATGGATTCCAGGATGAAGGAGCTCGAGCGCTACCTGGCACAGTTCAGGACGGAA

>cheW LfeRT32a_1813 Chemotaxis protein CheW

ATGCAGGAGCTTTCGACAACGAAATCCATCGAACCTTATTCGGAAGGAGTCCTCTCAGGGAGGGACTTTGACGGTGGGGA

TGAAATCCTTCAGCTGGTCAGTTTTACTCTTGCGGGAGAGAATTATGGCTGTGAAGTTATTCACGTGCAGGAGATCAACC

GGCTTTCTGATTTGACCCGGGTTCCTAAAGCTCCCCATTATGTTGACGGTGTGGTCAACCTCAGGGGAAAGATCCTGCCG

GTCATCAATTTCAGGAGGCTTCTCGGGTTTCCGTCGGCATCAGAGATCACGGAGGACATGCGAACGATTGTTGTGAATGC

CGAAGGAATTCTTGCCGGTCTGACCGTGGATTCTGTCAACCAGGTTATCAGGATTCCGCGAAAGGATATTGAAGGACGCC

AGGACTTCAATATGGCGGGAAGCTTTGGCGATGCCATTACCGGGGTGGCCCATCTTGAGGATTCTCTGGTAACGATTATT

GATATCATGAGCCTCCTCAGAAGCCATCAGTCCGATGTTTCGGGATCCGGGCGT

>motA LfeRT32a_1812 Motility protein MotA

ATGGATATCACAACGCTTCTGGGTTTTTTGATCGGTATCGGAGGGATCCTTGGCGGGGCGACCATGGAAGGGTTGCCTCT

CGGAACCATTTTTCAGCTGACCGCTGCCATTATTGTCTTTGGGGGAACGATCGGGGCGACGATGGTGACAACGCCTCTTC

CCCAGGTGATTGCCGCTGTCAAGGGAATCCCCAGGCTGTTTCTGAACTCCAAGTCCGATCCCGTCCCTCTGATTCTGAAG

ATTGTCGAACTTGCGAAGGTATCGAGAAAGGAAGGTCTTTTAAAGCTTGAGGCCTATCTTGAAGATCCGTTTATCAAGGC

CAATGCTTTTTTTACCCGTGGCGTCAGGATGGTGATGGACGGAACGGACATCGCAAAGGTCCGCGAGGCGCTTGAGAGCG

AATCGTTCTACATGGAAGAAGAGGAGGGCGGGGCCGCGAAGGTTTTTGAGGCGGCTGGCGGTTATGCTCCGACCATCGGT

ATCCTTGGGGCGGTTCTGGGGCTGATTCATGTCATGAGTAACCTCTCCGATCCCAACAAGCTTGCGGAAGGTATCGCGAC

AGCCTTTGTCGCTACTGTATATGGGGTGGGTTCTGCCAACCTGATTTTTTTGCCGCTTTCGGGAAAGCTCAAGATCAAGA

ATCGCTCCGAAGGAAAGTTTCGTGAAATGATCATTGCGGGTCTGGTCGCAATCGGCCAGGGAGAAAATCCGAACAATATC

CAGGACCTTCTCGCAGGATTCTTAAGCGAGGCGGAGAGGGTGCATCTGCCTTTGACC

>motB LfeRT32a_1811 Motility protein MotB

ATGGCCAAAAAAGCCCGTCATGAGGAACATGAAAATCTGGAGCGCTGGCTGGTTTCCTATGCCGATTTTATAACGCTCCT

TTTTGCCTTTTTTGTGATGCTTTACGCAATTTCCTCCCTGAATACCGGAAAATTCCGGGTCATGTCCAACGCGATCACCG

CCACTTTCCAGCATAAAAAAGTGATTGGTTCGACACATGTGATTATTCCGAAAGACAACACCCAGCAGTCCGGAAAGGTG

ACCCCCAATACACAGGCGGTGATGGTCCAGGCCATTCAGCTTATGGTTGAGAAGTCATCACGGAAGGGTAATATGGAGGT

TGTCCAGACAAAGCATGGGATTGTGCTCAGGATCCAGTCGAAGCTTCTTTTTGAGTCGGGCCATGCCAAAATTCGCTCCC

GGGCCTTGCCTGTCCTTAAATCCGTGGCAGGAATCCTGGCCAAGTCTCAAAAAGAGATCCGCATCAGTGGCTATACGGAC

AATCAACCGATCCGGACATCAAGGTATCCCAATAACTGGGTCCTTTCCACAATGAGAGCGGTCAATGTGCTGACGGAATT

GATCCGCGATGGTCCGCTCAGTCCTCAAAGGGCGGGAGCTGCGGGATTCGGACGCTACCGGCCGATCGCTTCCAACCTGA

CGGCGTCAGGACGGGAGAAAAATCGCCGTGTCGAAATCCTGATCCTGAACAGGGAGTATCACCCTCCCCATGTCCTTCCC

ATGGAGAATGCCGGTCCGGGCCCGCACCAGACATTTCCGAACCGGAAGGCGGACCAGGCGAAGCCATTGCCTCCTCCGGA

TATT

>fliM LfeRT32a_3777 Flagellar motor switch protein FliM

ATGGCTGAAAGTATCCTTTCCCAGGATGAGGTCAATGCCCTTTTGAGGGGGCTTTCGGATGGAGATATCGATACCGAGGG

GTCCGGGAAGGAAAAGCCCGAAGATGGCGATACCCATCTTTACAATCTGGCCAGCCAGGAGAGGGTCATCCGGGGAAGGA

TGCCGACCCTCGAGGTGATCAACGAACGTTTTGCCCGATTTTTTCAGGTGACTCTTTCGGCAACTCTCCGCAAGAACATT

GAATTTGCCCCGCAGGGAATCGACATGCTTAAGTTCGGGGAGTTTCTCCGGAAACTTCCGATGCCATCGAATATCAACAT

ACTGCGGCTTGAATCCCTTCGGAGAAACATCCTCCTGGTGATCGATGCACGATTGGTCTATCTCATCGTGGATCACATCT

TTGGTGGAAATGGCAGGGGACATGTGAAGGTTGAAGGGAGAGACTTTACTCCGATCGAGGCCAGAATCACCCGGAATATC

CTGGATCTTGCCATCGACGACTTCGAAAAGGCCTGGGCACCGGTCTACAGCATGCCGCTGACCTACATCCGCTCGGAGAT

CAATCCCCAGTTCGCGGCGATCGTGGCTCCGACGGAAATGGTCATTACCCTGGCTTACAAGCTTGAGATTGAAGGTCAGG

GGCGCATTGTCTATATCTGTATTCCTTATTCCACAATCGAACCGATCAAGGAAAAGCTCTATACCGGTTTCCAGAGCGAC

CAGTTCGAAGTCGACAGCCTGTGGACGACCAGGCTTAAGGACCGTATCGAGGCGAGTCCGATCAGGATCCAGGCCATTCT

GGGGAAAACGAGCAGAACAGTTCGGGAGGTTCTGGACTGGAAAGTGGGCGACGTGATTGCTCTGGAGCGCCATATCAGCG

ACCCGATCGATATTCATGTGGAAGGAATCAGGAAATTCCTGGCCCGTCCGGGAACCCACAGGGGAAACAGGGCCATCCGT

ATCGAAAAACGGGTGCCGCCTCCAACGATTTATCAGGAGGGGGATGGCGAAGAAGGGCTCAGTCTCCCGCCGGTA

>fliN LfeRT32a_1827 Flagellar basal body, switch, biosynthesis protein

ATGTCTGATGAAGAGATCGATCAGGAAGCCCTTGCAGCCGCATGGGAGGCTGATCTGACCAAAGATGACGCTCCGGAGCC

GGAAGTGAGCCGCGTCGATGCGGGCAAGACAAATGAAAGTCCCGAAAATATTGATTTTCTTCTGGACGTTGCCCTGACGA

TTTCTGTCCAGGTGGGAACTGCAAAAATGTTGATCAAGGATCTTCTTCAACTGGGGCAGGGTTCGGTTGTTGAACTTGAG

AAGCTTGCCGGCGAGCCGATGGAGATCCTGGTCAACGACAAGCTGATTGCACGTGGAGAAGTTGTTATGGTCAACGACAA

GTATGGTGTCCGGCTCACTGATATTGTCAGTCCGGTCGAGAGAGTGAGAAGCCTG

>fliO LfeRT32a_1826 Flagellar basal body, switch, biosynthesis protein

ATGATCAGTCATGCCCATGTCCACGGTCCATCCATGATGATGATGGGAGTCCGGCTTTTTGCCTCCTTCTTTTTTGTTCT

TGTCCTTTTTTTGGGTGCTGTTGCCCTGATCCGGTATCTCCAGCGCCGGGCCCCTTCCCTCAGAAGGGAAAACAGGGACT

CGATCGAGATCCTGACAAGTTGCACGCTGGCGCCAAAGACAACCCTTTCGGTGGTTTCGGTCAAGGGGGAACAGTTTCTG

ATCGGTGTGACGCCAAATTCTGTCAACCTGCTTTCAAGGCTTGGTGCCGGGGAGTCTTTCCAGGGTGTGTCGCCTGTTGC

ATCTCCGGCAGAGAAGAAATCTCCCGCCGAGCGAAGCGATCCCACGGATTTTCCAAAAGGGGACCCAAAGGGATTTGATC

ATCTGCTCAAGGAAACAGTAGGTCGTCTGAAGGATGTCCGGGAACCCCGGGAACAGTCTTCCAACGGTCGAAGGTGGAGT

GTT

>fliP LfeRT32a_1825 Flagellar basal body, switch, biosynthesis protein

ATGGGGTTTCCTGCCCTGCTCAGGCGCAGTGCTGCCATTTTAGGTGTTCTGTCTCTCCTCTGGATCTGTGTTCCGGAGTC

CAATGTGGCATGGGGGAGCAACCTGTCGGTCGCTCCTGTCGCCCAGCTTCCCGAGCTTTCCCTGAAATTCGGGAGCGACA

ACGGCCAGCCAAGCCAGGTGGCAATGACTGTCGAGCTTCTTTTGATGCTCACCGTCCTTTCTCTTGCGCCTTCGATCCTG

ATCATGATGACTTCCTTCACGAGGATCATCGTTGTCCTTTCGTTTCTGCGGCAGGCGATGGGCACCGCCCAGGTTCCCCC

GAACCAGGTGATGATCGGGCTTTCTCTTTTCCTGACCTTCTTCATTATGGCTCCTGTCTATAAAACGATGGACGAGAAAG

CCATCACCCCTTACCTTGAAAAGCGGATGGCTCCGGAGGTCGCCATGACAGAGGCGATGAAACCGGTCAGAAGCTTCATG

ATGAGGCAGGTGAGGAAAAAGGATCTGGCTCTCTTCATGAAAATTGGCCATCTTCCCGAATCTGTCAGCAATCCGGATGA

TATCCCGACTTATGTCCTGATCCCTGCCTTTGTGATTTCCGAGCTGTCGACCGCCTTTGAGATGGGGTTCATGATCTACC

TTCCGTTTCTGATCCTTGATATGGTGGTTTCCTCTGTCCTGATGTCGATGGGCATGATGATGCTTCCTCCGACGGTTGTC

TCCATGCCATTTAAAATTCTCCTGTTTGTTCTGGTCGATGGCTGGTCTCTTGTTGTCGGATCCCTTGTCCAGAGCTTTCA

T

>sirA-like LfeRT32a_2547 Response regulator with a BarA-like signal transduction His kinase

ATGGGTGGTGAATCCTTAAATCCAGATGAAGTAATCGATGCAAGAGGGCTTTATTGCCCTGGTCCGCTTATGGAGTTGAT

CCGGGTTATTTCGGTCAAGCCTGTCGGTTCAGTCCTGAAAATACTATCTTCCGACGAGGGTTCAGCCAAGGATATTCCTG

CCTGGATTGCGAAGGTTGGACAACAGTATATTGGTACGGAAAAAATGGACGGCTACTGGGAGCTTTTGGTCAAGAAAGTC

AAG

>rpfG LfeRT32a_3506 Response regulator RpfG

ATGTTATTTGAAAAGCTACGGTCTGACTTGTTCGGGATAAAGGAAGAAGGCGTCAATATGGAAGAGTTAGTGCGTTATCG

ATCATTTGACCATTTTCTTGAGTCTGCAAGGGTCATGATCATCGATGACCAGTTTGTCGGTCGCAGGGTGCTGGGGGAGA

TTGTCAGATCCATTTCCCAACGGATCACGATTGAAAGCTTTTCTGATCCGGAGGAGGCGCTGGCCTCTTTTTCAGGCCCT

TTGCCGGATCTGATCCTTTTGGACTACAAGATGCCGAAAATGAATGGAATCGACCTGATCCGGGCCTTTCGGGAGAAACC

CTCCGGAGAGGATATTCCGATCATTATGGTGACGATCCTGGAAGATAAAAATATCCGCTATCAGGCACTTGACGCGGGGG

CGACGGATTTTCTCACCCGTCCGCTGGACCAGATCGAATGCCAGTGCCGTTGCCGAAACCTGTTGTCGCTCCGTAAAATG

ACCCTTGAGATGCAAACCCATTCCCGGTTTCTGGAAAGGGAAGTGGCCGAAGCGACCCACCTTCAGCGACAGCGTGAAAG

GGAAACGCTTCTGAGGCTTGCCAGGGCGGGTGAGTTTCATGATTTTGAAACAGGAAACCATGTCGTCAGGATGGCCAGGT

ATGCCAAACTGATCGCCCAGGCCATGGGACTTCCCGAGGACCATTGCGAAACGATCGAACTGTCGGCGCCGATGCATGAC

ATCGGCAAGATCGGAATCCCGGACCATATTCTCAAAAAGCCGGGGGCACTCACTGCCGAGGAATACGAGGTCATCAAGAT

GCATCCGATGATCGGTCACCAGATTTTAAAGGAGAGCAGCTCCAGGTATATCGAGATGGGTGCAGTCATCGCCCTCGCCC

ATCAGGAGCGTTATGACGGTTCGGGCTATCCCTTTGGACTTTTGGGAAAAGAGATCCCTCTTGAGGCGAGGATTGTGGCC

GTTGCGGATGTTTTCGACGCCCTGACCTCTGTTCGTCCCTATAAGGACGCATGGCCATTGGACAGGGCCGTTGCCTACCT

GCGGGAATATTCGGGAATACTGTTCGATCCGGAATGTGTCGAGGCCTTTTTCCGTCAGACAGATGAGATCAGACGGATCT

CGATTGTCATGGGAGATACCGAAGATCCGATGAAA

>rpfC LfeRT32a_3505 Sensory/regulatory protein RpfC

ATGAATTGGCTTGAAGGTCTCCGATCGAGACTTCGAGAAAACAATAGCCAGGAACATGAACAGGCTCTTATTCGTCTTGC

CATCGGAGTGCTGGGATCCCTGTATCTTCTGGCGCTGCCTTCAGGCAGGGACCGGGCGGGACAGCTTCTCATGTCCTACC

ACCGGTATTCCATTTTTTTCTTCCTGTCCATTTCCGTCCTGATCCTTGTTTCGATCTTTCTCCGACCGAGGCCTTCATAT

CCGAGGAAGGTCCTGGGCATTTGCCTTGATCTGTCTACCGCATCCTTTGTCATGGCCACGAGCGGGGAGCAGGGTCTTCC

CATGGGGGTTGTCTACCTGTGGGTGATCATGGGAAACGGTTTTCGCTATGGACTGCGCTATCTCTATCTGGCGACGTCCA

TAGCCCTTGTTGGGCTCCTGTGTGTGTTTTTCTGGAGTCCGTTCTGGGAGAGCCATCCGACTCTTTTCTGGAGCCAGCTG

CTTGCGATTGGTGTCCTGCCTCTTTACATGGCCGTCCTTCTCAAAAAACAGAAGAGGCTCATTGATCTTGCAAACGAAGC

CAATCGGGCAAAATCACGTTTTCTGGCCAATATGAGCCATGAGCTCAGAACGCCCCTGAACGGGATTATCGGAATCGGCG

AGATTCTGTTGTCCGAAGCCCCGACCGAACGCCAGAAGGGTCTTCTTGAGGCCATCAAAACCTCTTCCGGGATTCTTGTT

GAAATGATCGAAAAAATCCTCGATATTTCGAAGATTGAAGCCGGCCGGATGACCTCCGAGTCCAGAGCGTTCAAGCTTGC

GGAACTTGTGTATCAGGCTGTTTCCTCGATCGAGCCCATGGCGGCGAAGAAAGGGCTTTCCGTCAAGCTGCTCTGGGATG

CCCGGCTTCCGATTTCCGTTAAAGGGGATTTGTCCCATCTGAGACAGATCCTCGTCAATCTTCTGGGAAACGCGGTCAAG

TTCACTCTGGAGGGGGAGGTCTCCCTTTCGATCCGTCTGGCTTTTGCCGATACTTCGGGCCCGCGTGTCCGATTTGAGGT

TGCCGATACGGGAATAGGAATCTCAGAGTCGATGAAGCCCAGGATCTTTGAGCGTTTCATGCAGGGTGATGAATCGGTTA

CAAAACGCTTTGGCGGGACGGGGCTGGGCCTGTCCTATGCCCGCCAGCTTGTGGAGCTGTTGAACGGAAATATCGGTTTT

TCCAGCAGGGAAGGTGCCGGGTCGGTTTTTTGGGTTGAAATTCCTTTTGGGGATGCGGAGAACGCTCTCTCCGGCGAACC

GACCATTCCGGCGATTTTCTTCTGGGGACCGGAGCAGGACTTCGGACGCTACGAAGAGCTTCTTGCCCCCTCTACCGGAG

ACCTGCGATGTGTTCCCTCTTCTGCGACGGTCCTTCCTTTCGGGCCGGGTGAGCTCCGGGGGGTGCTGGTGGCAAAGATT

GACGAATCGAACCAGCCGGCCTTTTTTGAGATGCTTGAGCATCCTGGAAGCAGGAGTGTCCTTTCAGGGATGCTCAAAGT

GCTGCTTGTTCCTGAAGATTTTTCCCCGCCCGACCCACGGGATCTGCCTGTTGACGGCTCCTATGTGCTTGCAGGTCCGC

TTCCGGCGCTTGTCCAGAGATCATTGGCATTCTGGCCGTTTCAGAATCGTCCTTCGGGCGCTTTTGCCGATCCTCCCGCC

ATTCCGGCAATCGCTCCGGCGGGCCGTTCCTTGCGGATCCTTGTGGCAGAGGACAATGCCGTCAACCAGAAGGTGGTCGA

GGAGATCCTGTCCGCAGCGGGCCATCAGGTCCGGGTCGTTTCCGATGGAGAGATGGCTCTTGACTGTCTCGAGTCGGAAA

CGTTCGACCTGATGATCCTTGATCTCTGCATGCCGGTTATGGGAGGGCTTGATGTCCTGAAGACGCACCGGTTCATGGAG

AGAAAGTCTCCTGTTCCGGCGATTATCCTGTCGGCAAACGCCACAAAAGAGGCGGCCGATTCAAGTCATGAAGCCAAGGC

GCAGGCCTTCTTGACGAAACCGATCCAGATTCCAAGGCTCCTGGCGGAGATCGACCGGATCACCTCCCTGAGAAAGGCGG

CCGATTCTTCTTTCCGGAATACGGATCTGCCCGCAAGGGAACTGCCTCTTCTTGATGCGGAAGTTCTCAGGGAGCTCAAG

AAGGTCAGCCCTGATCCAGCCTTCATCAGGACCCTCCTTGAGGGTTTTCTGATGGATGGGGAGAGGCTGCTTTCCCAGAT

GGAGGATGCTTTGGTCCGTTGCGATTTTCCGGAGTTCATGGATGCTGTTCACGGACTGAAGGGAAGCGGTGTCCAGATAG

GCGCCCAGAAGCTGGTCAGTTTTCTTGCGGAATCCCAGTCGATGGAGATTTCCCTGATCCTGTCTGGCCGGGCAACATTT

TTCATGGATGGGATCAGGGACCTTTTCTTTTTGACCGCATCGGAAATCAGGAGGTTTGTCGATGGGGGGGCCCATTTGTT

GACCGATTCCGGGAAAACAAGAGATTCATTCACCAGACGC

>kdpD LfeRT32a_0804 Osmosensitive potassium His kinase KdpD

TTGAACGAAAAGCTTGAGGATTTTTCAAGATTCGATCCGTTTTTGGGAACAATTCTCAAAATGGGAAAGGGCCGCCTTAA

GATTTACCTTGGATGGGCTCCCGGAACAGGGAAAACAAGGCGGGCCCTCCTGGATCTTCGGGCGCTCAAAGACCGCGGGG

TTGATGTCGTTATAGGCTGGAGCGAGGAGAATCTGCGCTCCGACGTCGTGAAAATGCTGGAGATGTTTGAAACTATCTCC

CCGGTCAAAATGAATGTCGGATCGGATTGGTTTCCCGAGATGGATCTGGATGCCATTCTCGGGAGACATCCAGCCACCGT

TTTTGTCGATGAGCTGGCCCACGGGAATGTGTCGGGATCAAGACACCAGAAACGCTGGGAGGACATCGAAGAGATTCTTG

AAGCGGGAATCAGCGTTGTGACCACGCTGAACTCAATGCATGTCCAGGATCTTTCTGAAGCGGCCGAGGGGATTCTCGGA

TTTCCTGTCAAGGAAATCGTTCCACTGGATTTTATCAAGCGGGCCGATGAGGTTGTCGTGGTCGATCTTCCTCCATCGGA

GCTTATACTCCGGATCAAGCAGGGGAACGTCTTTCCTCAGGAGCAGATTGAAAGGGCCCTGAAAGGCCCTTTCAGGGAAA

GCAATCTGGTCAAGCTCCGGGAAATGACTCTGGCCTTCATGGCAAGAGTCCTTGATGTCCAGCTGACGCGACAGGGAGGG

AAAAAAGGTGTCTACGAGAGAATCACGGTTTTGGTTTCAGAGCATACGCCATCCCTTTCAAGGCTTTTGGGTTATGCCGG

AAATCTCTCAAGACGGATGGGGGGGGAGCTTCTTGTCCTTCACCTCAGGACGATTTCTCTTTTTGGAACCCGGTCACCAC

TCGATCCCGAGGTTCTTGAGATGTTCCATCAGGAGGCCCGGAAGGCTGGAGGCAAGTTTTCCATTCTCTGGACCAGGAAT

CCGGGGTGGACATTGTGGCGGTTTATCCAGAGGACAAAAACGACCAGACTGGTCATGGGCCATGCAGGAACACAACGTCC

ATGGAGAAAGTCCCTGGTCAGGTCTGTTCTCAGATATTTTTCCAGGATCGATGTCGAGATCCATCTTATTCCGACGCTTG

GGGAGCTTCAGCAAAGAGATCCTGTGGAGGAGCCGCCCTCCAGCCCTCTTTCCTCTTCAGGATCCAGGGGACGCCTGACC

CTGTTTCTTGGGGCAGCTGCCGGTATCGGGAAAACTTACAGGATGCTGCAGGTGGCGCATGAACGTCAGGAAAACGGGAC

GGATGTGGTTGTCGGCTATCTTGAAACGCACCGCCGGCAGGAAACCGAGCAGATGTCGGAAGGTCTTAGGACGATCCCCA

GAAAAATGGTTTCCTATCATGGACTGGTTCTTGGCGAGATGGATATGGATGCAATCCTTGCCATGAAACCGCGCCTTGTT

CTTGTTGATGAGCTGGCCCACAGCAATCCCCCGGAGTTCCGGAACAAAAAACGTTATGAGGATGTTATCCGGCTCCTTGA

GGAAGGGATCGATGTTTTTTCCACACTGAATGTCCAGCATCTTGAGAGCCTCAATGACCTCATCGAGTTCCAGACGGGGA

TACGCGTTCATGAAACTGTGCCGGACAGCATCGTTCTGATGGCAGACGAGATTGTTCTGGTTGACCTGACAGCCGAAGCG

CTTCAGGCACGCCTGATGGAAGGCAAGGTCTATCCTCTCCAGAAGGTGGAGGAAGCCCTCAGGAATTTCTTTACAAAAAA

TAATCTCACGGCCTTGCGAGAGTTGGCAATGAAGTGTGTTTCAGAGGGTTCTCGTGGCCGGGTGATCCAGCGGGGCAGGG

GAGGATGCGTTCTTGTCGGGGTTTCTGACCGGCCGGAAGATGCAGCGCTTGTTCGAAGGGGAGCAGTCCTTTCAGATCGC

CTGAGTCTTGAACTGAAGATTCTCTACGTTCGAAAAGAGGCTGACGAAGGGCTTCACTCCCAGGTGCTTGTCGATCTGAC

ACGTTCGTTTGGCGGGGTTTTCCTGTCGGAAGTTTCTCCACAGAAATGGGAAAGTCACTTCATCAGGCGATGTCAGGAAA

TCGGCCCAAGTCTTGTTTTGCTGGGGCAATCGGCATGGAGGCCAGGATTTGAGTCGACAGCCGAAATGATCGCCAGAAAC

CTGAACCAGTTTCCTCTTTTGATTATTCCCCTGGATATAAGGGAGCATGTCACGGAAAAC

>pilS LfeRT32a_3026 Putative PilS/PilR two-component system. Upstream of flgBCfliEFG

ATGAAAGACAGTCTGTCTGAGCACAGGGAAAGAATGGCCCATTCCGGGGAGGATGCCCTTGCCCTGGCATTTTCTACTTT

TCAGGAAGCATCGGAGTCGCTGTCCAGAAAATACGCCGGTCTCGAGGGGAAAATTTCAGAGCTGTCAAGAGAGCTTCGCG

ACCGTGACCGGGCGCTTGCCAGCCAGGGACAGTTTCTTGAAACGATCTTGAAGAGTCTTCCTTCGGGGGTATTGGTCCTG

ACTCCTGGAGGCCAGGTTTTATGGAGCAATCCGCAAGTGGAGAAATGGCTGTCTCCGGATGATTCAGAACTTCTGGCCCT

TCTCCGGAAGTGGGAAATATGGCCGATTGCCGAACGGGAAGATCCATTGGCCGTTTCCTGGAAAGGTCGCTCTCTTTTGA

TTGAGATTTCAAGGGTTGTCGATGATGAGAATCGCTCTTCAGGCTATGTTCTGATCGTGAACGATGTCACAAGACTTCGG

GAGATGGAGGAAGAGGTTTCGCGGGACAGACGACTTCGGGATATGGGAGAGATGGTTGCGATGATTGCCCATGAACTCCG

GAATCCCCTTGGAAGCATAGAGCTTTTTTCCAGTCTGCTGGCCCGGGATGCGGGCACCAGGGGAGGTCAGGCACTGGAAG

GGATCAGGTCCTCGGTTACATCAATGGATCGACTGATTGGAAATCTTCTTTTTCACACCCGCATTCCGAATGTTTCCCGC

TCTCTTTTTTCCGGGCAGGATCTTCTGGAGCGCCTGTCTTCCGATTGTGCCCGTATCGGTATCATGCGGGGGAGGGCCGG

AGGGCCCACGGTTTCTTTTGCGGTTGACGAGCCGTCTCCCTTTCTGGTTAACGGTGATGAGGGGCTTATCTATCATGCAA

TCTTCAACCTCGTCACGAATGCGTTTCAGGCATGCTTCGATGCGGGAGGCGGAAGTGTCACGCTGTCTCTTCTCCGCAGC

AGGTCAGGGGGAGCCATTTTTTTCGTAAAGGACAATGGTCCGGGAATTCCGCGGGAGATGAGGGAGAAGATCTTTGACCC

GTTTTTTACCACCAGGTCCAAAGGGACGGGGCTCGGTCTTCCGATTGTCCTGAAGATTGTGCTTGCCCACGGGGGATTGC

TGAAGGTCGATTCAGACGAAAATGGGAGCTGTTTCATGGTTCAGCTGCCCGGTCCGGGCGAGAGTCTGGAACGGACGGGC

GGAGACATCTGGTCAGGACAACTTCTGGAGGAAGAATTGAGA

>pilR-like LfeRT32a_3027 Putative PilS/PilR two-component system. Upstream of flgBCfliEFG

ATGAATGCACAAGGCCCTGTTCTGGTGGTCGATGATGAGTCCGAAATGTCGCTTGCTCTCCAGGAAACATTGCGGGAGGA

CGGATATATGGTTGATCTTGCCTCGAACGGAAAGGAGGCTCTCCGGCGTTTTGAGGATATGGGCCCATACCAGTGGGTCA

TCACGGATCTTAAAATGCCCCAGATGGATGGATGGGCTCTTTTGACCACGCTTCGCCGCATCAGTCCCGAAACCCGCGTC

ATTTTGATGACGGCCTTTGGTTCTGTCCCCCAGGCGGTTGATGCCATGAGACAGGGGGCGGTTGATTTTCTGATGAAACC

TTTTTCTCCGGAGGCTCTCCGTCGTCTTTTATCACCTGATGGCGCGCTCCTGACCCGCCAGAAGGGGGCTTCGGGGGAGG

GAGAAGGGATCTGGCAAAAGCTCAGACGTCCGATCCTGACCTCAGACGCCCGATTTTTGAGACTTCTCAAAATGGTCGAG

TCGGTTGCCAGGACCCAGGCGACCGTTTTGATCGAGGGGGAGAGCGGGACCGGAAAAGAGCTGTTGGCCCGATTTGTTCA

CGAGGCTTCTCCAAGGGCCCATCGCCCCTTTGTCGCGGTCAATTGTGCTGCCATCCCGGAGGGTCTTCTGGAGCCTGAGC

TTTTTGGTTATGAAAAAGGAGCCTTTTCCGGTGCCGTCTCGAGAAAAATAGGAAAGTTCGAGCTGGCGGATACCGGAACC

ATTCTTCTCGACGAAATAGGGGAGATGGAGCTTTCTCTCCAGGCCAAGCTTCTGAGAGTGCTCCAGGAAAGAGAGGTTGA

TCGTGTCGGGGGGAGTTCTCCCGTGCCGGTCGACCTCCGGATTGTCGCCACGACAAATCGGAACATGAAGGAGCTGGTGG

CATCAGGAAAGTTCAGGGAAGACCTTTATTACCGGTTGAGGGTGTTCCCCGTGCAGGTGCCTGCCCTGAGGGAGAGGAAG

GGAGATATTCCTCTCCTGTCCCGTCACATTCTCGGGAGGTTGCGCGAGGATGGAATACCTGTCGGAAATTTGACGCAGGG

GGCGATGGAAGCGATTTCCCGCGGAGAGTTTCCGGGCAATATCCGGGAGCTTGAAAATCTCCTGACGCAGCTGGCACTCC

TTTCGGGAGGAGATGACATTCGGATGGAGCACCTTGCGATGGGGGAAGGCGGCTCCCTCCCTCCCGAGGACCATTTATCC

TGTGAATCGGCAAAGGAGCGTGAGGTTCAGTTCCAGGACAGCTTTTCCTGTCGTCCAGGCAAGTCGGTCCGGGAGGTTGA

GCGTGACTTGATCCTGATGACTCTTGAGTCCTGTTCGGGGAATCGGACCCAGGCGGCCCGGATGCTTGAGATCTCGGTGA

GGACCTTGCGGAATAAATTGCATGAGTATGGTGTCTCTGTTTCCGGGGACTCCGAAGGAAACCAGGAA

>orf2505 LfeRT32a_2505 GGDEF domain protein. Putative Diguanylate cyclase

TTGATCTCTGACAACGTTCAATCCTTATTTCATTTCGAGCCGGAAGAAGACAGGGATTTTGTCCGTGACAAGATCCAATT

CCTCTATATTGGCGGATCGATCGGGATTGCCTCATCCATCACCATGACAATCATCGAGATTACCCGGAATGAGTTGTTCC

GGGTAATCCCGTCTTTACTTTTTTCCATCATTGCTCCCGTACTTCTCTTCTCTCTGTCCCGCAAAACGGAAAACTATCAA

AAGATCCTCACGGGATTTGCCGGCCTGATTCTCTGTCAGCAGATTCTGGGGGCCTTTATCTCTTTCAATGAAGTCCTGAT

GATTGTATGGTATCCGGTCTTCCCCCTGACCTATTTCTTCCTTCTGGGATATCAGAGGGCACTCCTCTGGAATGCCGCGG

CAATCGTGGGGATCGTTTCCGGATATTTCTGCTTTCCTTTTTTCAACCACATCCCCCCGGTTTCCTTCCCCATCTTCCTG

AGCTCCGTCTTTGCCTATTGTGTTGCAATGCTTCTGGCCTGGTACCACTACCGGGTCATCCATACCTACCAGAGCCGGCT

TAAAAGAGAGGCGCTCATCGACGGTCTGACCGGAGCCCTTGTGCGAAAGGCCGGCCTCGGAGAATTATCCAGACTGATGG

CCCAGAACGACCGCGATCAGAATAGGGAATTGTTTGTCGCACTGATGGACATCGACAACTTTAAGATCATCAACGATCAG

GATGGTCATCAGAGCGGAGACCAGGTTCTTGTTCTTGTGGCAGAAGCTGTCCATCGAACCATCCGGAAAGACGATCTCTT

TGTGCGACTGGGAGGCGAGGAGTTTCTCCTCCTGCTTCCGGGACAATCCTTCGATACCGCATACCTGATCTCTGAAGATC

TTCGCCAACGGATCGAGCAAGAGGTCATCCGCCCCGACGGATCGGCGGTGACCGTCAGTATCGGTCTGACACGATATCGG

CCGAAAGAGACGCTCGGAAGCCTTCTTCACAGGGCAGACAATCTGATGTATCGCGCCAAGAAGTTCGGCAAAAACAGGAT

CTGCTGGCAGGAGCACGGGCGTGCCGACGTGTTTTTACCCGCACCTTCACAAATAACCGTCCCCGAAATTCCC

>orf2506 LfeRT32a_2506 GGDEF/EAL domains protein. Putative Diguanylate cyclase/Phosphodiesterase

TTGATCCTCGCCGGTTGGCCGGTCCGCTGGATGAAAGGGGGGCTTTTGTCTCCGTCTTCAGGGCAGCAGAAACACATGAA

GGAAGGGTTATGGTTTTCGAGCTTATGGCTTATGTGCTTCCTTGCGCTTGCCGGAGCCATTTTCCTCGCCCTTTCCCTGG

ATCGTGCCAGCACGAACAGAAAACTGCTTGAGACAACCCGCTTCACCGATGCAGTCAGTTTCTTCGAAAAGATCAACGAC

ATTCTTGTCCTGAACTCCATCCCTTCCGCCAATCTCTCACCGATCAAATGGGAGACCTATCGCAACGGCCTTTTTGTTCA

GGGAAAGACAGTCCTCAGGGTCATGAAAGGCAACGACACTTTTCTGACAAAAGAAGAGCGGACCCGGCTTCAGGAAATGT

GCACCCTTTTTAAAAAACTTAAAAAGGGTCTTCTGGCAGGCAGGCCCCTTCGGGAGCTTGTAGGAATCGACCACCGGATG

ATGCTTCTGTCCATCGAGCTTGCGGGCTGGTCTGCCAGCAAGCGGGAGAGCTTTTTTTACCGGATTCGCCTTCTGGACTA

TGCCCGGGCGATTCTCCTTCTTTCTGCCCTGGCAGGAGGAGGGGTCTTCCTCTTCGGAAGAACCCGGCTCCATCGGAAAA

CCTCAAGGCAGAACCGGTTCTACCAGGCCCTTTTGCGAATCGACCAGCTGATCCTGACACTCCCCACCATGGAGGAATTA

CTCCCGCAAACATGCCGGATCATTGTCGAAGAAAGCGGTGTCATGCTCGTCCGGTTTATCGAACTCGACGCCGCGTCGGG

AGAGGGGTCCATCCTGGCTTATTTTGGAAGAGCAACAGAAGAGTTTGCCCGGAGAAAGCATTCCCCCGACCCCTCCGCGC

CCGGAGGAAGTGAGCTATGGGCAGATCTTGTCAGACTCAAAGAGCCTCTCGTATGGAACAGCCTCCAGAGTCAGCTCAGG

GATGGTTCCCTCCGGAATGTCCTTCTTGAAAATGGAATTTTTTCGGGAGCAGGCATCCCGGTTTTCCGGGAAGGGACTAT

GTTCGGCGCATTGATTCTTTATTCGGATGAAGAGAATTTTTTCGATCCCGACCTGATGAACCTGATTGGAATGATGGTCA

AGAATATCTCGTTCGCGATCGAAAATCGGGATCGGGAGGACGACCGGAAAAAGCGGGAGGAAGAGGTCACCCGACTTGCC

CTGTTCGACAATCTGACCGGTCTTCCGAACCGCCGACTTTTTCATGACAGGATGCAGCAGGCGGTAGAACGTCATCTCCG

GACAAAAGAGCGTTTTGGAGTCGGCATCCTGGACCTCGATGGCTTCAAGCAGGTCAATGACCGACTGGGCCACCCGGCTG

GAGACGCTCTTCTGGTACAGGTCTCCGAGCGGATCAAGGGTATTCTCCGGGGAACGGATACGCTCGCCCGCCTCGGCGGA

GATGAATTTGGAATCATCTTTACCCATCTGGAAGCAGAAAAAGGATCGGCTCTCTTCGACCGGGTCATCCGTTCTCTTGC

CGTGCCGTTCACGCTGGGAGAAGAGGATGTGACAATCGGGGGAAGCCTGGGGATCACCATCATCCCTCCCGACGATGGGA

GGGATGAGAGTCTGCTCAAGCATGCAGACCTTGCCATGTACCAGGTCAAGGAACAGGGCAAGAACGGATGGGAGATCTTT

CAGCCGGCCATGACCGAAGCGCTGGAAAACAGCTACCGGCTGAAGAAAGAGCTCGAAAAGTCCCTTGGGGAAAACCGCTT

CGCTCTTCATTATCAGCCCCAGGTCGAATTGGCCTCGGGAAGGCTGGTGGGCGTCGAGGCTCTCCTTCGCTGGAACCATC

CCGAACGGGGGCTGCTGGACTCGGAATCTTTCATCGGGATCCTTGCAGGGTGCGATCTGGCCATCGACATCGAAAGATGG

GCTCTGGAAGAGATCCTTTCCCAGATAGAGGATTGGAGCGCACAAGGGATCCGGCCCAGAGTCAAGATGAATATCGGAAG

CCGCCACTTGCTCTCGGGAAGATTCACGGATGATCTCAGAAATGCCTTGTCCCGGCATCCGGAGGTTCCTCCCCAGGCGC

TTGAACTGGATATCACGGAAACAAAATCATTTCCTGAAATCAGTAAGGTGAAAGAAATCTTCGATGCATGCCGACGCTTG

GGGGTTTCGATCAGCGTTGCCAACATCGGGACAGAACATGGATCCATTGCCTACATCCAGACACTCGGAGTCGACCGGGT

GACGATCGATCGGCGATTTGTCCGCGACCTTCCCAAAAGCCCGCAGGATATGGCCATCGTGGCAAGTCTTGTCACGTCGG

CCAAGTTGCTGCTGGTCGATGTGGTCGGAGAGGGGATCGAAACAGAAGAGGAGGGGGATCTCCTGCTCAGATGGGGATGC

CGCATCGGACAGGGATTCGGGATTGCCCATCCGATGGTTCCCGAAGAGATCCCTGGATGGGCCGAACGATATCGTCCGTT

TGAGTCCTGGTCCCATTGGGCAGAGGTTCCCTGGGACCCCCAGAACTATCCTTTGCTTCTGGCCAAGGAAGCGGCCCGTG

TTTTTTACGAGAATTTCCTGGATGGAATCGCAAAACCAGGCGTCAGCCGTGTCGAGTGGACCGACTCTCACCGATGCCTT

CAGGGAAGGTGGATCGATGGAGACGGTCAGCTCCGCTACGGGGCGACACTCGAGTTCCGACAGTACAGGGATGCCCACGA

GCATCTCCACTCGCTGATCCGGGAGGCTATCCTGTCCCGGGATACGGGAGATTTTCCACGACTTGACACGCTGAAAGAGG

CCATCCGGGAGGTCAATGCCGAACTGATCCGGCGGATCGATCAGATCCGCTCGCTGGGCGCTGCCGGACCGGGATCG

>orf3403 LfeRT32a_3403 Putative diguanylate cyclase/phosphodiesterase with GAF sensor

ATGACAGGGATAGGTGTCGTTTCCATGGCGAAAGAAGACAAAGAACCACCAGATCCACTATTCCGTTTTCGCTCTGAAAT

CCTGGGAAAGGGAGATCTCCTCCTGAATCTTCCTTTCGAAGGCATTTATGTGCTGGACGAGAAGGGATTTCTGGTTGATA

TGAGCGATACCTTCTGCAGCAGTCTTGGTTATTCGCGGGAAGAAATGAACGGGATGCATGTCTCCGGATGGAATGCCGTC

TGGGACGATCCGGTGGTGAGTGCGCAATTTGAAAGTCTTGTCCAGAACCCCCACCGGCGGACCATCACTTTCGAGACGGT

CCACCGGACAAAATCCGGCCGCTTGATCCCCGTAGAAATCCGGGCCTCTTCGATCCGGCTTGACATGGGGGTTTTTGTCT

ATTGCTCCTCCCGGGACAAAACCGCCGAAATCAAGGCCCGCCATCTCTCAGAGATCAATGACCTGATGCTGAGAGTCAAT

CAGGAGATCTCAACCGTCGAAAATGAGGGAGTTCTCCTCTCGAGGATCTGCGAAATGTCGGTGGAGTTTGGCCACCTGTC

TCTTGCATGGGTTGGCCGTCCTGACGATTCGGGCAAGTTCCTCTTCCTGGCATCTTCCGGAGACACGGAGTATCTCGATG

GGATCGAGATCTCTTCAGACCCCGGTCTTCTTTTCGGAAATGGTCCGGCGGGGAGGGCTTTCCGGGAAGACCGGGCCATT

TTCTTTGGTAACTTCGAGTCGGATAGGCGACTGGGCCCATGGAGGGAGCGGGCTTCACTGTTTCATTTTCAGACAACAGC

TTCTCTTCCGATCCATCGGGGAGGGCAGGTCTGGGGCGTTCTGGTCGTCTACCATAAGGATATGGACTTTCTCAACCCGG

AGATGAAAACGCTCCTGGAAGAGCTTGCCAGGGATATCTCCCGTGGTCTTGACCGGCTTGATGCCAGGGTCAGGGAGAGA

GAGCTGTTTGCTGTCCACCAGGCACTTCTGGACAATACCGTCGTGGGAATTGCCATGACCCAGGGGCGGAGGTTGTCTTT

TGTCAACTTCCGCCTCCTTGAGCTTCTGGGCTATGACAACCCGGAAGAGCTTCTTGGTCAATCCACGCATGTTCTGTATC

CGGATGAGGAAGAGTATTTTCGAATCGGTACAGTCCACAGTGAAATCGATAAAAAAGGAACCGTCGGCATCACCGGTGTC

CGGCTTGTGAAAAAGGACGGGACGATTCTTTTGTGCGATGTGTCGGCAAGTCTCGTAAGCATGACCCCGGAAAAAAAAGC

GGTATGGACCATCGAGGATGTCACCGAAAAACACCACCGCACGGAAAAGATCAAGCTGCTGTTGGGCCTCAACAAGATGC

TCGCACAGGTCAACATGGCGGTTGCAGAAGCCCAGGAGGAGCTCAGCCTGATCCGGTCGATCTGTGACCTTGTGGTTCAG

CTGGGGCACATGGAGCTTGCCTGGATCGGTCGCGCCGATGATTCGGGTGCGTTCTCATTCCTGGCCTCATCGGGGGAATC

CGGATATCTTGAAAAAATGGCCGTATCTCCGGATGATCCATGTGGTCTGGGCCCTGCAGGACAAGCTCTTAGGGAAGGCA

GGGCTGTTTTTAGTGGTTTGCGCAAGGAATCTCATTCCCCGTGGGTTGAGGAAGCCCGGAATTTTGGAATGAATTCGGCT

GCGGCCCTTTCCATTTTCCGAAAAGGGAAAATCTGGGGATTTCTCGCTGTCTATCACCGGGCAGAGGGTTTTTTTGACGA

AGACGATCTGAAGTCCTTGTTTCTTGAGCTGGCGCTTGATATTTCCCGGGGGCTTGACCGGATCGATGACCAGAAATCCC

GATGGCTTCTCTCGAATGCCCTCTCCTCCATCGAGGACGGGGTGGCGATTACCGATTCTTCCCATCTGGTGACATGGGTC

AACAAGGCGTTTACCACGGTCACGGGGTATGCACCGGATGAAATTGCGGGAAAGAATCTGAAAATTCTCCAGTCTCCGGA

GACGGATTCCGAAACGGTGGAAGACATTCGACAGTCATTAAAGAACGGAACGGTTTTTCACGGCCAGATCCTCAATCTCC

AGAAAGACGGGTCAAAATTCTGGAACCTTCTCACAATCAATCCGATGCACGATGCGTCCGGCACCATCACCGGTTTCGTC

GGGGTTCAGCGGGACATCTCGGATATGGTTGCACTAAAGGAGCAGCTCGAATTCCAGTCTCTCCACGATCCTTTGACCGG

ATTGCCCAACCGGAGGGCGCTTGATCAGCATCTGGCGATGGCAATGGCCAGATCCCGCCGAAATGGAACGGTCGTCGCGC

TGGGACTCCTGGATCTTGATGATTTCAAGGCAGTCAATGACCGTTTTGGACATTCTGCCGGAGACAGGCTTCTCATCGAT

CTGGTGGCGAGACTTGAGGCAAGGCTCAGGGAAAATGATTTTCTTGTGCGTCTTGGCGGAGACGAGTTCATCATTGTCGT

CGAGGATCTTCTGGAGGAGTTGGTTCCCGGGCAGATTGAACCCTTTCTGCATCGTCTTCACGAGGTGGTCGAGGCTCCAT

TTATCGTTTCAAACGGGCAACAGGCATTTGTCGGAATGAGTTTGGGGCTGGCTTTTTACCCCCTTGATGCAACGGAAGGA

GATGCTCTGGTCCGTCAGGCTGATATCGCGATGTACAAGTTAAAAAAAGAAAAAGATAATCGAACAGTATGGTGGCAAAG

AACTGACCAGTCTTCCCACATGGCCGGTTCTCCTGAACAGCTCAAGTCCTCCGATCCCTATGGTTCGGTGGTAAAGGTTC

TTTTGGGAGGATATCAGCGAGAGATTGCCGTCGTCATAGAAAAGGTGGTCGGGAGTTTTTTTGACAAGCTGGCTCTGGAA

TCTGAGCCCGCTGTCATCTTTTCAAGCCTGTCCCCGGAAGAAATGATGCGTCTCAGGCTTCGCCAGGTTGAACATCTCAA

GTTTCTTCTGGACCCTGAAACGACCAGGGAGGCCATTCTTGCCCGTGCAACGGAAATTGGGCGCATCCACTGTCTCGTGG

GTGTCGGGAACACTCTTCTGCTCAAGGGAAAATCTTTTTATGTGAGATATCTTAAGGACTATCTGGATCAGGAAATACTG

GTTTTGATGGATCGTACGTACATTTTGGTGGCAGCAGAATGGCGCCTCCATGAAGATGTCGAGTCCCAGCTTCGTTCCGA

GGTGACCACGATGGACCACTACCAGAGGATCCTGTCCTTGCCTCTTCCCCCGAAGGGAATAATGTGGACGGAGGCACGCG

CCATTGAAGTGAGGGAACTTGGGCGCTTGCCTGGTGTTCAGGCTGTTTTGTTGGTCAAACCTGACAGTCGTGGTGTATTC

GTGATTGAGGAAAATGGTGGAGCAGTCGGAGAGGTCGGTTCATTTGTATTGCGGACACAGTCGCTTGAGCCGGTCATAGA

TCCTGCTTCTCCCCGTGGTCAAGGCCTTTTATCCAGGACATGGCGCTCTCTCCATTTTCAAACGGCTCCTTCTTTTCATC

TTGATCCGACCCTTGCTCCCTGGCTTGATGTGGTCACCAGGCTGGGAATCAATTCGGCGATGGTTCTTCTGGTCAGGGAT

GATTCCAAAAAACCGGTGGCCGCCATTGCCATTTACGGAGCTTATCCCAATCAGTTCGAGTCGTCCTGGATGAAGCAGTT

TGCCCTGGGGGTTGAGCAACGATGGGGGAAAATCTGGACCCTGAGCCAACAT

>orf3404 LfeRT32a_3404 Putative diguanylate cyclase/phosphodiesterase with PAS/PAC sensor(s)

ATGTCCCCCGATATCTCTCGCCAGGGAGAGTTCCAGACCAGACTGCTACCTCAGCTTCTCGATCTTCTGATCGACCCCGC

TTTCATCGTGGAATTGAAAGAGGGAGATCCTTTTGTGATTTCGGGAGCAAACACTGCATTCTTGCATCTCATGGGATCCC

GGGAAAAAGAAGAGATTTTGGGGCAGTCGTTCGAGGGGTATCTTGACTCGTCGGACAAGACCCCTCTTTTTTTGCCGGAA

GACGTGGCTTCGGATTCTTCGCCTGAAAAAGAGGCTCTGCGCCAGGACAGAGTTCTCCTTCGAAAGGACAAGAGCCGATT

CCTGTCAGAGATACGGATGAAACGATTGATGTTTGGCGAAATCCCCTTTTTGCTGGTGACGGTTCGGGATATCACGGAGA

TGGCTCAACAGGGGCTGTTTCAGGAAGTCGGGAAGGAGATCGAACATTATGAGAAAAAGGAACAAAGCCTCGAATACCTG

CTTGCCCGCGTTATTGAAAAAATTTCCGGAACCTTTCCCTTCCCTGAGGTGATTTTTTCCGGGTTTGAGGAAAAGTCGTC

CATTTATGGCTCCAGAAATCTTCCCGCCTGTTCTGGGCTGCCCCAGGAAAATGTCTATTACGCAGCCATCGCTCACCCGG

AAAATAGAGCCGGGAATCAGGAAGAGTCCATGACCATGGCTGTCATTTTCAGGGATCGTCAGGATTTGACCCCATCTCTT

CTTTCCCGGCTGGAGCTGTTTTCCCGGAAAATGGAAAAAGCCCTGCGGGATTCCCAGCAACCGGGAAGCAGGGTTCACAG

GGATGCACTCTTTGAACTGGCGCCCGACGGAATATGTCTTCTCGATTCGGATAGTCTGGGCATTATTGACGTAAACCCTG

TTTTTTGCCGGTTGCTGGGATTTCCGGACAAGTCTTTTCTTGTTGGAACATCCATCCTTGACTGGTGGGAACTGACGGAG

TCTTCGGCGCGGGAGATCCTTCACAAGATGATCGATGTCCGTAACGTGTCTTTTTCGTTTGAGCAGAGACACGTCAAGAT

GGACGGATCCCATCTTTTGGCGAGCATCAGCGGCGCATGGATCCCTTACGGCGTGAAGGGAGCGCTCATGCTTCATGTCC

GGGATGTTACCACTGAACATGAAGACGAGATATTAAATCGCATATCGGTGGAGCTCGACCAGAAGATCCTCAGCGGACTT

CCCATCATGGATCTTCTGGAGTTCATTGTCCGCAAGATTTCTCTCGAGTTTTCCTTCCAGATCGTTTTCTTCACGATTCC

CGAGCCAAGTGGCGGGATTGTTTTTGTCGGACTCGATCCCTCATTCCCGAAATACTCTCCCGTTCTGGAGGAACTTTTTT

CAATGAACCGCTGGGATCGGTCTCCCGGAAGAGAGTCTTCCTTCGGACGGGCGATCCGGAGCGGGTTGCCCCAGTTTGTT

ACAGGCGATGAGATTGAAAGCTCTCCTCCTGGCAGTATTTATAATGCGTTTGGCATCGCATCGATTTTTTCCATTCCCGT

TCCCAGAGATGCGGGACAGCTTCCCTGGGGGGCCCTTACGATTGCCGACCACAATATCAACGATCTCTCGGGTCGTCTGA

GAACGCGCCTGATCGAACTGGCCGAAAGAATCAGGATCGCGTTTATGCGGCATGAAGAAATGGATCTTGTCAGGGTCCTG

AAGCTTGCAATGGAGTCTTCCCGAAATATTGAGATCATCGCTTTTAAAGATGGAAGGATTGAATGGGCCAATGAGTCCTT

TTTTAAAATGATCCGGAGCGACGGGAAGTTATTGCCGGATATTGATCTGGCTGGCATTTTTCCTGAACCAGCCGTTAAGG

GGAGAAAAGTCTCACTGATTGAGGCGGTCGCGCTGTCGGAACCTTTTACCGGAGAGTTTGAGGGAGCCACCGGGGGAGGG

CACCGGTTTCTGGTCGAGACGATGATTGTTCCATTGAAAGACCGGACCGGGAAAGCTGACCGGGTTTTAATTCAGCAAAA

GGATATTACCCGGGACAGGGAGATCGATCTCATAGACAGGCTTATGAACCAGCTTGATGAAATGATCCTTCTTGGAACTC

CTTTCCCCATGCTTTCATTTCTTGTCGCAGCAAAAGCACGGGAGATTTTTCATGCGGAAGCCGTGGCAATCGGACTGTTC

GGCGATGATGGAAGGGTTGCTTCTCAAGCTGTGTCGTCTATTTCTCCCGTATTTGAAGAAGAGCTCAGGGAGTGGGGAAG

CCATGTTAAAAGCAGGGAGTCCGGGTTTGGGGAGGATCCTGATTTCCTGCTTCCAGGTTCTTCTTATCTTGCCGGATGGA

TGAACGGGAACAGGATGCAGGAGTTCAGGCGATTTTCCCTGACAGAAAATAACAGGGAGATTGGATATATTGCGTTTTTT

TTCAAAAGAATCGCTGCGCTTGAAGCCGATTCACTGGCCCGGATCGAAAAACTCGCCAGACGATTTTCTCTTGTCTATGA

GCGTTATCAGCAGGAAGAGCAAAGACGTCTCCATGAAACGGCCATGTCAGCGGTTGCGAACGGTATTCTGATTACCGGAT

CGGATCGCCAGATCCAGTGGGTTAACGATGCCTTTTTGCAGATGTCCGGACATGAAAGGGATGATCTGATCGGACAGGTT

CCGTTTATTCTTCGCGATTCTCCGGGTGAAGACAGGTCAGCCAGCAATTTTTGGAAAAAAATCTTTTCCGGAGAAACGTT

TGAAGGATTTCTTGAGGATCAAAAAAAGGACGGAAGCCGCTATATGGTAGAGGCGACGGTTACCCCCATTCTCATCAATG

ATGAGATCAGGAATTTTGTGGTCATCCAGAAGGACCAGACACAAAGAATCCAGCAAGAACAGGAGTTCTGGAGACTGGCC

CATACCGATCATCTCACCGGTTTGCTCAATCGTCAGGCCTTTATGGAGCGGATCAATCTCGAGATTGGCCATTGTCAGAG

TTCAGGTAATGGGCTGGCGCTCCTTTTCCTTGATATGGATGGCTTCAAGGAGATCAACGACACCTGGGGCCATGGAGCGG

GAGATTACTTTTTAAAGAGCATCGGAGAGCGGATCCTGGCACATATCCGGTCTTCGGACATTGTTGCAAGGATAGGCGGG

GATGAGTTTGTTGTTCTTCTTGAAATGTCTTCTGAACAGAAAGACCTGGGTCCTTTCCTGGACAGTTTTGTAAAGCGCCT

TTCTGTTCCTGTGGACTATGATGGAAGAAGTCTTCAGGCGACTGTGTCGATCGGCGTATCTTTTTTCCCGAAAGATGCCC

TGTCTGCGGAAGAAATGATCCGGAAGGCCGATATGGCGATGTATGCTTCGAAAAACATGGGCAAGAACCGCTGGAGTTTC

TGCAGGAAAGACTCTGCGACA

>orf1094 LfeRT32a_1094 Putative diguanylate cyclase/phosphodiesterase with PAS/PAC sensor(s)

TTGAAAAAAGGATATTCTGAGTCACACTCTCTCTTCCCAATCCGACTGACCCGTAGATCTCTTTTTTTGGGGAACTTCCT

GGTTCTTTATGTTTATTTGGTTCTTGTTTTTGGTGTTGGATTCTTTTTTCCCCACTTTTCTCCCTTTTGGCCTCCCGCCG

CCGTTGCCGCTTTTGCCATCCTGGCTTACGGGTTCAGATTGGCGCCCGGAGTTTTTCTGGGAGCCTTTTTGGGCAATGCT

TTCATTCTGCACTGGTCTGTTGTTGAATCCCTTCTGATTGCTTCCGGAAATACTCTGGGCCCTCTGCTGGGCGCCAGATT

CCTTCACAGCGGAAATTCTCCGTGGAACGGGTTAGGGACAGTCAGGGATGCTCTCAGATTCCTTGGTGGAATGGGTGTTC

TCGGGAGTGGCCTGACTTCGATGATTGGAGCGCTGGTCCTTGTTGAGCTTTCTCCAGGTGGCCATGCACCGACGCTGCTT

GCGGCATGGCTTGCCTGGTGGACAAGCGATTCCTGCAGCATCTTCATGTTTACTCCGGCCATTTTTGTCTGGAGTTTTCC

TGAGAGGGATGTTCGCTCTATTGAATCTTCTGCGCCTTCGAATATCGTCATCAGTGCCCTGATCTTGTCCATTTTATTTG

TGGGCGGGATCATCTATTTTTTTCCCGGCCTGCCGGAAATGACAAGCCTTGGTCTGACTGGCCTGTTTCTGCCGATTCTT

GTATGGGTTGCGATGACGCGTTCCCAGCGGACGGCGTTCTCTCTTCTGGCGTTTGTCCTTTTCCTGCAGTTTGGGGCAAC

GGCCATGGGTTTTGGTCCTTTTGCCCACCTCGTGAAAAATCCACAAGATGCAATGATCGGCATGGAACTTCTCGGAATGA

TGACCGGATTTGCTGTCATTCTGGTCAACATATTGACCCTTGAAAGACAAGCGGCGATGAATCAGCTGGAACAGATGAAC

TACACCCTTGAGTTTAGGGTTGAAACCCGGACAAAAGACCTTGATCTGAAAACAAGGGAGTTGGGGGCACAGCTCCATTT

CCGTCAACTTCTTCTGGATTCTCTTCCTGTCCCGGTTTTTGTCGCGGATTCAGGGGGGCGTCTTGCTCTTTCGAACCAGA

AACTTGGTGAATTGTTTGGTGTTCCTTCATCTGATCTTCTGGGAAAGCCGGTGAGTGAGCTTTTCGATTCCGGATTTTGT

GAAATGGCTCTGGATCGTCCAGATTCTTTTCCGGATTCCCCTGAAGGGGAGAGGGAAGATCTCATCATGCTTTCAAACGG

GAAGAAGCGGACATTTATTGCAAACAGGGTCTGTATCCAGAGCCCGCTTCGGGGAATGATCGTTTCCCTCCAGGACATCT

CTGAAAGAGTTTCTCTGCAAAGAAGCATCGAGGAAAGGGAGCAGCTTTTTCGCCTGATCGTCGAGACGCTTCCTCTTCCC

ATGATCATTTCAAGAAAAAGCGATTCAGTGCTCCTGTATGCCAATCCTTCGGTAGGGGAGCTGTTTCAGATTGATATCGG

ATTATGGATTGGAAAACCTCTTCTTCCATTTTGGGGAGAGAGCCATGACCGGGACCTGTTTCTCTCTGAAGTTCGTCAGA

AAGGTGTTGTCATTGACCGGGAATTTGAACTGAAAATGCCATCGGGGGAAAGGCTCTGGATGGCGTTGTCCGGCGGTTTT

TCCCAGATTTCAGATGAAGAGGTTCTGATCGTTTCTTTCAGGGATATCCATGAGGACAGGCAACGGCAGATGACTCTTCA

TCAGGAAATCCGGACGGATTTTCTCACTGGTCTGGGAAACCGCAAGGATTTGCAGGAAACTCTTCCGTCAGCTATTGTGC

GGTCCAAATTTCTTGTCGTGTGCATTCTGGATCTGGACGATTTCAAGGAGGTTAACGACCGTTATGGCCATGCTGCAGGG

GATTTTCTGCTCAAGGAAGTCTCGCAGAGGATGCGCTCCTCCCTTCGCGCCGGAGATTATGCGGCAAGACTGGGAGGGGA

TGAGTTTGTCCTGATCCTGGAAAAGATGGAGTCAAGGGAGGATCTGTCCAGGTTTCTGGGCAGATTCAGGAACATCATTG

AACGTCCGATATCTCTTCCTCAGGGCGAAGTTGTTTCTATCGGATTGAGTCTTGGGCTTACGATCTGCCGGGAAGGGGAT

GCCGACCCGGATCTTCTGATGAGGCAGGCAGATGAGGCCCTCTATCAGGTCAAGTCTCAAAAAGGATCCAGAGATTGCTG

GTGGAAGAGCTATTCC

>orf1930 LfeRT32a_1930 Diguanylate cyclase

TTGAAACATCGCCTGGTTTGGTCAGTCGGAGCCCTCATCGCTTTTCTTGTCGGAACGACCCTTGTGTTCGGAATCCAGAC

TTCCCTCAAGAGAGCCCAGTTCAAAAAAGACTTCAAGAGCCTCCTTTTCCATCACCGTATCATCACTCTCTCCCTCAAGC

TCCTCTCAGACGCCAAAGATCTTGAAACCGGGCAAAGGGGATTTTTGCTGACAGGAGATTCTTCTTATCTTGAGCCTTAC

CAGAATAGCCGGACGTCGATTCTTGCCCACCTTGACATGCTGTTGGCGGCCAGCCAAAAATCTCCGGCCATTCTTGCCCA

GGCAGAAGCTTCCGGGCAGCTGCTTGCCAGAGAGATATCCATCTTGAACCGTGCTATCGTCACACAGGAGGTCTTCGGGC

AGAAGAGGGCTCTCCAAATAGTCACTACAGGAGAACCCAAAAGGAGGATGGACAACCTTCGCTCTTCCATCGGAAATATT

CTTTTGACCCAGAACAGGGAAATTGTTCTTATTGAAGCCCGCACCTACAAAGACCGCAGGAATGTGAAAAGGAACTTCCT

CATTTTTATGGGCGGCTCCGTCTCGTTCATTATAATCGCCCTTTTCATTATCCTCGGGGAGATTCGAAAGAACAGCAGAC

TTATGAAACGTCTTGAGAACGAATCTTCTCACGATGAACTGACAGGAATTCCCAACCGAAGATTCCTGCAGGAATGGATC

AGGATCGAAATACTCAAGGACAGGAATAAAGAATTTCCATTCCTTCTCCTTTTTCTTGATTTAAATCACTTTAAATCCAT

AAACGACCGCCTTGGTCATGACGTGGGGGATATGGTCCTGAAAAATGCAGTCAAGCGATTCCAGACAGCACTCAGGGAAG

GAGATCTTCTTGCCAGAATTGGTGGCGATGAATTTATCGCGATCATCAGGGGAAAGATTTCCAGGGAAGAACAGAACGAC

CTGATCATGAGACTCAAGAAGACACTTCTCCAACCATCCCTTCTTCCCAAAGATTTTCCTGTCCGTTTCGGATTAAGTAT

CGGAGTTGCCACCTACCCGGAAGACGGAGAAACTCTCGACGCACTTCTCCGCGTTGCCGATAAAGGAATGTACGAAGACA

AGCAAACCGGCCATGCCTCT

>clpX LfeRT32a_3602 ATP-dependent Clp protease ATP-binding subunit clpX

ATGGCTGGTAAGGAAAAAAAAGACAAGTCGGATGGGAGCGGGGTGTCATGCTCTTTTTGTGGAAAGCCAAGGGAAGATGT

CCGGAAGATGGTTTCCGGTCCTGGCGTCTTCATTTGTGATGAGTGTATTGATCAATGCTCCTCAATCATGTCCGAGACGT

GGGAAGAGGAAAAAGAGCCATCTGGAGCAGCGCTCCTGAAGCCTGTCGAGATCAACCGGATTCTTGACCAGTATGTTGTT

GGCCAGGAGAGGGCGAAGAAAGTCCTTTCTGTCGCAGTTTACAATCATTATAAACGCATTGCTGCCAATCAGAGTTCTGA

TGATGTTGAGCTCCAGAAGGGCAATATTCTGATGATTGGCCCGACCGGCACAGGGAAAACACTTCTGGCTCAGACATTGG

CGAGAATCCTGGATGTCCCCTTTGCGATTGCGGATGCAACGACTCTGACTGAAGCTGGCTATGTCGGAGAAGATGTTGAA

AATATCATCCTCAAACTTTTGCAGTCGGCAGACTATGATGTTGAAAAGGCGGAGAAAGGCATTATCTATATCGATGAAAT

CGATAAAATCTCCCGAAAGTCAGAAAATCCCTCCATTACACGGGATGTTTCCGGAGAAGGTGTTCAGCAGGCTTTGCTTA

AGCTGGTTGAGGGGACCGTTGCAAATGTGCCTCCACAGGGAGGAAGAAAGCACCCCCACCAAGAGTTTATTCAGGTTGAT

ACGACCAATATCCTCTTCATTTGTGGCGGTGCATTTATAGGATTGGATAATATTGTTTCGCAGAGAATTTCTAATAAAAA

CCTTGGTTTTGGCGCAGATATCAGGACAGTTGAGGATCGAAAGAAGGTTGGGGAGCTTCTGGTCAAATCTCGTCCGGATG

ACCTTCTGAAATATGGTCTGATTCCTGAGTTTGTTGGCAGATTCCCTGTCATGGCCGTTCTTGAGGATCTGGATGAAGAA

GCATTCCTGCGAATATTGACAGAGCCTAAAAATGCGCTCATCAAGCAGTTTGAGAAGCTTTTTTCAATCGAACATGTTCA

GTTGACATTCAGTGAGGCGGCTCTTAAGGCAATTGCCCACAAGGCCTTTGTCCAGAAGACTGGAGCCCGTGGGTTGCGCT

CGATACTTGAAGAAGTCATGCTTGATCTTATGTACGACATTCCTTCTGTGTCGAATGTCCGGGAGGTTATTATTTCTGAA

GAAACAATAAATGCTGGTGCGTTGCCAAAGATTGTCAGGGATGACTCTCCGAAAAAAGCTGCTCCTGGAGCCATGAGCGC

C

>clpP LfeRT32a_3603 ATP-dependent Clp protease proteolytic subunit

ATGTTGATACCGATGGTCGTAGAACACACCAGTCGCGGAGAGCGTTCCTATGATATTTATTCGAGGCTTTTGAAGGAGCG

GATCATTTTTTTGGGAACTCCTATTGATGACAATGTTGCAAATCTTGTAATCGCCCAGCTGCTCTTTCTGGAGTCGGAGG

ATGCATCAAAGGATATCAATATTTACATCAACTCTCCTGGTGGTGTTGTAACCGCAGGTTTTGCGATCTATGACACAATG

CAGTACATCAAGCCTGATGTATCAACGATATGTGTTGGTCAAGCAGCCAGTATGGGAGCATTTCTTCTTGCAGCAGGAGC

AAAGGGAAAACGTTTCGCTCTTCCCAATGCCAGAGTCATGATTCATCAACCGCTGGGTGGATTCCAGGGACAGGCAACAG

ATATTGCAATTCATGCCAAAGAGATTCTTAAAATGAAATCGCACTTGAATGAATTGCTTTCCCATCATACCGGACAAAGT

CTGCAGAGAATCGAACAGGATACGGAGAGAGACTACTTTTTGTCAGCCGATGAGTCAAAGGAATACGGTTTGGTGGATAA

AGTCATGAGCTCGCATGGGTTATCAGAGACTCCTTCTGTC

>lonD LfeRT32a_3201 ATP-dependent protease La 2

ATGGTTGAGGAACAGGTCCGGACACCTGGCGAATCCCCTTTGGTCATTTTGCAGGATTCGGTTGTTTTTCCACATATATT

GACATCGCTGGCTTTTCATCATCCCATGGATCTGGCCGCGATCGATGATGCGATGAACCGGGAGCCGAAGACACTGATCT

GTGTAACGGCCAGAGACTCTGGAAAAGAGGAGGCCGAGAGTACCGGGGATGATTCGACGGGTCATCCTCCGGTGACTCTC

GGGGATCTCCATGAAATCGGCACGATGGTTCTTATCCACAAACTCCTCAGAATTCCGGCAGGGGGTGTCGCCATCATGGT

CCAGGGGATCCGCAGGGTTAAAATCGATGACATCCTCCAGACAGACCCCGTTCTTCGCGTAAAGGTCCATCCTGCTCCCG

AGTCACCCGATCGTTCGGTGGAAACCGAAGCCCTGATGCGCCTGATCCTTGGACAGGTCAAGCAGTTGGCGGGACTTGCT

CCGTATCTTCCCGATGAGTTTGAGACAATGGCACTTAATATCGATAACCCCCATCACCTTGCCTATCTTGTCGTGACGTT

TCTGAAGTTGCCGGTAGATGATCGCCAGAAAATTCTTGAGATTGATTCGGCCGAGGCCAAATTGACCAATCTGTCCACCT

ACCTGGAAAGGGAAATAGAGCTTCTTGAACTGGGTGGAAAAATAAAGGCCAAAATTCAGGACGATGTCCAGAAAAGTCAG

CGGGATTTTTTCCTTCGCGAACAGGTCAGGGCAATTCAGAAAGAGCTTGGTGATGGTGAGGAGGGTGATGAGGACATCGT

CCGTTACCGGGAAAAAATAGCAAAAAGCCAGCTCCCTCCAGAGGTCCTGCGTGAAGTTAACCGGGAACTCGACCGTCTGG

TTCGTTCGGGTAACGGACAGTCCCAGGAGGCCGGAGTGATCCGCACCTATCTTGATGTTGTTCTTGATCTTCCTTGGGCC

AGGACGGCCACTGACCATTTCTCCATCGAAAAGGCCCAGCAAATCCTTGATGAAGATCATTATGGGATCGAAAAGGTAAA

GGACAGGATCCTGGATGAGCTGGCGGTTCACCTCCGTGCAAAGGGAAAGAACAGGGGACCGGTTCTGTGCTTTATTGGGC

CACCAGGCGTTGGCAAGACAACATTGGGACAGTCCATCGCCCGTGCGATGGGTCGGGCTTTTGTGCGTGTCTCTCTGGGA

GGCGTGAGAGATGACTCCGAGATCAGGGGACATCGACGGACCTATGTCGGGGCGATGCCCGGCAGGATTATCCAGGGGAT

GAGAAAGGCCGGAACACATAATCCTGTTTTCATGCTGGATGAGATCGACAAGCTCGGGGCTGATCAGCGAGGCGATCCAG

CCTCGGCCCTTCTGGAAGTCCTTGATGCCTCCCAGAACAAGGATTTTCGGGATCACTACCTTGATCTCCCCTTCGATCTG

TCCGAAGTCTTTTTTATTGCGACGGCCAATGTCTTTCAGACTATTCCTCCCCCGTTGCTTGACCGGATGGAAATCATCAG

GCTTGCCGGCTACACATGGGAGGAGAAGAGGCATATTGCACGGCAGTATCTGATCCCGCGTGTGATGAAAGAGCTTGGGA

TCGATCCTTTCGAATTCCGATTTGATGATCCGGCCCTGGTTCGTCTGATTAGGGAGTTTACCCGTGAAGCCGGGGTCAGA

ACACTTGACCGGAAAATCGCAACACTTCTCCGAAAGGTCGTGAGAGTTCGTTCTGAAATGTCACGGAAGAGAAAGGTGAT

CATTACGCCAAAATCCATTTCCACCTATCTTGGAAATGAATATGTTGAACCGGAACATCTTCTGGACAAGGCTCCTCCCG

GGGTTGCGACCGGATTGGCCTGGACTCCGAACGGGGGAGATGTGCTTTTTATTGAAACGCTGGCCATCGAGGGATCAAAG

GGGTTCATCCTGACCGGACATCTCGGGGATGTCATGAAGGAGTCGGCGAGAACCGCCTTTTCAGTTGTGCAGTCAAGAAT

TGGGCGGTTGGGAATCGAATCGAACTTTTTTACGAAAAATGAGATTCATGTCCATGTGCCAGGAGGTGCCATTCCGAAAG

ATGGTCCTTCGGCGGGGATTACGATTGCTGTTGCAATCGTCTCTCTCGTCACCGGAATCCCTGTTCCTGAAACGCTTGCG

ATGACAGGAGAAATTGCTCTTTCCGGAAGGGTCCTGCCCGTCGGCGGAATCAAGGAGAAGCTGATTGGTGCCCGGGAGGC

AGGCATCAAAAAAGTCTTTATTCCCGTAAAAAACGAGAAAGATCTTCTGGAGATCCCGGAAGAGGTCAAGAATGATCTGA

CAATTGTTCTTGTTTCCCATATTGATGATATTTTCGGGGAGTGTTTTAAGATCGGAAAAGGGAAGAAAGCTGCCTTGTCC

CCACATGGAAAGAAATCTCAG

>lon LfeRT32a_1195 Lon protease homolog 1, mitochondrial precursor

ATGACCGGATTCCGGATTCAGAAAAAGCGTCTCATGAAATCGGAAATTTCCCGCCCGACGGCGAAGAAGGAGCGAGCCCC

CGAGATCGACGCCCAAGAGGACGAGGCAGTCGAACCCGACGAACCCATTCCTCTTTATATGTTCGAAAATGTCATCCGGG

ACCCGTTCCTCCATTGTCTTCCGGTCGATCCCACGAGTTTGAGGAGGTCGATCGATGAATGCCGGAAAAACATGAATCCC

AAAAAGGAGGAAAACAGAAAGAGGTGGCTCGATCTGGCGGAAAAGGTTCTCAAGAACGAGAGGATCCGTCTGGCTTTTCC

TCCCACTGCCGAGACATGCGAACGTCTGGCCGACAAGTACCCCCATCTGGCGAATGCCGTCCGGGTCGTGCGGGACTTTC

TCGATATTTCCCGTCTGGCGGAGGGGCCGGCGACTCTTCCGCCGATTCTTCTGAGCGGGCCTCCGGGGGCAGGGAAGACG

GCCTTCGCCCTCGACCTGGCAATAACGCTGGGTGTGCCGTCTCGGGTCGTGAATTCGGCCAGTCTCACCCATTCGTTCAT

CCTGGGAGGGACCGATGCGGTTTGGGGGTCCTCGAAGGAAGGGATGATTCTGGACCTTCTCCTGGAAGGATACGGAAACC

CGCTTATGATCCTCGACGAGATCGACAAGGCCGGAAGAAGCCTGTCGGGATCCTCGAATGCGCCCTCGATTGAAGATTTT

CTGTTGAATGTGCTGGAGCCGGTCACGGCCAGACGATACACCGACGAATTTCTGTCCTCGGCCCACCCGGTGGATGCCTC

GAAGGTCCAATGGGTCTTCACGGCGAACGATCTGAAAGGCCTGTCGACACCGCTCCTGTCGCGCCTGACCGTGATTTCCG

TCCGGGAGCCGACAGCGGAGGAATTTCGGGAGGTGATCCTTCCCTCCCTCTACCGGAACATACTCGAAGAGTACGGCCTC

TCCGGAAAGGTGCCCGAATCTCTCCCGGAGGATGCCATCGGGTCGCTCTCGGGCTCGCCCCGGGAGTCCCGGAAACGGAT

TCTCCGGCTTCTCGCCGGATATGCCCGGGAGGGGAAGTTTGAGGCTCTGGAAGAAAAGGTCCCCGGGACGGAAAAACGGA

TTGGATTCTACGCAGGAGGAAGCAAA

>pepA LfeRT32a_1834 Probable cytosol aminopeptidase Leucine aminopeptidase

GTGGATCCTGAATTGGTCAAGATTGAAACGATGACGGGGGATCCTCTGGTTCACCCGGCAATGGGGCTTGTTGTTGGTGT

TTTTGATGATGGTCGGGTTGTCGGGGATCTGAAAAAGAAGATCGATGCAGCTCTTTCGGGGGAGCTTTCCAGAATCCTGG

ATGATGGTGCCTTCCGGGGAGAACGCGGGGATGTGATGATCCTCCCGACTCTTGGAAAAATGTCGCCGCAGCATCTTATC

CTGGCCGGGTTGGGGAATAAGGAGTCGCTGACGAACGAGGGCCTTCGCCGGGTATCGGGACAGGTTGCCAAGGCTGCTTC

CAAGCGCCGGATCAAAACGCTTCTTTCAACTCTTTCCGGCGCCTTGAAAGATGCTGCCCTGGCATCTGAAGCGGTTGTCG

AGGGGACTCTTCTGGCCCTTTATCGGTTTGATATCTACAAAACAACAGGTAAAGACAAGGGAGAGCCGAAAGTGCAGGCC

CAGCCACTTGAAGCACTCTGGATTTCTTCCTCCAAGGAGTCCGAGAAAAAGCAGTGCCGGGGGATTTCCGTTGCGAGGGC

GGTTGTCAGGGGCGTTTATCTGGCCCGCGATCTGGGCAATCATCCTGCGAATGTAGCCACTCCATCGATGATATCGGAGA

CTGCGGCAAAGGAAGCGGCTGCCAGGAAGATCTCTTTTGTCAGCTATGATTTCCAGACGATTTCGCAGATGGGTCTTGGT

GCCCTGGCGGGGGTTGCCAAGGGTTCCCTCGAGCCTGCCCGTCTGATCCGTCTTGAGTACAAGCCTAAGAAGCCCATCAA

TAAAAAGCCTGTTCTTCTCGTTGGGAAAACCCTGACTTTTGATTCGGGTGGCATTTCCTTGAAGCCAGCTGAGAAGATGG

AGGCGATGAAGGGTGACATGTCGGGTGGTGCCGCTGTTTTGGGAACGATGATGGCCTGCGCCGATCTTGATATTCCCCTT

CATATTGTCGGGATCATGCCTGCGACCGAAAATATGCCCAGTGGAACCGCTAACAAGCCAGGCGATGTTTTGACCGCTTT

TAACGGAAAAACGATAGAGGTGATCAATACCGATGCGGAAGGTCGGCTCATTCTGGCGGATGCCCTTTCCTGGGGAATCA

AGACATTTCAGCCGGAGTTGACGATTGACCTTGCGACCTTGACCGGTGCTGTTACCGTGGCCCTTGGCTCTCATGCCATC

GGTGTGCTGGGCAACAATCGGGACCTTGTGGGAAGAGCGCTTGAGGCGGGAGAAATCTCCGGAGAAAGAGGATGGGAACT

TCCTCTGTTTGAAGAGTACTACGAGCAGATCAAAAGCCCGATTGCCGACATGCAGAATGTGGGTGGGCGCGGTGCGGGAA

CGATTACCGCTGCGGCGTTTCTTTCAAACTTTGTCGACGACTGTCCATGGCTTCATCTGGATATCGCCGGAACGGCATGG

GTGGAATCCGACGAGCCTTATAAACCCAAGGGAAATGTCGGTGTTGGCATTCGTCTTCTCGTCCATCTTCTTGAAGGGAT

GGTGGAAAAGGAATGGAAAGACCCGAGAGGAGCAAAATCTTCGAAGAAACCGTCCAAAAAG

>oppA LfeRT32a_2777 Oligopeptide transport system

ATGCTTTTTATCCTGCTGCCGTTATCCATTCTTTCTTGTTCCCCCCATTTCCAGCCAGTTTCCCGCCATGATTCACACAC

TCTTTTTATGGCAATGGCAGATGATCCATCGACACTTGACTGGAATAAAGCAACAGACGGTATCAGCTTTGAGGTGATTA

CCAATCTGATGGATGGGTTGACGCGTTTTGACAAAAATCTCGTCGTTTCTCCTGATATCGCCCTTTCCTGGGAATCGGCA

GGGAACAAGGACTATATCTTTCACCTTGATCCCAAGAGAACGTGGAGTGATGGGAAACAGGTAACAGCCTATGATTTCAG

GGATTCTTTTCTAAGGCTCCTTTCCCCGGAAACAGCCTCCCCATACGCCTATTACCTTTTTGATATCAAAAACGCTTCCT

GCTTTCATGCGAATAAGTGCACTTCTTCACAGGTGGGAATAGAGGTTCCCAATGCCGAGACGCTTAAAATACACCTCTCC

CATTCGATGTCGGCCTTTCCCTCCTTACTGACAAGCCCGATTACGGATCCGATCCGAAAGGACCTGATCCAAAAATATAG

GGACGGATGGACACTTCCAGGCCATCTTGTGACAAATGGGCGATTTACTCTAAAAGAATGGTGGCATGGTGACTTTCTTC

TTTTGGAAGCACGAACAGATCTCCCTTCGAACACTTCGCTTGAAAGGATCCAGTTTCTGATCGTTCCTGAGCCGGTTACC

CAGCTTTTGCTCTACGATCGCCATATCCTGGATATCGTGGGCGTTCCGAGTTTTTATGTGAAAAAATACGAGCATTCCCC

GGATCTGCATCGGATTCCCCAGTTCAGCACGGTGTATTACAGCATGAACATCAAGCGTCCTCCTTTTGACAATCTTCATG

TCAGGAAAGCTTTTGCCCTTGCTGTTGACCGCTCGGATCTTCGGGAACTTTTTCAGAATGCATTTCCGGTCTCCCGATCC

TTCATTCCGAAGGGATTGTTGGGGTATTCGGAAAATGCCGGTTACTCGTATAACCCCGACAAAGCCCGTGCAGAATTGGC

GCTTGCCGGTTATCCCGGAGGGAAAAACTTTCCGAGGGTTACCTTTATTTTTCCTTCCGGCACACAAAGCCGCATTCTGG

CCGTTCACTTCCAGGAATCTTTCCGGAAAATTCTCCATGTCACGATCCATCTCCGGTCTCTTGAATGGAAAGCTTTCCTG

GCAAAGCTTGATTCAAAGACTCCCCAGATGTACCAGTCCGGCTGGCTGGCCGATTATCCTGACCCCAATACTTTCATGAC

GTTGATGACAACCGATTCGGGAAACAACCGCACTGGGTGGGGGGATCCTCTTTTTGACCATCTGGTTCTTGAGGCTTCCC

AATCGGCGAACGATGCTCTCCGTCAAAAATTGTACGAAAAAGCCCAATTCCAGCTTTTGAGAGCTGGAGTGCCGGTCATC

CCTCTTTCGGAAGGACTTTCCAACATGCTGGTCCATCAAGATATCAGGGGATTCTGGCATGACCCTCTGGGAACGGATCA

CCTGGAACATGTGAGCAAGGTTCCGGAG

>helA LfeRT32a_1710 chemiosmotic efflux system protein A-like protein

ATGTTCAACCGCCTGATTGCCTGGTCCCTGAACAACCGCCCGATCGTTCTGGCGGTCACCTTGGTTTTATTCGTCTCGGG

ATGGTTTGCCCTCAAGAAGATGCCGGTGGATGTGTTTCCCGAGTTCGCGCCGCCACAGGTCGTCGTTCAGACCGAGGTGC

CGGGTCTGGCGCCGACGGATGTGGAGGCCCTGGTGACCTACCCCCTGGAAAGCGCCATCAACGGAACACCAGGCGTGACC

CATGTGCGCTCAAAAACGTCGGTGGGTCTCTCCACCATCACGGTCATGTTCAAGGCGGGCTCGAACATTTATCAGGACCG

GCAACTGGTCAACGAGCGGATCCAGGCCGTGACGGGCCGCCTGCCTTCTGGCTCCAAATCTCCGATCCTGCTGCCGGTGA

CCTCGGCGGTGGGCTGGCTGGTCAAATATGCTTTGACAAGCAATACCGTGCCACCAGAAAAAATGCGCACCCTGTCGGAC

TGGGAAATCCGTCCGCGCCTCCTGGCCCTGGGCGGTATCGCTTCGGTTGTGTCCATCGGAGGGGAAGTCAAGCAGTATCA

GGTGCGCCTCGATCCTGCCCGCATGTTGGCCTACCAGGTCAGTGGCGAGGAAGTGCGGCGAGCCTTGGAAAAATCCAATA

GCAATGTGCCCGGCGCCTTTCTGGAAAAACCGGGACAGGAACTTATCGTGGCCGGAATCGGGAGAATTTCATCTCTGGAT

GATCTCAGAAAGACTGTGATCACTGTTCGCGACGGGGTGCCCATCACCATTTCCAATGTCGCCAAAGTGGCTTTTGGAGG

ACAAATCAAGCGTGGGGACGGCGCCTTCGGCATGAAAGAGGCCGTCATCGGGACTGTCTCCAAGACCTACGGGGCCGACA

CCGTCACCACTACGGAGAAGGTGGAAAAAACGCTGGCCGAAATCAAGGCACATTTGCCAGCAGGCATACAAATGCACACC

CAAGTATTCCGCCAGGCCAATTTCATCGAGGCGGCGATCCACAATCTCAGCATTTCTTTGCTGGAGGGGGCGCTCATCGT

CATTGTCGTCCTCTTCATCTTCCTCATGAACTGGCGGGCCTCTCTCATCACCTTTCTGTCCATGCCGGCTTCTTTCGTGG

GTGGTATCTTGACGATGCACGCGCTGGGTTTCGGGATCAACGCCATGACCTTGGGAGGACTGGCGATCGCCATCGGCGAG

GTGGTGGACAATGGCATTATCACCGTGGAGAACGTGCTTCGCCGGTTGCGCTTGAACAGAGAGACCGCCCACCCGTTGCC

GTCGATCGAAACGGTGTTCGACGCAGTGCAGGAGATCCTCAATTCGGTGGTGTATGCGACCTTGATCGTCATCCTGATCT

TCCTTCCGATCTTTTTCCTGCAGGGACTCGCCAACCGCATCTTTAGCCCCCTGGGGGTGGCCTATATCGCTTCCGTGACG

GCATCGCTTGTGGTGGCCGTGACCATGATCCCTGCACTGTGCTATCAGTTGCTGGTCGTGTGGGGCAAGAAAGAATGGCG

CGAAAAGGGGGTTACGCTTCTCCCTTTAAACAGCCGGGAGATACCCTCCGATACCACACCGGGGAGGGAGGCACCCATCC

AGGAAGAGAGGGAAACCCGATTCGTGCAATGGCTCAAAAGCCACTACCTGCGCCTCTTGAACCTGGCGCTACGAAAGTTC

TGGTGGGTGGTGGGCTTGGCTGTCCTGGGACTGGGGCTGGCTCTTTCTCTGCTTCCCTTTTTCGGGCGCTCATTTCTGCC

GGAATTTCACGAGGGCAATTACATCATCGGCATGACGACCTTGCCCGGCACCTCACTCGAGGAGTCCATGCGACTGGGGG

CACTGGTACGTCAGGACCTGCTTAAATACCCCCAAGTGATCTCGATCGACCAGCGCGCCGGACGCAGTGAGCTGGACGAA

GACGCCCAGCCGCCAAATTTCAGCGAATTCGATATGCGCCTGGATTTTACCAGGGATCCCAAGATGCCGCCGGATGTTTT

GCTCAAGCATATCCGACAGGATCTGTCCAGGGTTCCTGGGGCAGTATTCAACGTCGGCCAGTTCATTGCCCACCGCATGG

ATGAAGTGCAGTCCGGTGTCCGCGCCCAGGTGGCCGTCAAGATTTTCGGCGACAGCCTTTCCACCCTGTACCAACTCGGT

CAACGGGTGGAAGGGGTGTTGCGCGGCGAGCCCGGGGTGGTCGATGTCAACGTGGAGCAGCAGATCCGGGTGCCGCAACT

GACCATCAGGGTCGATCGCACCACGGCCGCGCGCTATGGCATCAAAGCAGGGGATTTGATGCAGAACATCCAGATGTACC

TGAACGGGGAAACGGTGTCCAGTGTGCAGGAGGGCCGCCGCAGTTTTGATCTTTACGTGCGATTGGAGCACTCGGCACGC

AACAGCGTGGAGACCATCCGCGACATGCTGGTGGATGCACCCGGACTGGATTCCACCCACAACGTCAAAGTGCCTTTGCG

CGAAGTGGCCAGCGTATCCCTCCAGGACGAGCCTTACTCGATCAGCCGGGAAAACGATCGGCGCCTGATCATCGTAAGCT

TCAACACCCAGGGGGGGGATTTGAGCGGCATCATCCGCGATATCCAGGCCAAAATCGGAGAGCAGATCTCACTGCCCCCC

GGCTACTCCATCGAGTTCGGTGGCCAGTTTGAAAGCCAGCAGCAAGCCAACCGGACCTTGCTGGTCTTCGGCACCCTGGC

CTTGTTCGGGGCACTGATTCTGCTATACAAGGCCTTCGGCACCTTCCGCGAAGCGTTGTTGGTGTTGATCAATTTGCCTT

TGGCAATGATAGGCGGCGTCATGGCGTTATACCTGGCTGGCGCCGATATGAGCGTGGCGGCCGTGATCGGCTTCATCACC

CTGTTTGGCATCGCCACCCGAAACGGCATCATCCTGATTTCCCACTACAATCAATTGCGCAAGGAAGGCAAACCCCTGGA

GGAAGTGGTCGTGTCGGGTACGCTCGATCGGCTGGTGCCGGTGCTGATGACCGCGGCCACCGCCTCGCTGGGACTGATCC

CGATACTGTGGGGATCGCCCGTTGGCAAGGAGCTGGAGCGTCCGCTGGCGCAGGTGGTGCTGGGCGGCCTGCTGACCTCC

ACCTTCCTGAATATGATTGTCATCCCCTCTGTTTACAACCGCATGGAGCAATGGCGAGAACGCCGGATGTTCAGAGGCTG

GGGCAATAGCACTTCAGGGATCGTTCCCGAACAAGATCCAAAATGTTCTTCAGGACCATGCGCA

>modB LfeRT32a_2725 Molybdenum transport system permease

ATGGACCATTCGGCTCTCTATGTGACCGTGTCGCTTTCTCTTCTCACCGCAACGATCCTCACCGTGTCGAGCCTCCCGTT

TGCATGGTGGATCGTTTTTTCCGGAAGCCGCTGGGTCACGCTCGGGGAGGCGTTCCTCACGCTCACACTGGTGCTGCCTC

CGGCTGTCCTTGGATATCTCCTTCTGGTTGTTTTCAGCCCCGACAATCCGTTCGGGGGATTTTTTGCAAAGGTCCTGGGC

CATCCACTCCCTTTTTCCTTTGAAGGCCTCCTTGTCGCATCCCTCCTCTACAGCCTTCCATTTGCCGTCCAGCCGGTGGC

CCAGGCCTTCAGGCAAATCCCCGAAAGCACCCTTGAGATGGCGAGACTCCTTGGTGCTGGACGGCTGAAAACATTTTTCC

GGATTGTCGTCCCCCTGTCTTTTTCCGGTCTCTGGACAGGATGGATCCTTGGATTTGCCCACACCGTCGGAGAGTTCGGC

GTCGTCATGATGGTGGGAGGAAATATTCCCGGTTCGACACGAACGCTTTCGCTAAAAGCCTATGACGATCTTCTTTCGAT

GAACAATAAAAGCGCATGGGAGTCCATTGGCTATCTTCTTCTCTTTTCCTTTCTGGCTCTCACCGCGCTGAACCTGATCC

GCCGAAAGGAAACACCCCGTGATTCCATCGAAGTCACTCGACGT

>secD LfeRT32a_2747 Protein-export membrane protein SecD

ATGAAGAAAAAGCTTGGATTCAGGATCGCCCTTTTGACAGGTGCGGTCATTATTTCTTTTCTCCTGATTTTGCCTTCACT

TCCCATCTGGAACAACGTTCCTGACAGCATGAAGAAGTTTCTTCCGGAAGGGAAAATCTCCCTTGGTCTGGATCTGAAGG

GCGGAATGTCCGTGACGCTTGAAGTGGACCAGGACAAGGCTGTTTCAGGGACCATCGATGAGATGGTCACAGGCCTCAAG

GAAGCAGGTTTTGAATCTGTCCGCTCGGGAGATTCCATTGTCCTGACCGTCCCCCCCGGGCCCCATCGGGCGGCTGTCAG

GAAGACCGTTTCAAGACGATACCCGGCCGTTGTGGAAACATCAAAAAATGACAGGACCATTACCTACAGCCTTCCGGCGC

CGGAAGTGAAGCGCATACGGGAACATGCAATGACGCAGGCGGTCGAAGTCATCAGAAATCGAATTGACCAGTTCGGAGTC

TCAGAACCGGTGATTTCCCGGCAGGGGAAAAATCGTATTCTGATCGAACTTCCGGGCGTCAGCGATCCCGAAAGGGCGAT

GGCCCTGATCGGAAGAACGGCAAGGCTTGAGTTCATGCTTGTCGACGACAGGAACCCCCAGGCGGAAGCCATTATCGAAA

AAAAGGTTCCGGTACCGGCCGACGACATCCTTCTTTCCGGTCAGCCTTCTGAAAAGTCAGGTGGCGTTGCCACTCCATAC

CTCCTGAAAAAGCAGGCACTGATGACGGGAGAGATGATCTCTGATGCCAGGGTATCCTTTGGCCAGTTCAATGAGCCCTA

TGTGTCGATGACCTTCAACTCTTCCGGCGCAAAGCTCTTCGACGAAATCACGAAAGACCATGTCAAGCAGCTTCTGGCCA

TTGTTCTTGACAACCGTGTCTACAGCGCCCCTGTCATCCAGGAAGAGATCTCCGGTGGACAGGCACAGATTACGGGATCT

TTTTCCCTCAAGGAAGCAAAGGATCTGTCGATTGTGCTCCGGTCCGGAGCCCTTCCGGCCCCCGTCCGGATTCTTCAGAA

TGTCACTGTAGGTCCTTCTCTTGGTCGTGACTCAATCCAGTCCGGCATTCGGGCAACGGTTCTTGCCGTAATTCTGGTTC

TGCTGTTCATGGCCTTCTACTACCGCTTCTCCGGTGTCATCGCCGATTTTGCGCTGGTCTTAAACCTGATCATTCTTGTC

GGAGCACTGGCGGCGCTTCATGCCACACTGACACTCCCGGGCATTGCCGGCATTATCCTGACGGTGGGAATGGGTGTCGA

CTCAAACGTCCTGATTCTCGAGAGAATCAGGGAAGAAATCCGTGCGGGAAAACCGGTCAGGACATCCATCGATTCAGGAT

ATGACAAGGCCTTTCTGACCATTGTGGATTCTCATGTGACGACATTGATCACCGCCCTGATCCTGTTTATCTTCGGGACC

GGACCGATCAAGGGATTTGCCGTGACGCTTTCAGTCGGGATCGGGATCAACCTCTTTACGGCACTTGTCGGAACCCGGGT

CGTCTATGATTATATGGCCTCCAGGCGGAAGATGCAGAAACTTTCGATC

>ffh LfeRT32a_0763 Signal recognition particle protein (SRP) protein Ffh

ATGTTTGAAGGATTATCGCAGCGTTTTGATCAGATACTGAGGAAAATCACCGGGAAGGGCCTTCTGACGGAGTCCCAGGT

TGGCGAGACCCTTCAGGAGATCCGTACAGCTCTTCTCGAAGCTGATGTGACGCTTGATGTTGTGACAAGGTTCGTTGAGA

CACTTCGTCAGAAGCTTGTTGGTACAGAGGTTTCTAAAGCATTGACCCCTGTCGAGACGGTTTTGAAGGAGGTTTACTCA

GAGGTTGTCAGGATCCTTGGCGAAAACAAGGCAACAATACAGCTTTCATCAAAGCCTCCAACGATCATTTTTCTGATGGG

GCTGCAGGGGTCAGGAAAGACTACAACCGTTGCAAAGCTTGCGTATCACTTCCGGCAGTCAGGAAAGCGTGTCTTGATGG

TGGCATCTGATCTCCAGCGCCTGGCTGCTGTTGAACAGTTGCGGGTGTTGGGTGAGCAGGTTGGTGTTCCTGTCGTTTTG

CCGAAAGATAATGTTACAAAGCCGAAAGACATGTACTCAGATGTCAGGCGTCGATGGATCGAAGGAATGCATGAGGTGGT

GATTGTTGATACTGCCGGTCGCCTTTCAATTGATGATGAACTGATGAATGAGCTCCAGGAGTTGAAGGCATTGTACAATC

CAAAAGAATCGCTTTTGGTCCTTGATGCCATGACTGGACAGGAGTCAGTTCATGTTGCCCAGACCTTTGACCAGAAAATT

GGTCTTGATGGGGTGATCTTCACCAAGCTTGATGGCGATGCACGGGCCGGTGCCATCCTGTCGATCAGAAGCGTTCTTGG

AAAACCGATCAAGTTTGTTGGAACCGGAGAAAAAACAGACCGGCTTGAACCTTTTTATCCTGACAGGGTTGCCTCCCGAA

TTATCGGAATGGGGGATATCCAGACTCTGATCGAAAAAGCTCAGCATGCAGTGACCAAGGAAGAAGCTGAGAAAATGGTT

AATCGGGTTGTCAAGAACCAGATAACCTTCGATGATTTTCTGGATCAGATTCGCACGATGAGAAAAATGGGATCCGTTAA

TGATATATTATCGATGATTCCGGGTGCATCGAGTGTCAAGGACAAGCTGGACATGGGTGTTGTCGAGAAAGAGATGAAGA

GGACTGAAGCCGTCATTCTTTCCATGACAAAAAAGGAAAGAATTTCTCCTGAGATTATTGATGGCAGCAGGAGACGCCGG

ATAGCATCCGGTTCGGGGACAACGGTACAGGTCGTCAATCAGGTATTGCGTCAGTTTGAGCAGGTGAGAAAAATGATGCG

CACAGCCCTTAAGCCAAATGGTAAAAGAGCATTGCGTTCGATGCTTCCCTTT

>ompA LfeRT32a_2326 Outer membrane protein OmpA family

ATGAAACAAAATACTATTAATGCCTTGATGCCCGGATCTGTTTTGGTCTGCATGCTTTTGACCAGCTGTGCCAGTGTTCC

ACCCCACAATGCCAGGCTTGATTCAGCCGTCTCGGACTACCAGTCTGCGAGCCATGATCCTGCCGTGATGCAGGCAGCCC

CCGAACAGCTTGAGAAGGCCTCGTCCGCAATTAAAAAGGCCGAGCGGCTATATGGCAAGAATCCATCCGATGCCGATGTC

GACCATTATGCTTATCTGGCGCAGAAGCGTGTCGAGATTGCCAGGGAAAAAGCGGAGAACGATGCTATTCAGGCAAAGAT

CAAGAAGGCCGGGGCGCAAAGGGATGCTCTGATGCTTGAGTCAAAACAGCAGAAGATTGACCAGCTCAGAAATCAGCTTT

CAAGCATGAAAGCCAAAAAGACAAATCGGGGTCTTGTCCTGACATTGGGGAGCGTGCTTTTTGATGTGAACAAGTCCACG

TTGCGGTCCGGAGCGATTCAAAATGTTGACAAGCTTGCCCGGTTCATGAAGAGCGATCCCAAAATAAATGTCATGATTGA

GGGGTACACTGACAACACAGGCAAGCCTGACTATAACAAGCGGCTTTCCCTTCGACGAGCAGAGGCAGTGAGGGATGCAT

TGGTCAGCGATGGAATCAATTCACAGAGAGTTATTACAAAGGGATTCGGGTCAGAGTATCCGGTTTCATCGAACAAGACG

GAGGCCGGTCGTCAGGAAAATCGCCGTGTAGAGATTGTCGTTTCTGATGAAAACGGAAATTTTCCAAAATCAAGA

>yfgL LfeRT32a_3773 Lipoprotein YfgL. Outer membrane assembly processes.

ATGAGACCGTTTTCCCGGGTATTCCTGATGGCTGGTCTCTTGGCTCTGGGAACGCTTTCTTTTGGATTGATGGATCTTGC

CCGGGCTTCTTCCCCGCTTGGTTTCCCCGGAAACAACCCTTCACCGTCAATGAACGAGGCGGCTTCGGAAGGGGATGGCG

GGTCGGTTGTTCCCCTTCCTCCGGAGATCTGGCCGGGAGAGTCTGCCCTCCCTTCCCACAATCCTGTTTTTGTTTCCACG

GTTCCGCTATCGGAGTGGTCCCGGCAATTTGCCCGGGATGGCGTTCCCGAGGACTTTTTCTCCGGAGTTGTCACCGCATC

CGGCAGGATTTTTGTGGCATCCCGATCGGGGCACATAGAGGCCCTCGCGGCTTCTTCGGGAAAATCGCTCTGGCACGTCC

GCCTGGAAGCGCCTGTGAATACTCCGATGATCCTTTCCAAAAAGACCCTCTATGTTGTCTCCTCGACCCCCTCCATCACT

CTTGGCCATCTGCTTTTTTATACCAGGACCAGGCATCTTCTGCGAGGAGATGGTTCTGAGCGGATCTGGGCCCTTTCCCA

GCAGTCGGGCCGGGTGATCTGGTCGAAGGGGCTTCCGGGAGCGGTTCTGGGAAGCCCTCTCCTGGAGCCCCATACCATTG

CCCTTGCGACAGGGGCCGGTCATCTTGTTTTCCTGTCTCGTCAGGATGGCCACATGGTTGTGGATCTTCCTGTCTCGGAA

GGTTCATTCGGATGGGCCTCCCCCCTCGATCGACCGGATGCGATCTATCTGGCACAGGAGAATCCTCCGATGATCGACAG

AGTGGGAAAATCTCCCCTGAAAACCGATTGGCGGCTTGCCTTGTCCAACACCAGGACCTATGACCGATTTTTTATCGGCG

GACCGGTGTCGACCTCTGCGGGTATTGCCGGTGTTTTTCGGGAGAGGGGAACAGGGAGGATGATCCTGGTCTCGATCGAC

GGGGTTACCGGCAAAATCCTCTGGACCCGTTCTCTTTCGGAGACATCGCCAGAAACTCCGGAAGAGATGGTCAGCATGGT

TTCCTCCGACGGCATTTTGTATGTCCCGGTTCCGGAGAAGGGGGATCTCTTCGCTGTTTCCGAAAAGGGTGAGCTGCTCT

GGAAAACCCATGTCGGGGACCATCCCCACACGGGGGGGACCGCCGTCGGTCGCCTCCTTTTTCTGCCACTGCCCTCTGGC

CATATTGCCGCGCTTGATCGGATGAATGGAGAGATCCTTCATGTATGGGCGGGGAAGGCACCTCTCCCGCCCCGCTCTCT

TCCTGTGATCGGTTCGATGATCTTTCTTTCCGACAGGAGCGGGGATGTGAGGGCAATGGACATGTCTTCGCTCGGAGAGA

AGGCCGACCTTTTGTCCAGAACTCCTTTTCTGGAGCCACGGAAGACATCGGGCGATGTGGTCCGGGGGATGCCTGATGAG

>orf2983 LfeRT32a_2983 Outer membrane efflux protein

TTGATACGGGCCCTTTTGTTTTTTACCCTCGTATTTCTTTTATTCGGCGAGACAATCCCCGTCCATTCGGATCCGCTTCC

GGAAGATTCAACGCAAAAACAGTTTTTAGCAGGAACCAGGACCATTTCAGTAGAGAGGGCGGTCAGAATGGCTCTTTCGC

AAAATACGTCCACACGATCCCTGCGTTCGTCCTGGCAGGCGAGAACGTTTCTGGAAGAAACCGCAATGGCACCGTCCGAT

CCCATCATTGAATTTCTCTACGGCATTGGTAACCAGGGGACAGGCACTCCTTATGGGTCTGGAACAGGATGGTCCATTTT

CCAGAACATGCTCTTTCCCGGAAAAGCATGGCTCGACCAAAAAATACTGGCGTCCCAAACAGCACTGCGTCACGAAGACT

ATCTCGACAACCGCCGCACTCTCATCAACCAGACAGAAAAAGCCTACTACACAATCCTTCTCGACGAGGAAAGGCTTCGA

GAAAATCTCCGGCTTCTTGACTGGCTCAAACGGGTCCAGAAAATAACACGGATACGGGTTGCGGCAAACAAAGCTCCTCT

CCTCGACTACCTGAGTGCAAAGAACGCGGTCAAACAGGCAACGCTCAGGCGCATTGCCATGACACTCGCACTTGTCTCTG

ACCGGCGCCAGCTCAATACGCTTCTTGGTCTTCCCCTGAATACCCCTTTACAGCTTGAAGAACCCAGGATTCCTCAGAAG

ATTTCCGTCCACTTTTCAAAGACCGCAGATCTCGATTTGTCCCTTGATAAAAAACGACCCGATGTCAAAAAACTCCAGGG

TGCTCTCGCACTCAGCATCAAACAGCTCTCCCGGGCCGAGATGGACTATCTCCCTGACTTTCAGCTCCAGGGTTCAGAGG

GGGATATCGGATGCTACGGATTTTCAAACACAAATTGTTATTCTCTTGGCATCATGTTCAATCTTCCCCTTTTTTTCTCA

CTCAAACAGCAAAAGAAAATTGCTTCGCTCAGGCAAAACGTATCTGCCCAAAAATGGCTTCTGATCTGGAAAAAGTCCCT

GGCTGTAACAGAAGCGCTCAATGCACAGGCCAAAACGGTTGTGGCCTTTAAAATATTCGAAACAAACAGCAGAGAGATCC

TTCCTGATTCTGAGCTCGCTTTTAATCTGGCCCTTTCGGCCTACGAAACACAAAAGATCAGTTTTCTATACCTGATCACC

TCGCTTAACAACTTTCACCAGGCACGATACAACCGTTTCAAAAGCCTGATTAACTACTACAATGCCTTATCCGACCTGGA

GGCCGTTACGGCAAGCTCCCCCTTTCTTGAAAAC

>orf2498 LfeRT32a_2498 Lipoprotein Surface Antigen. Probably involved in machinery assembly

ATGTCAGGGCTTCTTCCTGTGCTTAAAAGGCATGGTTTATTGATTCAGCGGGAGAGGGTGGGGTCTGTTTTCTCCCCGGG

GCTCTTCCGCTTGCAGGTTATCGGAATGGGGCCTCCCGATGAGGATGATGATGCGTTCTTGTCGCGCCTGAAACCAGATG

ATGTCGTTTTCCTGGTCGGTTTTGGGGGTGGAATTTCGAGCCGTGCCCAGAGGGGAGATGTTTTCTTTCTCTCTTCGGTC

TCCACTTTTCAGGAAAAGGATGGCGAGGAATCCCGGCGTTTTTCGGAGGCATCACTGCCTGGCTCATCAATAATGGGGAG

AATCTTGGGATTCCCGCTTGGGACAGGGGTCTCCCTTTTGAAAATTGCAGGAAGTCCCGAAGAAAAAAAAGAGCTTTTCC

GGTTGACAGGCTCTGATGTTTGCGACATGGAAACCGGAGCATGGGAAAGAGTCATTCAGTCGAAGGGGTGCCGGTTTATT

GTGGCAAGAGTCATATCTGATTCAGTTGAAGAAACATTGCCGGAGTTTTTAGGGGATCTCATCCAGAAAAACGGGAAACT

GTCCCTCAGAAAACTGTTGCTTGCGCTTCTTTCACCTCAAAAGCTTCTGGCACTGGTCAAGATCGGTTACTCACAGAGAG

TTGCGAGAAAAGGTCTTGCCGAGCTTGGAAGAAAAATTGGCGTCTTGTTGACGGAAGAATCAAAAGATAAAACCATGGGG

GAAGTTTTTCGGGGCTTTTTTCTTGGAGTCCTGGTGATGCTCTTCCTGGGGAGTTTTTCTGTCAGCGCTTATGCGATTCC

TCTCCCGCCACCTGCATCTTCGTCGGGGCCACCGGGAACCGGGCCTTTTTTGAATCCGGGCAATGGCGAAAGCTTTCCAC

AGTTGCCGATTGGTGTCCCATCTCCGTTTTCTTTCAGTACTCCAACTCAGAATACGGTTGTCATTCCACTTCCTGCTATC

GGTGTCAGCTACAATACGGGGGTAACGGTCGGGACGATTGTTCCCATCCTTTCTGCCTTGCCCAGCGGCCGTATTACTTC

AATCCTTGCACCATCCATCACCTACAATCCTTACCTGGGCACCCAGGTGGGCGCGCGTTATTACCGTTATTACAAAGGCA

ATCTCAAAAGATGGCATCTTATCGGGCTTCAGTCGAACTCTATCTGGAACTTTTATGAGTTTCATTACAGAGACCTTTCC

ATGGGAGATGGTCGATATATTCTTGATATCCGTCTGAAGGATTTCAAGAATCCCGCTGCCCGATTCTATGGCTTGGGGCC

GGACTCCGCTTTTGGAAATCAGTCAAATTACACACTGTCTGAAGCCTCGGCCCATGTGACGGTGGGTGCGAATCTTGACC

ATCAGAAAATCCGGGCCTGGTTTATGGAGCGACTCAGGGAATATGGTGCCTCCCCCGGAGTGATTCCCGGCCTGCCCTAC

TCCGGGACGGTTTTCCCTAATGTCAATGGTCTTTCAAACAATCCTGTGATTATCTCCCATCGGGCGAATGTGACTTATGA

TACCAGGGACAACCACCTGATTCCGTCGACAGGGACCTATGCACGGGCTTTTGCAGAACTTGACCAGAACGAAACGGCCG

GTGAGGTAAGTGTATTCGATCGCTTCAATGCCGAGTATAAAACATGGATACCCCAGGGGGAGAATGATCAGAATGTGCTT

GCGATCAGGGGGATGGTGAACCTGATGAATGGTCCGAACATTCCCTTCTACGCACAAAGCATGCTGGGAGGCGCGTATAC

CCTTGAAGGGTTTGGTACTGGACGTTTCTATGATCTTGATGCGGCCCTTTTTAATGTTGAAGAGCGGATACAGGCTTTTG

ACATGACACTTCTCGGTGTAACCAGTTCCTGGCAAATCGCTCCGTTTTTGGGGGTAGGAGAGGTTTTTCATGATCAGAGG

GAGCTTACAAATCCCTCGCTTTATGCAATCAATCCGGGCGTCGGCTTCCGGGCCCTTGTCGAGCCCAATGTGGTTGGGCG

TCTTGACATGGGTTATTCCACCCAGGCGGGGGTCGTGGAGTATGTGGGAATCGGGTTCCCCTTC

>orf2016 LfeRT32a_2016 Permease of the major faclitator superfamily

TTGCTTATCCTCCTTTTTTTGTCGAATTGCCTGAATTATCTTGACAGGCAACTCTTTCTTTCCCTTCTTCCTGCGATGAA

AAACCTCTACCAACTCAGTGATCCTGTTATTGGTCTTCTTTCCTCCTCCTTTACTTTTGCCTATCTTGCCGCAGCCCCTG

TTGCCGGTTTTTGGGGAACGAGGTTTAAAATGGAAAAGATGCTTGGTGGAGGTATTCTGCTTTTTTCGATCGGGATGCTT

GTTGCCGCCCTGGCTGAGTTCAGGTGGATGCTTTTCCTGGGAAGATTCCTCACCGGCTTCGGTGAGGCGGTTCTGTCAAC

ACTGGGACCTGTCTACCTCTACAATCTTGTCTCTTCTTCGAAAGGAGCAAGGCTTGGGTGGTTTTACCTTGCGATCCCTC

TGGGTTCGGCAATGGGCTTTTTGCTGGGAGGTGCCTTTCTTTCGAGACTGAGCGTGGAACAGATCCTCATGATTCCTGTT

CTTCCCGGATTCCTATTTGCATGGATCTTTATCATGAGGAAAGGGAGCCACTTTCCTGATTCTTTTTATCAGTCTCCTTT

GCAAAAAGATCTTTTCTCGTGGAGGGGGTTTCTGAAAAGGTTTTCCATGGAAGACAGGGCAATGGTCATGATGAGTTTTC

AGGTGCAGACGCTTGTGACCTTTGTTCTTGGCGGGATGGCTGCCTGGATGACTTTTTATTTTATCAGGGTGAAGATGTTT

CCTGTCACGTCGATTGAGTCCTTGACCGGCGGGATGCTTTTGGTTGGAGGAATCACCGGGATGCTTGGAGGAGGGAAGTT

GCTGGACAGGGAAATCAGGATTCATCCGGATAGGCCATTTCATGGACCCACCCTTTTGGGTTTGGGCGCATCTTTGCTGG

GGGTCCTGGTTGTTATTGCCGGAAACTCTCTGTTTGTCTGTGGCGCAGGATTGCTTCTTGCCATCTCCGGAATATTTCTT

GTTTTTGTGCCGTTGAACTGGATTCTTCTTTCTCGTGATGCCCAGGTCTCTCCAGCAGTCTTGATCGGAATGAGTCTTTT

TGTATCTCATCTTCTTGGCGACCTCCTTTCCCCCTCCATTATTGGAGAGGTTTCCCAGCAATGGGGGCTTTCCGTTGCAG

TTGAAGTCACTCTGATCATTCCTTTGCTCTTTGCCCTTGCAATCTATGCGATATATGCGAAAAAGAGGGGCTTC

>orf3233 LfeRT32a_3233 Heat shock DnaJ containing domain protein

GTGAAGGATTATTATCGGGAACGTGCAGCGCTCAGGCGGCGACTTGAGGAGATGGTCTCTGAAAGTCGAAAGGGGATGGA

GTATGCCCTCGAACGCATGTTTCTGGTCCAGCCCGAGTCCTATATACTGTTTGACGAGCGATACTCCGAACTCCTCCGGC

TGTTCAGCTGTCTTTTGAGGGAGCTTGACGATGTAAACTCCTGGGGAGCCCTTCTGCAGATCCAGAGAAGGGCGGGATTT

CTGGAGGAGCGCTTTGAGGATGTGGACTGTGTGTTGTGGAATCGGCCGAGAAAAGCCAGATCGAGGATTGCCTGGAGCCG

ATTCTTTGGTCACGCGACCGGATCGGGAGGTGAGGATGCGGGAGGCTCGTCCGGATCCGGTTCGGGGCTCAGCCACTCCG

AGGCATGCATGGTCCTGGGTGTCGAGGCAACGGCAACACTGGTCGAAATCCGCCATCGTTTCCGGAATCTGGTGAAGAGT

CTCCATCCCGATGTTCGTGATGGAGACCGAAGTTCAGAGGCGGAGATGAGAAAAGTGATTGAAGCGTATAATGTTCTGAA

AAGCATTCTTGGAGAAGAGGCGCGCGGAAGTTCC

>clpB LfeRT32a_3508 ClpB chaperone

ATGAATCTTGAAAAGCTGACGATCAAGTCCCAGGAAGCCTTGCATGCGGCGGTTGATGAGGCAAGGAGACGTGGCAATAC

CCTTGTTGAACCGGTTCATCTTCTCCGGGAGCTTCTGGTCCAGGAAGGGGGTCTGGTCATTCCGCTTCTCGAGAAAATGG

CCACGGACCAGGCATCCCTTAAAGCGAAAACGGAGGAAGCGATCAAGCTTTTGCCCACCGTAACCGGGTCGGGTGCCGGA

TCTGGGCCGTCCCTGTCCCGCTCGACGGGAGACCTTCTCGACCGGGCCCAGGAGGAGGCCAAAACGTTCAGGGATGATTA

TGTGAGTGTCGAGCATATACTCCTGGCGATGACCTCCGGCTCCGGCGCCGAAGCCAGGCTTCTGAAGGGCGCAGGCCTCG

ATCGTGAAAAAATCATGAAGGCGCTTACTGAGGTTCGGGGAAACCAGCGCGTGACCGACCAGAACCCGGAGGACAAGTAT

CAGGCCCTGTCGAAGTTCGGTCGGGATCTGACGGCCATGGCCAAGCAGAACAAGCTTGATCCGGTGATCGGCCGGGACGC

CGAGATCCGCCGGGTTGTCCAGGTCCTTTCCCGGAGAACAAAGAACAATCCTGTCCTCATCGGAGATCCCGGCGTGGGAA

AGACAGCCATCGTGGAGGGACTGGCCCAGAGGATCGTTTCCGGAGATGTTCCTGAAGGATTGAAGGACCGGACCATCTTT

GCCCTCGACCTGGGGGCGCTTCTGGCCGGTGCCAAGTACCGTGGAGACTTTGAGGAACGGCTGAAGGCGGTTCTGAAGGA

GGTGAGCGGATCGGATGGCCGGATTATCCTGTTCATCGACGAGCTCCACACCATCGTGCGCGCCGGAGCGACCGAGGGTG

GGGCGATGGATGCATCGAATCTTCTGAAGCCGGCATTGGCGAGGGGAGAACTTCGGGCAATCGGCGCCACAACCCTTGAC

GAGTTCCGGGAACATATCGAAAAGGACGCCGCGCTTGAGCGCCGTTTCCAGCCGGTTCTGGTGGGTGAGCCATCCATCGA

GGACACGATTGCGATTCTCCGCGGTCTCAAGGAGAAGTACGAGATCCACCATGGGGTCAGGATCAGGGACTCCGCCATTG

TCGCGGCGGCGGTTCTTTCCCAGCGGTATATTTCCGGTCGTTTCCTTCCCGACAAGGCGATCGACCTGATCGACGAGGCC

GCTTCCCAGCTTCGGGTTGCCATCGACAGCATGCCGAGATCCCTCGACGATGTCGACCGCCGCATTCGCCAGCTCGAAAT

GGAAAAGATGGCTCTTGCCAAAGAGGAAGATCCGGAATCTCTCGGACGCCGTCAGGAGGTTGAAAACGAGCTCGAAGAAA

AAAAATCAGAGCTTGCGCGGCTCAAGGTCCAGTGGGACGGGGAGAAAAAGGCCATCGGGTCGGTCAGGACGCTCAAGCAG

CAGATCGAGGAGGCGAGGCAGGCAATGGAAACCGCGATCCGGGAAGGGTCCTTTGACCGGGCCTCGGAGATCCAGTACAG

CCGCATCCCGAAGCTTGAAAGGGAACTGGCCGATGCCCAGAAGCTTCTTGAGGATCAGGGAAATCGTCTTCTGAAAGAGG

AAGTCGAAGAGGACGACGTTGCGGATGTGATCAGCCGGTGGACTGGAATCCCGGTTTCAAGGATGCTTGAGGGGGAGGTC

CAGAAGCTCCTCCGGATGGAAGAGCGGCTGGGAAGCCGGGTTGTGGGACAGCAGGAGGCGTTGGCCGCGGTGTCGAATGC

CATCAGGCGGGCCCGTGCCGGCATCCAGGACCCGAACCGGCCGCTGGGATCCTTTTTCTTCCTGGGGCCGACAGGTGTCG

GGAAGACCGAGCTGGCAAAAGCGCTGGCGGAGTTTCTCTTCGACAGCGACCAGGCCATGATCAGGATCGACATGTCGGAG

TATATGGAAAAGCATGCCGTGTCAAGACTTGTCGGGGCTCCTCCCGGATATGTCGGATATGAGGAGGGCGGTCAGCTGAC

GGAATCTGTCAGGAGAAAACCTTACTCGGTCATTTTGCTTGACGAGGTGGAGAAAGCTCATCCCGATGTCTTCAACATTC

TTCTCCAGGTCCTCGATGACGGTCGTCTTACCGACGGACAGGGACGCGTGGTCGACTTCAGGAACACGGTGATCATCATG

ACCTCGAACGTCGGATCGGACATTATTCGCCAACGGGTGGGTCTCGATGCGGACGAGACAAAGCAGGAAGTTCTGGAGAT

CCTGTCGAAGACGTTTCGTCCGGAGTTTTTAAACAGGATCGATGAAATCATCGTCTTCCATCCGCTTCTGAGACACGAGA

TTGGACGAATTGTCCGGATCCAGCTTGACGGCTTGAATGCCCGCCTGAAAGAACAGGGGATCCAGCTCGAAATCTCCCCT

GCGGCGGAAGAGGAGCTGACCGCTGTGGGCTATGATCCTGTCTATGGGGCAAGACCGCTGAAAAGAGCGGTTCAGTCCCT

GATTTTAAATCCCCTTGCGCAGAAGATCCTCTCCGGAGCGATCCCGGAGGGTTCTCTGGTGGCGGTGGACTTTGACGGGA

TCGACTTTATCTTCAGGCCCCAGGGCGCA

>clpC LfeRT32a_2328 ATP protease with chaperon activity

ATGATGGACAAATTTACAGAAAAAGGCCGTAAAGTCATCATTATGGCAAGGGAAGAGGCCGAGAAACACCAAAATGATTA

CCTCGGCACAGAACATATTGTCTTGGCACTTGTGCGTGAACAGGATGGAATACCGGTATCTGTCCTCAAAAGGATGGGAT

TGACCCCCGAGCAGATCCGCATGGAGATCGAGCGGAATCTTCTGGGCGGGACATCGACTCTTACCTTTGGGGAGCTTCCG

CTGACTCCACGGGTCAAAAAAGTGATCGAGTATGCGGTTGATGAAGCCCGGTTGCTTGGCCATACCCACATAGGGAGCGA

ACACCTTCTTTTGGGTGTTCTCCGGGAAGAGGATGGTATCGGCGGCAAAATACTGAGAGCGCTGGGCGCCAATCTGATGG

CTGCAAGGCAGCTGACCGCAAGTCTCCTGAAACGTGCAAATTCCGGTTCGGCCAAGGAAAAAGAGCGCAAGAGCAATACG

CCTGCTCTTGATGACTTTGGTCGGGACCTGACGCAGATGGCTGTGGAAAATCAGCTTGATCCCGTTATTGGCAGGACCGA

GGAAATCGAAAGGGTTCTTCAGATTCTGGGTCGCAGGGGGAAAAACAACCCGATCCTGATCGGAGAGTCCGGCGTTGGGA

AAACGGCCATCGTAGAAGGCCTGGCCCAGAAAATCATCGCGGGTGAGGTTCCCGACAACCTTTTGAACAAGAGGGTCATC

GCTCTTGACCTGGGCTCCCTCGTCGCAGGAACCAAGTATCGCGGACAGTTCGAGGAAAGGCTCAAGGTCGTCATGAAGGA

AGTGGTGACGGCAGGGAACATCATCATTTTCATCGATGAACTCCACACTTTGGTAGGGGCGGGTGCCGCAGAAGGCTCGA

TCGATGCCTCCAACATGCTGAAGCCGGCCTTGTCCAGAGGGGAAATCCAGTGCATTGGAGCCACCACCCTTGACGAATAC

AGAAAGTATATCGAAAAGGACGGGGCCCTGAAGCGCCGCTTCCAGCCCATCCAGGTGAACGAACCTCCTGTCGAGGAGGC

CGAGCGCATTATCATGGGGCTCAGGGACCGCTATGAAGAGCACCACGGCGTCCAGATCACGGATGCGGCGATCCATGATG

CGGTGACACTGGCGCAGCGGTATGTCACGGACCGTTTTTTGCCAGACAAGGCTATCGATATCATCGACGAAGCGGGATCA

AGGGCCAAGCTCAAGAGTTTTTCCCTTCCGGAGGAACTCAAGGGGCTTGACCAGGAGCTCAAACGCGTCATCAGGGACAA

GGAAAAAGCCATTCGCGTCCAAAACTTCGAGGAAGCGGTCAGGCTTCGCTCCGAAGAGGACACGCTCAAAAAGCAGGCCG

AAGAGGAAAAGCTTCGCTGGAAAACAGAGCAGGAGCAGAACAAGCCTGTGATCGGGCCTGAGGAGATCTCCGTCATTGTT

TCAAAAATGACAGGAATCCCCCTCTACAAGATCGAAGAGCAGGAAAGTCAGAAGCTTCTGAAGCTCGAGTCCGAGATGCA

TCAGAGAATTGTCGGCCAGGACGAGGCTATTTCCGCCATTGCCCGTTCGATCAGGCGTTCCCGTGCAGGAATCAAGGGAG

AGAAGCGTCCAATCGGCTCATTTATTTTCCTGGGTCCGACCGGAGTGGGGAAGACCGAGCTTGCCCGGACACTTGCCGAA

ACCCTTTTCGGAAACGAAGAGGCGCTTATCCGCGTGGACATGTCGGAATATATGGAACGCTTCAACGTTTCAAGGCTGAC

AGGCGCACCTCCCGGATATGTCGGCTACGAAGAGGGCGGACAACTGACGGAAAGGGTGAGACGCAGACCGTACTCCGTCG

TCTTGTTCGACGAGATCGAGAAGGCTCATCCGGATCTTTTCAATGTTCTTCTGCAGATTCTCGATGACGGTTATATTACG

GACAGCCTTGGAAGAAAGATCGACTTCAAGAATACCGTCCTGATCATGACCTCAAACCTTGGTGCCCGTTCGATTGAAAA

AGAAGGCAGTCTCGGATTCCAGAGAGAGGGGCTCGATTCCAAGGAGAAGTTTGTCAAGAATTCGATTCAGGAAGAGCTTA

AAAGGACGTTCAATCCCGAGTTCTTAAACAGGATTGACGACATCGTCGTCTTCCACCCGTTGACGGAAGTTCATCTTGAA

GCCATTGTCGATCTGAGGATTGAAGATCTGAACAAACGGCTTGTTGAAAAAGGAATCTCGGTCACCTTGACTCCTGAGGC

CAGAAAATGGCTCGTCAAGGAAGGATTCGAGGCCCATTACGGTGCAAGGCCCATGAGAAGGGCGATCCAGCGTCATATCG

AGGACAGGCTCTCGGAAATGATCATTTCCGGGCAGTTCAAGGATCCCAAGAAGATCAGGGTAGAGCTCAGGGAAGGACAC

CCTGTCTTTATCGAAGAAGACATGATGGCACAGGTCGGC

>gcp LfeRT32a_2330 ATP protease with chaperon activity

TTGAAAGTTCTTGGTATAGAAACATCCTGTGACGATACTTCTGTGGCCCTGGTCGATGAGACCGGGGCCACTTTGTTTCT

TGAGACGCTTGGCCAGGAAACCCTCCACGAACGCTATGGCGGCGTCGTGCCTGAACTCGCCTCACGAGCCCACGCACAGA

TCCTCCCGAATCTCGTCAGCAAAGCCTTTCAGGAAACCGGAACGGGTCTCTCCGATCTTTCCGCCATTGCCGTCACCCGG

GGTCCTGGACTTTCCGGATCACTTCTGGTCGGTGTCTCCCATGCGAAAGGTCTCGCTTTTTCCCTGAAAACACCCATTGT

TGCAGTCCATCATGTGGTGGCCCATCTCCGGGGAGCGTTCGGAGATGTTTCCGATCTTCGCGGAAAAACGTTGGGTCTTG

TCGTTTCCGGAGGGCATACGCACCTCTACGATGTCACCTGCTGGCCCAAGCTGAAGGGGATTTCAAAAACGGTCGATGAT

GCAGCGGGGGAAACATTCGACAAGGGAGGAAAGCTCATGGGGCTTCCTTTTCCCGGGGGACCGGCCATTGAACGGATGGC

GAGGGAGAATACCCGTCCGACCTTTCCGTTGACGAAAGGTCCCATCCGGACGGAGGACCCCCTGAACTTCAGTTTCAGCG

GCATGAAAACGGCCTTTTCCCTTAAGCTCAGACAGGCCCTGAATGGCCAGGAGGGGATGCTTCCCCCGGAGGATCTTCCT

CTCTGGGCGGACTCCTTGCAGACAGCCATTCTCGACCATCTGTTCAGTCGGATCACCAAAGTGATCGAGTCGGAATCTCC

CGAAACATTCCTTTTGGGAGGCGGTGTTGCGATCAACAAGGTCCTTCGTGAACGCATGGGAGCATTGTGCCAGAAAAAAG

GAATAAACCTCCTGATGGCTCCTCCCAGGCTTTGTGGAGACAATGCGCTATCCATCGCACTGATCGGTCTTGATTCCATA

AAGCAGGGGATCTTTACGCCGTTCCCCTATCGGGACCTTTCTGTTCAGGCCAGATGGGACCCATCCCTC

>grpE LfeRT32a_1255 GrpE chaperon

ATGGAAGAGAATTTTCGTGAGGACAATCCATCCGGCAAGGATTCTCCGGAGGCAGCAAATCGCCCGGAAGATCCTGTGGG

CGATGTCTCCCAGGCTGCTCCTGCCGAGCAGGAAAACTGGAAGGAGCAGTACGTCAGGCTTCTGGCCGATTTTGACAACT

ACAGAAAGCGGACCGCCAGGGACAGCGAAGAGTCCAGAAAATTTGCAAATGAGTCTCTTCTCTCCGCTTTTTTGCCGGTG

CTCGACAACCTTGACCGTGCTCTGCACCATTTTGAGAAGCATCAGTCGACCGGGGGGGAGACCGATTCCCTGATCGAAGG

CGTTCGGCTGACCCAGAAACAGTTCGGAGAGCTTCTTGAGAAGTATCATGTGACAAAAGTTCCGACCCAGGGTGAGCTCT

TTGACCCGAATGTCCATGAAGCGATGGGATATGCGGAGACGCCGGACGTGCCGGAAGGAACGATCGTTGATGTGTACCAG

CAGGGCTACCTCCTGCACAATCGTTTGTTGAGGCCAAGCCTTGTGACACTGGCAAGGAAGCCT

>dnaK LfeRT32a_1256 DnaK chaperon

ATGGCAAAAGTCATCGGAATTGATCTTGGAACGACAAACTCAGTAGTCAGCATCATGGAGGGGGGAGAGCCGGTCGTCAT

TCCCAATCAGGAAGGTTCCCGGATCACACCTTCAGTTGTGGCTTTTACCGACAAGGGGGACATTCTGGTCGGTCAGGTCG

CAAAGCGACAGGCGATCACCAACCCTGAAAACACCATCTTTTCCGTCAAGCGCTTAATCGGACGCAAATTCAATTCCGAC

GAGGTTGCCGATGCGAGAAAGAAGCTGCCCTACAAGATTGTCGCTTCTCCGAACGGAGACGCCCATGTCGAGATTCGTGG

CAGGGTCTACAGCCCGGCTGAAATTTCGGCAAAAATCCTTGGGAAGCTCAAGCAGGCGGCAGAGGACTATCTTGGCGAGA

AGGTAACTGAAGCGGTCATTACCGTTCCTGCTTATTTTAATGACGCCCAGCGGCAGGACACCAAAAATGCCGGACAGATT

GCCGGACTTAATGTCCTGAGGATCATCAACGAGCCAACGGCAGCTTCCCTTGCCTATGGCCTGGACAGCAAGAAAGAAGA

GAAAATCGCCGTCTACGACCTGGGCGGTGGAACTTTTGACATCTCTGTCCTTGAAATCGGTGACGGAATCTTCGAGGTGA

AGTCGACCAACGGAAACACCTTCCTGGGCGGTGACGATTTCGACATGAAGATCATCGACTTCCTGATTTCTGAATTCAAG

AAGGACAACGGAATCGATCTCTCCAAGGACAAGATGGCGCTCCAGCGTTTGAAGGAAGCCGCCGAAAAGGCAAAGATCGA

ACTTTCGACAGCGATGGAGACTGAAGTCAATCTTCCATATATTACCGCTGACCAGACAGGACCCAAGCATCTGGTCCTGA

AGATTTCCCGCTCCAAGCTCGAGCAGCTTGTGGACGATCTGGTCACCGGCTCGATCGAACCTGTCAAAAAGGCAATGAGC

GACGCTGGTCTGACGACATCCCAGATCGACGAGGTCGTTCTTGTGGGCGGACAGACCCGTATGCCCAAGGTTCAGGAGAC

GGTCAAGAAGTTCTTTGGAAAAGAGCCCCATAAAGGTGTGAACCCGGATGAGGTTGTCGCGATCGGTGCCGCCATCCAGG

GCGCGGTCCTGAAAGGGGAGGTCAAGGATGTTCTTCTTCTGGATGTCACCCCTCTCTCACTGGGTCTTGAAACACTGGGT

GGAGTCTTTACAAAGCTGATCGAACGCAATACGACCATCCCCACAAAAAAGAGCCAGGTGTTTACCACCGCCTCCGACAG

TCAGGGATCCGTGTCGATCAAGGTTTACCAGGGCGAGCGGGAAATGGCCGTCGACAACAAGCTCCTGGGTGAGTTTGACC

TCGAAGGGATTCCTCCGGCCCCACGCGGTGTTCCCCAGATTGAGGTGACCTTCGATATCGACTCGAATGGCATTGTCCAT

GTTTCGGCAAGGGACAAGGGAACCGGCAAGGAGCAGTCGATCCGGATCACCGCCCAGGGAGGTCTTTCGAAAGAAGACAT

CGAACGTCTGGTCAAGGAAGCGGAAATGCATGCCGCCGAAGACAAGGCGAAGAGAGAGTCCATCGAGGCGAAGAATAATC

TCGAATCCCTCATCTATTCGACGGAAAAGTCCCTGAACGAGCTTGGGGATACAATCGGCGATGTCGAGCGCGGAACCTAC

AGGGAAGCCATTGAAACGGCCAAGAAGGCGCTTGAATCGACCGATCCCCAGGTTCTGACCGCTGCCCACAAGGAGCTTGA

GACGCATTCCCACAAGATTGCGGAGATGATCTACAAGAAATCCCAGGAGGCTTCGGCACCTCATTCCGATGGCCAGTCCA

CTCCTCCTCCTCCTGGAGGCCCGGAAGTGGTTGACGCAGAGTATGAGGATGTGAAAAAGGACAATCAC

>dnaJ LfeRT32a_1258 DnaJ chaperon

GTGGCGTCCAAGGATTATTACAACATATTGGGAGTGAGCCGTTCGGCGAGCCAGGAGGAGCTTAAAAAGGCTTACCGAAA

GCTGGCGATGCAGTACCATCCGGATCGCAATCCGGGAGACAAGGCCGCAGAGGCCCAGTTTAAGTCCATCAATGAAGCCT

ATGAGATTCTGGGGGATCCGAAAAAAAGGTCCATCTACGACAATGGTGGCTTCTCCGAAGGCTTTGATATGGGAGGTTTT

TCCGGGAGAGGGGCCGGTGGTGGGCATTTTGGAGATGTCTTCGCCGACGTTTTTTCGGAATTTTTTGGAACGGCGCAGTC

CGGTGGTTCCCAGGCCCAGCAGGGAGAGCATATTCTGAGGCAGGTTGAGTTAACCTTTGAGGAAGCTGCTCTCGGACGGG

AAATCTCCGTCAAGGTTCCCCGGTGGGAGTCTTGTTCCCCATGCCAGGGGACAGGAGCCAAGGCCGGAAAAGCGGTCAAG

GTTTGTACGACATGCCGCGGATCGGGCTATGTACGCATTCAGCAGGGATTCTTCGCTGTCCAGAGAGCCTGCACAACCTG

TAATGGAGAGGGTCGCCTCATTACGGATCCTTGTCCTGTCTGTTCCGGCAGGGGAAGACAGTCGGTCGAGCGAACGATTA

CCCGTTCGATTCCGCCCGGTGTCCAGACCGGCATGCGGGTCCGAATCGGAGGAGAAGGCCATGCGGGAAGTCACAATGGC

CCTCCCGGAGATCTTTTCCTCGATATCATCGTACGGCCGCATGACATCTTTTCCCGAGAGGGCGATGATCTGGTCGTCGA

AAAAAATCTTTCATTCATTCAGGCCATTTTTGGCGATGAGGTCGATGTTCCCACCCTTGGAGCGAGCATCAAGCTCAAGG

TGGATCCCGGAACGCAGCCTGGAACGATCAAGCGCTTCAGGGGCAAAGGGCTCGTCAATCCGCAAAACCGCCAGATGGGT

GACCTTCTTGTCAGGCTGAAGGTGGAAATTCCGACGCAGCTTTCGAAAGAGCAAAGAGATCTTCTGGAGAAGTATGCGGA

AATATCCGGTCATGCTGTCCATGACTCCGGACACCATGAAGGCGGAATTTTCTCCAAGGTCAAATCGATGTTCGAA

>groES LfeRT32a_0575 10 kDa chaperonin (Protein Cpn10)

ATGAAGTTCAAGCCGTTGAAAGATCGCGTGTTCGTTAGCTATTCCGAGGAGGCCGAGAAAACACAGGGTGGTCTTTATAT

CCCTGATGCGGCAAAAGAGAAGCCCCAAAAAGGCAAGGTCGAAGGAATCGGCGACGAAGTGAAGTCGGTCAAGGTTGGCG

AAGTTGTTCTTTTCGATAAATATTCCGGTTCAAAGATTACGATGGAAGGGACTGAGTATCTGATCCTTAAGGAAGAGGAT

ATCCTCGGGGTCTTGACTGCC

>groL LfeRT32a_0576 GroEl chaperon

ATGGCAAAGCAGATGAGCTACAGTGAAAGCGCTCGTTCCTCGATTCTTCGTGGTGTGACCGCTTTGGCCGATGCGGTTAA

GGTAACGTTGGGACCAAAGGGCAGAAATGCTGTTATTGAGAAGAAGTTTGGCGCCCCTACCATTACAAAGGATGGCGTGA

CCGTAGCCAAGGAGATCGAACTTAAGGATCCCTTTGAAAATATGGGTGCTCAGCTTGTAAAGGAAGTTGCGTCCAAAACA

AGCGATATCGCTGGCGATGGAACCACAACAGCTACTGTTCTGGCTCACGCAATCTATCGCGAAGGCGTCAAGAACGTTAC

TGCTGGTGCCAATTCGATGGACCTGAAGCGGGGGATTGATCTTGCTGTTCAGGCTGTTATCGCAGAACTGAAGAAAATGT

CCAAGCCGTGCCAGGACAAGAAGGAAATTGCCCAGATTGCAACCATTTCAGCCAATAATGATCCTGAAATTGGCCGCTTG

ATCGCAGATGCAATGGACAAAGTCGGCAAGGACGGCGTTATCACTGTTGAAGAAGCAAAGAGCATGACGACATCTCTTGA

TGTTGTTGAAGGTATGCAGTTTGACAGGGGCTATACCTCCCCCTACTTCATCACTGATCAGGATCGGATGGAAGTGAGTC

TTGAAAATCCATACATCCTGATTCATGAGAAAAAAATCAGCAGCATGAAGGATCTTCTCCCGATCCTCGAGCAGACCGCC

AAGATGGGCAAGCCAATCCTGATCATTGCAGAAGAAGTTGAAGGCGAGGCACTTGCAACGCTCGTTGTGAACAAGCTTCG

TGGCACACTTCAGGTTGCCGCAGTCAAGGCCCCTGGTTTTGGTGATCGCAGAAAGGCCATGCTTGAAGATATCGCAGCAC

TGACCGGTGGTACAATGATTGCCGAAGATCTTGGCATCAAGCTCGAGAGCATCAAGCTCACCGATCTCGGCCGTGCAAAG

AGAGTGACCATCGACAAGGATAATACAACCATCGTCGAAGGTGCTGGAGAGCACTCCAAGATCCAGGCTCGTGTAAAGCA

GATCAAGAATCAGATTGAAGAGTCCACTTCTGACTATGATCGCGAAAAGCTTCAAGAGCGTCTTGCCAAAATTGTTGGTG

GCGTTGCCGTTATCAATGTCGGTGCCGCAACAGAAACTGAAATGAAAGAGAAGAAAGCCCGTGTTGAAGATGCCCTTCAT

GCAACCAAGGCAGCTGTGGAAGAGGGTATTGTCCCTGGCGGCGGCGTTGCACTTCTTCGTTCAATCGAGGCATTGAACTC

CGTTCAGGCCAGCGGTGACCAGATGATCGGTGTCAACATTATCCGTCGTGCTCTTGAAGAGCCTCTTCGCCAGATTTCCC

AGAATGCAGGGCTTGAGGGTTCCGTTGTTGTTCAGCGTGTCCGCTCTGAGAAGGGGATGATGGGATTTGATGCGGCTTCA

GAGACCTACGTTGACATGATTCAGGCCGGTATCATTGACCCGACAAAGGTTACCAGGTCAGCACTTCAAAATGCGGCATC

TGTTTCAAGCCTGATGCTGACAACAGAAGTCATGATTGCTGATATTCCGGAAAAAGAGCCCAAGGCACCTGCAATGCCAG

GTGGCGGCATGGGTGACATGTAT

>surA LfeRT32a_3243 Chaperon SurA. Prolyl isomerase/chaperon for OMP assembly

ATGACTCTGCATTCTTTCGGAAGAACAGGAATCCTTTTGGCGAACATACGATTTCTTGCAGGGCTTTTTGTCACCGCTCT

CTGCGTCTCCGTGACCGTCCCCGCCCACGGAGAAGAGAAGCCGATTCTCCTTGATTCGGTTCTTGCCGTGGTCAACCACC

ATGTCATTACAAAAAGCCAGATCGACCGTTCCCTCGCCCCGACGTTCAAGAAGCTCCATGCCCAGTATCGCGGTTCAGCT

TACAGGGAGCTCGTCGCATCCCTCGAATACAAGCTCATGATGAAAAAAATCAACGAACAGCTTGAAATGGAAGAGGCTGA

CCGGACCGGACTGACATTGAGCGATGAAGAACTTGACCGGACTATCGACTCGATCATGCAGAAGAACAACTTCACCGCCC

GGTGGCAATTGAAACAGGCACTTTCCGAGCAGGGGATGAGCTACCACCAGTATCGGGAACAGCTCAGAAAACAGATGACG

ATCCTGAAGCTCATCAATACCGAAGTCCGTTCGACGGTTGTCATCAGCCAGGATGAGGTCCGCCAGTACTATCTCGACCA

CCGTGAACAGTTTCGACTGCCCCCCCATGTCACGCTTGCCGACATCTTTCTCAAGATCCCCAAAGACGCAACACCGGCCC

AGATCGCGGCGGTCCGGGCAAAGGGCGAGCATATCCTCCGCCAGATTGCAAGGAAAGACGACTTCGCCATCCTCGCAGGA

TCCGAATCCGAAGGGCCGAATTCGGACAACGGGGGAAATCTGGGTGACCTGACCAAGGACCAGCTCCTTCCGGAGCTCAT

AGGCCCCGCCTTCTCGGTGCCGGTCGGGGGAACCAGCGGGTTGATCCAGACGGACAGGGGTTTCTACATCATCAAGGTTC

TGAACCGGGAAAGTCATCCCTACAAGCGGTTCCGGGACCTAAAGGCCCGCATCCTGGATGACCTTTCAAAGAAGACGACC

AGAAAGAGGCTTCTGACGTGGCTTGAGCAACTTCGGGGCAAATCCTATGTCGTGATCTACATGACCCCTCCATCGGACGA

GAAAACC

>prsA LfeRT32a_3242 Foldase protein PrsA

ATGCTCGTCCTGGCCTCCTGTCATTTCAAGGGACCCAATGACGAAATAGCGGGATCCATCGGGAAAGAACTCATTACTGT

TTCGGACCTTCGTCACACAGCCGCCTTCATCGGCATATCCGACCTGACCAAAGGGGATCCTTCCCTCTGGTCAGGATCCG

TTCGAAAAGCCGTTCTGGCCGAAACCATCCAGGATCAGCTATTGCTCGACTTCGTCAGGGACCGGCGCATTTCCCTGCCT

CCGGAGGAGGTTTCCCTGCTCCCCAAGGACAGAACAATGCGCGAACTCGAACGAAAAAGGATGTTGATCCGGGAAGCAAT

CAGGCGCATCGCTCCCAGGCAGATCGTAACCGACCAGGAAATCAGGACCTATTACCACAACCATATCAAGCGCTTTACCC

TGCCTGAAGAGGCGCTCGTGCGCCATATCGTCACCTCCGACCGGAAGGAAGGCATCGAAATCCAGAAAGCTCTGGAAGGG

GGAGCCTCCTTTTCAGCTCTCGCCAAAGCCAGGTCCCTGGGGGTCGAGGCTTCCACCGGCGGACTGATGGCTCCCTTTTC

AAAAGGGGAGATGCCTCAGCCCTTCGATGAGGTCTTCGATCTCTCCCCCGGAGAGGTCACGGATCTTCTGCCATCCACCT

ATGGGTACCATCTTTTCAAACTCATCAGACTGATCCCCGGATCAGAAAGACCTCTCCGCCAGGTCAGGGGGGAAATCCGG

AAGATCCTCATCGCACGACAGCGCCAGAAGCTCCTCGGCTCCTGGCTGTCTGAACAGGAGATCAAAAAACCCTGGAAACC

TGCCCGGCACTACAGGAAGATTTTTTCAGTCGATCTGGAA

>hsp LfeRT32a_3200 Small heat shock protein Hsp20

ATGAAGCCACATGATATTGATACTCTTTTTTTGCAGTTGGCCGGAGCCCTTTCAAGGGGACTCACTCCTGAAATCGTTTT

TCGCTCATCCGGAGAGCCCAGGTGGCAGCCGATGACTGATATCTATGAAACCGAAGGCGAGGTTGTCATCAAAATTGAAC

TTGCCGGGGTCCCGAAGGAAGAGATATCGATTGGAATGGATGGCAACCGGTTACTGGTCAGGGGGGTGCGAAAGGATGAG

TCAAAGAGTTATGCACCCCAGAAGAAAAAGAACTATCTTCAGATGGAGATCAACTACGGGGAGTTTGAAAGGGCATTTTT

GTTGCCGGAAGGGCTGGACCCACAGAGTGTCCGTGCTGAATACTCTCTTGGTTTTTTGAGAGTCATTGTGAAAAGGCGCC

CGCCAAGGCAGATTCTTACGGTTCAGGTCACCGACGGGGAGGAAGTGAAGAATGGT

>dipZ LfeRT32a_2968 Thiol disulfide oxidoreductase. Oxidative protein folding pathway

TTGGGTGTGAAAAAAGCCAGTGTACTGGCGATTGTCTTTCTCCTCTTTTTCTGGGGGACCATGGGGCCTGATTTTTCCCG

TGTTTCCTTCGCTTCGGATCTTCCGTCCTTTTCCGGTGCGACGGGATGGATCAACAGCCCTCCCCTGAATGATGCCTCCC

TTCGGGGAAAAGTGGTCCTGATCGATTTTTGGGAGTATACCTGCGTCAACTGCGTCCGGACCTTTCCGGTCCTCAGGCGC

TGGTACCGGACGTACGGGCCGGAAGGGCTGGTCATGATCGGTATTCATACGCCCGAGTTTTCCTTTGCAAAAAAGGAAAA

CAATGTGGCCCGCGCTGTCAGAAAGTACCGTCTGGACTATCCCGTTGCCATCGACAGTGACAGGAGGCTCTGGGACCGAT

TTCACAACCACTACTGGCCGGCACAGTATCTCTTCGACCGGAACGGAAAGCTGATTTTTCATACAATCGGTGAAGGCGAT

GACGAGAGGATGGAGGCCGCTATCCGCCAGGCTATCGGTGCAGGAACGGAGAAGGTCCAAAGTTCTTCCCCGCCGGAATT

TCCTGAATCCATGACTCCCGAGCTTTATGCCGGCACCGGACGTCAGGCTCTTTCCCCTCCTCCGGGTTTTGTTCCCGGGA

AGCCGTATACCTACCCGGATGGTCCGGTTGTCTCCGACAACATCAATCTTTCCGGGCCCTGGATCTCAACGGAAGAGTTT

ATTCGGCCGGTCGTTTCCGGTCATCCCAGATTGACCCTTCCTTATCACGCCTCCGGCGTCAGATGCGTTCTCCGCCGCGA

AACAGGGACAAAACCCGTAAGGATCTTTGTCTTTCAGGACGGAAAACCCCTTCCGAAGGGAATCTCCGGTGAGGATATCC

GTTATGACCGGAAAGGGGATTCCTATCTTCTGCTCCGGGCACCCCGGATGTATACCCTGGTTTCCCACCAGAGTTTTGGT

GGTCATACCCTGACGCTGGTACCGGCAGGTTCGGCGGTCAGGATTTATTCCTTTACCTTTGACCCTTCC

>mrcB LfeRT32a_3376 Penicillin-binding protein 1A. Penicillin-insensitive transglycosylase

ATGAAAAAAAAGGCGTTCTGGTTTTTTCTGGTTATTCTGTTGGCGTTGACCGTTGCAGCGGCCGGTGCCGGCTGGTTTTT

CATGAAAAAAATTGAAGCCGGCGTGCCATCTGTCGCTTTTTTGAAAAGCTTTGTTCCTGTTACAACCAGCCGGATCTATG

ACAGGAATGGAAAGCATATCGGAAGCTTCTACCGGGAGAGACGCGAATATCTTCCCCTGAAGAGTATTCCTCCGCTTGTC

ATTCGTGCTGTTCTTGCTGTTGAGGATGCCCATTTTTACGAGCATGGGGCGCTGGCTTACGGATCGATTATCAGGGCGGC

TGTTTCCGATGTCCTGGCAGGATATCTGCGGGAAGGAGCCAGTACCATCACGCAGCAACTGGCCCGTAATATTTTTCTCA

ATCACAAGAAAAATCTGACGAGAAAGCTTCGGGAAGCCATTCTTTCCTATCGTATCGAAAGGGTATTGACCAAGGATGAG

ATTCTGGAGCTTTATCTGAACCAGATCTATTTCGGGGAGGGGGCTTATGGAGTGCAGGCTGCTTCCGAGGCGTTTTTCGG

AAAGGATATTCACCAGCTGACACTGCCGGAGGCCGCCCTGATGGCTGGCCTGATTCGCTCTCCTGTGGAGTTTTCCCCGT

ATACGCATCCCGGAGCGAGCAAGCGGCGTCAGCTTGTTGCCTTGGAACGAATGGAGAAAGTGGGTTATATCACCCATGAG

GAAATGAAAAAGGCCTATGGCCAGCCACTCGTCTTTCGCCAGAGGATCCAGTCCAGGAACCCGAACGCCTACTTTCTTGA

ATACCTTCGAAAACGCCTCGAGATGACCATGAGTTCGACCCAGCTCTATGGAGGTGGGTTGCGTATTTTTACCTCCCTTG

ACAGCCGGATACAGGATATTGCCCTGCACGCTCTCCGGTCAGGTCTTCGGACCATAGACCGGAGGAAAGGCTTCCGCGGA

GCGATCCGTCACCTTTCTCCAGGCGAACTGCGTGAAGTGGAAAGGGAAACGGTACCACTTTCCACGACGGACAAAGGGGC

CTTCACTCTTGGAGGCAGGGAAGAGGTTACGATCACCAGTGTCGAAGAAAGAGGTGCCTTTTTTTCATGGGAAGGTCACC

CGGGTTTTATTTCGAAGGCAAAAATGGTGTGGGCGGCAAAGGTTCTGAGGGGGCCGGATTTTGACCATGATGCCCGGATC

CTTTCCCCGTTTTCTCCGAAGGATGTCCTGAAGCCCGGAGATGTCGTGATGGCCCATTTGACAGGGTGGATCCCGGTCAG

GAAGGGGTGGGTGATCTCGGCTTCTCTCGATCAGGTGCCACTGATACAGGGTTGTGTTGTGGCAATTGATCCGAAGACCG

GGGGAATTCTTGCCATGGTTGGCGGGTACAGTTTCGGCCGTTCCAAGTTTAATAGGGTTTCTCAGGCCATCCGGCAGCCG

GGGTCATCATTCAAGATGATCGATTATGGATCTGCCCTTGAGCACGGCTATACACCATCATCGATCCTTCATGATGAACC

GATCGTCTTTTCCGACCGGAGCCATCATCGAGTCTGGAGGCCGAAAGACTACGAGCATGATTTTCTGGGACCGGTTCCGA

TGAGAAAGGCCCTTGCGGAGTCCATCAACCTGGCAACCATCAGGATGGTCAGGCGCATTGGTGTGGGGAGTGTCATTTCT

TTTGGCCGGAGGCTGGGGCTTACCACCCCACTCAGTCATGACCTTTCCCTGGCTCTGGGATCATCGGGGGTCCGTCCCAT

TGAGCTCACGGCGGCTTATTCGGTTATCGCCAATGGAGGTATCCGGGAGCCTGTCTATTCAATCAAAAGGGTTGTTAATT

TCAGGAAGACGACCGTATTTGAGCATATTCCCTCTCCGACCAAGGTTTATGATCCTGCCTATGACTATATGCTGACGTCA

ATGCTTCAGAGCGTGATTTCCGAAGGGACAGGAAGGGACGCCCTTGTCCTTCCCCGGCTTCTGGCGGGAAAAACGGGGAC

GACCAATGATTTCAAGGATGCCTGGTTCATTGGTTTTTCTCCGGATATAGCCGTTGGGGTCTACGTTGGAATGGATGATC

ACCGTTCCATGGGCAGGGGTGAGTTTGGCGCTCATGCGGCTCTTCCGATATGGATTGACATTATGAAGAGGGCGTTGCCT

CTTCTGCCGAATACCCCGTTTTCCGTCCCGGATGATATTGTCAGTGTCCGCATCGATCCTGATACAGGGAGAAGGGCCGC

TGACAACAGCACTCATTTTGTTACGGAGATCTTTCGAAAGGGAGAGTTGCCTCCACGGGATGCGACATCAGGAAAAGCTC

CGGATGCTGACTTTTACAACATTCAGGGAGCGCCC

>mexB LfeRT32a_1958 Multidrug resistance protein MexB

ATGGTGAACTTTTTTATCAGACGACCAATTTTTGCGGCTTCGGTTGCCATTATAATGGTTTTTACCGGCTTGATTTCCTT

CAAGCTTTTGCCCGTTTCGCAGTTCCCGGAGATTACTCCCCCACAGGTGACTGTTTCAGCCAATTATCCGGGCGCTTCGG

CCCAGGTTGTCGCGGATACGGTGACGACTCCGCTTGAGCAACAGATCAGCGGGGTGCCGGGGATGGCCTACATGTCCTCG

ACAAGTTCGAACGACGGAAGCTCGACGATTACCATTACCTTCAATGTCGGGTATCCGATCGATATCGCAGCGGTTGATGT

TCAGAACAGGATTTCCCAGGCGACACCCCAGCTTCCGGCGATCGTGAATCAGGGGGGAATCATCGTCCAGAAGAAAAACC

CCAATTTCGTCCTGATCGTGAATCTCTACTCCCCCGATGGTTCTGTTGACCCGATCACCCTGAGTAATTATGCCTATCTC

CAGATTGTCGATCCTCTGAAAAGACTTTCCGGTGTGAGTGACGCTGTTATTTTTGGAGAAAGGCGCTACTCCATGCGGGT

CTGGCTCAATCCGGACAAGCTTTCCCGGCTGGGTCTGACTGCAGTGGATGTCCAGAACGCAATCGCAGAGCAGAATGTTC

AGGTTGCTGCCGGAAAAATCGGAGACGCGCCAGCGCCTTCCGGAACGATGTTCGACTATCAGGTCAATGCAACGGGAAGG

CTCAGCACTCCGGAGGAATTTGGAAATATTGTCCTGAGGGCAGGGACAGGAAGCAATGCGGCGGTCCGTCTCCGGGATGT

TGCCAGGGTCGAGCTCGGTGCCCTCCAGTACACATCCTCCGCCAACATGGACAAAAAGCCGACGATCATTATCGGTATCT

TCCAGACGCCGGGATCCAATGCTCTGGAGCTTGACAGCCAGGTCAAGTCGAAAATGAAGGAGCTTTCCGCACGGTTCCCC

AGGGGGATCAGCTATTCCATCAAATATGATACGACCATGTTTGTCTCTGCTTCGATGAAAGAGGTTGTCTTTACGCTTCT

TGAGGCGTTGCTTCTTGTGGTGGGAGTCATCTTCCTGTTTCTCCAGAACGTTAGAACCATGATTATTGCCGGTATCGCCA

TTCCCGTCTCCCTGATCGGTACATTGACGATCATGGAGATCGTAGGATTTTCCCTGAACACCGTGAGCCTTCTTGGCATG

GTTCTGGCTATTGGGCTTGTTGTGGACGATGCGATTGTTGTCGTCGAGAACGTCGAGCGCCAGCTTGAGCATGGTCTGGC

TCCGATGGAGGCGGTCCGAAATGCCATGGGTGAAGTGACAGGGCCAATTATTGCAACCTCCGCCGTGTTGGGCGCGGTTT

TTGTCCCGGTGGCCTTTCTCCCGGGTATCACCGGACAGCTTTATCGGCAATTTGCCCTGACGATAGCCATTTCTGTCGGG

CTGTCAGCCTTTAACTCCCTGACCTTGACCCCGGCTCTTTCAGCCTGGCTTCTTCGTTACCAAAAGCCCTCTGAATTGTA

TTTTTTTCGTAAGTTCAACGCGTTTTTTGAATGGGCGAGAGACGGTTATGCCCATATGGTCCGACGGGCAATCGATTACC

GCTGGTATGCCATGGGCGTTTTTCTGGGCGGAATCTTCATGACCTATTTTCTCTTTGTCCGGGTTCCGGAAACATTTCTT

CCGGTGGAAGACCAGGGCTACTTTTTTGTCATTATCCAGTTGCCGGATGGAGCTTCTCTCGAGAGAACCGATGCTGTGAC

GAAAAAGGTTCGGGATATTCTCCTTAAAACGCCGGGAGTCCAGGATGTCGTGTCCATTTCCGGACTGAATTTCCTGACCT

TCGCCAATCAGTCGAACAGTGCGGCCGAGTTTGCGATCCTCAAACCATGGGAGGAAAGGGGAAGCGCCTTGACCGCCTCG

ATGATTGTCAACTCTGTCCGACCCAAGCTTTTCATGATTCCTGAGTCGATCGCACTGAGTTTTGATCCACCCTCTATTCC

AGGTCTTGGCTCCACCGGAGGGTTTGAGTTTGAAGTGGAGGATCTGACGGGAAAGGGAAGCAAGGCTCTTGATGCAGCGA

CCCAGTCGCTTATGGCTGAAGCCCGCAAACAGCCTGAAATTGAAGCCCGTCAGGTTTTTACAACTTTTTCCACCACGACG

CCTCAGTTTGACTATGAGCTTGACCGGAGCAAGGCCAAGCTTCTGGGGCTTTCTCTTCCGGATATTTTCAGCACCCTCCA

GATTTATCTGGGATCCCTCTATGTCAATGACTTCAATCTTTATGGAAGAACGTTCAGGGTGACCCTCCAGGCTGAAAAAG

ATGCCAGGGGGACTCCGGGAGACCTGTCACGCCTGTATGTCAGGAATGTTTCAGGGAACATGATTCCCCTTGATGCACTG

GGACAGCTTACTCCGACCGTAGGCCCTGAAACGGTCAACCATTACAATATCTATGGCTCTGCTCTCATTAATGGCGGTCC

GGCGCCGGGGTTCAGCTCCGGAGATGCGATCAAGGCCATGGAGAGAGCGGCAAAGAAGTCATTGTCTTCAGATTTTGGAT

ATGAGTGGACGGGGATTACCTATCAGGAACTGAAGGTCGGTTCCATCGAAAAGTGGGTGTTCGCCATTTCCCTGGTCTTT

GTCTTTTTGTTTCTGGCGGCCCTTTATGAGAGCTGGTCCATGCCATTCATGGTGATCCTTGTTGTTCCTCTTGCCATTTT

TGGCGCTATGGTCGCGACCTGGCTCAGGGGAAAGCAGGTGGATGTTTATTCTCAGATCGGGTTCGTCATGCTGATTGGTC

TTTCGGCAAAAAATGCCATCTTGATTGTTGAGTTTGCCAAGCGCCTGAGGGAGAGAGGTCTTGGCATTGTTGAGGCGGCA

ATGGAAGCGGGAAGGTTGCGTCTCCGTCCCATTCTGATGACTGCATTCGCCTTTATTCTTGGAGTCGTTCCCCTGATGTT

TGCCTCGGGTGCCGGAGCCGCTAGCCGGCAGTCCATCGGAACGACTGTTTTTGGAGGCATGTTGGCGGCGACGATGTTGA

GCCTCTTCTTCGTTCCTATTTTTTATGCAGTGATCGAAGGCATTCGCGAAAGAGCCGGATTGTCGAAAAACTCTTCTTCG

GAGAAA

>fusC LfeRT32a_0925 Fusaric acid resistance protein

GTGAAGGGGCTGGAGGGTGTTTTTCCCTTTGCCGTCCCGAAATCTTTTCCAGGAGATCTGCTCTTTTCCCTGAAAACCCT

TCTCGCCTCCGGGCTGGCCCTTTACGTTGCCTTTTCCCTGAACCTTCCACAACCTTACTGGGCTTTGGTAACAGTGCTGA

TCATCGCCCAGCCCTATTCGGGAATGATCAAGTCGAAGGCCCTTTATCGCATTATCGGCACGGTCGTGGGTGCCAGTTTT

ATTGTTTTTGTCATGCCTCTTCTGATCAATTCCCCTGAACTTTTTTCCCTTGCGATATGCCTCTGGATCGCTTTTTGCCT

TTTTTTGTCTCTCTACGACGGTTCTCCCAGAAGCTACGGGTTTATCCTGGGGGGGTATACCGCGGCGCTTATCGGGTTTC

CTGCGGTGGATTCTCCCGGGACAATCTTTCTCCTGGCAAGGTCACGTGTTGAAGAAGTGGTTCTGGGTGTTTTGACGACC

TTCCTTGTCCATGAACTTTTCTTTCCCCGGCAGGTTACTCCCCTGTTGCTTTCAAAAATGGATCAGTGGATTTCCCATGT

TGCAAAATGGGGTGGGGAGGAGATTTCCGGAAGGGGAACCCCCGGGTTTCCTCATGGCATTGGTGCGGAAATATCGGCAA

TATCGGCGATGGGGATCCATGCGGCGTACGAATCCCCCGATCCGAGAGCGATCGGGTGGCTTGATGCACTGATGTTCCGC

ATGCGTGATCTGCTTCCTGTTCTTGTCGATCTGGGGATCCACCGAAAGGCTCTTTTGAAGATATCTCCGGAACTTTCGGA

GGAGATGGACATTCTTGGCGGCGAGGTTTCCGACTGGATAACGGGAAAGAGCCATCATTCCTCAAAAGAGATGGATCTGA

TGGTTTCCCGCTGGTTTATGAGCCATGGAGCTCCTTTTAAAATGACGGAGGCCATGGCGATCCCTACGGAGCTGGGATCC

TTGATGGAGCGTCTTTCCGTGCGATTTCACGACTTGATCCTGATCTGGGGAGACTGCAGGCACATGCGCCAGAGGATTGC

TCATGGCGGTGTGGAGGAGCCTCCTCCACCAAAAGGCCGGGTCTCTGTGTTCCGTGACCCGCTCCTCCCTCTTTTGTCGG

CGCTGGCTGTTTCGCTGGCAGTGCTGGCTTCCATCTTCTTGTGGCGGTCTCTGGACTGGCCCGACGGTTTTGCCGCGGCG

ATGATGGCTGCGGTCAGCGGGACATTTTTTGCAGCCATGGATGATCCGTCCGTGGCGATCCTGGACTTTCTGGCCAAGAT

CAACATAGGGTCGGTGGCAGGGCTGTTTACCCTTTTTTTTCTGCTTCCGAAAATGCATGATTTTTGGGGACTGATGTTTG

TCCTGTCGTTGGCACTTCTGCCTGCGGGAGTGATGCTGGCCAGACCGGATGGTCCACTGAAGGTTCTTCCGTTCATGATT

GGCTTCTCGGGACTGATTGCCTTGCAGTCGACGTATCATGCGGATTTTGCCCATGCATGGAATACGGCCATTTCAGAAGC

TGTGGGTGTTTTTCTGGCAGCGCTTTCAACGATTCTGGTCCGGTCCGTTGGTGCTTCCTTTAGCATACGGAGGATCCTGG

CATCGCTTCATGCTGAACTCAGGCAGCTGGCCATGCTTCAGGGAAAGATGGAGCGTGAGGTTTTTGTTGACCGGATGTTT

GACAGGATCGCTCCCCTGATGTCGAGGATGGGGGTCATTGCTCTGGAAGAAAGGAAAAAACTTCCGGACGGACTCTATGA

GATGGTTGTCGGTCTTGACCTGATCCGGTTGGCCGAAATGCGGGAAAAATTGCCATCCGGTCTGGGAGAGCCGCTCTCTC

GCCTCATCCATGCCATCGGAGAGTATTATGATCGTCCGGACCAGACCCGAAAGGCTTCGGTCAAGATCCTGTTTGACGAG

GTCTGGACGGCTGTCGTCACGTCCGTGGATCTGGATGTTGAAGCCGAGACGCTTTCTCTTCTGGCAAGTCTCCGCTGGAC

CCTTGACGGAGGCGGGTTCAGATCAGCACATGGCGGAGCCGTGAGA

>arsA LfeRT32a_3341 Arsenite-activated ATPase ArsA

ATGATGAACTTTCTGACTGGTTCCCCTCAAGTTTTTTTCTTCACGGGAAAAGGGGGTGTCGGCAAGACCTCGCTTTCTTG

CGCCGCATCCGTTTATCTGGCTGGTCTTGGAAAAAAGGTTCTCCTTGTCAGTACTGATCCGGCATCCAATATCGGGCAAG

TTTTCGGGCAGACAATCGGGAACCGGATTATGCCGATCGACGGGGTTGATGGGCTTTTTGCTCTTGAAATTGATCCGGTT

GATGCTGCGAGAAAAGTTCGGGAACGTGTTGTGGGGCCGGTCAGGGGGGTCTTGCCGGATGATGTCGTTCGCGGGATCGA

GGAGCAGTTGTCCGGTGCCTGTACGGTGGAAATTGCCGCTTTCGGAGAATTTACGGAACTTCTGTCCGATGGAAAGATCC

TCTCCGAGTATGATCATGTTTTATTCGATACTGCACCGACAGGACATACGATCCGACTGCTGAGTCTGCCAGGAGCCTGG

AGCAATTTTATCAGAGACAACCCTCAGGGAGCTTCCTGTCTTGGACCTCTCTCCGGACTTGAGGAACAGAAAGAGCAGTA

CGGGGAGACTGTAAAAATTCTTGCCGATCCGGATAAAACCCGGCTCGTTCTTGTTGCCAGGGGGCAGAAATCCTCTCTTC

TGGAGGTCCTGAGAACGGCAACAGAGCTCGGGACGGCCGGATTGTCCAGCGAATATCTTGTTATCAACGGGCTATTGCCG

GAGTCGGAGGTCAAAGACGATCCGTTGGCTTCGGCCGTTTACCAGTCGGAGCTCAGGACCCTGGAAGAAATGGAGCCTTC

TCTTTCAGGATTCAAGGTGGACCGGTTGCCTCTTAAGGGATACAACATCGTGGGAATCGACCGATTGAGGGCACTTTTCT

CTCCGGAGGACGAGCCTGTCGAATGGTCTCCGGCCATTTCTTCCCGGGAGGAGCAGTTGTCTCCTTCTGCCTCGGGACTT

GATCTTGCGGCATTGATCGATGCCCTTTCAGATGATGGTCGCGGGCTTGTCATGATGGTCGGAAAAGGCGGTGTGGGGAA

AACAACCATGGCGGCATCGATCGCCCTGGGGCTTTCCGAAAAAGGGTTCCCGGTCCATTTGACCACGACCGATCCTGCCG

CACACCTTTTGAGAACATTGCCGGATGCTCCTTCTGGGCTCACGATCAGCAGGATTGACCCCGAATCGGAGACCAGAAAA

TACAGGCAGTTGGTGATGGACGAAAAGGGAAAAGGGCTCGACAGAGAAGGTCTGTTGATGCTGGAAGAGGATCTTCGGTC

GCCCTGTACGGAGGAGATTGCGGTATTTCAGGCCTTTTCCAGGGTGATTGAGGAGTCCGATACAAAATTTGTTGTGGTGG

ACACAGCTCCCACCGGTCATACGCTTTTATTGCTCGATGCGACAGGGGCCTATCATCGGGAAGTTCTCCGCTACAGCAAG

GGTGAATCCCAAAAAACGACTCCAATGGCCCGGCTTCAGGATCCGGAAAAGACAAAGATCATTGTTGTCAGTCTTCTTGA

GACCACGCCTGTTCTTGAAGCCTTGTCCCTTGAGGCGGATCTGATTCGGGCTGGAATTGAGCCATGGGGCTGGATTTTCA

ATAACAGTCTTTGTGCAAGCAAAACTCAGTCGCCTTTGTTAAGAAAACGGGCCTTGTCCGAGCAGTTTTTGTGGGATGAG

ACTCGAAAAAAGATCCGGAAACCCGTCGCTCTGGTAGGAATGCAAGAGTCTGAGCCTCTTGGAAAACTGAAGCTTCTTGA

TCTGGCTGGAGAGAAAGGTGCTTTTTCCGGGAGT

>arsD LfeRT32a_3342 Arsenical resistance operon represor

ATGAAAAAGATTGAAGTCTATGATCCCGCACTTTGTTGTTCAACCGGTGTCTGCGGACCTGATGTAGACACCGCTCTTGT

CGGGTTTGCCGCCGATGTCGACAGTCTGAAGAAGAGCGGTGGCGATCTTGTCCGTCTGAACCTTGCCCAGGATCCCTCGG

CATTTGCTGAAAACCCACTGGTCAAGGGGCTTCTTGCCCGCTCCGGTCAAAGCGCCCTTCCGGTTATTTTGGTCGACGGC

GAGATCGCCCTTGTCGGGAGATATCCGACCCGGTCCGAACTTTTTTCGTACGCCGGACTGTCTGGAACAGGTGAAGGCGA

AAGCAAAAAAATTTCCACAAAAGGTTGTTGTGACAACAGCGGGTGCTGT

>pgl LfeRT32a_3611 6-phosphogluconolactonase (pentose phosphate pathway)

ATGACAGCAGGCTCTGATTCAACGCATTCAGCAAAGGATCATCAGGATCCGGGGATGATTCCCTCTTTCAGGGTTTGGTC

CACACCGGATGAGCTGGCGAGGGAAGGTGCTATTTTGTTCAGGGAGCTCCTGAAGAAGGATGTTTTGTCCAGAAATACCT

CCGCTGTTGTCCTGTCAGGGGGAAATTCTCCGCGAGGGCTTTTTGGAGAAATGGGAAAGTTGCTCTCCGTTGGCTGCGAA

GACTGGATTGGAAAGGTTGAATGGTTCGAGGGGGATGAGAGAATGGTCCCACCGACTCACCCCCGCAGCAACTTCAGGAT

GATCAGGGAAACTCTCCTGGATCCACTGGGCATTTCTCCGCACCGGATTCACAGAATCATGGGGGAGGCGTCGGTCATAT

CCGAGGAAGCGTTGAGATACGCTCATGAAATCATGCAGGTCATGGGAGCCCCCGGCCCCCATCTTCCTGTCATGGACTAT

GTTTTTCTGGGGATTGGGCCTGATGGGCATACCGCAAGTCTTTTTCCCGGAACCTTTCCTGAGCTTGAGCACCATCAGCT

TGTTGTGAGCGCTCCGGCGACAAATGAAAGAGAAGCCCGGATCTCGATGGGCTACCATTTGCTGGCCCATGCCCGTCACC

TGATTTTTCTGGTGATGGGAGAGGAGAAAAGGACTGTTCTTGATGATATTCTTCTCAGGATGAAGCCTTCACCGGTCCAG

GAGCTCCTTTTACTCCGTGCGGAATTGGGCCAGGCCGCAACCTTCTGGGTCGACAGGTCGGCGCTTTCTGAAAATCTGGC

TCGTGTGGTCAAGCTAATGTCC

>treS LfeRT32a_1370 Trehalose synthase (Maltose alpha-D-glucosyltransferase)

TTGATCGGGAACAAGGAAGATGCTCTCTGGTACAAGGATGCGATTATTTATGAAATTCCTGTCAAATCTTTTTTTGATTC

AAACAATGATGGCGTGGGTGACCTGAAGGGGCTCACACAAAAGCTCGACTATATCCAGAGCCTTGGTGTGACGGTGATCT

GGCTTTTGCCGATCACGGACTCGCCACTCAGGGATGACGGCTACGATATACGGGATTATTACACCATACACAAGTCATAT

GGTTCGATGCAGGATTTCGAGGAGTTCCTTGCCCAGGCTCACCGTCGGGGGATCCGGGTCATTGCGGAGATGGTCCTCAA

TCATACATCGGACACTCATCCATGGTTTGTTTCGGCGCGGTCCTCCAGAAATTCTCCTTTCCGGGACTATTACGTCTGGA

GCGATACGGTGGAGCGCTATCGGGAAACACGGATCATCTTTGGAGATTCGGAAAAGTCGAACTGGACGTGGGAGCCGAAA

ACACATCAGTACTACTGGCACCGGTTTTTTTACCACCAGCCAGACCTGAACTTTGACAACCCGAAAGTTCAGGAGGAGAT

GCTCAATGTCGTCAAGTTCTGGTTTTCCCTCGGAGTTGATGGTCTCCGGGCCGATGCGGTCCCTTATCTGTACGAGCGTG

AGGGAACATCCTGCGAGAATCTTCCTGAAACACATGCTTTTTTGAAAAGGCTCCGGGACGAAGTCGATCGACTTTTTCCC

GGAAGGATGCTTCTGGCCGAGGCCAATCAATGGCCTCAGGATGTCCTTCATTATTTTGGAAATGAAGACGAATTCCATAT

GGCCTATCACTTTCCCCTGATGCCCCGCCTTTTCATCGCCATCGCGCAGGGAGACAGAAAACCGATTGTCGATATTCTGG

ACCAGACACCCGCAATCCCTGACTCCTGTCAATGGGCAATCTTTCTCCGCAACCATGACGAACTGACCCTTGAGATGGTT

TCGGACCAGGAACGGGATTATCTCTGGTCGACCTATGCATCGGACCACCGGATGCGCCTTAATATCGGAATCAGGAGACG

CCTTTCGCCGCTCATGTCGAATGACAGACGGAAGATCGACCTGATGCAGAGTCTGCTCCTGACTCTCCCGGGGACTCCGA

TCCTTTATTACGGGGACGAGATCGGGATGGGCGACAATATTCACCTCGGAGACCGAAACGGGGTACGGACCCCCATGCAG

TGGTCTTCGGACAGGAACGGCGGATTTTCGTTCGGAGATCCTTCCTCGCTGTTCTTTCCAGCAATCCAGAATGCTGTCTA

TGGTTTTCAGGTGGTCAATGTTGAGGTCCAGACAAAATACCCGACAAGTCCCCTCAACATGCTCAGGCAGATGATCGCGG

TTCGCCAGTCCGTGCGGATCTTCGGGAGGGGAACGATGGAGATTCTTGCTCCCAAAAACAGGAAAATAATGTCCTATCTC

AGGGTGTTTGAGGGGAAAGTGGTTCTGGTGATCAACAACCTGTCCGATTCAACCGAATCGTTATCGATCGATCTCTCCCG

CTATTCCGGCTGGACTCCTGTTGAAATGTTCAGCCAGACTCCTTTTCCGGAAATCTCAAGGCTTCCCTATCATTTTTCCG

TCAGCCCGTACGGCTTTTTCTGGCTTCACCTCTCCCCGCCCGAGAGGCGGGAGAAAAAACGAAACGGGACAGATAGACTC

TCTGCTCCTTTAGCCCAGACGAAAGGA

>jgt LfeRT32a_3721 Amylomaltase 4-alpha-glucanotransferase

ATGTCCGGACCAAACGATTTCATCTCCCCGCACTCCGGAAAAACCCGGGATACGAGAACGACCATGGTGATGGTTCTTCA

TCACCACCAGCCCGCAGGAAACTTCGACCATGTATTCGAGGAGTCGCAAAGAAACTGCTACCGGCCGCTTCTTGAGCTTC

TCGAAGGATTTCCGGATATCCATGTTTCCTACCACCTGACGGGCCCCCTGATCGAATGGATTGAACAGCATGATCCCGAC

TATATCGCCCTTCTTCACCGTCTGGTCGCGAGAAATCAGATCGAGATCGTCGGCGGAGGATTCTTCGAACCCATCCTGGC

GATGCTTCCACCGGATGCCATCAAGGATCAGACCCGTCTCATGGAAGCATGGATCAAAAGGCTGTTCGGGGGGTTCGACG

GAGGCTTCTGGCTGGCCGAGAGGGTCTGGGAAACAGATCTTCCCCTGAGAATTTCCGGATGCAATCTGACACACACCACC

GTCGACGACCACCATTTTCATCTTGCCGGATTCAAGGACGAGGACCTGCATGGCTACTACCGTTCATCCATCGCGGGAGA

AGGTGTCGACCTTTTCCCCATCTCGGCCCATCTTCGCTACCTCATTCCCTTTCAGAGCGTCGAGGGGGCCCTCACATTCC

TCAACAGGGCGGGGGAGGGAAAGGTCCTGACCTATGCCGACGACGCGGAGAAATTCGGCGTCTGGCCTGAAACCTTCAAC

TGGGTCTGGACGGAAGGCTATCTCCGGCGGCTTTTTGAAGCCTTTACAAGGGAATCTTCCTGGTTGCGCATTGCAAGCAT

GAAAGAGATCCGCAGTGAACGGAGCGCAACGGGAATCGCCATGCTCCCCAATGCCTCCTATCCGGAAATGATGGAATGGG

CCATGTCCCCCGGAAGCTCCACCGCCCTTCACAACCTTCTCGAGACTCTTTCCGGATATGGGCTGAGAGACGCCGCCATG

CCCTTTGTGCGCGGAGGAATTTTCGAGCAGTTCATGACAAAGTATCCAGATTCAAGAAGACTTTACAACAGGATGCTCAT

CGCCTCCCGGCTGTTTTCGGAGCGTGTTCCGAAAACAGCTCCCGACCGTGATGAAATCGGACACCATCTCTGGGTTTCCC

AGGGAAATGACGTATACTGGCATGGACTCTTCGGAGGTCTCTACCTGTCCAATCTTCGCCACAAGGCGTTCGACGGAATC

ATCGGGATGGAAAACCTTCTTGAGAAAAAGGGGCTTCTCAGCCCGGGAGAATCTCTCCTCGGAGATTTCGACCAGGATGG

AAGCCCTGACCGGGTTTACTATCAAAAGGGGTATGTTGCATGGCTTTCCATCGCCGGAGGCGGATGTCTCCAGGAAATAG

ACGACCGGCAACACTGCTTTCACCTGACAAACACCCTGACAAGACAGCTTGAGTCCTATCATTTGAAAAGCTCTTCCGGA

CTGAATGACCATCCCACAGACGGAGGATCTCCGGCCATTCTCTCCATTCATGACCGCTTGCCTCCGCCACCCGCCGAAGG

AGAAATCGCCTTTGATCCCAGGCCCAGACGGCCATTTTCCGAGTTCTTGTGTCACAAGGATGCCGGCCTTGCCTCCCTTT

CGGGCCAATCGGGAAAGGATCCCATGGTCCGTGATCTCGGGAAAGCGGTCTGGACGCTTGGGGAATGGGACAGCTCAAAA

GCCTTCCTCGTCACGTCGGAACAAGAGGGAGAGTCTCCATGGAGACTGGCCAAGAACTATCTTTTTGATCCCATGGAGTT

TACCGTCGGCTATCGCCTGACCGAGGGTTCGCTCCCAAAAGACCATCTTCTCGCAATCGAGTTTCCGGTGACACTTCTCG

TCGGCTCCGGCGAAGGAAGGGATCTCTGGATCATGCCGGAAACAGGTCCCTCCCCCACCTCTCCGACCACGTTCGGAGAG

TGGGGAGATCAACAGGCATCGGGGTTCCGGGGCAAGGACAACTGGTCCAGGGCCACCTTTTCCGTTTCCTTTTCAGCACC

GGTCCGTCTTGTCCGGTTTCCCCTCGAAACCGTCTCTCTCTCGGAAAAGGGGCTTGAACGGGTCTACCAGGGAACACTCT

TTCTCATGATGGTCTCTCCGGAGCTTTTGGAATCAGGAGAGGGATTTCGCATTTCTGTTTCATTTGAAAACGGGGAG

>araJ LfeRT32a_1898 Arabinose efflux permease

ATGAAGCCCTGGACTTTTCTCGCACACAGGGAGACCTCTCCTTGGCTGGTCCTCCTCTCCGTCATTGTCGGCGTCGGAAT

GCCTCTTGCCGACACAACGATCACCAATGTCGGACGAGTCTTTATCGTAAGCAGTCTCGGAGTCACAAGCTATGAAGCCG

GATGGCTGACCGCAAGCTACAGCCTGGCCCTTGCGGTCGGCGTTCCCCTGTCGCATCGGCTGAGGGGTTTTTTCGAAGAA

AAGAACCTGTATTCGGGCGCTATTCTGGCGTTCATGCTGGGCTCCCTTTTCATGGCCATGTCCACAAATTTCACCGAAGC

CATGTTCAGCCGGGGACTTGAAGGGCTCTCGGCCGGGATACTGCTCCCCCTTGCCCCGATATTGATCCAGGAATCCTTCC

CGGAAAAAATCCGCCCGGTCGCCATGTCCACATTTGCCATTGCAAGCGCCATCTGGGTGACCCTCGGACCGACAATTGGC

GGATTCATCATCGACAACCAGGGATGGCAATGGGCTTTTGCCGTCAACATCCCCATCGGTTTTTTGGCCATTCTCTTTGC

CCAGCTCTTCCTTTCCAATCACCCCAGACAGGACCCCCGCCAATTTGACGGGACGGGATTCATCATCCTGTCTGCGTCCC

TCGGATTTCTTTTCACCGGATTCATGCGGGCAGAATGGGTCGGATGGCATTCAGACCAAACCGTCTTCTTCCTCGTTGCC

GGGATTCTTTTCTTTTTTCTTTTCTGGATCTGGTCCTTTTTCCATCCGGATCCGATCCTCCCGACCGAGATTCTGAAAAG

CCCTTTATTCGCTGTCATTCTCGCAATCGTATTCCTGCAGGCAACACAGAGCTTCGGAAGGCTCTATCTTCTTGCACCCT

ATCTTGAGAAAAACTACCACTTCATGGCCCACAACGCCGGAGAGCTGATCGCTGTCGGTGCCCTTACGGAAATCCTGATT

TCATTATCTTTCCTGTTTTCCCGGTTCCTGCCGGGAAAGTGGCCCATCCTCCTTGCATCGGGATGCATTCTTGTCTCCAT

TTCCAATATCGATTTTCTTTTTCTTCCTGCAACCAGCTTCAGTCTCTTCTTTATCATAAAGTCCCAGCTCATTTTTGGAG

CCGGTCTGGCCCTGACCCAGATATCGCTGGCCCCTCTTGCGGCCACGATTTTTCCCGCAGACCGCATCCGCGCCGCAACA

ACCTATCTCCTTGTTTTCCAGTTCATTGGAGGTGCGTGGGGAACAATGCTCGGACGCCATCTGGTGCTTCATGTCAAACC

CGTCTTTTCACAGATGCTGCCCCAGCTGTCCTACCGTCCTGATCCCCATGCGTCCGCCATCCTTCCAGACAAGCTTGCCC

AGGCATTTACATCGAATATTATCTTTTACGACCTGGGTCTCATTGGACTCCTTGGAGGAGTCCTCGCAGTCGCACTTGTC

CCGTTCCTCTCGCCCAAAAGACAGGGACAAAAAGACCCACTAGGGAAGCCGGGATTCAGAGAGATGGCTTCCCTTCTCAA

GAAGGAA

>glgA2 LfeRT32a_2098 Glycogen shynthase

TTGAATTCCCGGAAACGTGGTCGGATCGTTCCGTTTCTCGACACTCCGGGTTCCCTTCTTTTTTTAACCTCCGAGATTTC

TCCCTTCATGAAGACAGGTGGCCTGGGCGACTATTCCCAGGCGCTTCCTCCGGCACTTCTGAGAGCGATGGAGGATGGCG

GGGAGGTCAGGGTTTTCTGTCCAGGTTTTGGGGGGGCGGACAAGGACCCAAGACTTGTCCGCGCGGGAGGCCCCCTGAAA

TTTGAGACCCCCTGGGGAGAAAAAACACTCAGGGTCCTGAGGTCGGGGAAGAAGGGGGATGCGGGAGATTCACAGCCTTC

TCCTGTCATCGACTTTCTCCTTGTCGATGACCTTTTTGACCGTAAAGGCCTCTATGGGGAGTCAGGGCAGGATTATCCCG

ACAACTTCATCCGCTATTTCTCCTGGTCACTTGCGGTCTTTGCCTGGATGCGGGAGACCGGGTTTATTCCGGATGTTGTG

CACGGAAATGACTGGCAGACGGGAATGTTTTTCCCTCTTTTAAGACTCAGGTCGATGGAGGATCCTTCGCTTTCCTCTAT

CAGGACAGTCTTTACGATTCATAACCTTGCCTTCAAGGGACTTTTTCCTTTCGAACTCTTTTCTCTGACAGGTTTTCCTG

ACTCATGGGGCGGTTTTGACGGTCTTGAGTATTACGGCGACCTCTCAATGATCAAGGGAGGGATTGTTTTTTCCGATTAT

GTCACGACGGTGAGCCCGACTTACCGAAACGAGGTTCTCTCCGAACCGCTGGGCGCCGGACTTTCGGGGGCCTTGAAATA

TCGGGGAGAGCGTTTTCTTGGGATCCTGAACGGGATCGACAATGATATCTGGAACCCCTCGACGGATCCGATGATCGAGG

TTCCTTTTTCAAGTGCAGAACATTCCGGTAAGGATCTGGACCGAAAATGTCTTCAGGGAAAGGGGAGGTTCGTTCTCCCT

GATGCGCCTGTCATCGGGTGTGTGACAAGAATGACTCCGCAAAAGGGAATCGACCTTTTGCTTGCAGGCATTGAGGAATG

GTTTTCAAGGAAAGGAAGCCCTTTTTCCTTTTTTCTTCTGGGAACGGGCGATCCTGTCCTCGAGAAAAAAGCGCGGGAGC

TTGAGTCGAAATACAGTGGGATGGTCAGGGCGGTGATAGGCTTTGATGAACCTCTTTCTCACCAGATTTATGCTGGATCC

GATTTTTTCATAATGCCGTCACGCTATGAACCATGCGGTCTGGCACAGATGTACGCCATGCGCTATGGATCAGTCCCTCT

GGTTTTTCCCACAGGCGGGCTCAGGGATACTGTCAGTGACGGGGTGGATGGTCTCTGGATGTCGGCTCTTTCCGTTGAGG

GAGTCACCGACATCCTGGATCGTGCCTTGAAGATCTACCGGGATAAAAAGGCCTTCCTCCCGCTGAGAAGACATGCGATG

GAAAAGAACAACAGCTGGGAAGAGCGGACAGGGGAATACCTCAGGCTCTACAAGGGGCTTCCTTCTTCCGTTCCGGGATC

AGGTCAGGATTTGTTGATGGCCCGGATGGCCTTGAAAATG

>glgC LfeRT32a_3719 Glucose-1-phosphate adenylyltransferase

ATGCAAAACAATTATCGTGCACTGGCCATCATTCTTGCCGGAGGCGAAGGGAAAAGACTTTTTCCCCTCACCCGGGACCG

GGTCAAATCCGCCGTTCCTTTTGGAGGGGCTTACCGGATCATCGATTTTGTCCTGTCCAATTTTGTCAATTCCGGACTTT

ACAAAATCAAGGTTCTCACCCAGTACAAATCCCACTCGTTAAACACCCATCTTTCAAGGGGCTGGCGGCTTTCCCCCCTG

CTTGACCAGTATGTCGACCCCGTTCCGGCCCAGATGCGCCGGGGCCCTCACTGGTTCCAGGGAACCGGAGATGCGGTCTA

CCAGAACCTGAATCTGATCCTCGACGACAATCCCGATTTTGTCTGCGTGTTCAGCGGTGACCATATCTTCAAGATGAACA

TCTCCCAGATGATTGAAGCCCATATCCAGAGAAACGCCCAGGTCACCGTCTCCGCCATTCCCGTTCCCATCGAGGAGGCG

TCTTCCTTCGGAATCGTCGGAATGGATTCTTCCGGCATGGCGACCTCTTTCATCGAGAAACCCAAAAATCCGGCGCACAT

GCCCGGCAACCCCCATATGTCCCTTGTGAGCATGGGAAACTATGTCTTCAACACCCGACTCCTGATTGATGTCCTGACCA

AGGATGCCCAGAACGAGAACAGCAATCATGACTTTGGGAAAGACATCCTTCCGAAACTGACCCATGAAGGAATGGTCCAT

GTTTACGATTTCAGCCAGAACACCATTCCAGGGATGACTGAACTCGAACAGGGCTACTGGAAAGATATCGGACAGCTCGA

CGCCTACTGGCAGACGCACATGGACCTTGTCTCCGTCTCGCCGGCCTTCAATCTCTACAATCCGAACTGGATCATCAGGA

CCTATCGCCCGCAGGTGCCTCCGGCCAAGTTCGTCTTTGCCGACGAGGAGAACAGGCGCATCGGGGTGGCAACGGATTCG

ATCGTCTCCGGCGGCTGCATCATCTCGGGAGGACAGATCGACCGATCGATCCTCTCCCCAAGCGTCAGGATCAACAGCTA

TTCCCGTGTCTCGGAATCGATTCTTTTCGACAATGTCGACATCGGACGCTATTCCCGCATCAGCAGGGCGATCATCGAAA

AGGGTGTCAGGATCCCTCCCCATACCGTTATCGGGGAAGATCCCGCCGAAGACAAAAGACGATTTCACGTCTCTGATTCC

GGAGTCGTCGTCGTGACCCGGGAGGACTTTGGCAACCACGAGGGGTCATCG

>glgP LfeRT32a_2323 Glycogen phosphorylase

ATGGTCAGAAAATCAGCTTCAGGAACCATGACCAGCCAGCGTTATACGACCAGACATTCCGATGTTGAGGGGTTCGATTC

GCTTGCGGAGCTTGCCCTTGATATGAGGTGGTCATGGAATCATTCCGCGGATCTGGTCTGGCGCCAGCTCGACCCTGATT

TGTGGGAGCTGACGCACAATCCCTGGGCGGTGCTTCAGACTGTGGCCCGGGACAGGATTCTGGAGGCTTTTTCGGATCCT

TCGTTCAGGAAGGATGTGGCAAGGCTTCTGAAAGAAAAAAAGAAGTCCACGGAAGCACCGGCATGGTTTCAGATAACGTA

TCCGGCTTCCGCCATAACGACAGTTGCCTATTTCAGCATGGAATTCATGTTGAGCGAAGCTCTTCCGATCTATTCCGGGG

GGCTCGGCAACGTCGCTGGCGACCAGCTCAAGGCGGCCAGCGATCTGGGGGTTCCGGTTGTCGGTGTCGGTCTTTTGTAT

CAACAGGGCTACTTTCGCCAAGTCATCGACAAAGAGGGAAATCAACGGGCTCTTTACCCATATAACGACCCGGGCAGTCT

CCCGGTTACCCCCGTTCGTGAGCCGAGCGGCGAGTGGCTCCGGTTGTCCATTGACATGCCGTCGGCAAAAATGTGGCTCA

GGGTCTGGCAAGTCAAGGTCGGAAGGGTGAGCCTGTACCTGCTGGACTCGAACGATCTGGCCAACCACCCCTCCCATCGC

CAGGTGACCAGCGAGTTGTATGGCGGAGGGGCCTCTCTCAGGCTGAAACAGGAAATTGTTCTTGGTATCGGTGGCTGGAG

ACTGCTTCGAAAGCTTGGAATTGATGCAAATGTTTGCCATTTGAATGAAGGCCATGCGGCCTTTGCCATCCTGGAACGTG

CCAGGGACCAGATGGAAAAGACGGGCCAATCTTTTGACGAAGCTCTTTCGACAACCAGGGCAGGCAATCTTTTTACGACG

CACACCGCCGTATCGGCGGGTTTCGATCGATTCGAACCATCCTTGATGGACCGCTACTTCAGAAAGTATTCGGAAAACGA

ACTGAAGATCGGTTTTGATGAATTGATGTCTTTGGGCAGGGTTCATCCTGAAGCGACCGACTCCCTGTTCAATATGGCAC

ATCTTGCCATGCGGGGAAGTGGGCAGGTGAACGGTGTCAGCGCCCTTCATGGTCGCGTCAGCCGGCATATTCTCGGGGAT

CTTTTCCCCAGATGGCCGACTCCTGAGGTCCCGGTCGGACATGTGACCAATGGTGTTCATGCTCCCAGCTGGGATTCCGA

GGGCGCGGACGCTCTCTGGAGCAATTCCTGCGGCAAGGACCGGTGGCTTTTGCCGACAGAATCCCTGGAAAGCGATATCC

ATCAGCTTTCCGATGAGGTCTTGTGGCAGTTCCGAAATGAATCCAGGGCAACCTTCATTACTTACGCCAGAAATCATCTT

GCTCGCCAGTTGCGTGTTTCCGGCGGGTCTTCCCGACAGATTGAACAGATATCCTCTTTTTTCTCGCCGGATGCTCTGAC

AATCGGATTTGCCCGGAGATTTGCAACCTATAAACGCCCCAATCTCCTTTTGTTCGATCCTGACCGCCTGATTCGTCTCC

TGCATCATCCAAAAGGCCCTGTTCAGCTGGTCATTGCCGGAAAGGCGCATCCCGCGGATCTGGCCGGCCAGGAAATGGTC

CGGCAATGGATTTTGTTTACGAGGAGGCCTGAGATTTCCGGTCGGGTCATCTTCCTCTCGGACTATGACATGCTGCTGAG

CGAGAGGCTTGTTCAGGGTGTCGACCTCTGGCTGAATACTCCGCGAAGACCATGGGAAGCTTGCGGGACAAGCGGAATGA

AGGTCCTGGTCAACGGAGGCCTGAACTTTTCCGAGCTTGACGGATGGTGGGCTGAAGCCTATTCGCCCAAGGTCGGCTGG

GCGCTTGGCGATGGTCTTGAGCATGGAGATGATCCGGGATGGGACCATGTGGAGGCTGAAGCTCTTTATGAAAAGCTCGA

ACAGGAAGTTGTTCCGGCATTCTACGAACGCGACAGGAATAACCGTCCGAAGGGCTGGATCGAACGAATCCGCGAAAGCA

TGGCTTGCCTGACACCACATTTTTCAAGTAACCGGGCGGTCAGGGAGTATACAGAGACGTACTATCTTCCTGCGGCGGAG

AGAGTTCAGTTGAGATCCCGGGAAAATGGAGTTCTCGGAACCCGTATCTCTGCATGGAGGAAAAAGATTTCGGAACACTG

GAACACCCTTGGAATCGAGAGTGTGTCGGTGTCCAATGGAGAGAATCTTTTTATTTTTTCCGTGTCGATCGATCCCGGAA

AGATGGACCCGTCGCACTTTTCGGTGGAGTTATATGCTGATGGCAATCAGGATTTGCCAATGTTCAGACAAACGATGGAG

AGGTCTCCCGAGAACGCTGCCGGCAAAACGCTCTATACACTGTCTGTTCCCGTTCATCGCCCACCGGGAGATTATACAGC

CCGTGTTGTTCCCTGTTTCCCGGGAGTCTCTGTCCCGCTGGAAGCTTCGCATATCCTGTGGCAAAAA

>rfbC LfeRT32a_1231 dTDP-L-rhamnose synthase

ATGAAAGTGATAGAGACCACACTTCCGGGGGTTCTTCGGGTCATCCCTGACCTGATCAGGGATGACCGGGGATTTTTCAT

GGAGACATACCATGCCGGGAAGTTCAAGGCACTGGGACTTCCGGAAATTTTTTTGCAGGACAACCATTCCCGTTCAGGAA

AGGGGATCCTGAGAGGGCTTCATTTTCAAAAGACTCGCCAGCAGGGAAAGCTTGTCCGGGTTGTTGCCGGGTCCCTCTTT

GATGTGGCGGTGGACATACGTCAGGGGTCTGCGACCTTTGGTCGATGGTATGGACAAGTTCTCACCGCAGAATCCCCGGA

ATTTTTGTATATTCCTGAAGGTTTTGCTCATGGATTTTATTCCCTTTCCGATAATACGGAACTTGTTTACAAGTGTACGG

AACTTTATGATCCATCCGATGAGGGGGGCATCCTCTGGAGCGATCCGGATATTGCGATCAAGTGGCCAGGAGAAACGCCA

ACAATTTCACAAAAAGATGCTTTGTACCCGTTTTTACGCAACCTGTCTCCAGAGAATCTTCCCAGACATAATCCAGCC

>rfbF LfeRT32a_1415 Glucose-1-phosphate cytidylyltransferase

ATGAAGGTCGTCATTCTGGCCGGAGGAATGGGGACCAGGATCAGCGAGGAAACATCCGTCCGCCCCAAACCGATGGTCGA

AATCGGAGGGAAACCCATTCTCTGGCATATCATGAAGATATATTCCTCCTATGGTCTTCATGACTTCATCATTTGTCTGG

GCTACAAGGGGTATGTCATTAAAGAGTACTTTGCCAACTATTTTCTTCACATGTCCGATGTGACCATCGACATGTCCGAG

AACAGGATGGAGGTTCACCAGAACAGTGCAGAGCCCTGGCGCGTCACCCTGGTCGACACGGGCGAGAGCACGATGACCGG

CGGACGCATCAAACGGGTTGAGAGATATCTTGATAACGATGATTTTTGCTGCACCTATGGAGACGGCGTTGGTGACGTGA

ACATCGCTCGCCTGATCGAGTTTCACAAAAAAAATGGAACATTGGCCACGCTGACCGCCACCAAGCCTCCTGGACGATTC

GGATCGATCAACATGAGCGAGAACAAGATCGTCAACTTTCAGGAAAAGCCCGAAGGAGACGGTGCCTGGATCAATGGGGG

GTTCTTCATTCTCAGCCCAAAAGTTCTCGACTATATCGAAGGCGACGAGACGATCTGGGAGCGGGCCCCGATGGAACGGC

TTGCAAAGGAAGGTCAGATCTCGGCTTACATGCACGAGGGATTCTGGCAGCCGATGGATACCTTGCGCGACAAGACCCAT

CTCGAAGAGCTGTGGTCTTCCGGCAAGGCTCCCTGGAAGATGTGG

>poxB LfeRT32a_2281 Pyruvate oxidase-deshydrogenase (Ubiquinone)

TTGTCCAGGACAGTGAGCGACCGTATCGTTGAGTTTCTGGCAGAACGGGGTGTTCGAGAGATCTTCGGCATCCCGGGGGA

TACCATCGACTCCCTGATGGAATCTCTCCGGAAACAGAGTGATGTCCATTTTGTCGTCATGCGCCATGAAGAGGCCGGGG

CTTTTGCCGCCAGTGCCCAAGGCAAGCTTTCGGGAAACCTGGCCGTCTGCGTTGGCTGTCAGGGACCGGGAGCGATCCAT

CTTTTAAACGGACTCTACGATGCTGCGCTGGATCACGCTCCGGTTCTTGCCATCACCGGCCAGATTCCCCGGGATCAGAT

CGGCACCGGACGCCCCCAGGAGATCGACCAGCTCCGTCTGTTTGGAGATGTGGCAATCTACAACCAGGAGGTCCGTTCTC

CTGAAAATCTTGAGGCTGTTCTTTCCCAGGCGGTGCGACAGGCTCTCAGTTTGCGGGGGGTGGCTCATCTGTCGATTCCA

TCGGATGTCATGCGGCTTCCTGCTCCACCACGGCCCCCCGGAGTCTCAAAAGACCGTTTCGAATTCCACTCAATTACCCG

GCCTCCCCTGTCCGAGATCCTGCGCGCGGGAGAGATTCTGGACAGGGCGTCACGCGTTACGATCCTCTATGGCGAAGGGG

CACGGCGGGCCGAAGCGCCTCTTCTGGCGTTGGCATGGCGTCTGGGTGCACCTCTCGTTCATACCACCCGATCGAAAGAC

ATCCTCCCTTCCCGCCATCCTTCTGTCATGGGCGGAATTGGCCTGATGGGCTCCCGGGCGGCCAACCATGCCGTCCAGAA

CTCCGATGCCCTTCTGGTGGTTGGATCGGATTTTGCCTTCCGGGAGTATTACCCAGAAGACATCCCCATTGTTCAGGTCG

AGATCGATCCATCCCGGATCGGGCGTCGGGTTCCTATTGAGGTGGGGCTTCTGGGGGAGGCCAGAGAGGTTCTGCCCGCC

CTGGCCGAACAGGTCCACGAACGGACCGACACATCCTTTCTCGACCGGATGAGAAAAGAACGGGAAAAGGAAGTTGCCGG

CCAGGACCGGAGGATGGAAGATGGTCTTCTTCATCCGGGGGATCTCGCAGGGATGATCGGAGACCGAGCACCGGACGATA

CGATTTTTCTGCTCGACGCGGGGACGGTGACCGTCTGGGCCAACAACTATCTCAATATCCGGGGGAGCCAGAGATTTATC

TGGTCGTCGAACCTCGGATCCCTGGGATTTGCACTTCCGGCGGCAATTGGGGCGAAGTTTGCCTACCGTTGCCGTCCGGT

TGTGGCCATGACGGGAGACGGTGGGTTCGGGATGCTTCTGGGAGATCTCGCAACCGCCGTCCATTACCGTCTGCCCTTGG

TGGTTGTTGTCTTTAACAACGGTTCCTACCAGTTCATTGAATACGAGGAGGAGGCCGAAGGGAATCCCGTGTTCGGAACC

AAGCTCACCAATCCGGATTTCGTCGCCCTTTCCCGTGCTTTCGGAGGGGACGGAGCCACTGTCCGAAGCCTGAAAGAGGT

TCCGGAAGCCCTTGATGCCGCTTTCTCCTCCCGGGTTCCCTTCGTGATCGATGCCTTCGTCAACCCGAATGAACTCTATA

TTCCGCCTCTCCTGACTCCCCACATGGTCATGGAGTTTGCCCGCTCCCAGATCCGCAGCTTTTTCGCCAAGCCTTCGGAG

GCCGAAGGA

>ackA LfeRT32a_0606 Acetate kinase. Acetyl-CoA biosynthesis

GTGAAATTGGTTGTGGTCAACCCAGGTTCATCAACAATCAAGCTGTCATTATGGGAGGGCGGTGCAAGAGTTGATTCGTC

GACCGTTCCCTTCCATGACCGGTCCGATCTGCTTGAACAGGCTCTCTCTTCATATTTTCAGGAGATTCCTGGACCCCCTC

CGGACGCTGTCGGTGTCAGGGTCGTTCATGGAGGTCCTGTATTCAGGGATCCGGTCCTGTTTCATGAAAAAGTGCTCAAT

GATCTCAAGCGCCTTCGCCCTCTTTCTCCTTTACATACCGATGGTGCGATCCGGGCCATCAATGCCCTGACCGGACTCTT

TCCGGGTCGTCCTGTTGTGCTTTCTTTCGATACAGCCTTCCATCGCACTCTTCCCCCCGAAGCTTTCCGCTATCCTGTTC

CCGAATCCTGGTACCGGGATCATGAGGTAAGGCGCTACGGCTTTCATGGTCTCTCATACGACTATATAGCCCACCGCCTG

CAAGAGACTGTTCTGGCAGGGTCGATTGAAAGAACCGTTGCCCTTCACCTTGGAAACGGAGCTTCGGCCTGTGCCATCCT

GCGTGGTTTGTCTGTCGACACGACAATGGGGCTGACTCCGATGGAGGGGCTGATGATGGGAACAAGATCCGGCTCTATCG

ATCCCGGGATTCCTTTTTATCTTGAAACGAAAGGGTTGTCGCCCGGGGAGATTGAGCGACAGCTGAACCATGAGTCAGGA

CTTTTGGGCGTTTCCGGCCTTGATCGTGATCTGGCCGTTGTGGAGAAGGCTTTTCATTCAGGAAACAAGTCTGCGGAAAT

GGCGATTTCCATGTTCACAAGAAAAGCGGCGCAGGCGGTTGCCCAGATGGCGACATCGATGGGGGGAATTTCCTGCCTGG

TCTTTGCCGGTGGGATCGGCGAGCATTCCAGTCTGGTCAGAAACTGGATTGCCAGGGACTTAGGCTTTCTGGGGATCGCT

CTCGATTCGGCCAAAAACGCCGAGGAGCAAGCATCTTCCCGTGATCGCATTATTTCGCTTTCTGGATCCCCTGTCCTTGT

GATGATTGTTTCCACCCGGGAAGACTGGACCATTTCCAGGGATGTCCAATTGGTCCTCGGGAGATCTTTC

>acsA LfeRT32a_0742 Acetyl-CoA synthetase

ATGGACAAACCAGACAACAATATGAAAGATCAACAGGAAAGCCTGCACTACCTTCCGGACCCGGACCTTCTTGAGACACT

TCATGTTCCGGATTACCAGTCAATTTATGAGGAATCGATCCATGATCCTGAACGGTTCTGGGGAAAAATCGCCGAAGACT

TCGTCTGGACACGGAAATGGGACCGGGTTCTCGAATGGAACCCGCAAAACTATGAAGCCCGATGGTTCCCCGGAAGTCTT

CTGAACATCACGACCAACATGCTCGACCGGCATATCGAAGAAGGTCTTGCCAACAAAGTCGCTCTCATCGCCATCTCCGA

TGACGGCAATGAGAGGATCTATACATACGGACGCATCATGGACGAAGTCAACCGACTCTGCCACAGCTTTGCTGAAATGG

GTCTTCAGAAGGGAGATCGGGTCACGATTTTTCTTCCACCCACTCCGGAGCAGGTTATTTCCATGATCGCATGTGCCAGA

AGTGGACTGGTCCACACCGTTGTCTTTTCCGGATTTTCCGCCGGGGCATTGAAAAGCCGCATGGAAGACTCGGAACCGAG

ACTTCTCATCACCGCCGACTGCGCCTACCGGAGGGGAAAGCGCATTGCGCTCCTTGACACCGCCAGGGAAGCCAGGGCTG

CCATTTCATCTCTTCTAAAAACCATCGTCATCAGGCGGGAGAATCCGGATCTTGCCCTTGCAGACGACGAGATTCCCATG

GATCTCCTGATGAAAAAGCACGCCTCTTCGGGGTTCTTCAAGGCCGTCGACTGCACAACCGACGATCCACTCTTCATCCT

CTACACGTCGGGAACCACCGGGAAACCCAAGGGGATCGTCCATACCCACCCGGGCTACATGGTGGGAACATTTCTGACCA

CCCGCTGGGTCTTCAATCTCCATCATGATGATGTCTTCTTCTGCGTTGCAGACCCCGGCTGGATCACCGGCCACAGCTAC

ATCGTATACGGACCTCTCCTGAACGGAGCCACCGTTCTCCTCGCCGAAGGCTCACCTGACTACCCGGACCCGGGACGCTG

GTGGCATCTTGTGGAAAAGTATCGTGTTACCGTTTTCTACTCAACCCCAACAGCGATCAGGCTTCAGATGAGGCTGGGAA

AGCAGTGGCCCCAAAAGTACGATCTTTCCTCCCTGAGACTTCTGGGATCGGTGGGAGAGCCCATCAATCCGGAAGCATGG

CTTTGGTTCAGGGATGTCACCGGTGGGCGACTGCCGATCATGGACACCTGGTGGCAGACAGAAACAGGAATGCATATGAT

CACACCCCTTCCGGGCGTCCCTCTTGTCCCTGGATCGGCGACACGACCTTTTCCCGGAGTCATCGCTGATGTGGTCAATC

GCCAGGGGGAAAGTGTTGGCCCCGGAGAAGCTGGCTTCGTCATCATCCGGAAGCCATGGCCCTCCATGTTCCGAACCGTC

TACAAGGATCCGGAACGATACAGAAAGTACTGGGAAGAGATCCCCGGAGTTTATTTTTCGGGTGACTCTGCCAGACGTGA

TTCCAACGGGCTCTTCCACATGATTGGCCGGGTCGATGATGTCATCAAGGTGGCAGGACACCGCCTTGGAACCGCCGAAA

TCGAGTCCGCGCTCGTTTCCCATCCGAGTGTTTCCGAAGCTGCCGTCATCGGAAAACCGGACGACCTCAAGGGAGAGGTC

ATCAAGGCCTTCGTGATCCTGAGAAAAGATGCCGAACGGACCCAGGATCTCGAGCTCAAGATCCGACAGCATGTCAGAGA

GGAGCTCGGAGCTATCGCCATGCCTGATGAAATAGAAATCACTGAATGGTTGCCACGAACACGCTCAGGAAAGATCATGC

GGCGTGTTTTGAAAGCCAGGGAACTTGGGCTTCCGGAAGGAGACACCTCGACACTCGAGGAT

>mdh LfeRT32a_1895 Malate dehydrogenase

TTGGGCCAGAAGAAAAGAAGAACAGTCTCTATTATCGGGGCTGGAAATGTAGGCGCCACAACCGCCCAGAAGATCGTTGA

AAATGGGTTGGCGGATGTCGTCATCCTCGATGTGAGAGAAGGAATTGCCCAGGGCAAGGCTCTTGATATGCTCGAGTCCG

GGCCTTTGCTGGGTTTTGACACCAGAGTGACAGGTTCAGGAGACTACGCTGATATCGAAGGCTCATCGGTTGTTGTTGTC

ACTGCGGGATTTTCACGCAAGCCAGGAATGACGAGAGACGATCTTCTCCATAAAAATGGCGAGATCATGATCGAGATTTC

CGAAAAAATCAAAAAACATGCGCCCGAAGCGATTGTCATTATGGTGACAAACCCCATGGACCTGATGGCTTATACCCTCT

GGAAGGTGACAGGGTTCAAGCGGGAGCGTGTGATCGGAATGGGCGGGGCGCTTGATTCCTCCCGTTTTGCCTACTTTGTC

AGTGAGGTCAGCAAGACATCGGTTTCGAATATCCAGACGCTTGTTATGGGTGGACATGGTGATCTGATGGTGCCGCTTCT

TAATTTCTCGACGATTTCCGGAGTGCCCCTTCCCAAGGTTCTCGATCCCAAGGTCCTTGACGGGCTGGTGGCACGAACCC

GGGACGGTGGCGGCGAGATTGTCCGGCTGATGAAGGATTCCTCTGCCTATTTTGCGCCTGCGGCGGCCGTTTATCTGATG

ATCGAGTCCATTCTTCACGATCGCCACCGGGTTGTTCCCTGTTCTGTCTACCTTGAGGGAGAGTACGGTGTTCAGGGGGC

TTTTTCAGGAGTTCCTGTCCGTATCGGAAGCGCCGGTCTTGAAGCGGTTATCGAACTCCCCCTTTCGGATGCTGAAAAGA

CCGCCTTTGCGGCATCCGTTGAGGCCATTCGTGAGGGTGTTGCCTCCCTTCACAGGCATTTTCCCGATAAGCTT

>namA LfeRT32a_3139 NADPH dehydrogenase (Xenobiotic reductase)

ATGTCCGAGCTGTTCAGTCCCCTTCAGTTGGCTTCCGTGACCTTTCCCCATCGTATTTTTCTTTCGCCGATGTGTCAGTA

TCAGGCTAGGGAAGGTTATGTGACACCGTGGCACTTTGTACATCTGGGTGCGCGTGCCCAGGGAGGTGCGTCTCTTGTGA

TGATGGAAGCTTCTTCGGTAAGCCCCGAGGGCCGACTCTCTCATGGCGATGTTGGTCTCTGGGAGGATGGACAGGGAAAG

GCGCTGGCTCCGCTGGTGGATCTTGTTCATTCATTGGGTTCCAGAATCGGGATCCAGCTGGCCCATGGAGGGAGAAAAGC

CTCCTGCCACCGGCCATGGGAAGAAGGTGGCCGCCCCCTGTCAGAATCTTCTGGTGCCTGGCCTGTTGTCGGACCCTCTC

CGATGGCTTTTTCGGAAGGTTACCCAATCCCGAAAGAGATGGATTCCCCAGAGATGGAGAGGGTTGCGGAGTCTTTTGTC

CGGTCGGCTATCCGGGCGAAGGCCTCCGGGTTCGACGTTCTTGAGCTTCATCTGGCCCATGGATACCTTCTTCACGAATT

TCTTTCTCCTTTGACAAACATGAGACTCGATCAATGGGGAGGGAGTATCGAAGGGCGCATGCGCTATCCTCTGGAGGTTG

TTCGGAGGGTGCGGCAGGTTTGGCCGCAGGAGTTGCCTCTGTTTGTAAGAATATCGGCAACAGACTGGGTTCCTGGAGGC

TGGTCGATCGAAGACTCGGTTGTCCTTGCAAGACAGCTCAAATTGTTGGGTGTCGATCTGATCGATGTGTCCAGCGGAGG

AATTGTGCCCCATGCAGCGATTCCCGTTGCCCCCCTCTATCAGGTTGCTCTTTCGGAGAGAATACGCAGTGAGGCCAAAA

TACCGACAGGTGCTGTCGGTCTTGTCACGGCTCCGGAAGAGGCTGAGTCCATTGTCCGGTCTGGAAAGGGTGATGCGGTC

CTGATCGGGAGGGCCATGCTCCGAAATCCGAACTGGCCTCTTGAAGCAGCGTTGAAGCTGGGCGGTTTTTTGAGTCCTCC

GGATTCCTACCTGAGGGCGTGGCCGCCAGTTGGTTGCCCTGCCGGG

>lysA LfeRT32a_3825 Diaminopimelate decarboxylase

ATGCTTCCTGGTTCCAATGAAGAAACAACAATATCAACCGGATGTTTGGGGAGGTTTTTCGTGAAGCAGTTTCATTATGA

AAATGGCGAACTCTATGTCGAATCGGTTCCTGTCGGGAAAATCATCAGGGAAGTGGGCTCTCCTGCTTATATCTACAGCG

AACAGGCCATTTCGGAAAGTTATTCCTCCTACCAGGAAGCTTTTGCCTCTCATAAGACGGTCATCGCCTATGCGATGAAG

GCCAATGGAAACCTTTCGATCCTCTCCCTTCTCGGGAAAAAGGGGTCAGGAGCGGATGTTGTTTCGGGCGGTGAGCTTTT

CCGGGCCATGAAGGCCGGTATCCCTCCGGAAAAAATCGTGTTTGCCGGTGTTGGAAAAACCGAGCGGGAAATGGAGGAGG

CTCTTGCGGCGGGGATCCTGATGTTCAATGTCGAATCCTCCATGGAGCTCGACACGCTGTCGAAGGTGGCTTCCCGGATG

GGAGTGTTGGCAAATGTTGCCTTGAGGGTCAATCCGGATGTCGATCCCCAGACCCATCCCTATATTTCTACCGGGCTTAA

GAAAAGCAAATTTGGCGTTCCGGTGAAAGAGGCTCTTGCAGAATATGTCAGGGCATCGAAACTTCCGGGAATCCGTCTGA

CAGGAATCCATCAGCATATCGGTTCCCAGCTGACCCAGATTGCCCCGTTCCGGGATTCCTTCACCCGGATGATCGCTTTT

TCAGAGGTCCTCAGGGAAAACGGGATCCGGATCTCCTGGCTGGACGTTGGTGGCGGACTCGGCATCCGATATGATGATGA

AACGGTTCCGACACCCGGGGATATCAGCCGGGAGATCCTTTCAAGGGTCAAGGATCCCTCCCAGGGGATCATTCTGGAGC

CCGGAAGATCGATCGTCGGAAACGCCGGGATTCTGGTGACGCAGGTTCTCTATGTCAAGAAGACCGAGGTCAAGACCTTC

TATATCGGAGATGCTGGCATGAACGACCTGATCCGGCCTTCCCTGTACGGGGCCTATCATGATCTCTGGCCCATCCGTGT

CCGGGAAGGTGCCCCTGTTAAGGCCGACCTTGTGGGCCCTGTCTGCGAGACCGGAGACTTCCTGGTCCAGGACAGGGATC

TGCCGCCGATTGCGGCGGGCGACCTGGTCTCTGTCATGAGTGCCGGAGCCTATGGTTTTGCCATGGCATCAAACTATAAT

GCACGGCCAAGACCGCCAGAGATTCTGGTCAGCGGAAACCACTTTGAAGTGATCCGTCCACGGGAGAGCTATGAGGATCT

GATCCGGTCGGAGTCCGTGCGT

>trpE LfeRT32a_2073 Anthranilate synthase

ATGATTCTTTCTCTTTCGAAAGACCAATTCCGGAAGTTATCCTCTGGAAAACGATTTATTCCTCTTTTTGGAGAAATTCT

TTCGGATAGGATAACCCCTGTTTCGGCCTATGCATCTCTTGATCACGCAAAATATCGATTCCTTCTCGAAAGTGTTGTCG

GAGGAGAGAGCTGGGGAAGATTTTCCTATGTCGGTGGCGGTGTTCTGTACCGTTTTGAGGGTGATGTTTCGAGGGGGTTG

TCTGTAACTGACCTGACTCAATCAGGCCAGGGAAAATCCTGGCACAGGGATGGTGACCTTGTTTCTTCCCTGAAAGATGA

GATGAGGTCTCTGTCAGTCGATTCTGATCTTTTGCCGGTTGGTCTTGCCGCGGGTGTAGTGGGATATCTCTCGTACGATA

TGGTGCGTGAATTTGAGAGGTTGCCCGATATTCTTCCTCCTCAGGAAGATTTTCCTGATCTTTATTTTGTTTTTCCGGAG

TTCTTTCTGGTTTTTGATCATGTTCTCGGTAAAATCAGAATTCTTACATGGATCGATTGTGCACAGAAAGCCGCTCCGGA

TGACTTGTATGAAAATGCCTGCCTCAGGTTGATGAGGCTTAGGGAATCTCTTTCAGTCCCATCCGATTCTGAGAGTTCTG

CGGTTGGTTCGAGACCTCTTTCATTTAATGAAACTCCCACGTCCGAGGTTTTTGAAGAGAATGTCCTGAAAGCTAAAGAA

CATATAAGATCAGGGGATATTTTTCAGATCGTCCTGTCCAAGAGATTTTCCTTCCACTTTGATGGGGATCCGCTAAAGGT

CTATCGTGTATTGAGGTCGATCAATCCTTCCCCTTACATGTACCTGATCCAGGATGGAGAAATGGCGATTGTCGGATCAT

CACCGGAACTTCTTGTCCGGGTTAAAGGAGAAAAGGTTGAGGTTCGGCCTATCGCCGGCACTGTCAGGCGGACCGGTGTT

CCTGAGGAAGATGCATTGCGGCAGAAACAACTTCTGTCGGATCCCAAAGAGCTTGCGGAGCATGTCATGCTCGTTGACCT

TGGACGCAATGACATCGGGCGGGTAAGCCGCCCGGGAAGTGTCCGGGTTCCGGAAATGATGGTTCTTGAGCAGTATTCTC

ATGTGACACATATCGTCTCCCATGTAGAGGGGCTTCTTTCGGAGCAGAACGATGCTTTCAGCGTCATTCGCGCAACGTTT

CCAGCGGGAACCCTTTCCGGAGCTCCTAAAATCAAGGCGATGGAAATTATTGAAAAGCTGGAGACGATGCGCAGGGGACC

TTATGCCGGAGCGGTCGGGACGATCTCGTTTTCCGGGGACTGCGATCTGGCCATTGCGATTCGGTCTATTTTTATTCGTG

GAAAAAATGCGTTTCTTCAGGCAGGGGCCGGGATTGTCGCAGACTCGATCCCCCGCAATGAAGATCAGGAAGTGGCGGCG

AAGGCTGCGGCGATGATGGAAGCCTTACGCATTGCTAACGGGGAAAGAGGCTCATGGCTTTTT

>cydA LfeRT32a_1644 Cytochrome d ubiquinol oxidase subunit 1 Respiratory chain

GTGGATCTCTCCCGTTTGCAGTTTGCCGTTACGGCTCTTTACCATTTTTTGTTTGTGCCCCTGACCCTGGGGATGACTTA

TCTTCTGGTCATCATGGAAACGGTCTATGTCATGACAGGAAAGCCCATCTACAAAGAGATGACCAGGTTCTGGGGAAAGC

TCTTCGGAATCAACTTCGCCCTGGGAGTCACGACAGGTCTGACCATGGAGTTCGAGTTCGGAACGAACTGGTCTTATTAC

TCCCATTATGTGGGAGATATCTTCGGAGCGCCCCTGGCCATTGAAGCTCTGATGGCCTTTTTTCTCGAATCCACCTTTGT

GGGCCTGTTCTTTTTTGGATGGAACCGGCTCTCCAGAGGCGGCCATCTTTTTGCCACCATCATGACGGCGACAGGAACCA

ATCTCTCCGCCCTGTGGATTCTTGTGGCCAATGGATGGATGCAGAACCCAGTGGGGGCCCAGTTCAATCCGGATACCATG

CGCATGGAGGTCACAAGTTTCTGGGCTCTCTTTCTGAACCCGACAGCCCAAGCCAAGTTTGTCCACACCGTGAGCGCAGG

TTATGTCACAGCATCCTGCTTTGTGCTTGGTATCAGCGGCTGGTACCTCCTGAAGAAAAGACATATGGAAGTCGCCAGGA

GATCCTTCCGGATTGCCGCCGCCTTTGGCCTTGCAGCCACAGTCTCTGTCATCATCCTTGGCGACGAGTCAGGATACCTT

GATGGAATCGGGCAAAAAACGAAGCTTGCGGCCATGGAAGCCATGTGGGACACCGAGCCGGCTCCGGCCTCATTCAACCT

GATCGCAATACCGGACCAGAAGAACATGAAAAACGATTTCCAGATACGCATCCCCTGGGTCCTTGGGCTGATCGCAACCC

GCACCTATGACACAGCCATCCCCGGGATCAAGGAAATCGTGGTCAACAATGAAGTGAAGATCAGGAAAGGGATCCTTGCG

GTCGAGGCTCTCGAAAAATTCCGCAAGGATCCCAAAAACACCGAACTCAGAAAAGAATTTATGTCTGAAAAGGATAATCT

CGGCTTTGGACTTTTGCTAAAAAAATATACACAGGACGTTTCCCTTGCGACGCAGGAACAAATCCATCAGGCAGCACTTG

ATACCATCCCTCGCGTAACACCATTGTTCTGGGCGTTTCGGGCAATGGTCGGTCTTGGATTTTTCTTTCTGGCTCTTTTT

GCCGCCGCTTTCTACTATTCGAGCAAAAGCCAATGCGACGAGAAAGCGTGGCTTTTAAGGCTCGCGATTTTCTCCATTCC

CCTGCCTTATGCCGCAAGCGAGCTGGGATGGCTTGTCGCGGAGTACGGACGGCAGCCATGGTCTATCTATGGGATCCTCC

CCACCTTTCTGTCCACCTCATCCCTCACAACCGGAAGCCTGATTTTCTCTCTCGTGGGGTTCACCCTGTTTTATACCGTG

CTTCTGGTTATCGAGATTGTCCTGATGAGAAAATATGCGAAAATCGGCCCCGTCTCCGAGCTTTATTCCGAAACCGACAC

ATCGAGGGGCGTTTCAGGAATGCTTGATAAAGGAGACCTCCATGTT

>hycD LfeRT32a_2964 Hydrogenase. Formate hydrogenlyase

ATGACACAGACCAGCACTGTCCCGCACCAGTGGATTGCGGACCTTCTCTTCAAAGGACTCGGGGAGCTCTTCCAGACAGC

TTCTGTCATCTTTTTTGCACCCATTTTTCTCGGGTGGGTCAATCTGTGCCGCTCCTGTCTCCAAAGCCGTCGACCGGCCG

GGATCCTCCAGCCGTATCGGGACCTGCTGAAATTACTGAGAAAAGAAGTCGTCCTTGCCACCGGGGCATCACCTCTTTTC

CGGATCACCCCCTATATCGTTTTTGGCTCGATGGGCCTGGCAGCGGCTCTTGTTCCCGCTGTGACAACCGACCTTCCCTT

CGGACCGGCAGCCGATGCAATCGCCCTTGTCGGCATTTTTTCCCTGGCGCGCATTTTCATGGCGCTTTCCGCGATGGATA

TCGGAACCCCCTTCGGCGACATGGGGGCCCGACGTGAGATGATGATCGCCTTTCTTGCCGAACCCGCGACGATGATGGTC

CTTTTTACGGCATCCCTGATCTCGAGGTCCACCTCACTTTCCACCATTGCCGAAACACTTGGAGGGCGCCATTTTACCCT

CTATCCCAGCCTCGTGTTTGCGACAACGTCCTTCTTCCTGATTATTCTGGCCGAAAACTCACGATTGCCCATCGACAATC

CTTCGACACACCTTGAACTCACCATGATCCATGAGGCGATGCTCCTCGAATACTCCGGAAGGCATCTCGCACTGATGGAA

TGGGGAAGTTCCATCAAGCTTCTGCTCTATTTCTCCATCCTTATTTCGTTCTTCCTCCCGTGGGGTATCTCCCATGAGTC

ATCCTTCGGAGGATTGTTGTCCGGATTTGCTTTTCTCGTCCTCAAGCTTTTCATCCTGGGGACATTGCTGGCCATTCTGG

AATCAACGCTTGCCAAACTCCGGCTTTTCCGGGCCCCCGAGTTTTTAGCTGCGGCATACCTTCTGGCCGTGATCGCCTTT

CTTTCCCATTTCATCCTGGAGGTG

>hycE LfeRT32a_2961 Hydrogenase. Formate hydrogenlyase

ATGGATGAAAGTCGCTCCCCCTGCAGCCCGTTTCTTGAAGTCATCGAAACAGACAGGGAGGGGTATCTCCATCTGGCAAT

GGAAATGAGCGAAACCGGAGGGAGGCTCGTTTCGGTCTGGGGATTTTTCAGCGAAAGCCTCTCTCTTCTTTTTTCCTCCT

ACTTCAGGGGAGAAAGGCTCTTTACAGGCCGGCTCTCTCTTTCAGGAAAGATTTCCGACCTTCCCTCCATCACCCCGTTT

TTTCCCGTCGCTTCAAGAATGGAACGAAGCATACGGGATCTGTGGGGAATTTCTTTCTCCGGCGCTCAGGACCATCGTCG

ATGGCTTGACCATGGTCTCTGGGAAAGGGTCCCCCTGACCCAAAAGGCGCAGCCGGAAACTTTCGGAGAAGGAGACTATC

CCTTTGTCCGGGTGGAGGGAATGGGTGTTCATGAAATACCTGTCGGACCGGTCCATGCCGGAATGATCGAACCTGGCCAT

TTCCGTTTCCAGGTGGTTGGCGAAAAGGTTCTGAGGATGGAGGAGCGTCTGGGATATAGCCACAGGGGAATCGACGGTCT

CTCCCGGAATATCCCCTGGAACAGGGGGATAAAGCTCGCAGGAAGAGTCTCCGGGGATACAACGGTCGGACATGCCCTGG

CCTATTCCTTGTCGATCGAATCGGCCATCGGGATGGAGGTCCCCAAAAGGGCGCGGTTTCTTCGGGGATTGCTGCTTGAA

CGGGAAAGAATCGCGAACCATCTTGGGGATCTTGGCGCATTGGCCAATGATGCAGGACTGTCCTTCGGGCTTTCCCAGTT

TCTGATCCTGAAGGAGGATCTCCTGAGGCAGAACAAGTCCGTATTCGGTCACCGGCTTCTCATGGATCTCATTTCACCGG

GAGGGGTGAGGAAAGATCCAGGCCCCCTTCCCATCGCCGAAATGTCCGGCGAAGCCTCCCGGATTCTTCAGGAAGTCGAG

ATCCTCGAGATGATCTTCGAACAGCACGGAGGTCTTCAGGACCGATTCTTCGGAACGGGGACCCTTCCTCCGGAAACAGC

TCAAAAAATGGGTCTTTGCGGAGTTGTCGGAAGGGCAAGCGGACAACCTTTCGATCTGAGACGTCTCTGGAAAGAGCCGC

CGTTTGACGAGACTGGCTTCACCCAGGCACTCGAAACGGGAGGAGATGTCAGGGCCCGCGTGGCGGTCCGATTCTTTGAG

GTGAAGGAATCGCTAAGACTTGTCCAGGAGATTCTCCTGAAACTTCCCTCCGGAGACGTCTTTCTGGAGCCTCCCTCCGG

GGAGTCGGGAAAGGAGGGAATCGGTCTCGTCGAAGGGTGGCGCGGGGAAATCCTCTGCTGGTCGAGGCTTGAAGGAGGAG

ACATCGTCGCAGCCAGCCACTTCCATGACCCTTCATGGATTCTGTGGCCGGCACTCGAAATGACCATTCCAGGAAATCTG

GTCGCGGATTTCCCGCTGATCAACAAATCCTTCAACCCGAGCTATTCCGGCCACGACCTG

>hycG LfeRT32a_2960 Hydrogenase. Formate hydrogenlyase

ATGCATCATATTCTTTTAAGAACCCTGAAAACAGGCATCGTCACTGAAAAAACGCCAGAACTTCCTGAGGAAATGAAAGA

AATTTCAAGAAAACTCCTGGAGCGGGGGATCGGCCGTTTTGGGCGCAGCCTTTTTATCCGCCATCTCGATGCGGGATCCT

GCAACGGATGCGAAGTGGAAATCGGGATGCTCGGAAGCCCCTGCTTCATGCTCGAACATCTGGGGTTCAAATTTGTCGCC

TCTCCGCGACATGCAGACCTGCTTCTGGTCACGGGACCGGTCTCCCTGCATATGAGGCAGGCCCTTCTCGATACCTATGA

AGCGATGCCATCCCCGAAACTGGTCGTTTCGGTCGGAGGTTGCGCAGATGACGGCGGAATTTTCAGGGGAAGCTATGCGA

TCGCAGGCGGGGTTTCGGAAGTCGTCCCGGTCGACCTGCATATCCCCGGGTGTCCCCCCGAACCGCTGGATCTGATCAGG

GGTCTGATGACGGCGGCCGGTGTT

>hemL LfeRT32a_3236 Glutamate-1-semialdehyde 2,1-aminomutase (GSA-AT)

TTGACGCGCTCAGAAGAATTGTACCGGCGGGCAAAAACGATTTTCCCCGGAGGAGTCAGCAGTCCTGTCAGGGCGTTTGG

GGCTGTCGGCGGAGTCCCCCCATTCATCAGGACCGCCAAGGGATGTCTTCTGACAGACGTCGATGGAAATGAATACATAG

ACTATGTCCTCTCATACGGCCCCCATATTCTGGGTCACGGAGATCCCGATGTCCTCGAGCGTCTTCATGCGGCCATCGAC

CGCGGAATCAGTTTTGGCGCCCCTTCCGAAGAGGAGCTCCAGCTCGGGGAGATCATCGTTTCAGCCCTTCCTTCCGTTGA

CCGCCTGAGATTCGTCAATTCCGGAACCGAAGCCACCATGAGCGCAATCCGACTGGCAAGAGGATACACCGGCCGCTCCA

GAGTCCTGAAATTCGAGGGCGCCTACCATGGTCACGCCGACTCCCTTCTCGTCAAGGCCGGTTCAGGTGGCGCCACCTTC

GGCGTCCCATCGTCCGCCGGAGTTCCCCCTGAAATAGCCCGGGAAACACTGACCCTTCCCTATAACGACACAGAGGCGCT

CAAAACTCTTTTTTCCTCGGAAGGAGAAAAGATTGCCTGTGCCATTATTGAACCCGTCAGCGGCAACATGGGAGTTGTCC

TTCCGGACGAGGAGTTCCTCAGAGAACTCCGACGCCTTACAGAACAATACGGTGCACTTCTGATCGCCGATGAAATCATG

ACCGGATTCCGCCAAACGTACGGAGGCGCGCAAATCCTTTTCTCCATGGAACCCGACATCACAACCCTGGGCAAGATTAT

AGGCGGCGGTCTTCCGGTCGGTGCATACGGAGCCTCTGCCAAAATCATGTCCAGGATTTCCCCCGAAGGACCGGTTTACC

AGGCGGGCACACTTTCCGGAAACTCCGCATCCATGGCCGCCGGTCTGGCAACGATCCAGAAACTCAAGACCCCGGCCCTT

TATTCCCAGCTTGAAGAGCAGGGCAGAAAACTTTCCCAGGGGCTCAAGGAAATCATAGGACGCCTCTCCCTTCCGGCTAC

GGTCAATCGAATGGGGTCAATGATGACACTCTTTTTTACCCCGGATCCCGTTGTCGACTGGACATCCGCAGCCCGATCGG

ATACAGCCCTGTTTCAGGTGTTCTTCCACGAATCCCTGAAACAGGGGATTTACCTTCCCCCCTCCCAGTTCGAAGCCTTC

TTCCTCTCCACCTGTCACGACGATGAGGTCATCCGCACCTCCATCGAAAAAATATCCAAAGCCCTGATCGCCTGCCGCAA

CTGGATTGACCAGGGCCGGCCGGAATACAACCCCTATGAGGAGAAACACTCT

>moaA LfeRT32a_3614 Molybdenum cofactor biosynthesis protein A

ATGGCAATATCCATCCACCAGGCCGTCAAGGTTGGTTCATATATTCTTAGCCAGAAACTGCGCGGACACAGAAAGTTCCC

TCTTGTACTGATGCTTGAACCTCTTTTCCGGTGCAACCTTGAGTGTGCCGGATGCGGAAAGATACAATATCCCGAGGAAA

TCCTGAACAAGAGGCTCTCTCCGGAGGAATGCTTCAGAGCCGTTGACGAGTGCGGCGCACCGGTCGTGACCATTGCCGGT

GGAGAACCCCTCATCCATCAGGAAATCAGTGAGATCGTCGAAGGAATCGTCGCAAGAAAGAAGTTTGTTTACCTCTGTAC

CAACGCGATCCTGCTTGAAAAACATCTTCACCGGCTCAAACCTTCACCCTACCTGACCCTTTCGATCCATCTTGACGGAT

TGAAGGAAGACCACGACCGTCTCGTATGCCGAAACGGCATATTCGATGTTGCAGTCAGGGCAATCAAGGATGCCAAGTCA

AAAGGGTTCAGGGTCACAACGAATTCGACGGTCTTTGAGGGGGAAAACCCGGAAGACCTTCACAGATTTTTTGATTTTGT

CTCCACGCTCAAGACGGACGGGATGATGATTTCCCCTGGCTATTCCTATGCATGGGCCCCTGATCAGGCCCATTTTCTGA

GACGGGAGAGGACCAAAGAGTTTTTCAAGAAAATCTTTGCTCCAATGCATGAAAAGGGAAACACAAAACAGTGGAACTTC

AATCACAGCCCCTTTTACCTGGATTTTCTCGAAGGCGCAAGGGATTACGACTGTACTCCATGGGGGAGTCCGAACTATTC

CGTTCTGGGGTGGCAAAAACCCTGCTATCTCTTGAACGATGGTTATGCTTCAAGCTTCAAGGAGCTTATGGATACCACCA

ACTGGGACCAGTACGGCCACAAAAGCGGTAATCCCAAATGCCAGGACTGCATGGTTCACTGCGGATTTGAGCCTTCCGCA

GTATCAGATGCGACATCAAGCATCAAAAACACAATCCGTTCAATATCGAGTCTGATTCCTGCTGGA

>cobT LfeRT32a_1266 Nicotinate-nucleotide-dimethylbenzimidazole phosphoribosyltransferase

ATGACGATTTTTGAGCAGGTGGATCTTTGGGTATCAAGAATTCCGGGTGAAGATGAACATGCATCCCAAAAAAAGGAAAT

CCGGCAGGCGATCCAGTCTCGTCTGGACTCCCTGACCAAACCGCAGGGATCTCTGGGAAGGCTTGAGGATCTGATCTTGT

GGTATGGCCTTGTTTGCCGCCAAACCCTTTTCCCGGATCCTGCCGGGGTGGTCTGTGTTTTTGCCGCGGACCACGGGATA

GCCGCAAGCGGAGTTTCTGCCTTTCCCCAGGTCGTGACCGTGGAGATGGTCAAGAATTTTTCAAATGGCGGAGCGGGTGT

CAATGTCCTTGCCCGGCAGGCCGGGCTTGAGTTGATGGTTCTTGATATGGGAGTCAATGCCGATCTTTCATTCCTTCCCC

TGGTCAAAAATGCGAAGGTTTCTTATGGAACAAGAAATTTCCTCTCGGAAAGGGCGATCGCTCCGGATGAAATGATGCAG

GCGATGAAAACAGGATTTGACCTCGCACTAGGACTGAAGTCGCAGGGAAAAGGATTTCTTGTCCTGGGGGAGATGGGGAT

CGGAAACACGACCTCCGCATCCGCGCTGATCTCGGCGCTTCTGGCTCTTCCTCCTGTTCTGGTGACCGGACGCGGAACGG

GTCTTGACGATCAGGGTTACCGTAAAAAACTTGGAGTGATTGAGCAGGCCCTTCTGTTCCACGGTCCGACCATCACCTTT

CCGATTGAGTGGGGGATGGCGGTGGGAGGGTATGAGATCGCCGCCATGGCAGGAGCGATCCTGGGAGCGGCCACGATTGG

CCTTCCGGTCATACTCGATGGGGTGATCACATCGGCTGCGGCACTTCTGGCCTGGAGAATCTGCCCGGCTGTCAAGAATT

GTCTTCTGGCCGGACATGTTGGCCACGAAATCGGACACAAGGCCGTTCTCGAGCATATGGGACTCAGTCCGGTTCTTGAT

CTGGACCTGCGCCTGGGAGAGGGGACCGGAGGCGTTTTGGCTTCGTTGATCCTGAAGTCCTCTATTGCACTCTATCACCA

GATGGCCACATTTGAAGAAGCCAGCGTATCGGGTAAAACCGGA

>nadA LfeRT32a_2948 Cofactor biosynthesis; NAD(+) biosynthesis

ATGAATAACCTCTCTCCTGCAATAGAAAACAGCACAACCGTCGTTCAGGATCCTGTCGTGGCCGATCTTGTTGCCCGGAT

CCAGAAGCTGAAACAGGAACGGGGTGCGGTCATCCTTGCCCATAATTATCAGGTTGGCGATGTTCAGGATGTGGCGGATC

TGGTGGGCGACTCCCTGGAGCTTGCCCGATGGGCAGCTTCAGGGTCGGCTCCGGTCATCCTGTTTTGCGGCGTCCATTTT

ATGGCGGAGACAGCCAAGATTTTGAACCCCACCCGACGGGTCATCGTTCCGGATCTCAATGCGGGATGTCCGATGGCGGA

CATGATTACCCCGGAAGAGGTCGACCGCTTGAGAGCCGAAAACCTCGGAGCGGTCGTTGTCACCTATGTGAACTCCCCGG

CGGCGGTCAAGGCAAAAAGCGATATCTGCTGTACATCGGCAAATGCGGTCAAGGTTGTGAACTCGATCTCCAAAGACGTT

CCGATCATCTTTATTCCCGATCAGTTTCTGGGCGACTTTGTCCAGCGGCAGACAGGCAGGGATTTGATCCTTTTCAGGGG

ATTTTGTCCGACTCACATGAGAATCATGGCAAAGGACATTCATCAGGCAAAAAAGGAGAATCCCGACGCGCTGGTTCTTG

CCCATCCTGAATGTCAGCCAGATGTTGCAATGATTGCCGATGTCGTGACTTCCACCTCGAAGATGGCGACTGAGGTGAGG

AACTATCCTGGGGAGACGGTGATTGTCGGCACTGAAACGGGGATGATCCACCGTCTTCAGAAGGAGAATCCCGGGAAGAC

CTTTGTCGCGGCCTGCGCGACCTGTGATTGTGCCAATATGAAGGTCAACTCCCTCGAAAAGATTCTCTGGGCACTTGAAG

ACATGTCCCCGGAAATTGAGATCCCTGAAGAGGTCAGGATTCCTGCTCGTCAGGCACTTCAAAGAATGCTGGACATTGCC

>thiG LfeRT32a_4626 Thiazole biosynthesis protein. Thiamine biosynthesis

ATGGAAAACGGAGTTCTGGAAGACATTGCTGGCACTGATGATCCCATGGTTATCGGTGGTATCTCATTTCGCTCCCGTCT

TTGGGTCGGAACCGGAAAGTACAAGGATTTCGAGCAGACCAGGGAGGCGATTCTGGCTTCAGGAGCCGATGTTGTCACGG

TGGCTGTGCGCCGGGTGAATATCCTGAAAAAAGATGAGCCCAATCTTCTGGACTATCTTCCGGTGGGCAAGTTCAAGATC

CTTCCGAACACCGCCGGATGCTACTCTGTCGAGGATGCGCTCCGATATGCAAGACTTGCCCGGGCTGCAGGTATCTCGGA

TATGGTCAAGCTTGAAGTTCTGGGTGATCCAAAAACCCTTTTCCCGGACGTCATCGGTCTTCTTGAAGCGGCAAAGATTC

TTGTGAAAGAAGGCTTCATCGTCTTCCCCTATACCAACGATGATCCCATCCTGGCAAAAAAACTTGAGGATGTCGGATGT

GCCGCGGTCATGCCATTGGCTGCCCCGATCGGTTCTGGTCTCGGGATCAGGAATCCCTACAATCTGAGGATCATTCAGGA

GACGGTATCGGTTCCGATCATCGTTGATGCAGGAGTCGGAACGGCTTCCGATGCCGCTGTCGCCATGGAGATGGGGGTCG

CTGGCGTTCTGATGAATACAGCCATTGCAGAGGCCAAGGATCCGGTCCGGATGGCAAGAGCCATGCGGTATGGCGTTCTG

GCCGGACGGGATGCTTTTCTGGCCGGTCGTATGGGCCGCAAGCTCTATGCAAATGCTTCAAGCCCTCTTGAGGGAATGCT

GGAGATGCCTTCTCCCGGAGCCCGCTCC

>thiD LfeRT32a_2854 Phosphomethylpyrimidine kinase. Thiamine biosynthesis

GTGGTCTGGGCTATTGGAGGAATTGATCCATCGGGAGGTGCGGGAATCTACCAGGACCTGAAGGTCATGTCGTGGTCAGG

AGTCCATCCGATGGGTCTTCCTGCGGCTCTTACGGCGCAGAACATCGATCATGTCAATGGAGTGATGGCTGTCGATCCGG

ATTTTTTCACCAGAATGGCCAGGACCCTTTATGAGAAGCACCCTCCGGTTGCCGTGAAGGTGGGTCTTCTTCCTGTGGCG

ATTTCAAAAGCTGTTATCGGAGTCTTGAAGGGTCTTCCTGAAGATGTTTTTTTGGCGGTTGATCCGATTTTCAGGTTCGG

ATCAGGAGATTCCTTTCTGGAGGAGGCCGCTTTCCGGGAGATGGCCCACCAGATTTTTCCCTTGGCGGATCTTGTTCTTC

CCAATATTCCTGAGGCTGAGGTTCTCCTGGGGCATCCTCTCCTTCCGGGGGTGTCCGGTCTTGAGAAGGGTGCTCTTGAG

CTTCGGGAGCTTTATGCTCCGGCTGCTGTCTACCTGAAAGGAGGGCATCGTCAGGAGCAGGTCAGGACCGATGTTTTTGT

CGATGGCGAGGGAGCTTTTTTTCTTGATCGTCCGGAAGTTCCTGTTCCATTTCTTCATGGAGGAGGCTGTACACTGGCAA

GCCTCCTTGTTTCGGAGATTGCCAGGAGTCCCCTGTCACCGCTCCGGAATCTCCTGTCTTCTCCGAGGGATATCTTTCAG

CGTGCGCTTCTGTGGGAAGCGACCCGGCCGGGAAGCGGGAGAAGGACTTTTGAAGATTTCTTTTCGACTTCGAAGACAGC

AGGC

>thiE LfeRT32a_4627 Phosphomethylpyrimidine kinase. Thiamine biosynthesis

GTGAGCAAAATTCTCCCTCTTTTGTTCGTGACGCCCGAGGACCTTCCCCCGAAGATTATCGTTTCAAGGGCGCGGGAAGC

CGTTTCCGGTGGTGTGAGTGCTGTTGTCGTGAGGCGAAAGACGGGTCCGTCCCGGGATTTTCTGGATCTTGGGTATCTTT

TGAGAGATGGCCTTGGGGAGGACTTTCCGATCCTGGTGAATACCCGCCTGGATCTGGCCCTCTCCATCAATGCGATGGGC

ATTCACCTGCCAGAAGACCATGTTCCGATTGAGGCCATTCGTAAAAAGGCCCCCGCTCATTTTCTGGTGGGAGTTTCCTG

TCACAGCCTTGAATCTGTCAGGAAGGCGGTGAGGGAAGGCGCGGACTATATTTTTTTTGGGCCGGTATATGAAACTCCAT

CGAAAGCGTCCTATGGGCCTCCACAGGGGCGGGAACTTCTGGGACAGATTGTGCGGGAAGCTGATATCCCTGTTATCGCC

ATCGGAGGGATCCATCGGGAGAATGTTGAAAATGTCCGCAAGACAGGAGCGTCCGGGGTGGCAATGATTGCGGAGATAGC

CTATTCGGCCGATCCAAAAGCCAGAGCTTTTTCTCTCCGTGGGGGCTGGACCCGCGGGGCTCAGCCA

>csd LfeRT32a_3634 Cysteine desulfurase

ATGAAAAAAGTATCACCGGCCGAGATTATTCCTAATGATCTTTACAGCCAGAAACGAGCGGAGTATAGAAAAAGTATTAT

TTCCATCAAGAAAGACCGGTCAATCAATCTCGGTCCCCTACTGCGAATCGTCTTTGAAAATCATGCAACGGTACTCTTTC

AGATCCAGGAAATGCTTCTCGTGGAGGGAATCAGCGATCCCAAGAAGATCCAGGAAGAAGTTGACACCTACAATGAACTC

ATTCCCGGACCTTGCGAACTCAGCGCAGTGTTATTCATCGAAATCACCAGCGAAGACAAAATACAGGAAACACTTGAGAG

ACTTCGTGGTCTTGATCGACAGCCGTCTGTCCACCTTCTCTTCCCTTCCGGAAAAATCAGTGCCATTTTTGAATCAGACC

GCTCTGATGATTCAAAAATGAGCTCAGTGCATTACATCAGATTTCCATTGGGTAATCAGGGTGCATCTCTCCTCAAAAAC

TTGAGTGAAGGCGAAAAGGTTTTCCTTTTATCTGACCATCCGGCCTACTCGGCAATGGTCCAGCTTCCAACACCTCTTAT

TTTAAGCTTGAGGAGCGATCTTGAC

>pyrR LfeRT32a_2024 PyrR bifunctional protein

TTGTGCCTGGTTGGAATCCTTCAGGGTGGTGCGATTCTTTCCCAAAGAATTGGGGCTGTGATTGAGAGCATAGAAGGAAT

TGCCCCACCAATGGGGACGCTCGATATCACTCTTTACAGGGATGATCTCGCCATTCGCGCTGCCCCTCCTTTCTTGAGAG

AGAGCAATATCCCCTTCTCCATCGATGACAGCCGAATTGTTCTGGTCGACGATGTGTTGTATACGGGGAGAACGGTTCGT

TGTGCCCTGGATTTTCTGATGGATCTTGGCCGGCCATCTTCTGTACAGCTTGCGGTATTGATTGACCGGGGCCACAGGGA

ACTTCCAATCCGGGCGGATATTGTCGGAAAAAGCATCCCTACGAGCAGGCAGGAGAAAGTTCGGGTCCTTCTTGAAGAAA

ACAATGAATCTGTTACCTTGGTCAGGATGTCATCA

>pyrF LfeRT32a_2970 Orotidine 5'-phosphate decarboxylase (OMP decarboxylase)

TTGATTCAGGACAAGCCAATCTTCCATTCTTCGCCTGCTGTGGACCATTTGAGACGATCGCTCAGGAACAGGATCTGTCT

GGCCCTTGATGAGCCGGATATTGTCCGTGCCCGGTCGCTTCTCGGGGCGATCGGGGAAAGGATCGGCATGGTGAAGGTGG

GGCCGGTCCTTTTCATGAGGGAGGGGATGAGACTCCTGGATGAGATTTCCGACAGGGGCATTCCTCTCTTTCTGGACCTG

AAGTGGCACGATATTCCCAATACCGTTTTTGGTGCGATATCCGGTCTTTCTCTTCCGACGCTCAGTCTGCTGACGGTTCA

TGCCCAGGGCGGGGAGGCAATGATTCGCGCGGCAAGGGAGGCCTCCGAGAATCTTTCCGGTGTTTCTCCCCTGGTTTTGG

CGGTGACGCTGTTGACCCATCTGGATTTCCGCGAGCTGGATCTTTTGGGGATTCCCGACCGACAGGAGAAAGTTCTTGAA

CTCGGCGGCCTGGCTCTTGCTTCCGGTGCCCATGGGCTTGTCATGGCTCCTGGCGACCTTTTGTCCGCCAGAGGGCGTTT

TGGTCCGGCCCCCTATCTGGTGACTCCCGGCATCCGGTGGGAGGGGGAAGCGACATCCGGCCCGAAAGGCAGAAAGGATG

ACCAGGTTCTGGCGGGAAGTCCCAAAGATGCACTTCTTGGCGGGTCGGACCTTCTGGTCATCGGCCGTCCTTTTTTGAAC

AGTCCTGACCCCAAAAGGCTGCTTTCAGATCTTCTTGGGCTC

>lig LfeRT32a_3197 DNA ligase

GTGGGCTTTTCGGTTGATGAGGTCAGTGAAGAGTTTCTCAAGATGGAGGGGACCAAGGGCAGGACCGAACTGGCCGGAAT

CCTTGGAAATCTTTTCATCCGCTGCGATGAGGAAGAAATATCTCCGCTTGTATATGTCGTTCAGGGACAGCTCTCGCCTC

CCTTTCTTGGGGGGCCCATGGGAATGGCTTCAAGGCTTCTGATCCGCTCTCTTTCGGAGGTTAAGACACAGAAGCCGTTT

TCTGAACTCTCTCCTGAGGAAGCCAAGCGAATGGAGAAATCCCTTGATGTGATGCTTGCTCATTATGGGGATCCGGGAGC

CCTCGCGCAGGAGGTCCTGTCCCGAAATGGACCCTCAAAGCTGTCTTTTCTTGAAGTGTTCAGGATTCTTGAGCGGCTGT

CAAGGATGGAAGGGGAAGGTTCGCAACTTGACAAGGTAGGCGAGCTTGCAAGCCTTTTTTCCAGGCTGTCGCCATTGTCG

GCCCGTTTTGTTGCGCGATTCGTCATGGGAAAACTGCGTCTTGGGGCCGGTGATTCAACCATTATCGAGGCACTGGCTGT

TTCAGGTGGCGGCAGAAATGCAAAAACAATCGTTGAGAAAGCTTATAATATTTGCTCGGATCTTGGTCTTGTTGGGACAA

AGATTAAGCAGGGCGGGTTGGAAAGCCTGTCTTCTCTGACTCCTTCTCCCGGCTTTCCGATAAGGGTTGCGTTATGTGAG

CGTCTGTCTTCCGGGGAAGAAATCATTGCCAAGATCGGACGATGTGCTATCGAGAGCAAGTATGACGGTTTCAGATGCCA

GATCCATATCATTTCCGGAAAGGTCGAAATCTTTTCAAGGAATCTTGATTTGATGACCTCCATGTTTCCGGAGATTGTTC

GAGCCACAAAAGAAATTTTTGGTAACCGAAGTGCCATCTTTGAGGGAGAAGCTCTGGGGATTGATCCCGAATCGGGAACA

TATCAGCCTTTTCAGGTCACGATTACCCGAAAGAGAAAACATAATGTTCTGGAAAAATCCATGGAGATCCCTTTGCGGCT

TCTGGTTTTTGACCTGCTCTTCCTTGACGGAGTTTCATGGATGGATCGTCCTTTTATCGAGCGGCGGGCTGAGGTTGAAG

CCATCTTTGGGGCAGAACAGTTTGCCATTGAAGACGTGGAGAGACCACCACAGGGAGTTATTGGTTCTTCCCGACTGATT

TTTACCGGGGATGCTTCCGAAGTGAATGAGTTTTTTGAGGAAGTGCTTGAAGAGGGCTTCGAGGGGATTATTGCGAAGCG

TCTGGACGGAAACTATACTGCGGGATCAAGAAATTTTAACTGGATCAAGCTTAAGAAGAGTTACCAGGGAAAAGTTTCAG

ATACACTGGATCTCGTTATCATTGGTTACTTTTTGGGAAAGGGGCAAAGGCTCAAGCTGGGTATCGGGGCCATTCTGGCC

GCTGTTCGGGATCGCGAGTCCGGGACCTTTCCCTCTATTGCCCGTATTGGATCCGGGTTGACTGAAGAGAAGTGGATGAT

GCTTGCCGAAATGTTGAGGGAGAGTGTTGTCCCGGATCGTCCTGTGGATGTTGTGAGTGACATTGTTCCCGACTTCTGGG

TCCTTCCCAGGTTTGTTGTGACGGTCAGGGCTGATGAAATCAGTCGTTCCACCATGCATGCCTGTGGCAGGGGGGGGGCA

TTTTCCTCCGGGGGTCAGATGGAAGGGTATGCCCTTCGCTTTCCCCGGATGGTATCTTTTGTGCGTGCCGATAAAAAGCC

GGAAGATGCCACGGAAGTTTCTGAAATTGTCTCCCTGTTTGATTTGCAGAGAAACCTCTCCCGGAGATCGTCAGAGGAAA

AGACTTCTTCTGCCCTGGAG

>recJ LfeRT32a_2750 Single-stranded-DNA-specific exonuclease RecJ

ATGCAATCGCCAAGGAATGAATATGACAGTCCTAACGGTTGGTCAATTCCGCAAATCCATGACGAGAAAATTCTGACCCT

GGCCAGGGAAATGGGAACGGACCCGGTCTGGGCCAGGATGCTTCTTGGCAGGGGGTTTGATGGCGGATCCGCCAGGCGTG

CGTTTACTTCTCCCCAACCTCCAGGTCCCGACTCTCTGGGGGACTTTACCCTTGCGTTTCAAATTCTGCTTCATGTTCTC

GACACAGCCTCCCCCATAGCGGTCTATGCCGACCTTGATGTTGACGGTGTGACCTCCGCCACCATGTTTTCAAGGTTTTT

GAGGCGAAGGAAACACCCCTACATCACCCTTCTGGTCCATAGAAACGACGGCCACGGGTTAAAGAGGGAGCGGCTTCTGG

CCCTGATCTCAAAAGGGTACCGGTATCTTCTGGTTGCGGACATGGGAACATCCGATATCTCCCTTCTCTCCGAAATGGAA

ACGCTTGGCCTCAGAACGATCGTTGTGGACCACCACCTGTTAAGAGAGTCATGGCCAGAAAATCTTGCACTGGTCCATCC

TTGTGGCCGATCCTGCCTGACTGCGGTCGGGAGCCTTTACGTCCTTCTCGCACCCTTTTTCCATCCGGACGAAGAACGGG

AAATGGCCTTCCTTGCCGGTCTTGCCGTTCTTGCCGACCGAGCACCGCTGATGGATGGAAACCGTTACTTTATAAGAGCC

CTCAGGGAGCAATCCTGCCTCGAGGGATTTCCCGGCATCCGCTCCCTCCTGAGAAGACGGCTTCAGCGCCGGGTTATGGT

CAACGACTATTCCTTCCTGGTCATCCCGGCGATCAATGCGCCGGGGAGAATGTCCGATCCCTATCCGGCGTTCGAACTTC

TGATGGCGACAACGGAGTCCGAAGGGGAGCGGCTTGCGCAGATCGTCCTTGAAATCAATCGGCGCAGGCAGGAAACGGAG

TGGTCGCTCTACGAAGAGATCCGAAAAGTATGGGACGGATGTCCGGTTGTCTTCCAGCCGGGGTGGGCCCCCGGTATCAT

GGGCCGGATTGCGCACAGGCTCTGTGAAGAGTTCAATGTCTCGGTCTTTGTCGCCACCCTGACAAAAGATGGGGATGTTC

GGGGTTCTTTGCGCCTCCGGGGAGGAGATACCCTCGATACCGTTCTCAAATGTCTTGAAGGTCTTCCAATTCAGGGTGGA

GGCCATGCCAGGGCGGGAGGACTGGGGTTTCCCATCGAAATCCTGGACAAGGTGAGAGGAAAACTCTCCTCCCAGTTAAA

CAAAACAGAAGACACTTCTTTTGACATGAATCAGGCTCTTGAAATCGACGCGTTTCTCCCGGCCTACTACCGCTCGACCT

CCTTCTGGGAAGGTCTTGGAGAGCTCCTTCCATTCGGTGAAGGGTTCCATCCCCCCAAACTTGGAGTCAGGAATGCGCTG

ATTGAGCGCATTGAAGATGGGAGAGGAAGGACGGTTCTGTGTCATTTCAGGTGGAAGCATGGGCTGGAACGTGCAGCGAT

CAGAAGCGGACTTCAGGTTCCTCCCTCGGGAGAGCGGGTGGATCTTGTCATGACTCCCGAACTAGTTGGAAAGGGGGATA

GGGTAGAGCGGCATTTCTCGATCTGCTCTTATCGCATCCATGAA

>ksgA LfeRT32a_1002 Dimethyladenosine transferase

ATGGAAAAGATGATGATGACCCCGCCCCACCGAGCCAGGAAAAGTCTGGGGCAAAATTTTTTGACCGATCCATCAATAGC

TCAAAAAATTGCCGATTTTCTTGATCCTGGAATTCCAAGGGAAACACCAGTGATCGAGATCGGTCCCGGAAAAGGGATTC

TCACGAAGGCTCTTTTGAGCAAAACATCCAATCTTGTTCTTCTGGAAAAGGACCACGACCTTCTTCCTGCCCTCACCGAA

CGGTATGGATCCAGCCCGGGAGTCCGGATTATCCAGACCGACGCACTCGAATATCCGTTTGGAGAGGGAGAGGATTACCT

TTCACCCCCTTTCTCCGGAGGGGGCTACCGGATCGTATCCAATCTCCCATACAATATATCCGTACCGTTGCTTTTCCGTT

TTTTGTCGAGTCCGAAGCCGCCCTTGGAAATGGTTCTCATGTTCCAGCGTGAAGTGGCCAGAAGGATCGTTGCATCTCCC

GGCTCCGATGACTACGGTCATCTGTCGGTGGCAATGGCGCTTTCGGCTTCGTCAAGAAAAGTTCTGGACCTGAAACCCGG

ATCGTTTTTCCCGGCACCCAAGGTTCACTCCTCTGTCGTTGTGATACGACCAAGAATATTCGACACCAACCAGGAAAGAG

AGCAAACGAGGCAAGCCCTGAATTTGTCAAGGAAACTGTTTTCCTATCGGAGGCGGACGCTCCAGAATGCGGCCCGGCTG

GCTTTCCCGGAAATACCGAAAGGGTATTTCGAGGAAGTTTTCAACATATTGGGAATCCCCCCGGAGAAAAGGGTTGAACG

ATTGCTTCCGGAAGAACTGTTGCTCCTGTCGACAGAAATACACAAAAAAGGACTGGAACAAAACTTCACA

>prmA LfeRT32a_2969 Ribosomal protein L11 methyltransferase (L11 Mtase)

ATGAACGAAAAAGGGTATCGCTTGCTGAAGGTGGACGTTCCTTCCGGAGAAGCCCAGAGTGTTGCGGACTGGCTGGTCAA

GAAAACCAAGCTTCCTGTCCTTGAGCAGGAAGTGTCGGGAATCGTCCGCCTGACGGCCTCTCTTGAGCGTCTTGACGGTC

TTGAGTCTCTTCTGGTCCGGGAGTCGGTCAAATCCATGGGAGCGATCGAGGCAACCGATGAGATCCTTCCGGGAGAAGAC

TGGGAGGCGTTGTGGAAAAGCCAGGGATTCCAGAGGTTTATGGTCAATCACTGGCTTTCGGTCATTCCCGAATGGGATCT

GGCACCAAAACCGGCGACTCCTTTCATAAGGATCCATCCGCATCTGGCCTTTGGAACCGGACTTCACGAAACGACAGGGA

AGTGCCTCTCCCTTCTGGTGGATCACTATCCCAGGGTTCCTGCTGAAGGTGGGAGGGTTCTGGACTTTGGGTCGGGTACG

GGGATTCTTGGGATCGCGGCACTTGTTCTCGGGCATGGGGATTTTCTGGTTGCCGTCGACAATGATCCCCTTGCGGTTGA

GGCCACATCGGAAAACATCATGCTGAACGGTCTGACCGGGTTTTCCAGAACGGGAGTGTCGCCCGATACTCCGTCGGAGG

GTGCCTTCTTTGGAAATGACGGGATGTTCCGTCTGATCATCGCGAATGTGACCGGTGGTGTGCTTCTCCACTGGATTCCG

TTCCTGTGGGATCTTCTCGAGCCGGGAGGAGCGATGGTCCTGTCCGGTATCTCGACGCGCGAGCGGAAGAGTGTCGAGGA

GATTCTTCCGGCCCCCAGATCTTTTCACAAGGCACGAAAGTTCCATACATTCTGGCTTAGAAAGGCCGACTTT

>mod LfeRT32a_2137 Type III restriction-modification system StyLTI enzyme mod

ATGGAAGAAGCAATCAATGATCTCAAAGCCCCATTTTCAGGATCGGTCACCGAAGATCAGGAGTACTCCCGCACTATTCC

CGATCCGGTACACCGGCTTTATACCGGAGACAACCTTCCCGTTTTAAAATACCTTCTTGCAGAAATTCCCCAGGGGGTCA

AGCTGATCTATATCGACCCCCCTTACAACACAGGAAGCATTCTGAACTATCGAGACCGATTTTCCGTCCCCTGCAAAAAG

ACTCCCCCCTTGAAAAAGAATACAGACCTTGATGGGTGGGTCACCAATCCAGCGACCGATCATAGCCCCTGGATGAGCTT

CATGTTTCCAAGGCTTGCCATTGCAAGGCAGCTTCTCCGGCCGGACGGGGTGATTTTCACAAGCATCGATGACCGGGAGT

TCCCGAGACTGAAACTTCTGATGGACTGGATCATGGGAGAGAAGAATCATGTCGGAACCATTGTCTGGCGTAAAAAGGTG

GTCAGGGGAAGAGGCAATCGTCATATTATTCCCCAGACGGAATATATACTTTGTTATGCTAGGGATGTTTCCATGCTCCC

ACCCTTTCGCGAGCCTTTGACTGATGACATGATTGATTCCTATACCAAAAAAGACGCGTCTGGCCCCTACAAGGAAATCC

CTCTCGCCAAATCCGGAACAGCCCATTCCCCCCGCCCCAACCTTGTCTACCCGATTACGGCACCTGACGGATCCACCATT

CACTGCCCGACTCACCAATGGAGATGGAGCGAAAAGACATTTCTGGAACGCCAGAACGAGATTATCTTTCGGAAGACCAG

AAAAGGAGTCTGGAGAGTCCTGACCAAACAATATCTGGAAACAGTCGATGGTCTTCGATTTCGCACACCCACATCGCTCT

ATGACAAAGTCACCACCACCGATGGAACAAGAGAGCTTCGGAGCATTTCCGGAACGGGAAACTTTGACTTTCCAAACCAT

CAAGAC

>FHIT LfeRT32a_2019 Diadenosine tetraphosphate hydrolase

ATGGAATATATTAAGAAAAATGGTGAGGTCACTCCGAAGAGCGACTGCATTTTGTGTGATCTCGGAAAGTCCGGCGTAGA

ACGGGATCGTCTTGTCCTCTACTCCGGGCAGGACTGTTACATTGTCCTGAACGCTTTTCCTTATACAAGCGGCCACCTGA

TGGTTGTTCCCTATCATCATGCGGGAACATTTTCCGAACAGTCCCAGTCAACACTCAGTGAGATGATGTACCTTTTAAAA

CATGCGGAACGGATTCTCCTTGAGGAGTATCAGCCGACAGGTTTTAACATGGGTGTGAATATCGGGCGTACTGCAGGTGC

GGGGATCCCGGGACATCTGCATTCTCATCTTCTGCCAAGATGGGATGGCGATACGAATTTTATGACTTCCATCCATGAAG

TGCGTGTGTTGCCTGAATCCTTGTTGATGACTTATGACCGGCTTTTGCCTGCGTTTTCCCGTGTGCCCCAGATGGACAAG

TGTGCCGAGAAAGGCGAA

>ydcM LfeRT32a_1324 Putative IS 1341 element transposase

ATGCAGACCGGCAACCGCTTTCGCGCGTATCCCACTCCCGCCCAAGAGTCGATCCTTCTCCAGTGGATAGGACATCAGCG

CTTCATCTACAACGCCAAGGTCTCCGAAGACCGGTACTACCGGACCTTCGCCCGAAAAGCGGTCTCTCTTTCGGGAACGG

CCGTTCCCATCGACCAGGAGTACGCCCGATTCATCGGAGAGAACACCCCCTGGCTCCGGGAGGTTCCCTCCCAGATACTC

CGTAACGGGGCGGTTCGGTGGAAACAGTCCTATACCAGGTTCTATGCCGGACTTTCCCGAAGACCGGTCTTTCAGAGAAA

AGAGGGACGGCAATCGGTCTGGATCACCTCCGAGCTTTTCTCCTTTCGATATGACGAGAAGACACACACCGAGGAACTTG

TTCTCGGGACGAAGAAGTTCCCGGTGGGGGTTCTGTCCTTTACGGCCCATACTCCGTACTCCCGCCCTGCCTCACTTCAC

GTCTCGGTCGAGGCGGGAAAGTGGTACGTCTCCTTCTCCTCGGACGATGGGCAGGCCGAGCCTAAAGAGGAAGACACGAT

CGCCTGGCTTCGGATGTTTACGGAAGAGGAACTCCGGAGCAAAGCCGTTGGATTCGACCGGGGTGTGGTGACGCCGGTGA

TGGCGAGCGATGGGGGAAGGATCGATTTCTCCTCCATCCAGAAGATCCGGATGGAGAAGAAGGAGCGGAGCCGGAGACGG

TGGCAACGAACACTCTCCCGTCAGCAAAAAGGATCGCAGAACCGAAAAAAGACCAGACAACGTCTGGCCCGGACTTTCGA

ATACACCAAAGACGTCCGGAAAGATGTGGTCCACAAGGCCACCCATGCCATAGCCGAAGGTCCTGACCGAAGCCTCTTCG

TTGTCGAGGATCTCAGGGTCAAAAACATGACGAAAAAGCCGGAACCCAAAAAAGACGCGTCCGGCAATTTCCTCCGGAAC

GGGGCACGTGCCAAGGCCGGTCTCAACCGGTCGATCCTGTCGTCCTGCTGGGGCCTGTTCGTCCTCTTTCTTTCGTACAA

AGCCCGACGGAACGGGAAGCTCGTGATTAAGGTACCACCGCAATTCAGTTCGCAGGAATGTGCCCACTGCGGGCACATTC

ACCCGGACAACCGGCCTTCGCAGGCCGAGTTTGTCTGCCAGCGCTGCGGACTTACGGATAACGCCGATCAAAACGCCAGC

CGCGTGATCGCCCAAAGGGGGATCCGACTTCTTCTGGAGGGGACTATTCAGAAGAAACGGGTCCGGCGGTGCGGGATCGG

AAAAGAGAAACAACCAGGGCCGGAACGGTCCGAAGTCAAAGCCTCCGGAGAGGAGCATAAGACGCAAAGGCCCAAACGCC

TTCGCGCATCCTCGGCGAAAGAGGAACATCCGCTCGTGAGATCGGAAACCCCTCCTACAGCGCAAAGCGCT

>orf_3972 LfeRT32a_3972 Hypothetical 45.4 kDa protein in snaA-snaB intergenic region

TTGAATCCTCTCCCGGTGGGGATCGATGTGGGGATCAAAACCTTCGCCTCCCTTTCCGATGGAACAGCCATCGAAAACCC

GAAGTTCCTGAAACGATCGTCTAAAAGACTGGCGCAGGCTCAGCGGAAACTGGCTCTTCAGGAGAAAGGATCTCCTGAAA

GAATAAAAGCCAAAAAGGTCGTGGCAAAAGTTCATGAGCGCATTTCGAACCAACGCTCCGACTTTGCCCACCAGGAATCG

ACGAAGATCGTGAATCAGTACGGACGGATCTTTGTTGAGGATATTACGGTCAACGAAATGAACTCCCACCGATGCCTGAA

CCGAAGCATCCGTGACGTGGCGTGGTCGCAATTCTTCTCCTTTCTCTCGTACAAAGCTGCAAATGCTGGTCGGGAGCACA

GGAAGGTGGATCCGGCCTATACAAGCCAGACCTGTTCCTTCTGTGGCCATCGGCAGAAGATGCCGCTGTCCTGCCGGACG

TATGTCTGTCCGTGTTGTGGAATGGAAAAGGATCGGGACCACAATGCCTCCCTGAATATTTTGAGACTCGGGCTTTCGTC

TCGGGGT

>yncI LfeRT32a_3445 putative transposase yncI family 11

ATGCGGGCTCACCACTACCTCGGATTCCGGACCATGCCTGGAGAGTCGATCCGCTATGTTGCCCTTCTCGACGGGGAGTG

GGTCGCCCTCCTGGGTTGGGGATCCGCCGCCTGGAGCAATGGATACCGGGACCGGATGATCGGATGGACCACTCCCCAGC

GGGCCCGGCGCCTGTGCTATCTCGCCAACAACCTGCGCTACCTCATCCTTCCGGGGGTCCGTCGTCCTCACCTGGCCTCC

AAAGTGCTGGCTCTCTGCATGCGCCGGCTTTCCTGCGACTGGGAAGAGCGATACGGCCATCCCATCCTGTTCGTCGAGAC

CTTCGTCGATCCCTCCCGCTTTTTGGGAACCTGCTACCGGGCCGCCGGCTTCCGCGATCTTGGCGAAACCAAAGGGTACC

GAAGAAACGCCGGACGATACGACTACCACGGAGAGGTCAAGCGCATCTTCGTCCGGCCCCTGAGAAAGGATGCGCTTCGT

CTTCTCTCGTCCCCCGACCACCTTCCCATTTTCCAGACAAAGGAGGCTCGCGTGCCCGTCAAGGCTCTCAGCCAAAAAGA

CATCCAGTCTCTCATGGACGATCTGTCCAGGGTGACGGATCCCCGGAAGCGGCGGGGGATCCGTCACTCCCAGACTTCCC

TCGTCGCCACCCTCGTGTGTGCCATTCTCTCCGGCGCCTGCCATACGCTCGCCATGGCCGAGTGGGCGAAAAACCTTTCG

AACGCCCTCAAAAAGCGCCTGGGATACCGGAGGCATCCCGAGACAAAGGTCTGGATCGCGCCCTCCGAACCCACCCTGCG

CCGCGCCCTCCAGTCGCTCGACGTCCTGAAGGTCGAGCAGGCGATCTCGGGCTGGCTCACGCGGATCCTGGCCAAGTCCG

GATTGGCCGAAGACGGGAAGGTCCTCTCCGTCGACGGGAAGACGGTCCGGGGAGCCTCGAAGGCCGAGGGCGGTCAGAAG

GTCCACCTCCTCTCCGCCTTCCTCCAAAACCGGGGGATCGTCGTGGCCCAGAAGAACGTGGACGAAAAGACCAACGAGAT

CCCCGAACTCCGCGCCCTTCTGGCTCCGATGGAGATCTCCGGTCAGATCGTGACGGCCGATGCCATGCACACACAAGTGG

AAACGGCCCGGTTCATCACCGAGGACAAAAAGGCCGATTATGTCTTCACGGTCAAAAAAAACCAGCCCACCATGTTCGAA

GACATCGAAAGCCTCCCCTGGGAGGCTTTTCCCCCCTCGGCAAACGGCCTCGACAATCGA

>orf_3153 LfeRT32a_3153 Transposase

ATGCGTAAAACATTCAGGTATCGGCTGTATCCGACCCGCAAACAGGAAGCTCTTCTGAATCGACAATTGGAAGAGTGCCG

CTGGCTCTACAACCATTTTCTGGAACACAGAAAGAACGCCTGGGAGTGGTACGGCGTTTCCCTGTCCCATTACGGACAGC

AGAATACCCTTCCCTCCCTGAAAAAGATCCGTCCTTCCCTGGAGATGGTCTATTCCCAAACGCTCCAGAACGTGGTTGTC

CGCATCGACCTAGCGTTCCAGGCGTTTTTTCGCCGGGTCAAAAGCGGTGAAGACCCCGGCTATCCCCGGTTCAAGGGAAA

GGGGCAGTACTCTTCCCTGACCTTTCCCCAGTGGAATAGCGGATGCGATCTGACGGGAAAGGGGCTCCGCCTGTCCAAGA

TCGGAGTCGTTCCCGTCGTGCTCCATCGCCCGGTCGAAGGAAAGATCAAGACCTGTCACATTCTCCGGTCTTCTACCGGG

AAATGGTGGGTGACTCTTTCCTTCGAGACTGTCCAGGAACACGTTTTTCCGTCCAGTCCTCTCCCGGTGGGGATCGATGT

GGGGATCAAAACCTTTGCCACTTTTTCGGATGGAACGGAGATTGGAAATCCGAAGTTTCTGAAACAATCCGCAAAGCGAC

TGGCCCAGGCGCAAAGAAAATTGGAACTTCAGGAGAAAGGATCTCCTGAAAGAACAAAGGCCAAAAAGGTCGTGGCAAAA

GTTCACGAGCGTATTTCGAATCAACGCTCCGACTTTGCCCACCAGGAATCGACGAAGATCGTGAATCAGTACGGACAGAT

CTTCGTCGAAGACATCACCGTGAATGAAATGAACTCTCATCGCTGTATGAACCGTTCTATCCGTGATGTGGCGTGGTCGC

AATTTTTCTTCTTTCTCTCGTACAAAGCTGAAAATGCTGGTCGTGAGTTCAAGAAGGTGAATCCGGCCTACACAAGCCAA

ACCTGTTCCTCCTGTGGTCATCGGCAGAAGATGCCGCTGTCCTGCCGAACGTATGTCTGTCCGTGTTGTGGAATGGAAAA

GGATCGGGACCACAATGCCTCCCTGAATATTTTGAGACTCGGGCTTTCGTCTCGGGGT

>dfrA LfeRT32a_2496 Putative dihydroflavonol-4-reductase

ATGGGAACAGGAAAGCGAACAGCTCTGGTGACCGGGGCGACTGGGTTTGTCGGCTCACATGTGGCGCAACTTCTGCTCGA

AGAAGGATATTCGATCCGCTGTCTGGCAAGGGAAGGCAGCGATAAAAGAAATCTTCCCGGAGAGAGTGAGCATGTCTCCT

GGGTTACGGGAGATCTCCTTGACCCATTGTCCCTTGTGCGGGCGCTCGACGGGATGCAGGAGCTGTACCATGTCGCGGCG

GACTATAGGCTCTGGACCCCCCGCAAAGGTGAGATCATCCAAACCAATGTCGAGGGAACCAGAAATATCCTGGAAGCTTG

CAGAATTTGCCGTCCGGAGAAGATTGTCTACTGTTCGAGCGTTGCGGCATTGGGGACTCGTTCAGATGGAATTCCCATTG

ATGAGACGATGGAAGTTGACACAAAAACGCTGATCGGGGAATATAAGCTGTCAAAGTATCTTTCCGAGCAGTTGGTGTTG

TCCTACGCCGGGGGATGCGATGGGGGAGAGCCTCTTCCCGTCGTCATCGTTAACCCCAGTGCTCCAATCGGGGCCGGAGA

TATCAAGCCAACTCCCACTGGGCGGATTGTTCGTGACTATATGCGGAAAATGATGAAGGCGTACGTCAGGACAGGGCTGA

ATGTCATCCATGTCCGGGATGTGGCCAGGGGGCATCTTCTTGCAGCGCAAAAAGGTGTTCCCGGGCAGAAGTACATTTTG

GCGAATCAAAATATGCAACTGGTCGAGATATTCCGTCTTCTGGCAGGAATAACGGGGGTTCCGGCTCCAAAGGCCGAGAT

GCCAAGATCTCTCCTTTACCCCGTTGCCATCGTTTCTGAGGGAATCTCCCTCTTGACAGGCCGTGAGCCGCTTGTTCCTT

TCGATGCGGCGCGTATGGCCCATAAAATGATGTTTTTCTCAGGAAACAGGGCTGTAAGGGAATTGGGCCTTGTTTTGACG

CCGGTGGAGAAAGCTTTTGATGATGCTGTCCGGTGGTTTTCAAAAGAGGAGTATATGGGGGCCGGGACCTTCCCCGGA

>orf2555 LfeRT32a_2255 Small orf for hypothetical protein

ATGCGGGGACAGGAGTTCAATACAAAAATAAATCCCCTCATTTCGCTGCTTTCTGAAATTGTCATCCTTCCGCCACCAGT

CTATTGGGTGGATTTCGGCCCCGCAAGCGAAAGGCCCATTTTGGCCAAGATGTGCGAAGTCGGT

>orf2556 LfeRT32a_2556 Small orf for hypothetical protein

ATGGACTCAAAAGAAGCCTACAAACAAAAGCTGAATGCCCAAATGAAGGAATGGAGTGCCCAGATCAATCTTTTGAACGC

AAAGATTGAAAACAAGGGAGCAGACATGAGGATCAAGTACGCAAAAGAGCTTGATGCCGTGAAAGCAAAGCAGGAAGAAG

TCTTGCAGAAAATCAAGGAACTTGATGAAGCGACGGGGGAGAATTGGGAAAAGGTCAAGAACACAGCAGACCAGATCCTG

GACGATTTGAAAACCGGCTTGAACAATGCGCTCTCAAGGCTCAAA

>orf2557 LfeRT32a_2257 Small orf for hypothetical protein

ATGCCTATGGAGAAGACTCTTCAGGATCTGGTGCAGAAACGCGATCGGTTGAAACAGGCTCTGATACAAATCGAGGAGAT

GCGGCAAGGATCTCTGTCAGAAAGCTACCGAAAATGCGGAAAGCCCACCTGTCACTGCGCTTGGGAAGGAGATCCGGGAC

ATGGTCCCTTCTATGTCCTGACGAGAAAGGACGCCTCCCAGAAAACGGTTGGACGAGCCATTCCCATTCGCTTTGTCGAA

GTGACCCGGGAACAGATCAAGGAATATCATCGTTTCAAGGAACTCTCCAAAGAACTTCTCGAAGTTAACGAACAGATCTG

CGACCTCAAGCTGAAAAATTCGGACGAACCTTCCTCCGAATCCGTAAAAAAAAACACTCGAAACCGACCTCGTCCG

>orf3039 LfeRT32a_3039 Putative oxidoreductase

GTGCGAACAAAAACAGCGATCGTAACAGGCGCATCCTCCGGTATCGGAGCAGCAACCGCAAAGGCTCTTTCTGCTGCCGG

ATACCACGTCATTCTGGGGGCCAGAAGGGTCGACCGGGTGGAACGGCTCGCCGGAGAAATCGGCGGGGAAGGCTATGGCC

TCGATGTCACCGACCCCGCTTCAGTCAGGAGTTTTATGGACAGATTGCCTGACAGGATCGACCTTCTGGTCAACAACGCC

GGTGGGGCCTTGGGTCTGGACCCGGTTATCTCCGCCGACGAAGATCGATGGATTGAAATGTTCCAGTCCAATGTCATGGG

AACTCTCAGAATGACAAAAGGTGTCTTCCCGCGCCTTGAGCGCTCGGGAGACGGGAGCCATATCGTCAATATCGGGTCGA

TTGCCGCATGGGAGACCTATCTCGGCGGAGCGGGTTATACCGCCGCAAAGCATGCGGTCCGGGCCCTCACCGAAACATTG

AGGCTTGAGTGGCTCGGGTTGCCGATTCGCGTCACGGAAATTGACCCCGGGCTTGTCGACACGGAATTTTCTGTTGTGCG

CTTTTCCGGAGACGAGATACGGGCCAGAAAGGTCTACGAGGGGATGACTCCCCTTTCCGCCGGGGATGTTGCCGAGGCCG

TTGTCTGGGCAGCACTGAGACCTCCCCATGTGAACATCGACCAGATCCTGATCCGGCCGAGAGATCAGGCCCGTGCGGAC

AAGGTTTCAAGGGTCCCT

>orf1736 LfeRT32a_1736 HD superfamily phosphohydrolase

ATGTCCAGGAACGAGAGCCTCCACCCCAATCTTTCCCATCTTTCGGATGTTTCTCTTTTTTCCGATCCGATACACGGTTA

TATTCCCTTTGTATCCAAGCCGGTTCGACCTGATCGCTCTTCTGAAAGGGATCTGATCGATTCCGACTGGATACAGCGTC

TTCGTTCGATCCTGCAGCTTCAAAGCGCAAGGCTGGTTTTTCCATCGGCGGAACACAGCCGGTTCGTTCATTCCCTGGGG

GCGATGCACATTGCCGGGCGCTTCGCCGCGCATCTGTATCCCGGATTCAATGAAAGTTTCCCGGACGCGCTCTCCCCCTC

CCTGTTTGAATCCCTTCTCCGTGTGTCGGCACTTTTCCATGATAGTGGCCATGGTCCATTCTGCCATTTTTATGATGACC

ATGTTCTCAAGGATCGCTTCGGAATCTCTCATGAACGGCTGAGTCAGGAGATTATCCGGGGGCCGATGAGAGGGGTGATT

GAAAATCTTGACCGGTCTCCATCGGGGCACTTTTTGTCCGGTGAGAGCCTCTCTGCCGATTGGGTGGCCTACCTTGTCGG

AAAGGGGAGCCGACAGGATCTTTCCATTCCTGAGCCACCAGAATTCATCAAGCGGCTGAAGCCACTTTTTTCCGGTCTGT

TTACGGTCGACAACCTTGATTACATCCTCCGGGACTCATACATGTGCGGCGTTTCCATTGGGCCGATTGATCTTGATCGG

ATCCTTTTTTATACCCGATTTGAGGAAAAGTACCTTTCTTTTCACCGTGCGGGGTTAGGTGCCCTTGAGATATTCCTCGT

GATCCGCTCTTTCATGTATCAGCAGATCTACTTTCACCGGACTACCAGGCTGTTCGACATGTCTCTCGAGGGGGTGCTGG

GGGAGACGGTCGACCTTCTGTCTCCGGGGAATCCTCTTGAGCACCTGGATGAGTATTTGTGTCTTACCGATCACTTTCTG

ATTGAGTCGGTCCGTCTCTGGAGGAAGGATCCCAACCCGCTCAGGAAAGCTCTGGGGGAGAAGTGGTCGTCGTTTCTTTC

GAGAAAGAAACAGTGGGAGCTTGTCTATGAGTCTGAGTGGCGGGCAGAGAGGGTCCAGGCGCTCTTTCCTGAAGAGCTTT

CAGTCAGTTCCCGGGAAAAAGAGATTGGCTTGTCGCTGGGGTTGTCTGAAGGGGACTTGAGAATTGATATTGCCTCAAGG

GATACCCGCCCTGAAAATCCTTCTGACTTTCAGAAGAGGGGCCTTCTGGTTTGGGATCCGCTTTTAAAGACGCTTTCGAG

CGACCCGATGGTAGATCTTCTCCTTTCTCTTCCGGTCAGGCGGGTGATGGTCAGGGCTTTTATCCAGAGACGAGAAGGGT

CTTGCTCTGGAAGCGGGCATCTGAATCTTGTTGTAAAAGAGTCATTGGAGAACCTTGGG

>pta LfeRT32a_3388 Phosphotransacetylase

TTGGCCGGCAACCTTTCCTGCCCCACAAGCTTTCTTTCGATTCTCGAAGAAGCCAGAAAGGCACCTCCCCTGAGAGCCGC

AGTCGTCAATGCATCAGATCCACTGGTTCTCGAAGGCGTACTCGAAGCCATGAAGGAAAACTTCATACACCCTGTCCTTC

TGGGAAACGCCTCGGTGATCAATGATTTTTTCAGCCGCAACCCCGCCCCTGTTCCCGTCGAGATCATTGAAACCGGCTCC

GACCGTGAAGCGGCCGATCAAGCCGTTTCTCTTTTCACAGACGGCTCTGTTCAGATCCTCTTAAAAGGCCATATCCATAC

CGATACGCTATTACACTCGTTTATTAAAAAAATCCCGCTTTCCACCAGAATTTCCCATGTCTTTGTCGCAGAGCTTCCCT

CGTATCCCAAACTTCTTTTTGTCACCGACGCCGCGGTCAACATCTCCCCGGATCTCTCCAGCAAAATGTCCATCACCCAA

AACGCAATCGATTTTGCAATTCTTATGGGCATTCAGACGCCACATGTTGCCATTCTTTCCGCCATAGAGACCGTCAATCC

GTCCATACCGTCAACAATCGATGCCGCATGTCTTTCAAAAATGTCGGAAAGGGGGCAAATCCATGGAGCAGTCATTGACG

GTCCACTTGCTTTCGATAATGCCATTTCCGCCGAATCGGCCAGGGAGAAAGGGTTCGAAAGTCCTGTTGCAGGAATAGCC

GATATTCTTGTCGTACCCGACCTGGTGTCAGGAAACATTCTGGCAAAAAATCTCGAATATTTTGGAGGAGCCACATTGGC

AGGAGTCGTTATTTCGAGCCATTCTGTTCCGGTCGTACTGACATCGAGATCGGACCCTCCCCGATCCAGACTTTTGTCAA

CAGCCATAGCGGTCCTGGCCCATGAACGGCTTGCCCGGAAAAAACTCGGCAACATA

>kdpB LfeRT32a_2642 High affinity ATP-driven potassium transport

ATGAAAAAAAAGGTAATGCCAGATTACGGAATTTACATGGAAGCAATCATCCAGTCACTTTCCATGCTGAATCCGGCAAA

GCTCTACAGAAATCCTGTGATGTTTATTGTTGAAATCGGAAGCGTCCTGACGACATCGATGGCGATTTATGCCGGCGTGA

CCCATTCGGGTGAAGCCGGGTTTGGAACGATGATCTCCATCTGGCTCTGGCTTACGATCGGGTTTGCCAATTTTGCAGAA

GCGCTGGCCGAACTTCGGGGAAAAGCCCAGGCATCGAGCTTGAGGTCCACACGTGCCGAGACGACCGCCAGGCTTGTCAC

ACCGGTCGGGTTCTCCCATGTTCCCGGAAGCGAACTGAAGAAGCAGGACCAGTTTTTAGTCGAGGCAGGAGAAATCATTC

CAACGGATGGAGAGATCCTTGAAGGAGTCGCAAGCATTGACGAATCAGCCATTACCGGCGAATCGGCTCCGGTTATCCGG

GAGTCCGGAGGCGACCGGTCCGGCGTCACCGGAGGAACGAGAGTGCTCGCAGGAAAAATCCGGGTCTCTGTCAGCAACAA

TCCGGGAGAGACCTTCCTCGACCGCATGATATCTCTCGTCGAGGGAGCCAGCCGGCAAAAGTCTCCCAACGAGATCGCCC

TGACGATCCTGCTTGTCGGACTCACCCTGATCTTTCTTTTCGTCGTGGCGGCGATCCCGGCATTTTCTTCCTATTCGGGA

ATCAAGTCTTCCCCGACGATTCTCATCGCTCTTCTGGTCTGCCTGATCCCCACAACCATCGCCGGTCTTCTCCCTGCCAT

CGGAATCGCCGGAATCGACCGCGCATTTCGCGCCAATGTCATCGCAAAGTCCGGAAAAGCGGTTGAGGTTGCCGGAGATA

TCGACATTCTTCTTCTGGACAAGACAGGAACCATTACCTTCGGAAACAGGCAGGCCGTCCAGTTCATTCCCGCAAAAGGG

GTTCCCGAAAAAGAGCTGGCCCATGCAAGCTGGCTCAGCTCGTTGTCCGACGAGACCCCGGAAGGGAAAAGCATCGTTGC

ACTGGCCGAATCCCTGATCGAATCCAAAACGCCTCCCGATAGACTTGTTCCGATTCCGTTCACACCCGAAACCAGAATGA

GTGGAGTCGACAGCGCGGACGGAGAAATCCGCAAAGGAGCGGAAGACGCGGTGAGCACCTATACCGGAGCTCCTTTTTCA

GCCGACATCCAGGAGATTATTTCCAACATTGCCAAATCCGGTGGAACGCCACTGGTGGTTGCAAAAAACAAAAAACCCCT

TGGAGTCGTCCATTTGAAGGACATCATCAAGCCGGGCCTCAAAGAACGGTTTGCCCGACTCCGAAAAATGGGCGTCACAA

CGGTCATGGTCACGGGAGACAACCCTCTCACCGCGAAAGCCATTGCCGAAGAAGCGGGGCTCGATGACTTCTTTGCCCAG

GCGAAACCGGAAGACAAGATGGCCCTGATCAGGAAAGAGCAGGAAAAGGGAAGGCTCGTCGCCATGACGGGAGATGGCAC

AAACGATGCCCCGTCACTGGCTCAGGCGGACGTCGCCCTTGCCATGAACTCCGGAACGCAGGCGGCCAAGGAGGCCGGAA

ACATGGTCGACCTCGATTCCGATCCCACCAAAATCATTGAAGTCGTTGAGATCGGAAAGCAGCTTTTGATCACAAGAGGC

GCCCTGACAACCTTTTCCATCGCAAATGATGTGGCCAAGTACTTTGCCATTCTTCCGGCAATGTTCGTTGTGGCTTATCC

CCAGCTGAAGGTGTTGAACGTCATGGGACTTGCGACACCCGAATCAGCGGTGACCTCCGCCATCATCTTTAACGCGCTGA

TCATCCCGGCACTCATTCCCCTCGCCTTAAAGGGTGTTTCCTACAAACCGGTCGGAGCTTCGTCCCTCCTCAGGAGAAAT

CTCCTGATTTATGGGCTCGGGGGAATCATCGTCCCTTTCATCGGGATCAAGCTGATCGATATGGCACTTGTGCTATTGCA

CTTTACT

>kdpA LfeRT32a_2643 High affinity ATP-driven potassium transport

ATGATTGCAAATGACTGGATTCAAATTTTTATTTCCCTGGCTTTTGTTGTTTTAATGGCGCCGCTGGTGGGGCGTTATCT

GGCCTGGATCTACCAGAGCCAATCCCTCTCCCTCGAGAAGGGACTCTACCGTCTGCTGAAGATCGACGCCGACAAGGAAA

TGACCTGGAAAGAATACGGTGTGGCCATTGTGGTCTCAAACGGATTTTTCTTTGTGGCGGGATTCCTGGTTCTCTGGGCA

CAGGCCATTTTGCCCCTCAACCCCAGACACCTCTCGCCTCTCTCAGCCGAGGTCGCCTTCAACACAGCCGCCAGTTTTGT

CACCAACACCAACTGGCAGGCTTATGCGGGGGAGGCCCAGCTCTCAAACTTCTCCCAGATGCTGGCCATCACCTTTCTCA

TGTTCGTCGGCGCCAATACCGGCATGGCATCGCTTGTCGCCATCTTCAGGGGGTTCACGCGCTCCGGAACAACAATGATG

GGGAACTATTGGTCGGATTTCTTCCGGTTTTTTGTCCGGGCCTTTCTTCCCGCCTGTTTTTTGATGGCACTCGTTCATGT

ATGGCAGGGTTCACCCCAGACCTTCAAGACAACAGTGACCGCAAAGACGATGGAAGGAAGCCAGCAAACGCTGATTGTCG

GACCTGTTGCATCCCTTGAATCGCTTAAGCAGCTGGGAACAAATGGGGGAGGATTCTACAACGCCAACGGAGCGCACCCT

TATGAAAACCCGACCCCCCTGACCAATACGCTGGAATTTCTGGAAATGGGCATCTTTACCTTTGCTCTTCCCTTTTTCTT

CGGAAGGATGATCGGACGCCCTCGTCAGGGACAGGTTTTTTTCGCCGTTATTTTCTCTCTCTACCTCGCCGGTCTCGGCG

TGATCGAATACTCGGAAAAAATGCCCAATCCCCTCATTGCGCACCAGGCGATCTCCCAACAGATCACTCCGCTTCAGGAC

GGAGGCAATACTGAAGGAAAAGAAACGCGGTTCGGGATCACGCAGACCAGTCTCTTCTCAAATACAACCATCGCCGCCAC

CACCGGAGCGGTGGACGGAGCCATGGACAGCATGAATCCACTGTCCGTGCTGGTCTACCTCGTACATATGTTCCTAAACG

AAGAACCCGGAGGCAAGGGGGTCGGCTTTGCCGGTCTTATCAAAGAGGCCATCCTGGCCATTTTTCTGGCTGGACTGATG

GTGGGGAGAACGCCGGAATTTCTCGGAAAGAAGATCGAGTCAAAAGAAGTCAAGCTGGCAGTCCTGTCGCTCCTCATCAC

CCCACTTCTGGTTCTGGGGCTCACCGCTCTTTCTGTTGTCATCCCCGCTGGAAAAAGTTCGATGGACAATCCTGGTCCAC

ATGGATTCATGGAGGTTCTTTATGCCTTCGCATCCGGCAATGCAAACAACGGTTCCGCCATGGCGGGACTCAACGTCTCA

ACGCCCTACTACGCCATCCTGATCGGGGTTGAAATGCTCTTTGGCCGTTTCCTGCCGCTTCTGCCGCTTCTTGCGATGGC

AGGTTCCCTGGCCAGAAAAAAGACTCATCCGGAAACAGCCGGAACCTTCAAGACGGATACGACCCTCTTCGTCGTGCTTC

TTTTCGGGTTCATGGTCCTGTTTGCCGCACTGACATTTTTCCCGCCACTGATCCTCGGTCCCCTTCTGGAGCATCTGTCC

ATTCTCCATGGGATCTCATTC

>kdpC LfeRT32a_2644 High affinity ATP-driven potassium transport

ATGAAACTTCTTGGACAATCGTTTCGTGCCACCGTTTTACTCATGATCGTGTGCGGGGCGCTCTACCCTGCGGCGGTTAC

GGCGGTGGGCAACATTCTCTTCAGCCATCAGGCCCAGGGAAGTCTGGTGAAGGGCGGCAATGGCCAGGTTCTGGGATCCA

CGCTGATCGCCCAGGGCTTCAAGGATCCCAAATATTTTCATCCCCGCCCATCCGCTGCCATGACGCCGGATGGATCGGCC

GCCCAACCCTATGATGCGGCTTTTTCAAGCGCTTCCAACCTGGGCCCGGACAACAGTGCCGAGATCAAGGCGATGACCGA

TGCAGCCGCAGCCTATCGTCTGGAGAACCATCTTTCCCCGGGCTCCGTGGTCCCCATCGATGCGGTCACAGCCTCCGGAT

CAGGTCTCGACCCCGACATCAGCCTCATGAATGCCGAACTCCAGGCCCCCAGAGTCGCAGAAAGCCGCCATATCAGTCTG

GATCGGGTCAAGGAACTGATCAGGGAAAAGACCATCAAACCCCAACTGGGATTTCTCGGAACAAGCAGGATCAATGTCCT

GATGATCAATATCGCACTGGACAACGAACATCCAGCCGAAAAAACAAAC

>pstS LfeRT32a_3757 Phosphate transport ABC system

TTGTTTTATAAGGGTTCCGGGAAGCTTCCGGATCGGAAATCCGGGGGGTCGGTGAGAAATGTCATCGTTTTGTCATCGAT

CTGTCATGCGGATGTATCAGCGCTGTGGGAAAACTTAAAAAACATTTTCATGAGGAGGTTTACAATGTCCAAAAAAATGC

CGGGGGGGCTGTTTTCTTTGGCCGCGATAAAGTCTTCGGTATCAACGGTTGCTGCAGGACTTTTTGTTGCCACGGCCTTG

TTCGGCCCGGGGGTTTCATCGGCTTACGCCGGATCGGATCTGACGATCTCGGGTTCCTCGATGCTTTTTCCCCTTGAGCA

GGTCTGGGCGGAGGCTTACCAGAAATCCCACAAGGGAGTTCACATCTCTGTCGCCTCGACCGGTTCCGGTTTCGGCATTG

CCAATGCTGCCAACGGAAATATCTCCATCGGAGCATCGGATGCCTATCTCACAAAGACCTTCAGGAAGCGTTATTCCAAT

CTGGTCTCCATTCCGATCGCTTTCGACGATGCCCAGGTCATCTACAATATTCCCGGCATCGACAAAAAGACCGTCCTGAA

GCTTGACGGACCGACGGTTTCAAGAATCTACCTCGGCAAGATCCGCTACTGGGATGATGCCAGGATCAAGGCCATGAATC

CGGATATCTCATTTCCGCATACCAAGATCAAGGTGACCCATCGGGCAGACGCTTCCGGGACAACCTTTGTTTTCACGGAT

TACCTGAACCAGACGTCAAAGCTCTGGTATAACCAGGTCGGCCGGGACCTTGCCCCTGCCTGGCCTGTCGGGTCGGGATA

CAATGGATCCGATGCGGTGGTGGCTGCGGTCATGTCGACACCCGGTGCGATTGGATATGTCGGTCTGGGATGGATCAAGG

AGTATCACCTGTCTTCCGCGGCACTTAAGAACAAGTTTGGAAATTATGTCGTCGGAACCATCGACACGATCAAGAAAGCG

GGATACTCCGCCCTGAAAGATCCTGAATTTCCCAATGACTTCAATCGCTCGATCGTATGGAACCTCAAGGATCCAGGTGC

CTATCCGGATGCGAACTTTGAGTTCTGGATGGTGTCGACGAACCTCGACGGTGCTTCGATGAAGCAGGTCCGAAAGCTGA

TCTTGTGGGCTTTGGGTCCTGGCCAGGCCAAGAAATATACGGTGTCAAGCGGCTTTGCTCCCCTTCCGTTTGAACCGTTG

AAGCCCCGACTTACCCACATCCTCAACCGGCTTCTTCCTGGAAACAGCAAAAACGAAGTTTCTCCTGGA

>pstC LfeRT32a_3756 Phosphate transport ABC system

ATGGAAAAGACCAGGGACGAGAAGATGGAAAATGCCGAGATCAATGCCAGCGATGTGCTCCGCCAAACTCCTTCATCCGG

GGATGGACAGCTTGCTCAGGCTTCCCGACGCCTGATGAGAGGATCCCATCTTACAAGGGAAGACCGGATGTTCGGATGGA

CGCTCAAGGGAGGGGCCCTTTTTATTCTCATTCTCTTTCTGATTGTTCTCGGTGTTTGTTTTGTGACCGCCTGGCCATCG

ATCACGAAGTTCGGTTTCGCATTCCTCTGGTCCTCCGCCTGGAACCCCAGCCGGGAAATCTTCGGTGCATTCCCCTATCT

TGTCGGGACCCTTTGGGTGACCGGTATCGCACTTGTCGTGGCGATTCCCGTTGCGATCCTTTCGGCGCTGTTCCTTTCCG

ACTATGTTCCCAAATGGCTTGGCGGGATCGTTTCAACTCTTATGGAAATGCTTGCCGGCATTCCATCGATCGTGTTCGGC

GCATTTGGAATTGCCTTTGTCGTCCCCCTTCTCAGGGATCATGTCGAGCCGTTTATTGCCCGCACCCTCGGCGCCCACTG

GGACTTTTTCAACGGAGATCCGATGGGATACGGAATCCTCGCTGCGGGTCTTGTGGTGGCTTTCATGATCATCCCGCTTG

TTGCCACGGTGACAAGGGACTCCCTGTTGATGGTTCCCAAAGAGATGGTTGAGGCCTCCTATGCTCTGGGTGCAACCCAG

GTCGAGACATTGCTTTTTGTAAAACTGAAGGCGGTCATCCCCGGGATCATCGGATCGGTGACTCTTGGTTTCGGCCGCGC

TGTCGGCGAGACGATGGCGGTGCTGCTTCTTATCGGAAACCAGGGTTCGATCCCCCAGACGATCCAGGATGTCGGCTATA

CGATCAGCGCCCTTCTGGCCAATACCTTTACCTATGCGGTCATTGATCCCCTGTACTCTGCTGCGGAAGTGGAACTTGGC

CTGATTCTTCTCCTGATCACCCTGGTCATGAATACTCTCGGCCGAAAACTTCTGATGAGACTTATGGGAGGAAGGCTTGC

CGGTTCGGTGGGGGGAGGA

>pstA LfeRT32a_3755 Phosphate transport ABC system

ATGAATCAGGTCAGGGGCCTCTCCCTTTTCAGACGAGCGAGGAACGCTGTATTCGGAACGATTACCCTGCTGGCTTTCGT

CCTCTCGGTCACCCCCCTTCTGGCCATTATCTGGGTCTCCTGGGAAAAAGGAAAAAGTGCGCTGAACCTCAACTTTCTGA

CAACGCTTCCTTCAGGGTTCCCGGTCGGATCTGAAGGCGGGATCCTGAACGGAATCGTCGGGAGTCTCGTTCTTGCGCTT

CTTGCAAGCGCGCTTTCTGTCCCGATCGGAGTCTGGGCCGGAATGTTTCTGTGGGAGGGAGGCAATGACCGTCTTCCATC

GCTGGTCAGGCTGTTCTCAGATCTTCTTCTGGGTGTGCCTTCGGTGGTATGGGGCGTTGTCGGGTATTATGTTTTTTCCA

CGTCAACAGGTTATGGCCTCCACTGGGGATTTTCCGCCCTGGCCGGCGGACTGACCCTTGGGTTCATCATGATCCCGATC

GTCACGAGGGTGACCGAGCAGTCGCTCCGGGATGTTCCCCGATCCTATATTGAAGGGGCGTATGCCCTGGGCGCCACCCG

ATGGCAGACGCTTCGCGGCGTTGCCCTGCCGGGAGCTGTCGCGGGAATCGGGACGGGGATTCTTCTTGGCGTCCTGAATG

TTCTGGGCCAGACGGCACCGCTCGTTTTTACCAACTACTACAATACCGGCATTCCTTCATCCCTTGTTGGCAGCCAGGGG

GCTGTCGGCGATCTCGCCATGCAGATCTTCATCTATATCCATGAACCATCTCCGGAGATGCATACAAAGGCGATGGCGGC

CTCTCTCGTTCTGCTGGTTATTGTGCTGGCTCTTGATCTTGGGATACGCTTTTTGACCTGGGGCGGTCGCAAGTTTTCGG

CCCGGCAGAGA

>pstB LfeRT32a_3754 Phosphate transport ABC system

ATGGAGAGGCTGAGCATCAGAAACTTTTCCGCCTACTATGGTGAAACGCAGGCGCTCAAAAGTGTTTCGATGGAGATTCC

CGACCGGCAGGTCCTGGCGCTGATTGGTCCTTCCGGTTGTGGAAAGACGACCTTTCTCCGGAGCCTTAACCGGATGAACG

ATTTTGTCAAGGATTTCTCGGTCGATGGGCAGGTTCTTCTTGACGGGCAGGACATCTATTCCCCCGAGATGCATCCGGTC

ATTTTAAGGCGCCGTGTTGGAATGGTTTTCCAGAAACCCAATCCTTTTACGAAATCGATCTTTGAGAATGTGGCCTATGG

ACTCAGGATCCAGGGAATCCGCGCAAAGAGCGTCCTGAGAGATGTGGTCGAGAAATCCCTTCAGGCTGCAGCCCTTTGGG

AAGAAGTCAAGGACAGGCTCGACGATTCTGCTTTTGCCCTTTCCGGGGGACAGCAGCAACGTCTCTGCATTGCCCGGGCG

CTTGCCGTTGAGCCGGAAGTGATTCTCCTCGATGAACCGGCATCGGCGCTCGATCCCATTTCAACCGCCCGCATTGAGGA

GTTGATCCAGGAGCTCAAGATCCGTTACACCATCGTGATTGTGACGCATAATATGCAACAGGCCGCTCGTGTTTCCGACC

AGACTGTTTTCTTCCTGAACGGTGAGATGGTCGAGATGGGGGTGACTTCCAAGATTTTTACGACACCGTCGGAGTCGCGG

ACGGAGGATTATATTACCGGGCGGTTCGGT

>tonB LfeRT32a_2204 TonB like biopolymer transport system

ATGAGTACGATTTCCGGGGCAAAAGACCCTTCATTTTCAGACTATATTGAAAATCCGACATTCAGGCTCATGGGACTCCT

GTTTCTCGTGATCCTGATGGAGGGGGGGCTCCTTCTCGCCATCCACATCAACCCACCCCTCGATGTCCCCGATAAAAAAA

TCGAGCCGATCCGAATCACGCTGACCCACCCGCCAGCGCCTCCGAAACCAATTGCCAAGCCGACTCCGCCAAAGCCGGTT

CCTCACCAGATCAAAAAAGTGGTGCGACAGGTTCATCCACACCCTAAAGCAACCCCGCCTGTCCTTCCCGCAGCCAACGC

CACATCCGAAAATCAGCTGGTGGCATCCGTGGGAAACGAGGTTTCTCTGGGCTGGGGCTCTGCGGCACCGGCAAAGGGAA

CACCTTCTGACTTTGTTTCCCCACAGCTTTTGACAAAGGTCGACACCTCCAACTTCTACACGCAGAAGATGAAGGATTCC

GACGAAGAAGGAGATGTCGTCATCGAAGTCTGGGTCGACCCGAAAGGGGCCATCTCTCATTACAAGATGGTTATCCCTTC

CGTTTACGATGACATCAACCGCGTCTCTCTGGGAATTCTGAAAACCCTGAAATTTGCCCCTGCGACCTACAAGGGCAACC

CGGTTGAGGGCCAGTTCCAGCTAAATTTCCGCTTCAGGATCCAGAACAGC

>idh LfeRT32a_2165 NAD(+)-dependent IDH (isocitrate dehydrogenase)

ATGAAAAAGCATGTCATTACGATGTTGCCTGGAGAGGGTACCGGACCGGAAATCTGTGAGGCGGTAAGAAGAGTCATTGA

CCATAGTGGTGTTGACATCACATGGGAATATGAAGAGATCGGTCTCGATTGCCTTAAGGAACATGGCACGCTTTTACCGG

AAAAAACGATCAAATCAATCGCAAAAAACAAGCTTGCCATCAAGGGACCAACGACAACACCTGTCGGCACAGGCCATAAG

AGCGCCAATGTGACCCTCAGAAAGATGTTCGACCTTTATGCAAACGTTCGCCCTGCCAAATTGATCCCTGTCTTGAAGCG

TCCCTGGGACAAAATCGATATCCTGAGCTTCAGGGAAAATACGGAAGATTCTTATGCTGCCATCGAGCATATGGTTTCAG

ACGAAGTTGCCCAGTGTCTTAAGGTTATCACCTGGCCGGGATCCGTTCGTATTGCCGAGTTTGCTTTTAAGTGGGCGAAG

GCGAATAACAGGAAGAAGATTCAGTGCGTTCACAAGGCTAACATCATGAAGATGACCGATGGTCTCTTTCTGGAAGCCTT

CCGTGAAGTTGCAAAGAAATACCCGGAGATTATCGCCGAAGACATCATCGTCGATAACTGTTCAATGCAGCTTGTTCGCA

ATCCAGGTCAGTTTGACTGTCTGGTTCTTCCTAATCTCTACGGAGATATCCTCTCGGATCTTTGTGCTGGATTGGTCGGT

GGTCTTGGTTTCGCCCCTGGTGCCAATATTGGCGATAACTGCGCAATCTTTGAAGCTGTTCACGGTTCCGCTCCAAAGTA

TGCCGGCATGAAAAAAGTGAATCCTTCTGCAGTTCTTCTTTCTGGTGTGATGATGTTGAAGTGGTTGAATGAGCATGAAG

CCGCCACCCGTATTGAAAAAGGTGTGGACAAGGTTTTGGCTGAAGCAAAACATTTGACTTACGATGCAGGCGGGACGGCG

TCTACCGACGAATATGCCGATGCCATCATCAAGGCGATGGAAACCGTT

>porA LfeRT32a_3231 Pyruvate ferredoxin oxidoreductase (POR)

ATGTCAGAAGGTGCTGGGGTAGAGGTTAAATCGACGTCTGCCGGAGCTGGGGTAGTCAAGGATTCTGAAACGTCCGAGGC

CGCCGGGCAGAAAGTTGTTACCCCCGAATATATGTTCTTTGAGGCACCGCGGGAACGGGCGTTCATTACCGGTTCCGAGG

CCGCCAAGGAAGCAATCCGTCGCGCAAACGTGGACGTGGCCATTTCGTACCCGATCACTCCCCAGAGCGAGACGATGCAG

CAGGTAGGTGCTCTCTGGGCTGAGGGATACGTCAAGGAATACTACCGTGGAGAAGAAGAGATCGGTGTGATGTCCGCGAT

CGCAGGTTCTTCCAGGGCAGGGGCCCGTTCGCTGACGGCGACGGCAGGACCAGGCCTCATGCGTGGCATGGAAGTTGTCG

CTTCCTGGCCCGGAGGCAGGATGCCGATCGTTCTTCTGATCATGTGCAGGGTTATCAACGCTCCTCTTTCAATTCAGCCG

GACAACGCCGAACTCGCCTACCTCATCAACACGGGAAACATCGTGTTTCACGCAGAAAATCAGCAGGATTTCTTTGACTA

CACACTGAAGGCCTTCATTATCTCCGAACGTCCCGAAGTGACCCTTCCGGTGGCCGTGGCCGTGGACGGATTCTTCGTGA

CCCATGCCCGCGGGTACGTCATGATGCCATCGAAGGATATCAAGCTTCCGCCAAGGGATCCCTATCATGAAGCCGTTCCG

GTGATGGACAATGAAAATCCGCCAGCCCGTCTTTCGAGGGATGCGCCGATCCAGAAGTCAAACTTCATCAGCTACCATGT

TCATGCCTCCTGGCAGCAGGAAGTCTGGGCAGCGGTCGAGCGCTCCAGAAAATATATCGACATGTATATGGGCGGTCTTG

TGGAGATTGTGAACCCGGATGCCGATACGATCGTGATCGCCTCCGGAAGTGCAGTTTCCCAGTCCAGGGAAGCTGTTCGG

CAGGCAGAGGAAGATCTGGGCGAGCGCATTGGCCTGATCAAGGTCCGCTCCTTGAGACCGTTCCCGACAAAGGAACTCAG

AGAGGCCTGCAAAAACGCAAAAAGAATCATCGTGCCGGAGTTTAATTGCGTAGGATGGCTCTACAGGGATGTCGCCGCTG

CCCTTTATGGACACTCAAAGGCGGAAATTATTCCTGGCCCCCGTGTTTTCGGCGGAATTTCCCTTCCGACGGAAATGATC

CTTCAGTATATTTTCCCGGACAAGAAATTCATCTTC

>porB LfeRT32a_3230 Pyruvate ferredoxin oxidoreductase (POR)

ATGAGCATGGATAATCTTAAGATCTCGCCAGCCCTCGAAAAATATATGACCAAGGAGTACAGGGATCTGGTCGAGCGTGG

CCCTTATGGAAAACAAAAGAAGGTTTCCGAAATGGGAACATTCAAGGAAATCATTGAGGAACATCCGATGTGTGCAGGGT

GTGCGATGACTCTCTTCATCCGTCTGACCATGATCGGCCTTCCAAACCCGGAGCATACCATTCTGGTTGGAACCGCAGGA

TGCGGTAGACTGGCTCTGTCCCAAACATCGATCCCTTTTATCTACGGTAACTACGGAGATACAAATGCTGTGGCTTCCGG

TCTGAAGCGCGGATTGGAAATGCGTTTTCCTGAGCAGCCGAAGGATGTTGTCGTCATGGCAGGTGACGGTGGATTGGCCG

ACATCGGTTTTTCGACCGTGTTGCATTCATGGTTCCGGAAGGAAAAATTCACCACGATCATGCTCGACAATGAAGTTTAC

GGTAACACCGGAGGACAGGAATCCGGAATGACCCAACGCGGTGCAATCCTTAAAATGGCCCCCAAGGGCAAGAAGTACGA

AAAAATGGACATGCTTGGCCTGGCCAAGACCGCCAATGTTCCTTACATCGTCCGCATGACTGTGACCAATCCAACAAGAG

TTGCATCTGTTGTCAGGAAAGCTGTTCTGATTGCCAGAGAAATTGGGCCAACCTTTATTCAGGCCTATACCTCCTGCAAC

ATTGAATATGCCATTCCGACACCAAAAGTGATTGATGATGCCAAGAGCCTTGAAGCAGAGCGATATGGGTTCCAGGAATT

CATTTCTGATGAAGCCAAGGCATTCCTTGAATCAATCGAACCAAAGAAAGCCAAGGCAACCAAATCGACTGAAGCA

>porC LfeRT32a_3229 Pyruvate ferredoxin oxidoreductase (POR)

ATGAACAAGGAACGCTATAACATCAGAATGGCCGGTATCGGCGGTCAGGGAGTTGTCACCGCTTCCCACATTCTCAGTAA

TGGAATGGTGATCATGGGTGGCGAAAGTACGCTCGTGCCGTTTTTCGGTTCAGAAAAACGCCTGGCTCCGGTTGAAAGCT

ATGTCAGAATTGCAAACGGGAAAATCTATGAGATCGGGGAGATCATTTACCCGAACTTGATCATGATCTTCCATCCTCAG

GTCATTACCCATGGCAAGTCCTATACCATGCCTTTCTACTCCGGCTTGAAACCTAACGGAGTGGTGCTGATCAACTCCGA

AACCCCGATCCATCTCGTTGCTGATGAGGAAAGGGAGTTGATGGAGCGGAATGCCAAGGTTTATTACCTTCCGGCCACCC

AGCTCTCAAGAGAGATCGCCGATACGGACCTGGCCACAAATATGGCAATGGTCGGAGCGGTAAGCGCTATTATGGGCATC

CCGGACCTTCCGTCCCTTGAGCAGTCGGTCAAGGAGCGATTCCTTGGAAAGGGATTTGTTGTGTCCGGTGGAACAGCGGC

CCTGGACAACGTTATTGAGCGAAAGTTCGCAAAGAAAGAACAACTGTTGAAGAAGAACATGGAAGTCATTGTTGCCGCTT

ATAATTTTGCAGTCGAACATGGCTGGGCAAAGGTGAAAGAAAAGGTTCAGGTT

>forD2 LfeRT32a_3228 Pyruvate ferredoxin oxidoreductase (POR)

GTGAGCATATGCGATACGATGGCAAAGCATCATGCGATCAAGATGATCATGATCAGCGATCTTGAGAATCCGTTCGAGAT

GAGCAAGGCGGGATTCAATCCGGCGAAGTGGAAAGAACTGCCGGAAACTGTTGTCCTGGAAAAGGTTGCCAAG

>fusA2 LfeRT32a_3570 Elongation factor G

GTGCGTCAGTACCCTCTAGATAAAACCCGAAATATTGGAATCATGGCTCACATTGATGCCGGAAAAACAACGACGACCGA

GAGAATCCTCTTCTATACTGGCATGTTGCACAGGATGGGAGAGGTTCATGACGGAACGACTGTTATGGATTGGATGGATC

AAGAGCGAGAAAGAGGGATAACGATTACCTCTGCAGCTACTTCAGCCGCTTGGAGAGATCATCGGATTAATATTATCGAT

ACTCCTGGTCACGTGGACTTTACTATTGAAGTAGAACGATCGTTGCGGGTTCTTGATGGTGCTGTTGCAGTCTTTGATTC

GGTCCAGGGAGTAGAGCCTCAGTCTGAAACAGTATGGCGTCAAGCTGATAAGTACCGTGTTCCCAGAATAGCCTTCATGA

ACAAGATGGATCGGGTTGGTGCGGACTTTTATGAATCGGTTAAAACCATGGTTGACAGGTTAAAGGCAAACCCGATTCCT

ATACAGATTCCAATCGGTAAAGAGGGAGATTTTGTCGGTGTAGTTGATCTAATCAAGATGAAGGGAATCTACTACGACGA

TGAAACCTTAGGTGCTAAATATGTTGTGAGAGATATTCCGGAAGACCTTATTGAATTGGCTCAATCATACAGAGAGAAGC

TGGTTGAATCTGTCGTTGAGCATCAGGAGAGTCTTACTGAGAAATATCTTAATGGTGAAGAGATCTCCAATGAAGAATTA

GTGGATGGTTTAAGGAAAATTACCGTTTCAATGAAGGGAACTCCAATCCTTTGTGGAGCAGCTTTTAAGAACAAAGGAAT

CCAATTGCTTCTTGATGCGGTTGTTGATTATCTTCCTTCTCCATTAGATGTTCCTGACACTGAGGGGATCGATCCCAAAA

CACAAGAGCCTTCCTTCAGGAAGGCTGCCGATGATGTGCCTTTTTCAGGCTTGGCTTTTAAGATTATGACTGATCCTTTC

GTTGGGCAGCTTACATTTTTTAGGGTTTACTCTGGGAAGCTTGAAGCAGGATCTTATGTTTACAATTCGACTAAAGGGGC

GAAGGAAAGAATCGGCCGTCTCCTTCGGATGCATGCTGATAAAAGGGAAGATATTAAAGAGGTTCTTGCTGGTGATATAG

CTGCTGCTGTTGGCCTTAAGAATACTACAACTGGTGATACTCTTTGTGATGAGGCAAATCCAATTATCTTGGAATTAATC

GATTTTCCTGAACCGGTTATATCTGTGGCAATTGAGCCAAAGACGAAGAGTGATCAAGAAAAACTTGGAATGTCTCTCGG

TAAGCTTGGTCATGAAGATCCGTCCCTTAGAGTGCATACAAATGAAGAAACAGGTCAGACTATCCTTTCAGGAATGGGTG

AACTTCATTTGGAGATTATTGTTGATCGCCTTAAGCGAGAATTCAAGGTGGATGCCAATGTTGGAAAGCCGCAGGTCGCT

TATAGGGAAACGATCACCACTAATACAACCCAAGAAGGAAAATATATTAGACAAACAGGAGGACGTGGGCAATACGGCCA

TGTCGTTTTGGAAATTGAACCTCAAGAAAGAGGCGCTGGTTTTCTGTTCGAGAATAAAATTGTTGGAGGTACGATCCCGA

AAGAGTACATTCCGGCAATTGAAAAAGGCGTTGTAGAGGCGCTTTCTGGAGGTATCATAGCCGGATATCCTGTTGTCGAT

CTTAAGGTAGCAGTTGTTTTTGGATCTTATCATGATGTTGATTCCTCTGAGATGGCTTTTAAAATAGCCGGATCTATGGC

AATCAAGGAAGGTGTTAAAAAATCCAAACCTATCATCTTGGAACCGGTAATGAAGGTAGAGGTTATTGTTCCAGATGAGT

ATATGGGTGATGTCATGGGTGATCTTAATAGCCGCAGAGGTAAGATTTTAGGCATGCATGAACGTTCAGGTGCTCAGGTT

ATTGATGCACATGTACCTCTTGCGGGAATGTTTGGATATGCTACTGACCTCAGGTCGATGACTCAAGGTAGAGGCTTGTT

TACTATGATCTTCTCTTCCTATGAGCCAACTCCAAAGGTAGTCGCTGACGAAATTATGGCAAAAGCGAATTTAGAG

>tuf1 LfeRT32a_3569 Elongation factor F

ATGGCAAAAGCGAAGTTTGATCGAAGCAAGCCCCATCTGAACATCGGAACGATCGGCCATGTCGATCATGGAAAGACGAC

ATTGACGGCAGCGATAACTCGGGTGTTGGCAGCGAACAAGATGGCAGAGTTTTTGGCTTACGACCAGATTGACAAAGCCC

CAGAAGAACGAGAGCGCGGAATCACGATCGCAATTGCCCATGTTGAGTACCAGACATCAGCAAGACATTATGCCCATGTT

GACTGTCCAGGACATGCTGACTACGTGAAAAACATGATCACGGGAGCGGCCCAGATGGACGGAGCTATTTTGGTTGTCTC

GGCTGCGGATGGTCCGATGCCTCAGACGAGAGAGCATATTTTGCTGGCGCGTCAGGTAGGTGTTCCTTATATTGTGGTAT

TCCTGAACAAGGCAGACATGGTGGATGACCCTGAATTACTTGAGCTTGTAGAGCTTGAAGTGAGGGAACTTCTCTCGAAA

TACGATTTTCCTGGAGATGATATTCCTGTGACAAAGGGCTCAGCGTTGAAGGCCTTGGAGTGTGGCTGTGGAAAACCGGA

ATGTCCTGCATGCAGCCCGATTCTGAAGTTGATGCAAACAGTAGACGAGTATATTCCGACACCAACGAGAGATGTTGATA

AACCATTCTTGATGCCGGTAGAAGATGTTTTCTCGATTAGTGGACGAGGAACGGTTGTTACCGGACGAGTTGAGCGTGGT

GTGATCAAGGTAGGCGAAGAAGTGGAAATTGTCGGGATCAGGGACACAGCCAAGTCAGTTGTGACAGGTGTGGAAATGTT

CCGGAAGATTCTGGATTCAGGTCAGGCAGGTGATAATGTTGGATTACTGCTGAGAGGAACAAAGAAAGAGGATGTCGAGC

GTGGAATGGTATTGGCCAAGCCCGGATCGATCACTCCGCATACAGTGTTTGAAGCAGAGGCCTACATATTGACAAAGGAA

GAGGGTGGACGGCATACGCCATTTTTTAATGGATATCGACCCCAGTTCTACTTTAGGACTACGGATGTGACCGGAGTTGT

TACCTTGTCAGAGGGTGTTGAGATGGTGATGCCGGGAGACAATGTTCGCGTTAAGGTGACATTGATCACTCCGATAGCGA

TGGAAGACGGATTGCGGTTCGCGATTCGGGAAGGTGGCCGAACGGTTGGTGCTGGTGTTATCACCAAGGTCGTTCAG

>rplK LfeRT32a_3578 Ribosomal protein L11

ATGGCAAAAGAAATTACTGGGTACGTAAAACTTCAACTTCCTGCGGGAAAAGCGAATCCATCTCCTCCTGTTGGACCTGC

ACTTGGTCAACATGGTGTTAACATCATGGAGTTTTGTAAACAGTTCAATGCAAAAACCCAAGGCCAGGGAGATGCGATTG

TTCCTGTATTAATCACTGTTTATAAAGATAGATCCTTTACATTCGTTATGAAAACCTCGCCTGCTTCTGATCTCCTGAAA

AAGGCCGCTGGAATTCCGAAAGGATCAAAAACTCCCAACAAAGACAAGGTTGCATCATTGTCAAAGGCAAAAATCCTTGA

AATAGCCAAGATCAAACTGGTTGATCTCAATGCTTACGATGTAGAGCATGCGAGCAGGATCATTGAAGGTACCGCAAGAA

GTATGGGTATTACTGTAGAAAAC

>rplA LfeRT32a_3577 Ribosomal protein L1

ATGCCTGAAGGTAAAAAAGTTAAGTTAGCAAAATCAAAAGTAGAAAAAAGACCTTATCTTGTTGATGAGGCTTTGCGGCT

TATCAAGGAAGTCAAGTTTGCAAATTTTGATGAATCTGTAGACTTGGCCGTGAACTTGGGTGTTGACCCAAGACACTCTG

ATCAGATGGTCAGGGGGGCAGTTCTTCTTCCTAATGGTATTGGTAAAACAGTTAAGGTGCTGGCCTTTGCCAAAGGTGAT

AAAGAGAAAGAAGCCCTTCTGGCGGGTGCTGACTATGTTGGTGCAGATGATCTAGTCGCAAAAATCCAGGAAGGTTGGTT

AGACTTTGACACCGTTGTTGCAACTCCTGATTTGATGGTGATGGTCGGAAAGCTTGGTAAGGTCTTGGGTCCTCGTGGAT

TAATGCCTAATCCGAAAACAGGAACCGTTACCTTCGATATTGGCAAAGCTGTCAGGGAGGCTAAGCAGGGAAAGGTAGAA

TTTAAGACTGAAAAAGGAGGCCTTCTTCACTTTCCTATAGGAAGAGCCTCTTTTGCTGAAAATCAGTTGAGAGAAAATCT

TCTTGTGGCTATGTCTGCTATATCAAAGGCAAAACCCCAAACATCAAAAGGGAAGTACATTCAGAAAGCGGTACTTTCCA

CGACCATGGGCCCTGGTGTTTTAATCGACATCAATAGTTTGTTGAAAGAGGTTATC

>acnA LfeRT32a_3441 Aconitase

ATGGCGCTTAATCTGACCCAGAAGATTTTCAAGGAACACCTTGTATCCGGAGAAATTGTTGCCGGAAAAGAAGTTGCCAT

ACGTATCGATCAGACACTCACTCAAGATGCTACCGGTACAATGGCCTATCTTCAGTTTGAAGCACTCGGCCTTGATCGTG

TCAAGACGGAGCTTTCTGTTTCCTATGTTGATCACAACATGTTGCAGACAGGCTTTGAAAACGCTGACGATCACCGATAT

CTTCAGACCGTCGCAGCGCGGTATGGGATTGTTTTTTCCCGTCCCGGAAACGGTATTTGTCATCAGGTTCATCTCGAGCG

ATTTGGCAAGCCAGGGAAAACGCTACTTGGTTCTGACAGTCATACCCCGACAAACGGTGGCGTCGGTATGATTGCCATCG

GGGCAGGAGGTCTTGATGTGGCTCTGGCGATGGGCGGTGAGGCATTTCATCTCACGATGCCAAAGGTTGTCCAGGTTAAG

CTGACGGGACGTCTCCAGCCTTTTGTATCGGCCAAGGATGTCATTCTTGAACTTCTGCGCCGCCTGACGGTCAAGGGAGG

CGTCAACAGAATTTTTGAGTATAACGGTGAAGGTGTGCGATCCCTTTCCGTTCCTGAACGTTCGACGATCACCAATATGG

GTGCCGAGCTTGGTGCAACGACGTCCATTTTTCCGAGCGACGAGAGAACCAGGGAGTATCTTAAGGCACAGGGACGTGAA

AAGGATTATCGGGAACTGGCTGCAGATCCTGGTGCGACATACGACGAAACGGTCGAGATTGACCTCAATACGCTTGAGCC

GCTGATTGCCCAGCCTCACTCTCCGGATAATGTTGTCAAGGTTCGTGATATTCAAGGGATTCCGGTTTCACAGGTTGCGA

TCGGATCCTGCACCAACTCTTCTTTCAAGGATTTGATGACTATCTCCCATCTTATGAAGGGGAAAATGGTTAGTCCCGGA

GTGAGCCTTGGTTTTTCTCCAGGATCGCGTCAGACACTTGAAATGATCTCCCAGAATGGTGTTCTTGGAAACATGATCAC

TGCAGGTGCAAGACTTCTCGAAAGTGCTTGTGGTCCATGCATCGGTATGGGTTTTGCTCCTCCCTCTGGTGGAGTTTCGG

TGCGTACATTCAACCGCAACTTTGAAGGACGAAGTGGCACCAAGGATGCCAAGGTCTATCTGGCCAGCCCTGAAGTTGCC

GCGGCCTGCGCGATTACCGGTGTGATCACAGACCCGAGAGATCTGGGTGTGGAGTTTGAACAGATTGAAATCCCATCCGA

GTACGTTCTTGATGACCGCATGTTTATCTTTCCCCAGGCTGATGGAAAAGACGTTGAGATCATGAGGGGGCCAAACATCA

AGCCTCTTCCGACTCGTGACAGAATTGCTCCAAAAATCAGTGGGAAAGCTCTTCTCAAGGTTGGAGACAATATCACCACG

GATCATATTATGCCTGCTGGTGCGAAGGTTTTGCCTCTTAGATCAAACGTGCCGGCCATCAGTCAGTATGTTTTTGAAGC

AATTGATCCTACCTTCCCCAAAAGGGCAAAGGAAGAGGGCGGTGGCTTTATTATTGGAGGAAATAACTACGGTCAGGGCT

CAAGCCGGGAACATGCGGCTCTTGCACCGATGTATCTTGGTATCAAGGGAGTTATTACGAAGTCGTTTGCCAGAATTCAT

CTGGCTAACCTGATCAACTTCGGTATCCTGCCGCTCACTTTTGTCAATGAGGCTGATTATGACAAGATCGACGCTGGCGA

TGTCCTTGAGCTGGAAACAGCCCATTTGGACAAAAAACCCCTTGCATTGAAAAATGTGACAAAGGGAATCTCCATTCCTG

TGACCCACGTATTAAGCAGCCGCGACCTTGATATTGTTGCAGCCGGTGGGACACTGTCCTATGTAAAGTCTCGAAAATCC

>fdhA LfeRT32a_0628 Putative formate dehydrogenase Fe only (anaerobic)

ATGGTTAAAGTATTTGTGAATGGGGTTGAGGTTGAGGTTGACGCAACCTCAACCATTTTGAAGGCTGCAGAAAAAGCTGG

GGTCGCAATTCCCACTTTTTGTTACCATCCCAGGATGGATCCAGCAGGATCATGTCGAATCTGTGCGGTCGAACTTGAAG

ACTCCAAACGGGTTGTCATGTCCTGTGTGACTCCTGTTGCAGAGGGGATGCGCATTCTTACCGAGTCGGCAAAGGTTGCT

GATGCCAGAAAAACAAATCTTGAACTTCTCCTTTTGCATCATCCTCTGGATTGTCCTGTTTGTGACTGTGGAGGGGAGTG

TCCTCTGCAGAATATGTCGTTTGCCTACGGTGCAAGTGACTCCCGCTTTGAATCACATAGAAATGATGAAGTGGAAGATC

TTAAAAGCGATGTCCTGGTCTTTAACTCCAATCGCTGTATCCTGTGCGGAAAATGCGTCAGGATCTGTGATGAGGTTCAG

GATGTGCATGCCATCGGATTTATCAACCGCGGTTTTGACACGGTCATCGGTCCTCCTCTCGGGAAAAAACTGGATTGTGA

GTTTTGTGGGGATTGTCTTGAAGTTTGTCCGACAGGCGCGATAACGGATAAGTTTGTTCGTTATCAATACCGTCCATGGC

AACTTGAGAAGACCAAGACGACCTGTACCTATTGCGCATCTGGTTGCCAAATGAATGTAGAGACCGAAAAAGAGGCAATC

ATAAGGGTTACCTCCTCCGAAGGAGAAGGTCCAAATGAAGGGTCCATCTGTGCGATAGGTCGATTCGGATTTTCTCACGT

TCAATCCACAGAAAGACTGGACAAACCTTATGCCCGGTCATTTTCCCGACTGGTTCCGACTGAATGGGACTCAGTGATCC

CGGAGATTGCAGAGAGAATCCGTAAAATTGCAGGAACTGGTCCTGACAGGATCGCTACTCTTGTTTCTCCAAGAATTACC

TTGGAAGACGCGTACTTGATCCAAGGTTTCACCCGTAGGGTATTGCGTTCCAACTTTATCGATTCAGGGGCCCGGTATGG

TTTTATGAATGCGGCGTTCCCGATTGCGAGGGCGACGGGAACACTGCGTCCAATGGTTGACCACCAGGATCTTCTTAAAG

CCAAAGTGATTTTGGTGATTGGTGCTGATCCAACCGCTGAGTCCAATATTACAGGTCTTTTTCTGAAACGGGCTATCAGA

AAAGAAAAAGCTCGAATGTATTATGTAGGTTCCGAATCTGTTTCGATCACTGCCCGCGCGAAAGAGCATATAAGGGTTTT

GCCTGGTGGCGAAGGTTTGTTTGCCTGGGTTCTATCGGAAGAGATCGCTGGAGAAGAGTCATCCAGACACCCGGAGTTTC

TCGCAAAAAAAGAAGACCTCTTCAAATCATTCAATGTTGATCCACAGGCTTATACCAAGCTTGTCCAGGACCTGAGAGAT

TCTCCGACCGGAGTCATTATAACCGGGCGAGTATTTCATCGTATCCAGTCTGCATCTGATGCAATGGAGAGTTTGCTCCG

GATGACGAACGCTCTCGGGTGGGTCGAAAAGGAAGGCTGCGGTATTCTTCCTCTGCCAGAGGTGGGGAATGATCTTGGTG

TATTGATGATGGGCGCAACACACGAGTGGCTGCCAGGTCTTCTTGATTCGCAGGATCCTGAAAATAAGAAGAAATGGGAA

GCTACCTGGGGGGAACCTCTTTCCTATGGGAAGGGCGGTGGGTTAAAGGAAATCCTGGAAGGAATCAAGTCAGGAGCTAT

TAAAGGACTCATATCCTTCGGTGAAAATCCGCTCACTCATTTTTCTCCAAATTCTGAAGTTCGTGAAATTCTGTCCAAGC

TTGATTTGCTTGTCTCTGTTGATTTGTTTCAGACTTCATTATCAGATCAGGCACACTTCCTTCTGCCGGCCGCTTCATCC

TATGAGCGCTCGGGGCATGTCGTCAGCGTAGAAGGTTTTGTTCAGGCACTCAATCCGGCGATGACCTATTGGGGTGAATC

ACTCACTGATGGTGAAATTGTTTCCCGTCTCGCCGAGTGCATGGGCGTCCCCTTTGCAACAAGATCAACGGGTGAAGTCT

CGAAAGAGATCTTTACGCTTATGCCCGATCTTTCTCCGTCGACTCGCAATGGAGATCACTTTATGGGGCCGACTGGAAAT

CTCATTCTACCGACAATTGTCAATCAACCTCCTCATAAGGCTGACAGAAAGACAGTAATGAAGAGAATGGCTTCCAGGGC

GGAAAATTGGAAATCTGCTATAACGCCGATACAACCAGAAATCATTTCTGGGGAGGGGGATTTTGTTTTTGCATTGAATA

AATCGCTCTTTCACTCAGGCAAGATGACCCTTCTGGATCCAAGCCTCATGAAAATGGAGTCGTCGGCATTTGTCAGGATC

AATCGCCAGACAGCCAAAAAGTTAAAGATCAAAAAGAACGAGAATGTTCTTATTGAAAATAGCTATGGTTCCTGTATCCT

TCCTGTCGAATTGACATCTGGTGTTACGGAAAATGAATTACAGGTGCCTTATCATTTTGATTCTCCTGACCTGATGGGCT

TGTTTCCCGGAGAGATTAACTATTCTGGCACTTGTTCAACGGTAACAATGTTAAGGACTCGTGTTACCTTGAAAAAGATC

GAG

>sucC LfeRT32a_3443 Succinate dehydrogenase

ATGAAACTTTATGAGCATGAGGCACTCGAGTCAATCTATAAAAAATTTCAGATTCCTTCTCCTAAATTTGTTTTTGGTAC

CGAGCCCAACGAGAAAGTTCGTGAATTTATCGAGAAACAGCCTGCGGTGGTGATCAAGTCCATGGTTCTTGTCGGGAAAA

GGGGCAAGGCAGGTGCGGTCAAGGTGGTAACGGACAAGGCCAAGGCGGTCGAGGTGTTCAAAGACCTTGCCACTCGCGAT

GTTTACGGTGAAAAGTCTGTCGGAGCCATTGTTTGCGAGAAGCTTGATATCCAGAAGGAATACTATCTGAGTGTTACCTA

CTCGACTAAGGAGCGAGCTCCAGCCATTACCTTTAGTGAACATGGTGGAATGGAAGTCGAAGAAATTGACCATAAATTAA

TTCACACATACATTGTTGACGATGTTCGCTCTGTCTACCCATACCAGATCAGAAAGTTTTTGGTCGGCATCGGTTTTTCC

GACCCTGAAATGCTTCGTCCTCTTTCTGAGGTTATCGTAAATGTTTATAAAGCGTTTTGGGAGACAGAGTGCCGTCTTCT

GGAGATCAACCCTCTTGTTGTAGCCAAGGTCGGAGACAAGAAAAAGATTGTTGCGGCCGATGCCGTTGTCCTTCTGGACG

ATGATGCTGCGGTTCCTCCTTCCATCCGTTTTTCCGCAAGAGGCGATATGGGACGGCCTCTTTCCCAGCGGGAACAGGAT

GCAATCTTGATCGACCAGGGAGACCATCGAGGAAAAGCAGGTTCTTATGTGGAGCTTGACGGAGATGTTGCCTTGATGAC

GTTTGGTGGCGGTGGTAGTACCGTGACGGCTGAAACAGCCATTGAAGCTGGTCTTCGTGTGGCGAACCTCACCGATATCG

GGGGGAATCCTCCGGCAGAAAAAATGTACAAGATCGCTCGTATTATTTTGTCAAAACCCGGGCTGAAGGCGGTTCTTGTT

TGTGGTGGAACTGCCAGCAATACGAGAATTGACGTAACGCTTGGAGAAGGTTTGGCCAAGGCGCTGGATGACATGAATGC

CGAGGGGGTCCTGAATAAGGATTTGATCTGGGTTGTTCGTCGAAGCGGTCCGGAGTATGTAAAGGGGCTAAAAATGTTGC

ATGAATGCTTTGTTCGCAACGGGATCAAGGGAGATATTTATGATTCCCAGTTGCCCATTACGGAAGCTCCTCTTCGGCTC

AAAGAGCTTTTGATCAAGCATGTCAATTATCAGCCAGAACAAATTGTC

>sucD LfeRT32a_3442 Succinate dehydrogenase

TTGAAGGGCCCTGTCTATCTTAATGAGAATACTGGCATTGTCGTAATCGGTGCGACCGGAAGGGAAGCATCTCAGGTTAT

CAAGGAGTCTGAAGCTCTTTATCCTGGAATCATCAAGGCTGGAGTTACTCCGGGAAAAGGTGGTTCAACGGAGCTTGCCG

TTCCAGTTTTTGATACCTTGATTCAAGCAAAAAAGGATCCGGCTTTGGGGTCAAAAATCAATACGGCCCTCATCTATGTG

CCACCGGCTTCAGTTCTTGATGCCGTCATGGAATGTCTCGATGCAGAGATCAAGGTGATTTATATCATTACCGAACATGT

TCCTATTCGTGATTCCGAAATCATCTACCAGGAGAAGGTTCGCCGAAACGCCATTATCGTGGGTGGAACCAGTCTTGGCT

GTTTTGTTCCTTCGGTTGGCAGAATCGGCGCTATTGGCGGCAAGGACCCAACGGTCGCTTTTCGTCAGGGCGGTTTGCTG

ATTATCTCCAAAAGCGGTGGTTTGACGGTTACAACGGCGGAGATGTTCAAGCGAAGGGGCTGGGGTACCTATTGTGCCCT

GGCTATCGGAGGGGATATTATAAGCAACACCACCTATGCAGATGTCCTTATGCAAGTGAAGGATGATCCAAACGTAAAGG

GTGTTGTGATGTTGGGGGAACCAGGCGGATCCTATGAAGAGCAGGCTGCCGAGTTGATTACCTCTGGTGTCTTCAAAAAG

CCTGTCGCGGCCTTTATCTCTGGCCGTTTTCAGGAGAGAATGCCTGAAGGCGTGGCCTTTGGTCATGCAGGAGCCATTGT

AGAAAGAGGAATGGGAAAAGCAACTGACAAGATAGAGCGTCTTGATGCGGCGGGAAAGAAGTATCCTGTCAAGGTTGCGT

TCTACTATCATGAGCTTGTTTCTGCGATCGAAAGTCTGGGTGTGCCCAGGGATTTTGAGGACAGTACTTCAGCCCATGTT

GCTCCCCTCTATTCCACAATCAGG
